# Differential Expression Profile of microRNAs and Tight Junction in the Lung Tissues of Rat With Mitomycin-C-Induced Pulmonary Veno-Occlusive Disease

**DOI:** 10.3389/fcvm.2022.746888

**Published:** 2022-02-16

**Authors:** Qing Song, Ping Chen, Shang-Jie Wu, Yan Chen, Yan Zhang

**Affiliations:** ^1^Department of Respiratory and Critical Care Medicine, The Second Xiangya Hospital, Central South University, Changsha, China; ^2^Research Unit of Respiratory Disease, Central South University, Changsha, China; ^3^Diagnosis and Treatment Center of Respiratory Disease, Central South University, Changsha, China

**Keywords:** pulmonary veno-occlusive disease, mitomycin-C, high-throughput sequencing, miRNA, tight junction (TJ)

## Abstract

**Background:**

Pulmonary veno-occlusive disease (PVOD) is characterized by increased pulmonary vascular resistance. Currently, there is a lack of effective treatment. It is of great significance to explore molecular targets for treatment. This study investigated the differential expression profile of miRNAs and tight junction in the lung tissues of rats with mitomycin-C (MMC)-induced PVOD.

**Methods:**

A total of 14 rats were divided into the control group and he PVOD group. We measured mean pulmonary arterial pressure (mPAP) and right ventricular hypertrophy index (RVHI). Pathological changes including those in lung tissues, pulmonary venules, and capillary were detected by H&E and orcein staining. Western blot was used to detect GCN2, ZO-1, occludin, and claudin-5 expression. We analyzed the miRNAs profile in the rat lung tissues by high-throughput sequencing. The top differentially expressed miRNAs were validated by using real-time polymerase chain reaction (RT-PCR).

**Results:**

There were severe pulmonary artery hypertrophy/hyperplasia, thickening, and occlusion in the small pulmonary veins, pulmonary edema, and dilated capillaries in MMC-induced rats with PVOD. In addition, mPAP and RVHI were significantly increased (*P* < 0.05). The expression of GCN2 was significantly decreased (*P* < 0.05). A total of 106 differentially expressed miRNAs were identified. According to the fold changes, the top ten upregulated miRNAs were miRNA-543-3p, miRNA-802-5p, miRNA-493-3p, miRNA-539-3p, miRNA-495, miRNA-380-5p, miRNA-214-5p, miRNA-539-5p, miRNA-190a-3p, and miRNA-431. The top 10 downregulated miRNAs were miRNA-201-3p, miRNA-141-3p, miRNA-1912-3p, miRNA-500-5p, miRNA-3585-5p, miRNA-448-3p, miRNA-509-5p, miRNA-3585-3p, miRNA-449c-5p, and miRNA-509-3p. RT-PCR confirmed that miRNA-214-5p was upregulated, while miRNA-141-3p was downregulated (*P* < 0.05). Functional analysis showed various signaling pathways and metabolic processes, such as fatty acid biosynthesis, tight junction, and the mTOR signaling pathway. In addition, the expression of the tight junction-related protein of ZO-1, occludin, and claudin-5 was significantly decreased in rats with PVOD (*P* < 0.05).

**Conclusion:**

miRNAs may be involved in the pathogenesis of PVOD. Furthermore, ZO-1, occludin, and claudin-5 verification confirmed that the tight junction may be involved in the development of the disease.

## Introduction

Pulmonary veno-occlusive disease (PVOD) is sometimes misdiagnosed with idiopathic pulmonary arterial hypertension (PAH) in the clinic and at least 3–12% of patients with PAH should be diagnosed as PVOD. In addition, PVOD is a highly fatal disease with a mortality rate of 72% within 1 year of diagnosis. Currently, there is a lack of effective treatment except for lung transplantation (1, 2). PVOD is related to a variety of risk factors, such as genetics, smoking, infection, and drugs [mitomycin-C (MMC)], but its pathogenesis is less studied (3). Therefore, in view of its high-fatality rate and lack of effective therapeutic drugs, it is very important to determine the pathogenesis and therapeutic targets.

Perros et al. (4) analyzed the clinical characteristics in seven patients of MMC-induced PVOD and found that all patients had severe hypoxemia and a low-diffusing capacity of the lung for carbon monoxide. Furthermore, right-sided heart catheterization confirmed that the mean pulmonary artery pressure (mPAP) was significantly increased, while a decreased of cardiac index. In addition, high-resolution CT of the chest identified septal lines, centrilobular ground-glass opacities, and lymph enlargement in all patients. What is more, a rat model of MMC-induced PVOD was successfully established by Perros et al. and found that the mPAP, total pulmonary resistance, and the fulton index were increased, and also the severe pulmonary vascular remodeling including smooth muscle cell hypertrophy/hyperplasia in pulmonary arteries, vasculitis of the pulmonary arteries and veins, foci of pulmonary edema and capillaritis, and foci of alveolar wall thickening. In addition, the expression of general control non-derepressible 2 kinase (GCN2) protein was decreased, whose gene is the major one linked to PVOD development and associated with heritability of PVOD.

microRNA (miRNA, miR) is a type of non-coding RNA with regulatory functions and a length of about 22–25 nucleotides. miRNA can regulate genes expression by incomplete or complete direct binding to the mRNA 3'-untranslated region to participate in genes regulation. It plays an important role in regulating genes expression, growth, proliferation and differentiation of cells, and participating in disease development (5, 6). miRNAs have been confirmed as being related to PAH. A study showed that miRNA-124 is down-regulated in a hypoxic PAH mouse model. However, miRNA-21 is upregulated in pulmonary artery smooth muscle cells, which leads to abnormal proliferation and pulmonary vascular remodeling (7–9). However, it is unclear whether miRNA is involved in the development of PVOD.

The tight junction is an important structure through which interactions between multiple-related proteins, maintains endothelial barrier function, and vascular permeability (10). Occludin, claudins, and zonula occludens (ZO) are key members of tight junction proteins. The ZO is an important component of the tight junction which includes ZO-1, ZO-2, and ZO-3 proteins. As a protein complex, the tight junction connects with F-actin through the ZO-1 protein (11). Pulmonary edema is a typical pathological change of PVOD. Studies have shown that the expression of occludin and ZO-1 is significantly decreased in lipopolysaccharide (LPS)-induced mouse, while piceatannol can recover the expression of occludin and ZO-1 and alleviate LPS-induced damage of the air–blood barrier and pulmonary edema (12). In PAH, tight junction destruction can lead to barrier dysfunction of pulmonary artery endothelial cells and promote the progression of the disease (13–15). So, based on the pathological changes of PVOD, we speculated that tight junction might be involved in the pathogenesis of PVOD.

Therefore, the purpose of this study is to investigate the differential expression profile of miRNAs and tight junction in the lung tissues of rats with MMC-induced PVOD.

## Materials and Methods

### Animals

A total of 14 8-week-old male rats with SD were purchased from the SJA Lab Animals Corporation (Hunan, China) and were divided into two groups: the control group (*N* = 4 rats) and the PVOD group (*N* = 10 rats). They were fed in a clean room in the experimental animal center of the second Xiangya Hospital of Central South University (Hunan, China), with the temperature maintained between 20 and 26°C and the humidity at 50–70%. Free access to water and food was provided, with 12 h cycles of light and dark.

This study was approved by the Qinghai Red Cross hospital, Xining, China, 2016, clinical trial number 72, registered on the Chinese Clinical Trial Registry (ChiCTR-TRC-16003142). We declare that all experimental design and analysis involving animal, animal tissues are in comply with the ARRIVE guidelines (16).

### Animal Models

**Figure 1:**
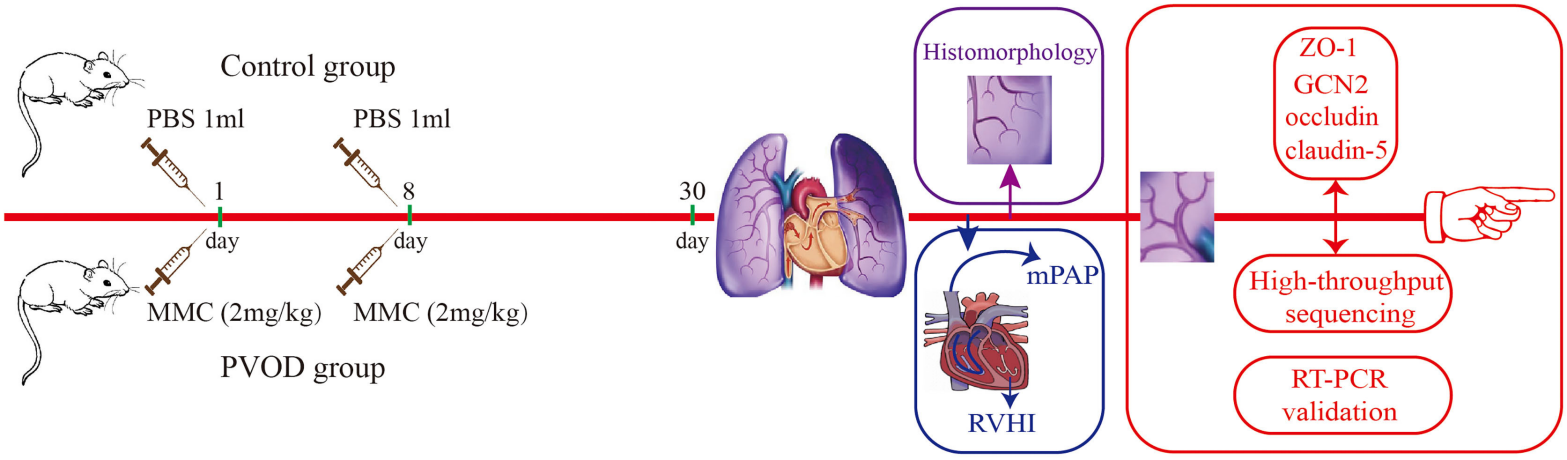
Flowchart of study. The control group (*n* = 4 rats) and the PVOD group (*n* = 10 rats). PVOD, pulmonary veno-occlusive disease; GCN2, General control non-derepressible 2; mPAP, mean pulmonary arterial pressure; MMC, mitomycin-C; PBS, phosphate buffered solution; RVHI, right ventricular hypertrophy index; RT-PCR, real-time polymerase chain reaction; ZO-1, zonula occludens-1.

**Figure 2:**
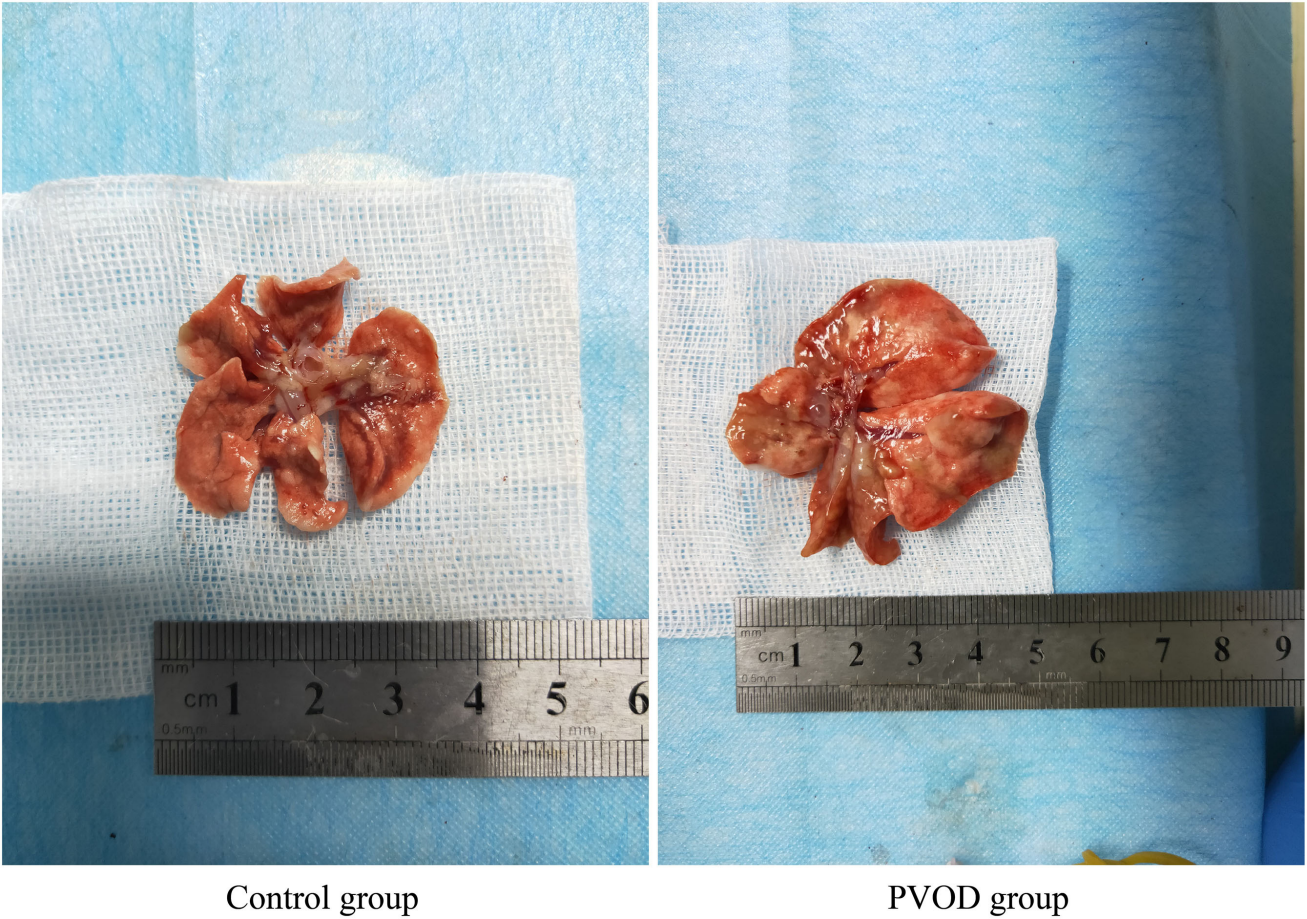
The whole lung tissues in control and MMC-induced rat with PVOD. The lung tissue was significantly swollen, and also severe hyperemia and edema in the PVOD group compared with the control group. The control group (*n* = 4 rats) and the PVOD group (*n* = 10 rats). PVOD, pulmonary veno-occlusive disease.

The rats in the PVOD group were injected intraperitoneally with mitomycin-C (GC12353, GLPBIO, USA) on the first day (2 mg/kg) and eighth days (2 mg/kg), while the rats in the control group were injected intraperitoneally with phosphate-buffered saline at the same doses and times. The experimental period for all groups was 30 days after the animal model was established (Figure 1). First, we measured mPAP by a right heart catheter and used the heart to detect the right ventricular hypertrophy index (RVHI). Then, we removed the bronchi, pulmonary arteriovenous and enclosed lung tissues near the right hilum, and tried to take the lung tissues near the periphery for detection under a dissecting microscope (Figure 2). Finally, the right pulmonary veins along with lung tissues were collected and placed in liquid nitrogen tanks for storage before RNA extraction and western blot, while the left lung tissues were fixed with formalin to observe the structure.

### Measurement of mPAP

Mean pulmonary artery pressure was measured as previously described (17). Briefly, rats were injected intraperitoneally with 1% sodium pentobarbital (40 mg/kg). Then, a PE-50 polyvinyl chloride catheter was slowly inserted through the right external jugular vein and connected with a BL-420 Biological Function Experiment System (AD-Instruments, Oxford, UK). After the catheter was inserted into the pulmonary artery, the position of the catheter was determined by detecting the change of the pressure curve, and the pulmonary artery pressure was recorded.

### Determination of RVHI

After the rats were sacrificed, the whole heart was taken out. The interventricular septum (S), right ventricle (RV), and left ventricle (LV) were separated and the blood was sucked up by filter paper. The weight of the RV and LV + S was weighed. RVHI = RV / (LV + S).

### Histomorphology of Lung Tissue

After rats were dissected, the left lung tissues were inflated with 4% paraformaldehyde at a constant pressure of 25 cm H_2_O and then fixed with 4% paraformaldehyde for 24 h. The lung tissues were embedded in paraffin to fix it, and then sectioned into 4 μm sections. The slices were stained with H&E (Solarbio, China) and orcein (Solarbio, China). Finally, lung tissues histomorphology was observed under an electron microscope.

### Profile of miRNAs by High-Throughput Sequencing

The main steps for the preparation of miRNA libraries as follows. At first, the right lung tissues total RNA was isolated using Trizol reagent (CW0580S, CWBIO, China) and RNA quality was determined by using a Qubit RNA kit (Life Technologies Corporation, CA, USA). Second, the 3' end of RNA was spliced using T4 RNA ligase 2 (0511412, NEB, Herts, UK). Third, RT primer was added to the 3' end ligands for reverse transcription primer hybridization. Fourth, the 5' end of RNA was spliced using T4 RNA ligase 1 (0011309, NEB, Herts, UK). Then, the cDNA strand of the linker was obtained by reverse transcription. Next, the reverse transcription was amplified by PCR. Finally, the cDNA purity was verified with 12% PAGE gelelectrophoresis, and PCR product bands of about 140–150 bp were recovered. We used a Qubit DNA kit to quantify DNA, and then used for library preparation and Illumina sequencing.

Sequencing and analysis of the transcriptome of the samples were performed at Sangon Biotechnology Corporation Ltd. (Shanghai, China). Briefly, in this study, Nextseq 550, SE75 was used to conduct high-throughput sequencing. There were three samples in control group and the raw data of reads count was 9,435,980, 10,052,754, and 12,553,433, respectively. In addition, there were three samples in the PVOD group and the raw data of reads count was 11,540,621, 11,284,053, and 11,420,588, respectively (Supplementary Table 1). For the raw data, we used cutadapt software (version 1.14) to remove the 3'-end connector (sequence: TGGAATTCTCGGGTGCCAAGGAACTC) and set reads after removal of connector length range within 17–35 bp. Reads after removal of connector were processed with trimmomatic software (version 0.36) to delete bases with quality lower than 20 at 5'-end and 3'-ends, and filter out four consecutive bases with average quality lower than 20 and reads with length lower than 17 to obtain clean data (Supplementary Table 2). The comparison of clean reads and rRNA, tRNA, snRNA, and snoRNA in RFAM database were analyzed using blast software (version 2.6.0), and the comparison conditions were set as follows: the value of gapopen is 0, the value of evalue is <0.01, and the value of mismatch is ≤ 1. The species were rat. For the count and normalize reads, the R package was used to process mature miRNA and the number of counts was quantified, and counts were normalized to reads per million. The detailed steps were shown in Supplementary Table 3.

### miRNA Validation

Total RNA was extracted from the right lung tissues using Trizol reagent (CW0580S, CWBIO, China). RNA was reverse transcribed using RevertAid™ First Strand cDNA Synthesis Kit (CW2141, CWBIO, China). Then, the real-time polymerase chain reaction (RT-PCR) was performed with UltraSYBR Mixture (CW2601, CWBIO, China) following the introduction of the manufacturer. The sequences of miRNA-214-5p former primer: 5'- AGAGTTGTCATGTGTCTAAAAA-3' and reverse primer: 5'-GCTGTCAACGATACGCTACGTAA-3'. The miRNA-141-3p former primer: 5'-TCCATCTTCCAGTGCAGTGTTG-3' and reverse primer: 5'-GCTGTCAACGATACGCTACGTAA-3'. The 5S was used as the internal loading control and the former primer: 5'-GCCTACAGCCATACCACCCGGAA-3', reverse primer: 5'-CCTACAGCACCCGGTATCCCA-3'. Each PCR analysis was done in triplicate.

Then, we performed a comprehensive literature search using the online databases PubMed and Embase up to October 2021 to determine whether multiple genes are regulated by miRNA-214-5p and miRNA-141-3p. The terms “miRNA-214-5p” or “miR-214-5p” or “microRNA-214-5p,” and “miRNA-141-3p” or “miR-141-3p” or “microRNA-141-3p” were used to identify the relevant literature. Studies were included if they fulfill these following criteria: (1) English publication; (2) A-identified target genes were validated by dual-luciferase assays.

### Target Gene Prediction

In this study, we used the miRanda algorithm to predict miRNA target genes. miRNA-3'UTR sequence matching and energy stability evaluation were used to comprehensively predict miRNA target genes, and dynamic programming algorithms were used to search for miRNA and 3'UTR complementary and stable double-stranded regions. The threshold for candidate target sites was S ≥ 150, Δ G ≤ −30 kcal/mol and demand strict 5'-seed pairing, where S is the sum of single-residue-pair match scores over the alignment trace and ΔG is the free energy of duplex formation (18).

### Functional Analysis

The Gene Ontology (GO) project provides information on whether the functions of differential genes are significantly enriched in certain functional annotations or pathways (http://www.geneontology.org). The ontology domains analyzed were biological processes, cellular components, and molecular functions. In this study, the purpose genes including differential expression of miRNA and their target mRNA were selected to perform the enrichment analysis of the GO. Pathway analysis is a functional analysis of mapping genes to the Kyoto Encyclopedia of Genes and Genomes (KEGG) pathways. This study selected differentially expressed miRNAs and their target mRNA to perform the GO analysis and the KEGG pathway analysis by using Cluster Profiler software which is an R package for comparing biological themes among gene clusters offers a gene classification method to classify genes based on their projection at a specific level of the GO corpus, and provides functions, enrich GO and enrich KEGG, to calculate enrichment test for the GO terms and the KEGG pathways based on hypergeometric distribution (19).

### Western Blot Analysis

An appropriate size of lung tissue was selected and then the total protein was extracted and measured. The samples were incubated with the primary antibodies for GCN2 (3302S, CST, USA, 1:1000), ZO-1 (21773-1-AP, Proteintech Group, Rosemont, Illinois, USA, 1:5,000), occludin (27260-1-AP, Proteintech Group, Rosemont, Illinois, USA, 1:3000), claudin-5 (35-2,500, Invitrogen, Waltham, Massachusetts, USA, 1:3,000), β-actin (66009-1-Ig, Proteintech Group, Rosemont, Illinois, USA, 1:5,000), and GAPDH (10494-1-AP, Proteintech Group, Rosemont, Illinois, USA, 1:3,000) at 4°C for 24 h. Then, incubated with the secondary antibodies of antirabbit IgG horseradish peroxidase (HRP)-conjugated (SA00001-2, Proteintech Group, Rosemont, Illinois, USA, 1:6,000) for 1 h at normal temperature. Antibody labeling was detected using enhanced chemiluminescence (ECL) (Santa Cruz Biotechnology, Santa Cruz, California, USA).

### Statistical Analysis

Statistical analysis was performed using a software package (SPSS version 26.0, SPSS Incorporation, Chicago, Illinois, USA). Continuous variables were performed using independent sample *t*-test. The data with non-normal distribution or uneven variance were used non-parametric test. A value of *P* < 0.05 was considered to be statistically significant.

## Results

### RVHI, mPAP, and Histomorphology of Lung Tissues

**Figure 3:**
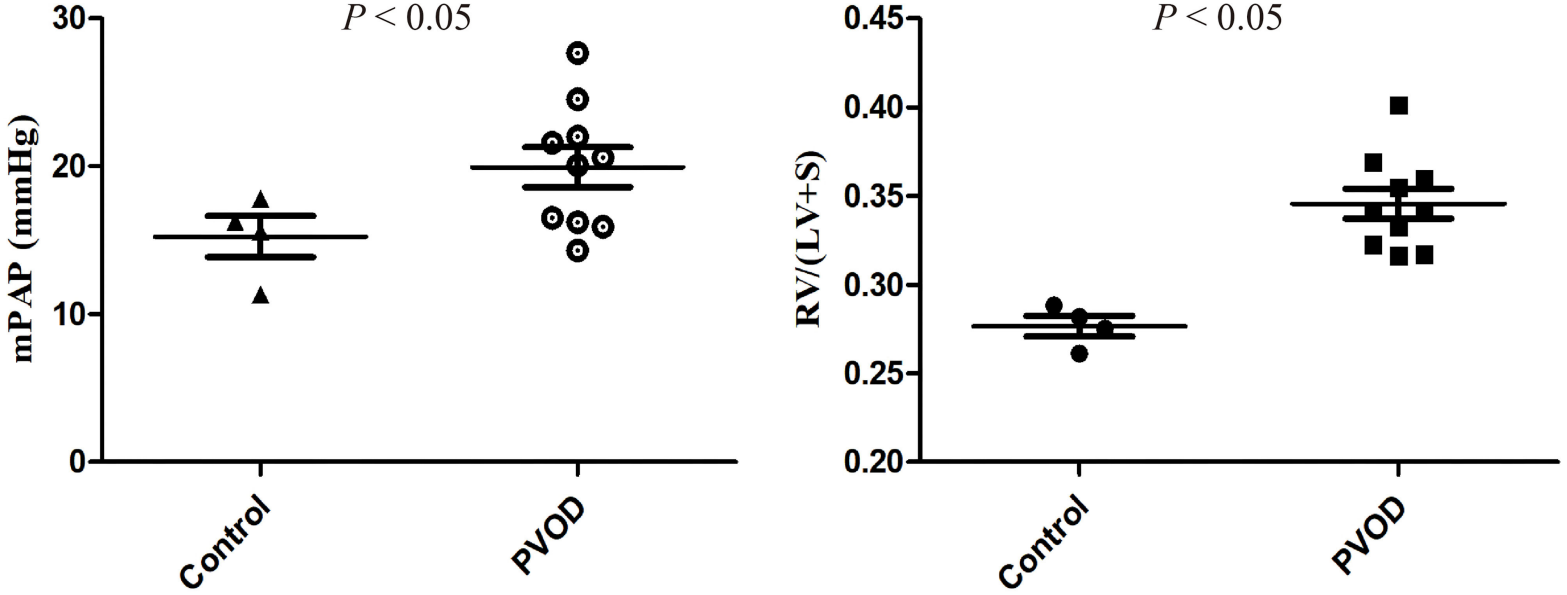
The mPAP and RVHI in control and MMC-induced PVOD rat. The control group (*n* = 4 rats) and the PVOD group (*n* = 10 rats). LV, left ventricular; mPAP, mean pulmonary arterial pressure; MMC, mitomycin-C; PVOD, pulmonary veno-occlusive disease; RVHI, right ventricular hypertrophy index; S, interventricular septum; RVHI = RV/(LV+S).

**Figure 4:**
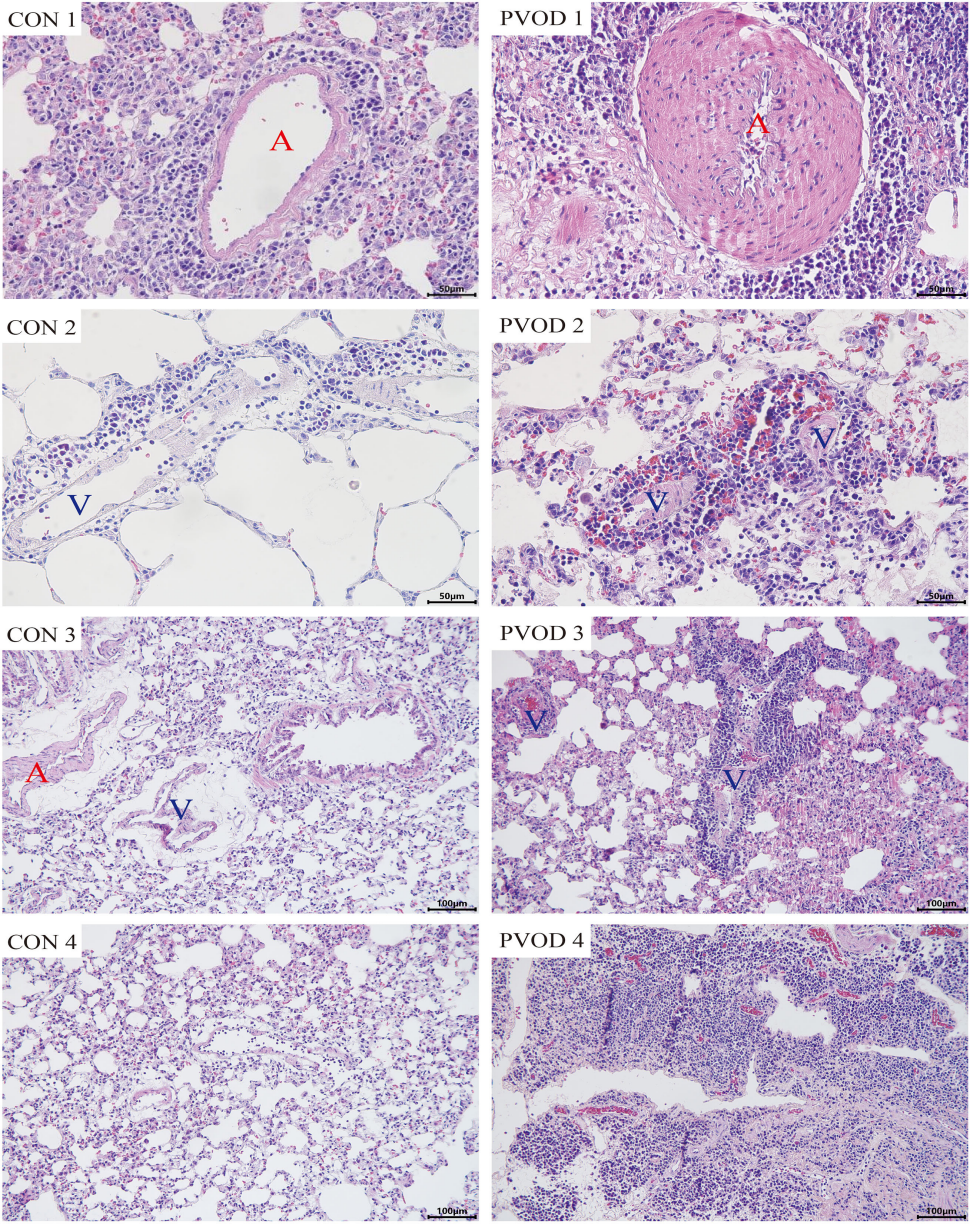
The pathological of lung tissues in control and MMC-induced rat with PVOD with H&E stained. PVOD 1, pulmonary artery with medial hypertrophy/hyperplasia; PVOD 2, vasculitis of pulmonary capillary; PVOD 3, foci of pulmonary edema and capillaritis hemangiomatosis; PVOD 4, alveolar wall thickening. CON 1–4, control lungs; PVOD 1–4, MMC-induced lungs. CON 1–2 and PVOD 1–2: scale bar, 50 μm; CON 3–4 and PVOD 3–4: scale bar, 100 μm. The control group (*n* = 4 rats) and the PVOD group (*n* = 10 rats). A, artery; V, vein; MMC, mitomycin-C; PVOD, pulmonary veno-occlusive disease.

**Figure 5:**
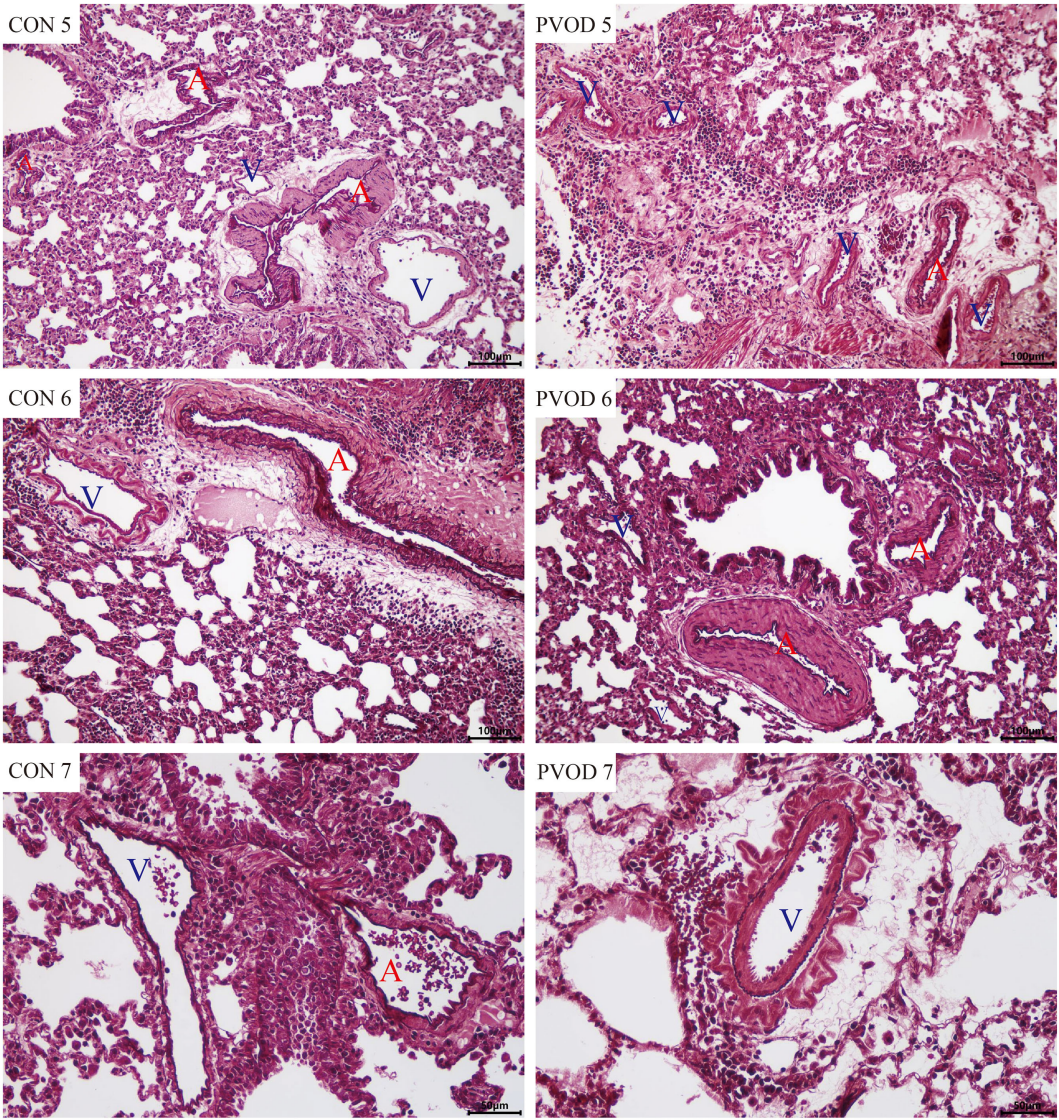
The pulmonary vascular changes in control and MMC-induced rat with PVOD with orcein stained. The pulmonary artery with medial hypertrophy/hyperplasia, thickening, and occlusion in the small pulmonary veins were observed in PVOD group compared with control group. CON 5-7, control lungs; PVOD 5-7, MMC-induced lungs. CON 5-6 and PVOD 5-6: scale bar, 100 μm; CON 7 and PVOD7: scale bar, 50 μm. The control group (*n* = 4 rats) and the PVOD group (*n* = 10 rats). A, artery; V, vein; MMC, mitomycin-C; PVOD, pulmonary veno-occlusive disease.

Compared with the control group, the mPAP and RVHI were significantly increased in rats with PVOD (*P* < 0.05) (Figure 3). In addition, marked pulmonary artery hypertrophy/hyperplasia, thickening and occlusion in small pulmonary veins, pulmonary edema, dilated capillaries, and inflammatory cell infiltrate were observed in rats with PVOD when compared with the control group (Figures 4, 5).

### Expression of GCN2

**Figure 6:**
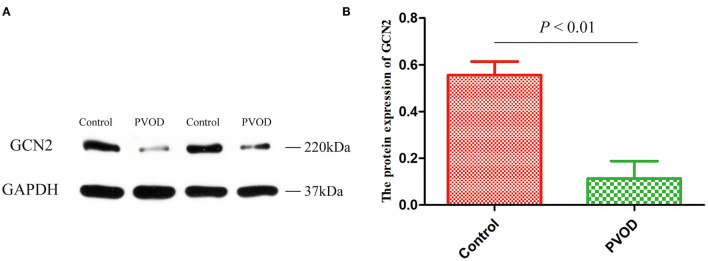
The protein expression of GCN2 in rat. **(A**) Western blot band of proteins expression. **(B)** Relative quantitative of GCN2 expression in the control group and the PVOD group. Values are expressed as means ± SD. GCN2, general control non-derepressible 2; PVOD, pulmonary veno-occlusive disease. The control group (*n* = 4 rats) and the PVOD group (*n* = 10 rats).

Furthermore, we detected the protein expression of GCN2 using western blot analysis. Compared with the control group, the expression of GCN2 was significantly decreased in rats with PVOD (*P* < 0.05) (Figure 6).

### Overview of the miRNA Profiles

**Figure 7:**
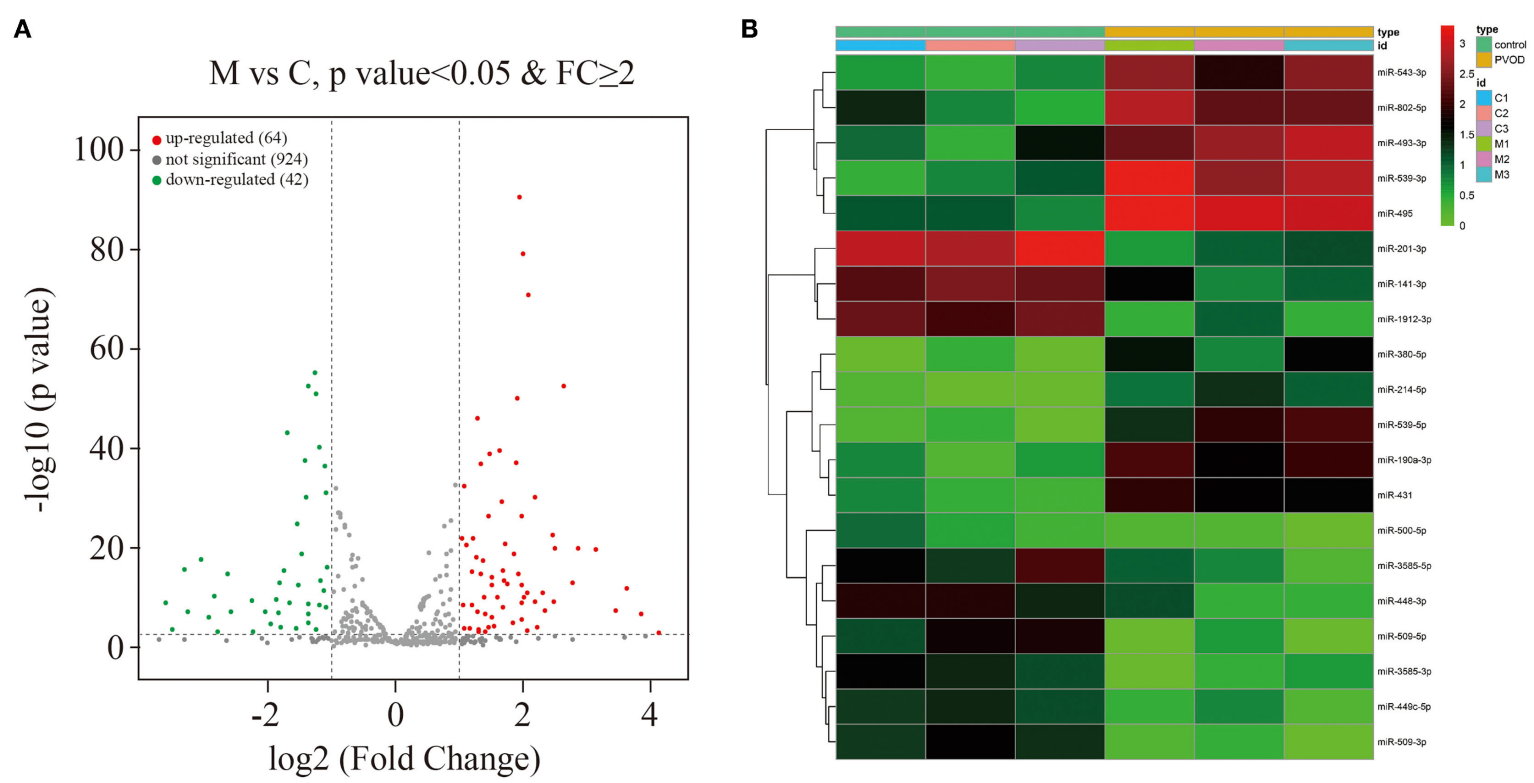
Overview of the miRNA profiles. **(A)** The volcano plot of differential expressed miRNAs in PVOD compared with control group. The upregulated expressed miRNAs were presented in red (≥2-fold change and *P* < 0.05), the downregulated expressed miRNAs were presented in green (≥2-fold change and *P* < 0.05), while no differentially expressed miRNAs were presented in gray (*P* ≥ 0.05). **(B)** Hierarchical clustering showed the top ten upregulated miRNAs and top ten downregulated miRNAs in PVOD group (yellow bar) compared with control group (green bar). The expression was displayed on a scale from light to deep. C, C1–C3 were the control group; M, M1–M3 were the PVOD group. FC, fold change; PVOD, pulmonary veno-occlusive disease.

A total of 1,030 distinct miRNA transcripts were detected. In rats with PVOD, 64 miRNAs were significantly upregulated and 42 were downregulated (≥ 2-fold change and *P* < 0.05) when compared with the control group (Figure 7A). The top ten upregulated miRNAs were miRNA-543-3p, miRNA-802-5p, miRNA-493-3p, miRNA-539-3p, miRNA-495, miRNA-380-5p, miRNA-214-5p, miRNA-539-5p, miRNA-190a-3p and miRNA-431, while the top 10 downregulated miRNAs were miRNA-201-3p, miRNA-141-3p, miRNA-1912-3p, miRNA-500-5p, miRNA-3585-5p, miRNA-448-3p, miRNA-509-5p, miRNA-3585-3p, miRNA-449c-5p, and miRNA-509-3p (Figure 7B).

### miRNAs Validation

**Figure 8:**
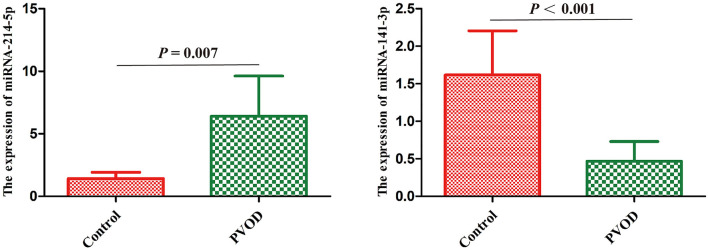
Real-time polymerase chain reaction validated the differential expression of miRNA-214-5p and miRNA-141-3p in rat. Values are expressed as means ± SD. PVOD, pulmonary veno-occlusive disease. The control group (*n* = 4 rats) and the PVOD group (*n* = 10 rats).

According to the results of high-throughput sequencing, the top differentially expressed miRNA-214-5p and miRNA-141-3p that have been shown to be involved in the development of PAH were selected for the validation by using RT-PCT. The result showed that miRNA-214-5p was significantly upregulated, while miRNA-141-3p was downregulated in group with PVOD compared with the control group (*P* < 0.05) (Figure 8).

### Target Gene Prediction

According to the miRanda algorithm, there were 64 upregulated miRNAs with 2,470 target genes, while 42 downregulated miRNAs with 1,098 target genes (Table 1). The detail target genes were showed in Supplementary Tables 4, 5.

**Table 1:** Prediction of number of differential expression miRNAs and their target genes.

**Comparison group**	**Up-regulated miRNAs**	**Target genes**	**Down-regulated miRNAs**	**Target genes**
PVOD vs. Control	64	2,470	42	1,098

*According to the miRanda algorithm to predict the target genes of differential expression miRNAs. PVOD, pulmonary veno-occlusive disease*.

### Gene Ontology Enrichment Analysis

**Figure 9:**
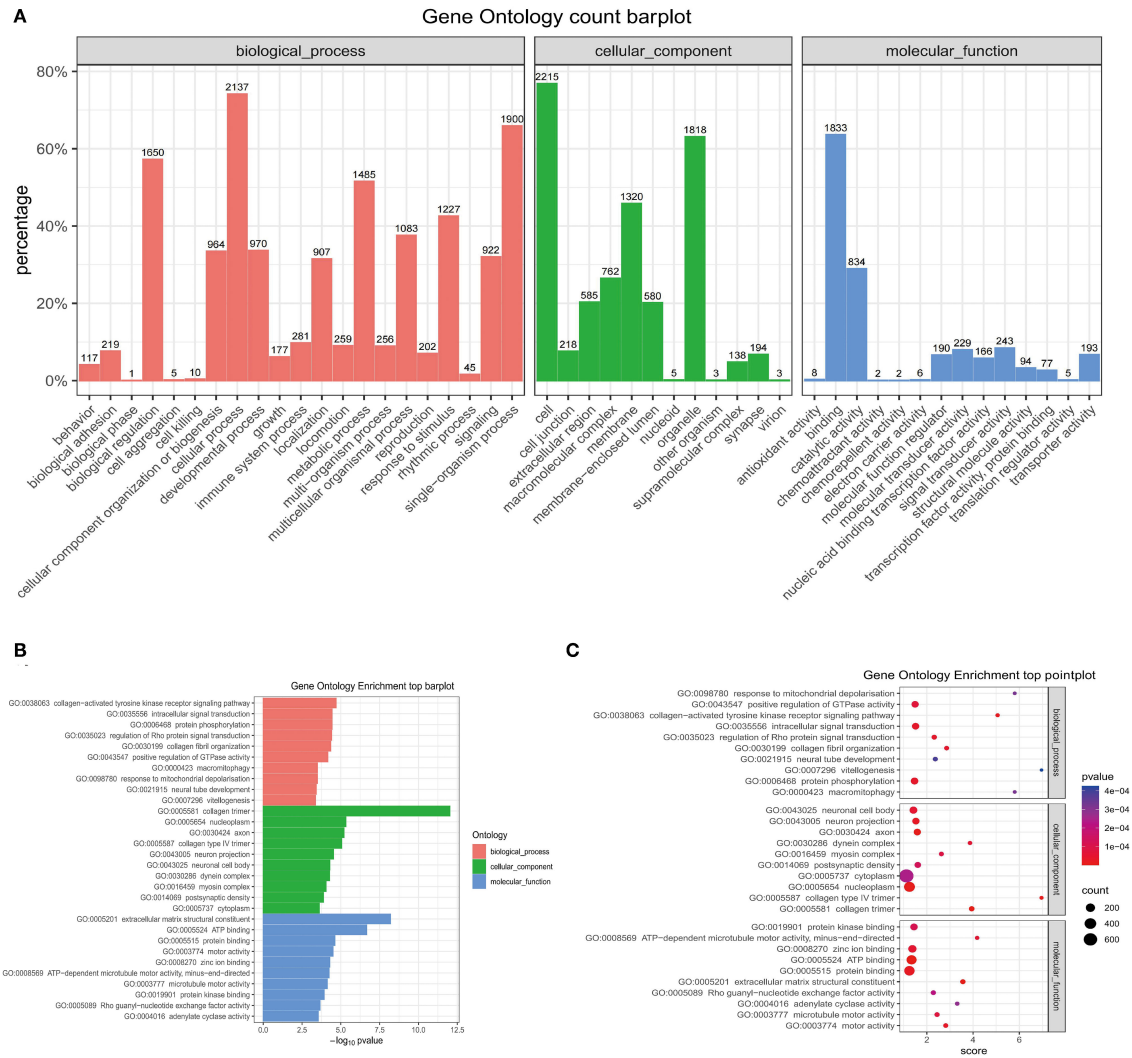
The GO enrichment analysis. **(A)** The barplot of the GO count. **(B)** The barplot of top enrichment in the biological process, cellular component, and molecular function. **(C)** The pointplot of top enrichment in biological process, cellular component, and molecular function. GO, Gene Ontology.

The GO analysis was performed to determine gene product enrichment. For the three ontology domains of biological processes, cellular components and molecular function, the classification of the GO count was showed in Figure 9A. They were enriched in the biological processes including the collagen-activated tyrosine kinase receptor signaling pathway, intracellular signal transduction, and protein phosphorylation. Collagen trimer, nucleoplasm, and axon were enriched in cellular components. In addition, extracellular matrix structural constituent, ATP binding, and protein binding were enriched in the molecular functions (Figures 9B,C).

### Kyoto Encyclopedia of Genes and Genomes Pathway Analysis

**Figure 10:**
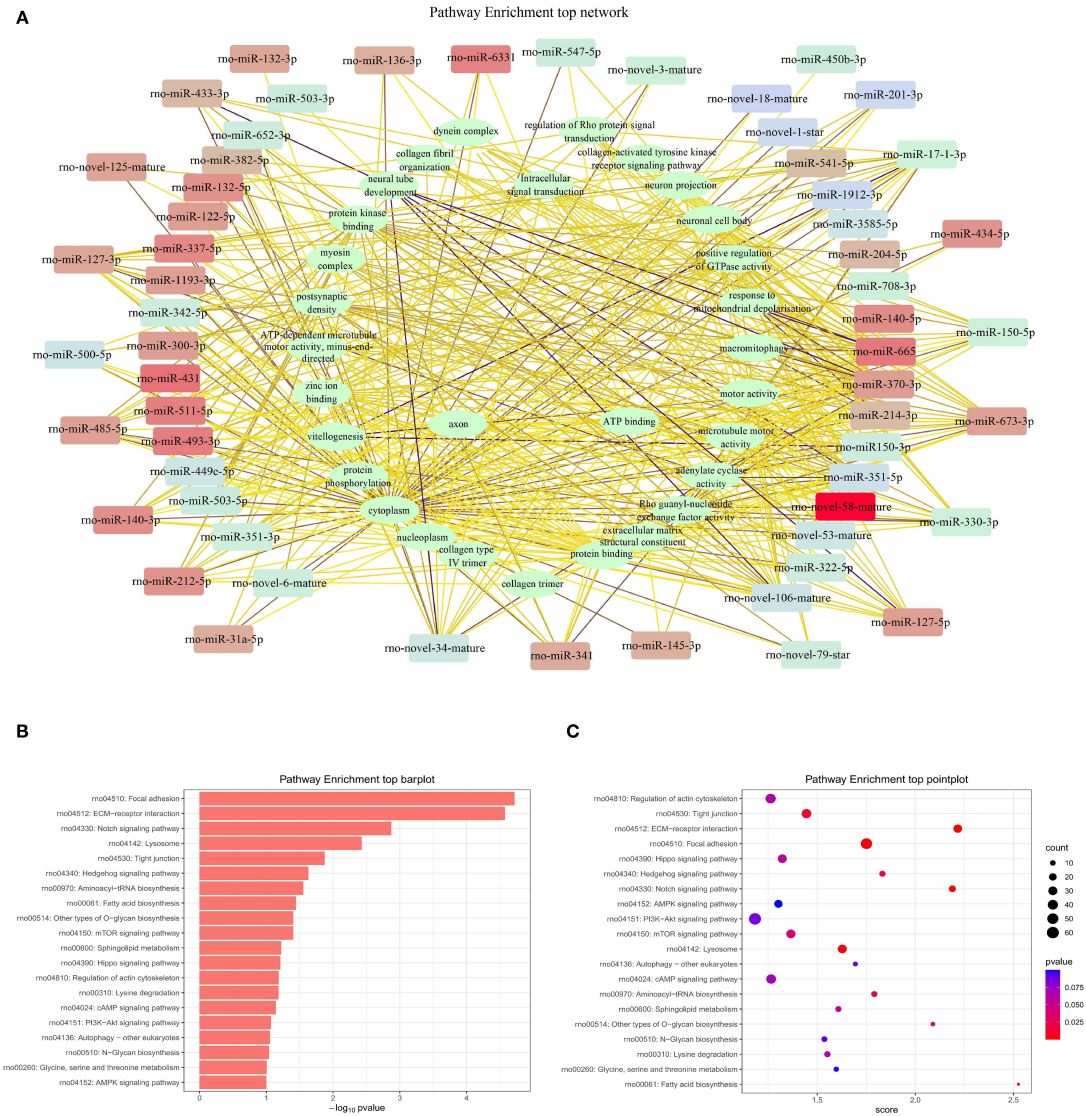
The top pathway enrichment analysis. **(A)** Network of top pathway enrichment. **(B)** Barplot of top pathway enrichment. **(C)** Pointplot of top pathway enrichment.

Pathway analysis demonstrated that there were several enrichment-related pathways. The KEGG pathway analysis involved PVOD-related pathways including focal adhesion, ECM-receptor interaction, notch-signaling pathway, lysosome, tight junction, hedgehog-signaling pathway, aminoacyl-tRNA biosynthesis, fatty acid biosynthesis, other types of O-glycan biosynthesis, and the mTOR-signaling pathway (Figures 10A–C).

### Expression of ZO-1, Occludin, and Claudin-5

**Figure 11:**
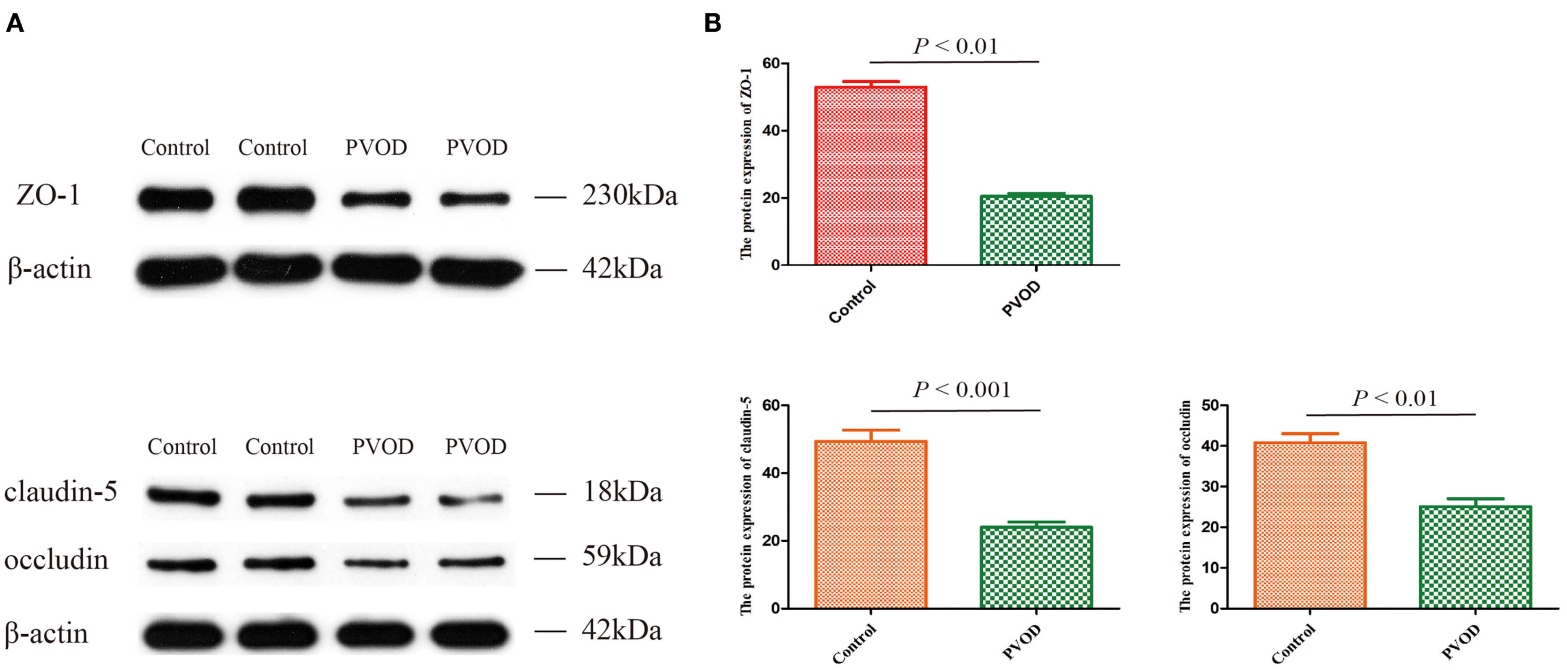
The protein expression of ZO-1, occludin, and claudin-5 in rat. **(A)** Western blot band of proteins expression. **(B)** Relative quantitative of ZO-1, claudin-5, and occludin expression in control group and PVOD group. Values are expressed as means ± SD. ZO-1, zonula occludens-1; PVOD, pulmonary veno-occlusive disease. The control group (*n* = 4 rats) and the PVOD group (*n* = 10 rats).

In order to explore the possible mechanisms of PVOD, we detected expression of the tight junction-related proteins of ZO-1, occludin, and claudin-5 according to the results of pathway analysis and our inference. The proteins expression of ZO-1, occludin, and claudin-5 was significantly decreased in rats with PVOD when compared with the control group (*P* < 0.05) (Figure 11).

## Discussion

Pulmonary veno-occlusive disease is disease with difficult to diagnosis and treatment, of the pathogenesis remains unclear. In this study, we found that the levels of mPAP and RVHI were increased, and there were severe pathological changes including pulmonary artery hypertrophy/hyperplasia, thickening, and occlusion in small pulmonary veins, pulmonary edema, dilated capillaries, and inflammatory cell infiltrate in MMC-induced rat. This was consistent with the study of Perros et al. (4). Therefore, the rat PVOD model was successfully established according to the typical pathological changes, mPAP and RVHI.

GCN2 is a serine-threonine kinase responsible for the phosphorylation of eukaryotic translation initiation factor to regulate the cell cycle and participate in the development of disease. Studies have shown that the mutation of GCN2 is linked to the development of PVOD and the expression of GCN2 is significantly decreased in patients with PVOD and MMC-induced rat pulmonary microvascular endothelial cells (20–22). In addition, Chen et al. (23) found that GCN2 deficiency decreased ATF3-dependent p38 phosphorylation inhibition in development of PVOD. This implies that GCN2 plays an important role in the pathogenesis of PVOD. We also found the same result, that the expression of GCN2 was decreased in rats with PVOD.

miRNA is a type of non-coding RNA with regulatory functions and plays an important role in regulating genes expression and disease development. In addition, miRNA is involved in the regulation of vascular proliferation and remodeling, as well as vascular endothelial cells apoptosis (24). However, whether miRNA is involved in the development of PVOD has not been studied. In this study, we used high-throughput sequencing which is an efficient, rapid method to analyze the differential expression profile of miRNAs between PVOD and normal rats, and screened out a total of 106 miRNAs among 1,030 transcripts that are differentially expressed in the lung tissues. This implies that the profile of these significantly differentially expressed miRNAs might be involved in the development of PVOD. As described in the results, the top upregulated including miRNA-495, miRNA-214-5p, and miRNA-190a-3p and the downregulated miRNAs including miRNA-141-3p, miRNA-509-5p, and miRNA-509-3p. In addition, RT-PCR confirmed that miRNA-214-5p was upregulated, while miRNA-141-3p was downregulated that were consistent with our results of high-throughput sequencing. PVOD is a rare form of PAH. Studies have shown that participates of miRNA in the development of PAH. Stevens et al. (25) found that miRNA-214 is shown to be significantly upregulated in rat models of PAH. In addition, miRNA-214 can promote smooth muscle cell phenotype changes and proliferation and participate in PAH vascular dysfunction (26). This is consistent with our results of sequencing analysis and validation. Fu et al. (27) found that attenuation of miRNA-495 derepresses phosphatase and tensin homolog to effectively protect right ventricular hypertrophy in rats with PAH. Another study showed that miRNA-190 plays an important role in hypoxic pulmonary vascular constriction which participates in the development of PAH (28). Lei et al. (29) found that the expression of miRNA-141 is downregulated in patients with PAH, while enhanced miRNA-141 expression can suppress the RhoA/ROCK pathway to regulate vascular remodeling of PAH. Tang (30) found that the expression of miRNA-509-3p expression decreases in the serum of patients with PAH. This is consistent with our results of high-throughput sequencing. However, the mechanisms by which these differentially expressed miRNAs participated in the development of PVOD need to be further researched.

Studies have shown that miRNAs are involved in the regulation of multiple-signaling pathways and functional analysis of the target genes is helpful for understanding the functional mechanisms of miRNAs (31). In this study, the GO and the KEGG pathway analyses showed that miRNAs are involved in various signaling pathways and metabolic processes, such as the fatty acid biosynthesis, tight junctions, mammalian target of rapamycin (mTOR) signaling pathway, PI3K-AKT signaling pathway, notch signaling pathway, hedgehog signaling pathway, AMPK signaling pathway, and cAMP signaling pathway. Therefore, we have performed a comprehensive literature search using the online databases PubMed and Embase up to October 2021 to determine whether multiple genes are regulated by miRNA-214-5p (Supplementary Table 6) and miRNA-141-3p (Supplementary Table 7).

Studies have shown that tight junctions are involved in the development of PAH (32). The study of Dalvi et al. (33) showed that Tat/cocaine-mediated production of reactive oxygen species activates the Ras/Raf/ERK1/2 pathway that contributes to disruption of the tight junction-related protein ZO-1, leading to pulmonary vascular remodeling and promoting the development of PAH. In our study, we also found significantly decreased expression of the tight junction-related protein of ZO-1, occludin and claudin-5 which might be involved in the development of PVOD. In addition, Lv et al. (34) study showed that miRNA-214 linked with ZO-1 to promote epithelial-mesenchymal transition process that was consistent with our finding.

Early studies have shown that fatty acid biosynthesis plays an important role in PVOD. Ogawa et al. (35) found that epoprostenol, as a metabolite of fatty acids, can be considered as a therapeutic option in patients with PVOD. Another study showed that a remarkable improvement of hemodynamics and of the clinical course of PVOD was produced by adjunctive use of oral sildenafil in association with intravenous high-dose epoprostenol (36). Brittain et al. (37) also found that abnormalities in fatty acid metabolism can be detected in the blood and myocardium in patients with PAH. This is consistent with our pathway prediction analysis results that it is indeed involved in the development of PVOD.

Although these pathways have not been studied in PVOD, they have been shown to be involved in the development of PAH. mTOR is a member of the serine/threonine protein kinase family that regulates cell proliferation, protein synthesis, and actin cytoskeleton; it is the catalytic subunit of two structurally distinct kinase complexes termed TOR complex 1 (TORC1) and TORC2 (38). Studies have shown that mTOR is involved in the occurrence and development of PAH. Inhibition of mTOR can reduce hypoxia-induced right ventricular hypertrophy and remodeling in animals (39) and improve PAH in patients (40). In addition, AKT as the substrate of mTORC2, can activate mTORC1 to involve in the development of disease (41). Jia et al. (42) found that osteoprotegerin induces pulmonary arterial smooth muscle cell proliferation by interacting with integrin αvβ3 to elicit downstream focal adhesion kinase and AKT pathway activation to facilitate PAH pathogenesis. Notch signaling is critically involved in the vascular morphogenesis and function including Notch 1–4. It has been determined that Notch 3 is associated with PAH. The expression of Notch 3 is increased in the lung tissues of patients with PAH and hypoxia-induced mouse with PAH (43). mTOR has previously been identified as a positive regulator of Notch 3 (44).

Hedgehog is a proangiogenic factor involved in the regulation of endothelial cell proliferation. Ghouleh et al. (45) found that the expression of hedgehog is increased in hypoxia-induced pulmonary artery endothelial cells and involved in the progression of PAH. Adenosine monophosphate-activated protein kinase (AMPK), a sensor of cellular energy, has been found to regulate cell proliferation (46). Studies have suggested that activation of AMPK by metformin prevents the development of PAH in animal models and activation of AMPK prevents the development of PAH by targeting nuclear factor-kappa B (NF-κB) to suppress autophagy and vascular remodeling (47, 48). cAMP signaling is involved in extracellular matrix metabolism as well as in proliferation control. The expression of p21(Waf1/Cip1) is regulated by C/EBP-α, which in turn is controlled by cAMP. Similarly, treprostinil, through cAMP-C/EBP-α p42-p21 (WAf1/Cip1) signaling, reduces arterial wall remodeling which benefits patients with PAH (49–51). So, the molecular mechanisms in PVOD are worth further exploration.

This study still has potential limitations. First, all the RNA and protein were extracted using the lung tissues, not pulmonary veins. In fact, it is also very difficult to separate small pulmonary veins and the capillaries also had serious pathological changes in rats with PVOD. In addition, in this study, we used lung tissues to perform high-throughput sequencing and validate the differentially expressed miRNAs so that it is unclear whether the results were from endothelial cells or smooth muscle cells. Then, not all of the differentially expressed miRNAs in rat with PVOD were validated by RT-PCR that might decrease the accuracy. Finally, we found that various signaling pathways might be involved in the development of PVOD by enrichment analysis, but we just detected the expression of tight junction protein of ZO-1, occludin, and claudin-5 which is consistent with the result of pathways enrichment analysis. Surely, we will further validate other differentially expressed miRNAs and molecular pathways to reveal their specific mechanisms of PVOD in the future.

In summary, we screened the expression profile of miRNAs closely related to PVOD by using high-throughput sequencing. In addition, the GO and the KEGG pathway analyses could help us to explored and predicted the function of certain miRNAs, which would provide a new experimental basis and ideas for further research on the effect of miRNAs on the occurrence and development of PVOD in future. In addition, the tight junction protein ZO-1, occludin, and claudin-5 might be related to be PVOD, which confirmed the GO and KEGG pathway analyses, but the underlying mechanisms need to be explored further.

## Data Availability Statement

The datasets presented in this study can be found in online repositories. The names of the repository/repositories and accession number(s) can be found at: https://www.ncbi.nlm.nih.gov/geo/query/acc.cgi?acc=GSE189080.

## Ethics Statement

The animal study was reviewed and approved by Qinghai Red Cross Hospital. Written informed consent was obtained from the owners for the participation of their animals in this study.

## Author Contributions

QS performed the data analysis, drafted manuscript, and drew the figures. PC, YC, and S-JW coordinated the study and performed the experiment. YZ conceived the study, performed the experiment, and revised the manuscript. All the authors read and approved the final version of the manuscript.

## Funding

This study was supported by the Major Scientific and Technological Achievement Transformation Project of Qinghai Province, China [2017-SF-122].

## Conflict of Interest

The authors declare that the research was conducted in the absence of any commercial or financial relationships that could be construed as a potential conflict of interest.

## Publisher's Note

All claims expressed in this article are solely those of the authors and do not necessarily represent those of their affiliated organizations, or those of the publisher, the editors and the reviewers. Any product that may be evaluated in this article, or claim that may be made by its manufacturer, is not guaranteed or endorsed by the publisher.

## References

[1] Montani D Lau EM Dorfmüller P Girerd B Jaïs X Savale L. Pulmonary veno-occlusive disease. Eur Respir J. (2016) 47:1518–34. 10.1183/13993003.00026-2016 27009171

[2] Holcomb BW Jr Loyd JE Ely EW Johnson J Robbins IM. Pulmonary veno-occlusive disease: a case series and new observations. Chest. (2020) 118:1671–9. 10.1378/chest.67.4.487 11115457

[3] Certain MC Chaumais MC Jaïs X Savale L Seferian A Parent F. Characteristics and long-term outcomes of pulmonary venoocclusive disease induced by mitomycin C. Chest. (2021) 159:1197–207. 10.1016/j.chest.2020.09.238 32979348

[4] Perros F Günther S Ranchoux B Godinas L Antigny F Chaumais MC. Mitomycin-induced pulmonary veno-occlusive disease: evidence from human disease and animal models. Circulation. (2015) 132:834–47. 10.1161/CIRCULATIONAHA.115.014207 26130118

[5] O'Brien J Hayder H Zayed Y Peng C. Overview of microRNA biogenesis, mechanisms of actions, and circulation. Front Endocrinol. (2015) 9:402. 10.3389/fendo.2018.00402 30123182 PMC6085463

[6] Yan S Shi J Sun D Lyu L. Current insight into the roles of microRNA in vitiligo. Mol Biol Rep. (2020) 47:3211–9. 10.1007/s11033-020-05336-3 32086720

[7] Li Q Qian Z Wang L. Pri-microRNA-124 rs531564 polymorphism minor allele increases the risk of pulmonary arteryhypertension by abnormally enhancing proliferation of pulmonary artery smooth muscle cells. Int J Chron Obstruct Pulmon Dis. (2017) 12:1351–61. 10.2147/COPD.S99318 28496318 PMC5422315

[8] Green DE Murphy TC Kang BY Searles CD Hart CM. PPARγ ligands attenuate hypoxia-induced proliferation in human pulmonary artery smooth muscle cells through modulation of microRNA-21. PLoS ONE. (2015) 10:e0133391. 10.1371/journal.pone.0133391 26208095 PMC4514882

[9] Green DE Murphy TC Kang BY Bedi B Yuan Z Sadikot RT. Peroxisome proliferator-activated receptor-γ enhances human pulmonary artery smooth muscle cell apoptosis through microRNA-21 and programmed cell death 4. Am J Physiol Lung Cell Mol Physiol. (2017) 313:L371–83. 10.1152/ajplung.00532.2016 28522568 PMC5582937

[10] Kakogiannos N Ferrari L Giampietro C Scalise AA Maderna C Ravà M. JAM-A acts *via* C/EBP-α to promote claudin-5 expression and enhance endothelial barrier function. Circ Res. (2020) 127:1056–73. 10.1161/CIRCRESAHA.120.316742 32673519 PMC7508279

[11] Feng S Zou L Wang H He R Liu K Zhu H. RhoA/ROCK-2 pathway inhibition and tight junction protein upregulation by catalpol suppresses lipopolysaccaride-induced disruption of blood-brain barrier permeability. Molecules. (2018) 23:2371. 10.3390/molecules23092371 30227623 PMC6225311

[12] Peng LY Yuan M Shi HT Li JH Song K Huang JN. Protective effect of piceatannol against acute lung injury through protecting the integrity of air-blood barrier and modulating the TLR4/NF-κB signaling pathway activation. Front Pharmacol. (2020) 10:1613. 10.3389/fphar.2019.01613 32038265 PMC6988518

[13] Good RB Gilbane AJ Trinder SL Denton CP Coghlan G Abraham DJ. Endothelial to mesenchymal transition contributes to endothelial dysfunction in pulmonary arterial hypertension. Am J Pathol. (2015) 185:1850–8. 10.1016/j.ajpath.2015.03.019 25956031

[14] Fazakas C Nagaraj C Zabini D Végh AG Marsh LM Wilhelm I. Rho-kinase inhibition ameliorates dasatinib-induced endothelial dysfunction and pulmonary hypertension. Front Physiol. (2018) 9:537. 10.3389/fphys.2018.00537 29867576 PMC5962749

[15] Dasgupta SK Le A Vijayan KV Thiagarajan P. Dasatinib inhibits actin fiber reorganization and promotes endothelial cell permeability through RhoA-ROCK pathway. Cancer Med. (2017) 6:809–18. 10.1002/cam4.1019 28316141 PMC5387130

[16] Percie du Sert N Hurst V Ahluwalia A Alam S Avey MT Baker M. The ARRIVE guidelines 2.0: updated guidelines for reporting animal research. PLoS Biol. (2020) 18:e3000410. 10.1371/journal.pbio.3000410 32663219 PMC7360023

[17] Deng C Zhong Z Wu D Chen Y Lian N Ding H. Role of FoxO1 and apoptosis in pulmonary vascular remolding in a rat model of chronic thromboembolic pulmonary hypertension. Sci Rep. (2017) 7:2270. 10.1038/s41598-017-02007-5 28536427 PMC5442111

[18] Enright AJ John B Gaul U Tuschl T Sander C Marks DS. MicroRNA targets in Drosophila. Genome Biol. (2003) 5:R1. 10.1186/gb-2003-5-1-r1 14709173 PMC395733

[19] Yu G Wang LG Han Y He QY. ClusterProfiler: an R package for comparing biological themes among gene clusters. OMICS. (2012) 16:284–7. 10.1089/omi.2011.0118 22455463 PMC3339379

[20] Zhang C Lu W Luo X Liu S Li Y Zheng Q. Mitomycin C induces pulmonary vascular endothelial-to-mesenchymal transition and pulmonary veno-occlusive disease via Smad3-dependent pathway in rats. Br J Pharmacol. (2021) 178:217–35. 10.1111/bph.15314 33140842

[21] Lai YJ Chen PR Huang YL Hsu HH. Unique wreath-like smooth muscle proliferation of the pulmonary vasculature in pulmonary veno-occlusive disease versus pulmonary arterial hypertension. J Formos Med Assoc. (2020) 119:300–9. 10.1016/j.jfma.2019.05.019 31202500

[22] Nossent EJ Antigny F Montani D Bogaard HJ Ghigna MR Lambert M. Pulmonary vascular remodeling patterns and expression of general control nonderepressible 2 (GCN2) in pulmonary veno-occlusive disease. J Heart Lung Transplant. (2018) 37:647–55. 10.1016/j.healun.2017.09.022 29108819

[23] Chen Z Zhang J Wei D Chen J Yang J. GCN2 regulates ATF3-p38 MAPK signaling transduction in pulmonary veno-occlusive disease. J Cardiovasc Pharmacol Ther. (2021) 26:677–89. 10.1177/10742484211015535 33988041

[24] Song Q Chen P Liu XM. The role of cigarette smoke-induced pulmonary vascular endothelial cell apoptosis in COPD. Respir Res. (2021) 22:39. 10.1186/s12931-021-01630-1 33546691 PMC7866753

[25] Stevens HC Deng L Grant JS Pinel K Thomas M Morrell NW. Regulation and function of miR-214 in pulmonary arterial hypertension. Pulm Circ. (2016) 6:109–17. 10.1086/685079 27162619 PMC4860547

[26] Sahoo S Meijles DN Al Ghouleh I Tandon M Cifuentes-Pagano E Sembrat J. MEF2C-MYOCD and leiomodin1 suppression by miRNA-214 promotes smooth muscle cell phenotype switching in pulmonary arterial hypertension. PLoS ONE. (2016) 11:e0153780. 10.1371/journal.pone.0153780 27144530 PMC4856285

[27] Fu J Chen Y Li F. Attenuation of microRNA-495 derepressed PTEN to effectively protect rat cardiomyocytes from hypertrophy. Cardiology. (2018) 139:245–54. 10.1159/000487044 29566365

[28] Li SS Ran YJ Zhang DD Li SZ Zhu D. MicroRNA-190 regulates hypoxic pulmonary vasoconstriction by targeting a voltage-gated K channel in arterial smooth muscle cells. J Cell Biochem. (2014) 115:1196–205. 10.1002/jcb.24771 24446351

[29] Lei S Peng F Li ML Duan WB Peng CQ Wu SJ. LncRNA-SMILR modulates RhoA/ROCK signaling by targeting miR-141 to regulate vascular remodeling in pulmonary arterial hypertension. Am J Physiol Heart Circ Physiol. (2020) 319:H377–91. 10.1152/ajpheart.00717.2019 32559140

[30] Tang P. Clinical diagnostic value of circulating serum miR-509-3p in pulmonary arterial hypertension with congenital heart disease. Hellenic J Cardiol. (2020) 61:26–30. 10.1016/j.hjc.2018.06.004 29890280

[31] Bartel DP. MicroRNAs: genomics, biogenesis, mechanism, and function. Cell. (2004) 116:281–97. 10.1016/S0092-8674(04)00045-5 14744438

[32] Dhillon NK Li F Xue B Tawfik O Morgello S Buch S. Effect of cocaine on human immunodeficiency virus-mediated pulmonary endothelial and smooth muscle dysfunction. Am J Respir Cell Mol Biol. (2011) 45:40–52. 10.1165/rcmb.2010-0097OC 20802087 PMC3145070

[33] Dalvi P Wang K Mermis J Zeng R Sanderson M Johnson S. HIV-1/cocaine induced oxidative stress disrupts tight junction protein-1 in human pulmonary microvascular endothelial cells: role of Ras/ERK1/2 pathway. PLoS ONE. (2014) 9:e85246. 10.1371/journal.pone.0085246 24409324 PMC3883699

[34] Lv JW Wen W Jiang C Fu QB Gu YJ Lv TT. Inhibition of microRNA-214 promotes epithelial-mesenchymal transition process and induces interstitial cystitis in postmenopausal women by upregulating Mfn2. Exp Mol Med. (2017) 49:e357. 10.1038/emm.2017.98 28729638 PMC5565960

[35] Ogawa A Miyaji K Yamadori I Shinno Y Miura A Kusano KF. Safety and efficacy of epoprostenol therapy in pulmonary veno-occlusive disease and pulmonary capillary hemangiomatosis. Circ J. (2012) 76:1729–36. 10.1253/circj.CJ-11-0973 22481098

[36] Kuroda T Hirota H Masaki M Sugiyama S Oshima Y Terai K. Sildenafil as adjunct therapy to high-dose epoprostenol in a patient with pulmonary veno-occlusive disease. Heart Lung Circ. (2006) 15:139–42. 10.1016/j.hlc.2005.07.002 16574537

[37] Brittain EL Talati M Fessel JP Zhu H Penner N Calcutt MW. Fatty acid metabolic defects and right ventricular lipotoxicity in human pulmonary arterial hypertension. Circulation. (2016) 133:1936–44. 10.1161/CIRCULATIONAHA.115.019351 27006481 PMC4870107

[38] Betz C Hall MN. Where is mTOR and what is it doing there? J Cell Biol. (2013) 203:563–74. 10.1083/jcb.201306041 24385483 PMC3840941

[39] Pena A Kobir A Goncharov D Goda A Kudryashova TV Ray A. Pharmacological inhibition of mTOR kinase reverses right ventricle remodeling and improves right ventricle structure and function in rats. Am J Respir Cell Mol Biol. (2017) 57:615–25. 10.1165/rcmb.2016-0364OC 28679058 PMC5705904

[40] Wessler JD Steingart RM Schwartz GK. Dramatic improvement in pulmonary hypertension with rapamycin. Chest. (2010) 138:991–3. 10.1378/chest.09-2435 20923803

[41] Memmott RM Dennis PA. Akt-dependent and -independent mechanisms of mTOR regulation in cancer. Cell Signal. (2009) 21:656–64. 10.1016/j.cellsig.2009.01.004 19166931 PMC2650010

[42] Jia D Zhu Q Liu H Zuo C He Y Chen G. Osteoprotegerin disruption attenuates HySu-induced pulmonary hypertension through integrin αvβ3/FAK/AKT pathway suppression. Circ Cardiovasc Genet. (2017) 10:e001591. 10.1161/CIRCGENETICS.116.001591 28077433

[43] Morris HE Neves KB Montezano AC MacLean MR Touyz RM. Notch3 signalling and vascular remodelling in pulmonary arterial hypertension. Clin Sci. (2019) 133:2481–98. 10.1042/CS20190835 31868216 PMC6928565

[44] Ma J Meng Y Kwiatkowski DJ Chen X Peng H Sun Q. Mammalian target of rapamycin regulates murine and human cell differentiation through STAT3/p63/Jagged/Notch cascade. J Clin Invest. (2010) 120:103–14. 10.1172/JCI37964 20038814 PMC2798675

[45] Ghouleh IA Sahoo S Meijles DN Amaral JH de Jesus DS Sembrat J. Endothelial Nox1 oxidase assembly in human pulmonary arterial hypertension; driver of Gremlin1-mediated proliferation. Clin Sci. (2017) 131:2019–35. 10.1042/CS20160812 28522681 PMC5705051

[46] Grahame Hardie D. AMP-activated protein kinase: a key regulator of energy balance with many roles in human disease. J Intern Med. (2014) 276:543–59. 10.1111/joim.12268 24824502 PMC5705060

[47] Li S Han D Zhang Y Xie X Ke R Zhu Y. Activation of AMPK prevents monocrotaline-induced extracellular matrix remodeling of pulmonary artery. Med Sci Monit Basic Res. (2016) 22:27–33. 10.12659/MSMBR.897505 26978596 PMC4795089

[48] Zhai C Shi W Feng W Zhu Y Wang J Li S. Activation of AMPK prevents monocrotaline-induced pulmonary arterial hypertension by suppression of NF-κB-mediated autophagy activation. Life Sci. (2018) 208:87–95. 10.1016/j.lfs.2018.07.018 30009823

[49] Lambers C Qi Y Eleni P Costa L Zhong J Tamm M. Extracellular matrix composition is modified by β_2_-agonists through cAMP in COPD. Biochem Pharmacol. (2014) 91:400–8. 10.1016/j.bcp.2014.07.026 25107701

[50] Lambers C Costa L Ying Q Zhong J Lardinois D Dekan G. Aclidinium bromide combined with formoterol inhibits remodeling parameters in lung epithelial cells through cAMP. Pharmacol Res. (2015) 102:310–8. 10.1016/j.phrs.2015.09.010 26546746

[51] Lambers C Kornauth C Oberndorfer F Boehm PM Tamm M Klepetko W. Mechanism of anti-remodelling action of treprostinil in human pulmonary arterial smooth muscle cells. PLoS ONE. (2018) 13:e0205195. 10.1371/journal.pone.0205195 30383775 PMC6211661

