# Supplementary material for: Differential Expression Profile of microRNAs and Tight Junction in the Lung Tissues of Rat With Mitomycin-C-Induced Pulmonary Veno-Occlusive Disease
Source: Front Cardiovasc Med. 2022 Feb 16;9:746888. doi: 10.3389/fcvm.2022.746888 (PMC8889576; doi:10.3389/fcvm.2022.746888)
Supplement: Supplementary file 1 [file Table_1.docx]

**Supplement table 1.** The raw data of reads count.

| **Samples** | **Reads**  **count** | **Bases**  **count** | **Average**  **length** | **Q10** | **Q20** | **Q30** | **GC**  **percentage** |
| --- | --- | --- | --- | --- | --- | --- | --- |
| **C1** | 9435980 | 707698500 | 75 | 100.00% | 96.45% | 94.87% | 48.54% |
| **C2** | 10052754 | 753956550 | 75 | 100.00% | 96.35% | 94.71% | 49.80% |
| **C3** | 12553433 | 941507475 | 75 | 100.00% | 95.78% | 93.73% | 51.09% |
| **M1** | 11540621 | 865546575 | 75 | 100.00% | 95.88% | 93.87% | 50.62% |
| **M2** | 11284053 | 846303975 | 75 | 100.00% | 96.04% | 94.13% | 50.79% |
| **M3** | 11420588 | 856544100 | 75 | 100.00% | 96.10% | 94.05% | 52.16% |

**Notes:** C1-C3 were control group; M1- M3 were PVOD group. PVOD, pulmonary veno-occlusive disease.
