# Supplementary material for: Differential Expression Profile of microRNAs and Tight Junction in the Lung Tissues of Rat With Mitomycin-C-Induced Pulmonary Veno-Occlusive Disease
Source: Front Cardiovasc Med. 2022 Feb 16;9:746888. doi: 10.3389/fcvm.2022.746888 (PMC8889576; doi:10.3389/fcvm.2022.746888)
Supplement: Supplementary file 2 [file Table_2.docx]

**Supplement table 2.** The clean data of reads count.

| **Samples** | **Reads**  **count** | **Uniq-reads**  **count** | **Bases**  **count** | **Average**  **length** | **Q10** | **Q20** | **Q30** | **GC**  **percentage** |
| --- | --- | --- | --- | --- | --- | --- | --- | --- |
| **C1** | 8177468 | 404409 | 184097036 | 22.51 | 100.00% | 98.11% | 97.23% | 47.60% |
| **C2** | 8810750 | 420052 | 197949009 | 22.47 | 100.00% | 98.12% | 97.24% | 47.45% |
| **C3** | 10749525 | 537190 | 241261069 | 22.44 | 100.00% | 98.07% | 97.15% | 47.22% |
| **M1** | 10293426 | 551525 | 239993476 | 23.32 | 100.00% | 98.12% | 97.24% | 46.21% |
| **M2** | 9946544 | 506074 | 231247189 | 23.25 | 100.00% | 98.10% | 97.21% | 46.70% |
| **M3** | 10199033 | 540885 | 238103811 | 23.35 | 100.00% | 98.08% | 97.18% | 46.86% |

**Notes:** C1-C3 were control group; M1- M3 were PVOD group. PVOD, pulmonary veno-occlusive disease.
