# Supplementary material for: Differential Expression Profile of microRNAs and Tight Junction in the Lung Tissues of Rat With Mitomycin-C-Induced Pulmonary Veno-Occlusive Disease
Source: Front Cardiovasc Med. 2022 Feb 16;9:746888. doi: 10.3389/fcvm.2022.746888 (PMC8889576; doi:10.3389/fcvm.2022.746888)
Supplement: Supplementary file 3 [file Table_3.docx]

**Supplement table 3.** The detailed steps of high-throughput sequencing in this study.

| **Steps** | **Detailed description** |
| --- | --- |
| 1. **Sequencing data processing and quality control** | The raw data were assessed for quality by FastQC, cutadapt software (version 1.14) was used to remove connectors, Trimmomatic software (version 0.36) was used to remove low quality bases at both ends and reads were filtered out. |
| 1. **The comparison of clean reads** | 2.1. The reads were compared with sRNA, tRNA, snRNA and snoRNA in RFAM database by blast software (version 2.6.0). The number and percentage of reads were counted and reads on the comparison were filtered out. |
|  | 2.2. The bowtie software (version 1.1.1) was used to compare reads with exon and intron sequences of species (rat), count the number and percentage of reads, and filter out reads that were compared with exon but not with intron. |
|  | 2.3. The bowtie software (version 1.1.1) was used to compare reads to the species reference genome sequence, count the number and percentage of reads that were compared, and filter out those that were not. |
| 1. **MicroRNAs analysis** | 3.1. The miRNA was analyzed which based on the miRNA family information in miRBase. |
|  | 3.2. miRNA and reference genome comparison, identification of new miRNAs, miRNA expression analysis, and miRNA secondary structure analysis by using miRDeep2 (version 2.0.0.8) (only for species with known miRNAs). |
|  | 3.3. The comparison results of miRDeep2 were used for miRNA base substitution analysis. |
|  | 3.4. The target prediction of miRNA and species reference transcripts was performed. |
|  | 3.5. The reads were compared with related miRBase species using blast software (version 2.6.0) which as the miRNA of species, and quantified it. |
|  | 3.6. The obtained miRNAs were analyzed for species origin. |
| 1. **The expression of miRNA analysis** | 4.1. The reads per million distribution of miRNA was analyzed. |
|  | 4.2. The sample correlation, sample distance, and PCA principal component analysis were evaluated. |
| 1. **Differential expression analysis of miRNA** | 5.1. According to the experimental groups, edgeR was used for differential expression analysis. P value <0 .05, the fold-change ≥ 2 was used as the screening condition to obtain miRNA with significantly different expression. MA, Scatter and volcano were performed, and significantly different expression miRNAs were counted. Corresponding difference analysis with biological repetition was conducted for miRNA cluster analysis. |
|  | 5.2. The venn analysis was performed to obtain common intersection genes and union genes. Then, the cluster analysis was performed, respectively. |
| 1. **Gene enrichment analysis** | 6.1. The differentially expressed miRNAs and the targeted mRNAs were obtained as the target genes. The enrichment analysis was conducted using Gene Ontology and KEGG pathway annotation. |
|  | 6.2. The classification statistics and P value distribution analysis were conducted for the enrichment items, respectively. The top items were selected to make bar graph, point graph, and gene network graph. |
