# Supplementary material for: Differential Expression Profile of microRNAs and Tight Junction in the Lung Tissues of Rat With Mitomycin-C-Induced Pulmonary Veno-Occlusive Disease
Source: Front Cardiovasc Med. 2022 Feb 16;9:746888. doi: 10.3389/fcvm.2022.746888 (PMC8889576; doi:10.3389/fcvm.2022.746888)
Supplement: Supplementary file 4 [file Table_4.docx]

**Supplement table 4.** The detail target genes of down-regulated miRNAs with differential expression.

| **miRNA** | **Regulated** | **Transcript** | **Gene** | **Name** | **Description** |
| --- | --- | --- | --- | --- | --- |
| rno-miR-150-5p | down | ENSRNOT00000064981 | ENSRNOG00000028238 | Sh3bgr | SH3 domain binding glutamate-rich protein |
| rno-miR-503-5p | down | ENSRNOT00000018640 | ENSRNOG00000013583 | Tbc1d8 | TBC1 domain family, member 8 |
| rno-novel-34-mature | down | ENSRNOT00000011065 | ENSRNOG00000008372 | Vamp7 | vesicle-associated membrane protein 7 |
| rno-novel-1-star | down | ENSRNOT00000001019 | ENSRNOG00000032930 | Trim26 | tripartite motif-containing 26 |
| rno-miR-351-3p | down | ENSRNOT00000045994 | ENSRNOG00000031210 | Vom1r48 | vomeronasal 1 receptor 48 |
| rno-miR-351-3p | down | ENSRNOT00000072122 | ENSRNOG00000031210 | Vom1r48 | vomeronasal 1 receptor 48 |
| rno-miR-503-5p | down | ENSRNOT00000080651 | ENSRNOG00000003261 | Usp9x | ubiquitin specific peptidase 9, X-linked |
| rno-miR-150-3p | down | ENSRNOT00000017019 | ENSRNOG00000012742 | Irx2 | iroquois homeobox 2 |
| rno-miR-150-3p | down | ENSRNOT00000015272 | ENSRNOG00000011180 | Irx5 | iroquois homeobox 5 |
| rno-novel-34-mature | down | ENSRNOT00000046054 | ENSRNOG00000010890 | Bmp1 | bone morphogenetic protein 1 |
| rno-miR-503-5p | down | ENSRNOT00000086242 | ENSRNOG00000015285 | Lrp4 | LDL receptor related protein 4 |
| rno-novel-34-mature | down | ENSRNOT00000079177 | ENSRNOG00000022698 | Vsig10 | V-set and immunoglobulin domain containing 10 |
| rno-miR-503-5p | down | ENSRNOT00000023385 | ENSRNOG00000017286 | Ephx2 | epoxide hydrolase 2 |
| rno-novel-53-mature | down | ENSRNOT00000055103 | ENSRNOG00000036760 | Tmc5 | transmembrane channel-like 5 |
| rno-miR-150-5p | down | ENSRNOT00000047347 | ENSRNOG00000046133 | LOC102553613 | SH3 domain-binding glutamic acid-rich protein-like |
| rno-novel-34-mature | down | ENSRNOT00000074379 | ENSRNOG00000056748 | AC229945.1 |  |
| rno-miR-503-5p | down | ENSRNOT00000021353 | ENSRNOG00000015285 | Lrp4 | LDL receptor related protein 4 |
| rno-miR-503-5p | down | ENSRNOT00000009152 | ENSRNOG00000006762 | LOC100359574 | NHP2 non-histone chromosome protein 2-like 1-like |
| rno-novel-106-mature | down | ENSRNOT00000027611 | ENSRNOG00000020349 | Rab3il1 | RAB3A interacting protein-like 1 |
| rno-miR-503-5p | down | ENSRNOT00000009041 | ENSRNOG00000011202 | Chrna4 | cholinergic receptor nicotinic alpha 4 subunit |
| rno-novel-106-mature | down | ENSRNOT00000000443 | ENSRNOG00000000394 | Srgn | serglycin |
| rno-miR-503-5p | down | ENSRNOT00000012578 | ENSRNOG00000009311 | Fstl3 | follistatin like 3 |
| rno-miR-503-5p | down | ENSRNOT00000070981 | ENSRNOG00000005336 | Prss27 | protease, serine 27 |
| rno-novel-34-mature | down | ENSRNOT00000019453 | ENSRNOG00000014424 | RGD1563354 | similar to hypothetical protein D630003M21 |
| rno-miR-503-5p | down | ENSRNOT00000048980 | ENSRNOG00000003261 | Usp9x | ubiquitin specific peptidase 9, X-linked |
| rno-novel-34-mature | down | ENSRNOT00000000304 | ENSRNOG00000000279 | Rtn4ip1 | reticulon 4 interacting protein 1 |
| rno-novel-106-mature | down | ENSRNOT00000080865 | ENSRNOG00000057782 | AC128476.2 |  |
| rno-novel-106-mature | down | ENSRNOT00000073991 | ENSRNOG00000047247 | Ptprs | protein tyrosine phosphatase, receptor type, S |
| rno-novel-106-mature | down | ENSRNOT00000074469 | ENSRNOG00000047247 | Ptprs | protein tyrosine phosphatase, receptor type, S |
| rno-novel-106-mature | down | ENSRNOT00000079999 | ENSRNOG00000047247 | Ptprs | protein tyrosine phosphatase, receptor type, S |
| rno-novel-34-mature | down | ENSRNOT00000016333 | ENSRNOG00000012163 | Plekhm2 | pleckstrin homology and RUN domain containing M2 |
| rno-novel-34-mature | down | ENSRNOT00000074210 | ENSRNOG00000047294 | AABR07043115.1 |  |
| rno-miR-503-5p | down | ENSRNOT00000075601 | ENSRNOG00000046971 | Ccer2 | coiled-coil glutamate-rich protein 2 |
| rno-novel-106-mature | down | ENSRNOT00000086722 | ENSRNOG00000051485 | AABR07017768.4 |  |
| rno-miR-503-5p | down | ENSRNOT00000013747 | ENSRNOG00000010252 | Hexa | hexosaminidase subunit alpha |
| rno-novel-106-mature | down | ENSRNOT00000065469 | ENSRNOG00000011440 | Ccdc39 | coiled-coil domain containing 39 |
| rno-novel-106-mature | down | ENSRNOT00000079678 | ENSRNOG00000011573 | Csad | cysteine sulfinic acid decarboxylase |
| rno-miR-150-5p | down | ENSRNOT00000071063 | ENSRNOG00000049480 | LOC686660 | similar to olfactory receptor 692 |
| rno-novel-34-mature | down | ENSRNOT00000007775 | ENSRNOG00000005911 | Rln3 | relaxin 3 |
| rno-miR-503-5p | down | ENSRNOT00000089574 | ENSRNOG00000012531 | Ephb2 | Eph receptor B2 |
| rno-miR-342-5p | down | ENSRNOT00000010377 | ENSRNOG00000007707 | Zfp467 | zinc finger protein 467 |
| rno-miR-503-5p | down | ENSRNOT00000016596 | ENSRNOG00000012354 | Trim23 | tripartite motif-containing 23 |
| rno-miR-503-5p | down | ENSRNOT00000016826 | ENSRNOG00000012521 | Shisa2 | shisa family member 2 |
| rno-novel-106-mature | down | ENSRNOT00000016205 | ENSRNOG00000011573 | Csad | cysteine sulfinic acid decarboxylase |
| rno-novel-106-mature | down | ENSRNOT00000066380 | ENSRNOG00000015822 | Klf13 | Kruppel-like factor 13 |
| rno-novel-106-mature | down | ENSRNOT00000018425 | ENSRNOG00000013314 | Avl9 | AVL9 cell migration associated |
| rno-miR-503-5p | down | ENSRNOT00000016649 | ENSRNOG00000012354 | Trim23 | tripartite motif-containing 23 |
| rno-novel-106-mature | down | ENSRNOT00000042200 | ENSRNOG00000005302 | Slc2a9 | solute carrier family 2 member 9 |
| rno-novel-79-star | down | ENSRNOT00000020419 | ENSRNOG00000015095 | Spryd7 | SPRY domain containing 7 |
| rno-miR-449c-5p | down | ENSRNOT00000019860 | ENSRNOG00000014644 | Zic1 | Zic family member 1 |
| rno-novel-106-mature | down | ENSRNOT00000046102 | ENSRNOG00000034242 | Vcp | valosin-containing protein |
| rno-novel-34-mature | down | ENSRNOT00000066561 | ENSRNOG00000021392 | Noc2l | NOC2-like nucleolar associated transcriptional repressor |
| rno-novel-6-mature | down | ENSRNOT00000034960 | ENSRNOG00000024119 | Ghsr | growth hormone secretagogue receptor |
| rno-novel-34-mature | down | ENSRNOT00000036235 | ENSRNOG00000026328 | Gpr137c | G protein-coupled receptor 137C |
| rno-novel-106-mature | down | ENSRNOT00000014211 | ENSRNOG00000010409 | Nol6 | nucleolar protein 6 |
| rno-novel-34-mature | down | ENSRNOT00000038915 | ENSRNOG00000023129 | Mfsd9 | major facilitator superfamily domain containing 9 |
| rno-miR-330-3p | down | ENSRNOT00000074667 | ENSRNOG00000045569 | Nol8 | nucleolar protein 8 |
| rno-novel-6-mature | down | ENSRNOT00000004283 | ENSRNOG00000003117 | Psmd12 | proteasome 26S subunit, non-ATPase 12 |
| rno-novel-34-mature | down | ENSRNOT00000032240 | ENSRNOG00000023210 | Trappc10 | trafficking protein particle complex 10 |
| rno-miR-652-3p | down | ENSRNOT00000026076 | ENSRNOG00000019020 | Bbs2 | Bardet-Biedl syndrome 2 |
| rno-novel-106-mature | down | ENSRNOT00000023116 | ENSRNOG00000016625 | Slc22a2 | solute carrier family 22 member 2 |
| rno-miR-330-3p | down | ENSRNOT00000072507 | ENSRNOG00000049866 | Plcxd1 | phosphatidylinositol-specific phospholipase C, X domain containing 1 |
| rno-novel-106-mature | down | ENSRNOT00000057122 | ENSRNOG00000019129 | Fcgbp | Fc fragment of IgG binding protein |
| rno-miR-449c-5p | down | ENSRNOT00000023448 | ENSRNOG00000017420 | Nudt6 | nudix hydrolase 6 |
| rno-miR-449c-5p | down | ENSRNOT00000084825 | ENSRNOG00000017420 | Nudt6 | nudix hydrolase 6 |
| rno-novel-6-mature | down | ENSRNOT00000023448 | ENSRNOG00000017420 | Nudt6 | nudix hydrolase 6 |
| rno-novel-6-mature | down | ENSRNOT00000084825 | ENSRNOG00000017420 | Nudt6 | nudix hydrolase 6 |
| rno-novel-34-mature | down | ENSRNOT00000002638 | ENSRNOG00000024955 | Scyl2 | SCY1 like pseudokinase 2 |
| rno-miR-150-5p | down | ENSRNOT00000066953 | ENSRNOG00000042367 | Cyp2ab1 | cytochrome P450, family 2, subfamily ab, polypeptide 1 |
| rno-novel-34-mature | down | ENSRNOT00000066106 | ENSRNOG00000008340 | RGD1309779 | similar to ENSANGP00000021391 [Source:RGD Symbol;Acc:1309779 |
| rno-miR-547-5p | down | ENSRNOT00000073679 | ENSRNOG00000045955 | Mrgbp | MRG domain binding protein |
| rno-novel-34-mature | down | ENSRNOT00000083485 | ENSRNOG00000060742 | AABR07029809.1 |  |
| rno-novel-106-mature | down | ENSRNOT00000002533 | ENSRNOG00000001849 | Mapk1 | mitogen activated protein kinase 1 |
| rno-novel-106-mature | down | ENSRNOT00000008630 | ENSRNOG00000006336 | Fyco1 | FYVE and coiled-coil domain containing 1 |
| rno-miR-503-5p | down | ENSRNOT00000007392 | ENSRNOG00000005336 | Prss27 | protease, serine 27 |
| rno-miR-503-5p | down | ENSRNOT00000087322 | ENSRNOG00000010492 | Sp2 | Sp2 transcription factor |
| rno-miR-503-5p | down | ENSRNOT00000002465 | ENSRNOG00000001807 | Sspn | sarcospan |
| rno-novel-79-star | down | ENSRNOT00000076079 | ENSRNOG00000002767 | Dlg3 | discs large MAGUK scaffold protein 3 |
| rno-novel-79-star | down | ENSRNOT00000003741 | ENSRNOG00000002767 | Dlg3 | discs large MAGUK scaffold protein 3 |
| rno-novel-79-star | down | ENSRNOT00000045082 | ENSRNOG00000002767 | Dlg3 | discs large MAGUK scaffold protein 3 |
| rno-novel-34-mature | down | ENSRNOT00000017369 | ENSRNOG00000012619 | Epor | erythropoietin receptor |
| rno-miR-449c-5p | down | ENSRNOT00000023437 | ENSRNOG00000017420 | Nudt6 | nudix hydrolase 6 |
| rno-novel-6-mature | down | ENSRNOT00000023437 | ENSRNOG00000017420 | Nudt6 | nudix hydrolase 6 |
| rno-novel-34-mature | down | ENSRNOT00000030097 | ENSRNOG00000042404 | Ccdc122 | coiled-coil domain containing 122 |
| rno-novel-106-mature | down | ENSRNOT00000079190 | ENSRNOG00000061004 | Asphd2 | aspartate beta-hydroxylase domain containing 2 |
| rno-novel-34-mature | down | ENSRNOT00000038293 | ENSRNOG00000022698 | Vsig10 | V-set and immunoglobulin domain containing 10 |
| rno-novel-34-mature | down | ENSRNOT00000082016 | ENSRNOG00000024955 | Scyl2 | SCY1 like pseudokinase 2 |
| rno-miR-351-5p | down | ENSRNOT00000027129 | ENSRNOG00000020010 | Kctd13 | potassium channel tetramerization domain containing 13 |
| rno-miR-503-5p | down | ENSRNOT00000085943 | ENSRNOG00000057803 | LOC100909439 | ankyrin repeat and SOCS box protein 2-like |
| rno-miR-150-5p | down | ENSRNOT00000023404 | ENSRNOG00000017326 | Ctbp2 | C-terminal binding protein 2 |
| rno-miR-547-5p | down | ENSRNOT00000086626 | ENSRNOG00000059891 | NEWGENE_1308612 | MRG/MORF4L binding protein |
| rno-novel-79-star | down | ENSRNOT00000077397 | ENSRNOG00000027139 | Krt20 | keratin 20 |
| rno-novel-34-mature | down | ENSRNOT00000001921 | ENSRNOG00000001417 | Plod3 | procollagen-lysine, 2-oxoglutarate 5-dioxygenase 3 |
| rno-miR-330-3p | down | ENSRNOT00000083991 | ENSRNOG00000013178 | Cmip | c-Maf-inducing protein |
| rno-miR-503-5p | down | ENSRNOT00000031291 | ENSRNOG00000028623 | Agpat5 | 1-acylglycerol-3-phosphate O-acyltransferase 5 |
| rno-novel-106-mature | down | ENSRNOT00000003512 | ENSRNOG00000002592 | Rps6ka6 | ribosomal protein S6 kinase A6 |
| rno-miR-330-3p | down | ENSRNOT00000017941 | ENSRNOG00000013178 | Cmip | c-Maf-inducing protein |
| rno-miR-150-3p | down | ENSRNOT00000073025 | ENSRNOG00000045846 | Rfx2 | regulatory factor X2 |
| rno-miR-17-1-3p | down | ENSRNOT00000064948 | ENSRNOG00000014375 | Adgrb2 | adhesion G protein-coupled receptor B2 |
| rno-novel-34-mature | down | ENSRNOT00000019923 | ENSRNOG00000014522 | Mlycd | malonyl-CoA decarboxylase |
| rno-miR-150-3p | down | ENSRNOT00000074820 | ENSRNOG00000023233 | LOC102546648 | uncharacterized LOC102546648 |
| rno-novel-106-mature | down | ENSRNOT00000027688 | ENSRNOG00000020434 | Ascl2 | achaete-scute family bHLH transcription factor 2 |
| rno-miR-503-5p | down | ENSRNOT00000000162 | ENSRNOG00000000150 | Dnajc1 | DnaJ heat shock protein family (Hsp40) member C1 |
| rno-miR-503-5p | down | ENSRNOT00000020702 | ENSRNOG00000015340 | Dnajc25 | DnaJ heat shock protein family (Hsp40) member C25 |
| rno-miR-503-5p | down | ENSRNOT00000066340 | ENSRNOG00000043093 | Ap1m2 | adaptor-related protein complex 1, mu 2 subunit |
| rno-novel-106-mature | down | ENSRNOT00000065181 | ENSRNOG00000017912 | Atp2a3 | ATPase sarcoplasmic/endoplasmic reticulum Ca2+ transporting 3 |
| rno-novel-34-mature | down | ENSRNOT00000022898 | ENSRNOG00000016892 | Nr2f6 | nuclear receptor subfamily 2, group F, member 6 |
| rno-novel-3-mature | down | ENSRNOT00000019135 | ENSRNOG00000013865 | Zmym6 | zinc finger MYM-type containing 6 |
| rno-miR-503-5p | down | ENSRNOT00000064779 | ENSRNOG00000002349 | Gabra2 | gamma-aminobutyric acid type A receptor alpha2 subunit |
| rno-novel-34-mature | down | ENSRNOT00000012533 | ENSRNOG00000009006 | Ccdc47 | coiled-coil domain containing 47 |
| rno-miR-150-3p | down | ENSRNOT00000077073 | ENSRNOG00000030160 | Zfp819 | zinc finger protein 819 |
| rno-miR-150-3p | down | ENSRNOT00000031291 | ENSRNOG00000028623 | Agpat5 | 1-acylglycerol-3-phosphate O-acyltransferase 5 |
| rno-miR-351-5p | down | ENSRNOT00000093048 | ENSRNOG00000021010 | Arl2 | ADP-ribosylation factor like GTPase 2 |
| rno-miR-150-3p | down | ENSRNOT00000007851 | ENSRNOG00000005935 | A3galt2 | alpha 1,3-galactosyltransferase 2 |
| rno-miR-351-5p | down | ENSRNOT00000031678 | ENSRNOG00000023318 | Tigd3 | tigger transposable element derived 3 |
| rno-miR-503-5p | down | ENSRNOT00000065043 | ENSRNOG00000015021 | Naxd | NAD(P)HX dehydratase |
| rno-miR-150-5p | down | ENSRNOT00000038898 | ENSRNOG00000027811 | Lilrb4 | leukocyte immunoglobulin like receptor B4 |
| rno-novel-34-mature | down | ENSRNOT00000051494 | ENSRNOG00000031247 | Lekr1 | leucine, glutamate and lysine rich 1 |
| rno-novel-106-mature | down | ENSRNOT00000019096 | ENSRNOG00000014226 | Plch2 | phospholipase C, eta 2 |
| rno-novel-34-mature | down | ENSRNOT00000064949 | ENSRNOG00000042502 | Smim17 | small integral membrane protein 17 |
| rno-novel-106-mature | down | ENSRNOT00000087723 | ENSRNOG00000017912 | Atp2a3 | ATPase sarcoplasmic/endoplasmic reticulum Ca2+ transporting 3 |
| rno-miR-150-3p | down | ENSRNOT00000071936 | ENSRNOG00000048975 | Trim42 | tripartite motif-containing 42 |
| rno-novel-34-mature | down | ENSRNOT00000050102 | ENSRNOG00000005298 | Zfp513 | zinc finger protein 513 |
| rno-novel-106-mature | down | ENSRNOT00000015740 | ENSRNOG00000011628 | Krt27 | keratin 27 |
| rno-novel-34-mature | down | ENSRNOT00000027015 | ENSRNOG00000027290 | Pcdhga7 | protocadherin gamma subfamily A, 7 |
| rno-miR-503-5p | down | ENSRNOT00000027920 | ENSRNOG00000020573 | Efna1 | ephrin A1 |
| rno-miR-201-3p | down | ENSRNOT00000083055 | ENSRNOG00000014251 | Capn5 | calpain 5 |
| rno-novel-106-mature | down | ENSRNOT00000073556 | ENSRNOG00000046601 | Unc5cl | unc-5 family C-terminal like |
| rno-novel-34-mature | down | ENSRNOT00000007578 | ENSRNOG00000005561 | Brinp1 | BMP/retinoic acid inducible neural specific 1 |
| rno-miR-17-1-3p | down | ENSRNOT00000088568 | ENSRNOG00000014375 | Adgrb2 | adhesion G protein-coupled receptor B2 |
| rno-miR-351-5p | down | ENSRNOT00000089729 | ENSRNOG00000018337 | Caly | calcyon neuron-specific vesicular protein |
| rno-miR-330-3p | down | ENSRNOT00000052360 | ENSRNOG00000031208 | Mgat1 | mannosyl (alpha-1,3-)-glycoprotein beta-1,2-N-acetylglucosaminyltransferase |
| rno-miR-503-5p | down | ENSRNOT00000021711 | ENSRNOG00000015843 | Itfg1 | integrin alpha FG-GAP repeat containing 1 |
| rno-miR-503-5p | down | ENSRNOT00000050908 | ENSRNOG00000015791 | Zdhhc12 | zinc finger, DHHC-type containing 12 |
| rno-miR-351-3p | down | ENSRNOT00000073970 | ENSRNOG00000048145 | Sstr1 | somatostatin receptor 1 |
| rno-novel-34-mature | down | ENSRNOT00000012322 | ENSRNOG00000009299 | Adra2c | adrenoceptor alpha 2C |
| rno-novel-53-mature | down | ENSRNOT00000015757 | ENSRNOG00000011414 | Psmc3 | proteasome 26S subunit, ATPase 3 |
| rno-novel-106-mature | down | ENSRNOT00000082904 | ENSRNOG00000011154 | Adgrf5 | adhesion G protein-coupled receptor F5 |
| rno-miR-652-3p | down | ENSRNOT00000077481 | ENSRNOG00000056904 | Gtf3c2 | general transcription factor IIIC subunit 2 |
| rno-novel-106-mature | down | ENSRNOT00000079388 | ENSRNOG00000053196 | Slc48a1 | solute carrier family 48 member 1 |
| rno-novel-34-mature | down | ENSRNOT00000085199 | ENSRNOG00000056033 | AC111632.2 |  |
| rno-novel-53-mature | down | ENSRNOT00000089754 | ENSRNOG00000053351 | Rhox3 | reproductive homeobox on X chromosome 3 |
| rno-novel-106-mature | down | ENSRNOT00000076531 | ENSRNOG00000046601 | Unc5cl | unc-5 family C-terminal like |
| rno-novel-34-mature | down | ENSRNOT00000015057 | ENSRNOG00000011071 | Nt5e | 5' nucleotidase, ecto |
| rno-miR-503-3p | down | ENSRNOT00000013899 | ENSRNOG00000009963 | Ctps1 | CTP synthase 1 |
| rno-miR-342-5p | down | ENSRNOT00000080654 | ENSRNOG00000007707 | Zfp467 | zinc finger protein 467 |
| rno-miR-330-3p | down | ENSRNOT00000085832 | ENSRNOG00000031208 | Mgat1 | mannosyl (alpha-1,3-)-glycoprotein beta-1,2-N-acetylglucosaminyltransferase |
| rno-miR-342-5p | down | ENSRNOT00000075777 | ENSRNOG00000047307 | Cntfr | ciliary neurotrophic factor receptor |
| rno-miR-500-5p | down | ENSRNOT00000085275 | ENSRNOG00000005302 | Slc2a9 | solute carrier family 2 member 9 |
| rno-miR-351-5p | down | ENSRNOT00000039447 | ENSRNOG00000028473 | LOC691551 | similar to F28B3.5a |
| rno-miR-150-3p | down | ENSRNOT00000024645 | ENSRNOG00000033729 | Zfp719 | zinc finger protein 719 |
| rno-novel-79-star | down | ENSRNOT00000042977 | ENSRNOG00000032706 | Kcnk18 | potassium two pore domain channel subfamily K member 18 |
| rno-miR-150-3p | down | ENSRNOT00000084312 | ENSRNOG00000045846 | Rfx2 | regulatory factor X2 |
| rno-miR-449c-5p | down | ENSRNOT00000009826 | ENSRNOG00000007477 | Edn3 | endothelin 3 |
| rno-novel-106-mature | down | ENSRNOT00000067774 | ENSRNOG00000005138 | Pip4k2c | phosphatidylinositol-5-phosphate 4-kinase type 2 gamma |
| rno-novel-106-mature | down | ENSRNOT00000025372 | ENSRNOG00000018748 | Slc16a11 | solute carrier family 16, member 11 |
| rno-novel-106-mature | down | ENSRNOT00000012340 | ENSRNOG00000009066 | Thra | thyroid hormone receptor alpha |
| rno-novel-106-mature | down | ENSRNOT00000079327 | ENSRNOG00000005138 | Pip4k2c | phosphatidylinositol-5-phosphate 4-kinase type 2 gamma |
| rno-miR-503-5p | down | ENSRNOT00000093604 | ENSRNOG00000002753 | Adam11 | ADAM metallopeptidase domain 11 |
| rno-novel-106-mature | down | ENSRNOT00000009015 | ENSRNOG00000006126 | Nup88 | nucleoporin 88 |
| rno-novel-34-mature | down | ENSRNOT00000059380 | ENSRNOG00000030039 | LOC100910852 | uncharacterized LOC100910852 |
| rno-novel-34-mature | down | ENSRNOT00000030082 | ENSRNOG00000021463 | Ppara | peroxisome proliferator activated receptor alpha |
| rno-miR-351-5p | down | ENSRNOT00000026286 | ENSRNOG00000019413 | Atg16l2 | autophagy related 16-like 2 |
| rno-miR-450b-3p | down | ENSRNOT00000016215 | ENSRNOG00000012103 | Nmbr | neuromedin B receptor |
| rno-miR-547-5p | down | ENSRNOT00000013698 | ENSRNOG00000010291 | Slc46a1 | solute carrier family 46 member 1 |
| rno-novel-34-mature | down | ENSRNOT00000015270 | ENSRNOG00000011320 | Igfbpl1 | insulin-like growth factor binding protein-like 1 |
| rno-novel-106-mature | down | ENSRNOT00000078464 | ENSRNOG00000046601 | Unc5cl | unc-5 family C-terminal like |
| rno-miR-503-5p | down | ENSRNOT00000012826 | ENSRNOG00000009243 | Oaf | out at first homolog |
| rno-novel-106-mature | down | ENSRNOT00000090717 | ENSRNOG00000010133 | Bpgm | bisphosphoglycerate mutase |
| rno-novel-106-mature | down | ENSRNOT00000023946 | ENSRNOG00000017756 | Mmp21 | matrix metallopeptidase 21 |
| rno-novel-34-mature | down | ENSRNOT00000072592 | ENSRNOG00000048651 | Nrtn | neurturin |
| rno-novel-53-mature | down | ENSRNOT00000045275 | ENSRNOG00000029528 | Cbs | cystathionine beta synthase |
| rno-novel-34-mature | down | ENSRNOT00000061129 | ENSRNOG00000039832 | Gpr12 | G protein-coupled receptor 12 |
| rno-novel-34-mature | down | ENSRNOT00000037867 | ENSRNOG00000022313 | Klk9 | kallikrein related-peptidase 9 |
| rno-novel-79-star | down | ENSRNOT00000064022 | ENSRNOG00000032706 | Kcnk18 | potassium two pore domain channel subfamily K member 18 |
| rno-novel-106-mature | down | ENSRNOT00000025576 | ENSRNOG00000018822 | Slc5a5 | solute carrier family 5 member 5 [Source:RGD Symbol;Acc:69267] |
| rno-novel-34-mature | down | ENSRNOT00000046557 | ENSRNOG00000013991 | Creg2 | cellular repressor of E1A-stimulated genes 2 |
| rno-novel-106-mature | down | ENSRNOT00000057034 | ENSRNOG00000020235 | Hnrnpl | heterogeneous nuclear ribonucleoprotein L |
| rno-novel-6-mature | down | ENSRNOT00000076164 | ENSRNOG00000051170 | Ddx17 | DEAD-box helicase 17 |
| rno-miR-351-5p | down | ENSRNOT00000024710 | ENSRNOG00000018257 | Hpx | hemopexin |
| rno-novel-106-mature | down | ENSRNOT00000039480 | ENSRNOG00000023467 | Fam168b | family with sequence similarity 168, member B |
| rno-novel-106-mature | down | ENSRNOT00000000824 | ENSRNOG00000000661 | Hps4 | Hermansky-Pudlak syndrome 4 |
| rno-novel-106-mature | down | ENSRNOT00000086611 | ENSRNOG00000057315 | Kcnh3 | potassium voltage-gated channel subfamily H member 3 |
| rno-novel-106-mature | down | ENSRNOT00000064840 | ENSRNOG00000017912 | Atp2a3 | ATPase sarcoplasmic/endoplasmic reticulum Ca2+ transporting 3 |
| rno-miR-652-3p | down | ENSRNOT00000067761 | ENSRNOG00000042628 | RGD1561145 | similar to novel protein |
| rno-miR-652-3p | down | ENSRNOT00000027614 | ENSRNOG00000020376 | Stn1 | STN1, CST complex subunit |
| rno-miR-503-5p | down | ENSRNOT00000084339 | ENSRNOG00000011202 | Chrna4 | cholinergic receptor nicotinic alpha 4 subunit |
| rno-miR-150-3p | down | ENSRNOT00000021802 | ENSRNOG00000015991 | Npr2 | natriuretic peptide receptor 2 |
| rno-novel-34-mature | down | ENSRNOT00000079819 | ENSRNOG00000032703 | Rasgrp3 | RAS guanyl releasing protein 3 |
| rno-miR-652-3p | down | ENSRNOT00000020062 | ENSRNOG00000014896 | Adgrg3 | adhesion G protein-coupled receptor G3 |
| rno-novel-106-mature | down | ENSRNOT00000010938 | ENSRNOG00000008217 | Madcam1 | mucosal vascular addressin cell adhesion molecule 1 |
| rno-miR-150-5p | down | ENSRNOT00000091149 | ENSRNOG00000000812 | RGD1302996 | hypothetical protein MGC:15854 |
| rno-novel-106-mature | down | ENSRNOT00000022717 | ENSRNOG00000016879 | Ldlrad4 | low density lipoprotein receptor class A domain containing 4 |
| rno-miR-450b-3p | down | ENSRNOT00000076049 | ENSRNOG00000012103 | Nmbr | neuromedin B receptor |
| rno-novel-34-mature | down | ENSRNOT00000012469 | ENSRNOG00000009050 | Amn | amnion associated transmembrane protein |
| rno-miR-503-5p | down | ENSRNOT00000006566 | ENSRNOG00000046159 | Gns | glucosamine (N-acetyl)-6-sulfatase |
| rno-miR-150-3p | down | ENSRNOT00000020630 | ENSRNOG00000015237 | Gle1 | GLE1 RNA export mediator |
| rno-miR-351-5p | down | ENSRNOT00000028097 | ENSRNOG00000020700 | Rnaseh2c | ribonuclease H2, subunit C |
| rno-miR-150-3p | down | ENSRNOT00000055111 | ENSRNOG00000012906 | Bcas1 | breast carcinoma amplified sequence 1 |
| rno-novel-106-mature | down | ENSRNOT00000065201 | ENSRNOG00000043357 | Zfp407 | zinc finger protein 407 |
| rno-novel-106-mature | down | ENSRNOT00000074868 | ENSRNOG00000047817 | Enox1 | ecto-NOX disulfide-thiol exchanger 1 |
| rno-miR-652-3p | down | ENSRNOT00000028416 | ENSRNOG00000020936 | Nradd | neurotrophin receptor associated death domain |
| rno-novel-106-mature | down | ENSRNOT00000078140 | ENSRNOG00000000394 | Srgn | serglycin |
| rno-miR-503-5p | down | ENSRNOT00000068083 | ENSRNOG00000010492 | Sp2 | Sp2 transcription factor |
| rno-novel-34-mature | down | ENSRNOT00000051239 | ENSRNOG00000032703 | Rasgrp3 | RAS guanyl releasing protein 3 |
| rno-novel-34-mature | down | ENSRNOT00000060996 | ENSRNOG00000014089 | Map3k2 | mitogen activated protein kinase kinase kinase 2 |
| rno-miR-503-5p | down | ENSRNOT00000004845 | ENSRNOG00000003648 | Cldn6 | claudin 6 |
| rno-novel-53-mature | down | ENSRNOT00000042432 | ENSRNOG00000029528 | Cbs | cystathionine beta synthase |
| rno-miR-150-3p | down | ENSRNOT00000074851 | ENSRNOG00000046700 | Wbscr27 | Williams Beuren syndrome chromosome region 27 |
| rno-novel-34-mature | down | ENSRNOT00000018464 | ENSRNOG00000013179 | Tinagl1 | tubulointerstitial nephritis antigen-like 1 |
| rno-novel-34-mature | down | ENSRNOT00000080073 | ENSRNOG00000059691 | AABR07033318.1 |  |
| rno-novel-34-mature | down | ENSRNOT00000079990 | ENSRNOG00000054519 | AABR07053179.1 |  |
| rno-miR-503-5p | down | ENSRNOT00000087153 | ENSRNOG00000020532 | Kcnq1 | potassium voltage-gated channel subfamily Q member 1 |
| rno-miR-330-3p | down | ENSRNOT00000073986 | ENSRNOG00000049866 | Plcxd1 | phosphatidylinositol-specific phospholipase C, X domain containing 1 |
| rno-miR-330-3p | down | ENSRNOT00000054863 | ENSRNOG00000026039 | Tspan32 | tetraspanin 32 |
| rno-novel-106-mature | down | ENSRNOT00000022658 | ENSRNOG00000016706 | Fanca | Fanconi anemia, complementation group A |
| rno-novel-34-mature | down | ENSRNOT00000006356 | ENSRNOG00000004563 | Sec24a | SEC24 homolog A, COPII coat complex component |
| rno-miR-503-5p | down | ENSRNOT00000012156 | ENSRNOG00000009180 | Xkr7 | XK related 7 |
| rno-novel-106-mature | down | ENSRNOT00000015223 | ENSRNOG00000011154 | Adgrf5 | adhesion G protein-coupled receptor F5 |
| rno-novel-34-mature | down | ENSRNOT00000014546 | ENSRNOG00000010882 | Sptlc1 | serine palmitoyltransferase, long chain base subunit 1 |
| rno-miR-503-5p | down | ENSRNOT00000001277 | ENSRNOG00000000962 | Slc15a4 | solute carrier family 15 member 4 |
| rno-novel-79-star | down | ENSRNOT00000076307 | ENSRNOG00000037251 | Zfp248 | zinc finger protein 248 |
| rno-novel-106-mature | down | ENSRNOT00000087346 | ENSRNOG00000009066 | Thra | thyroid hormone receptor alpha |
| rno-miR-450b-3p | down | ENSRNOT00000009584 | ENSRNOG00000006972 | Zfp189 | zinc finger protein 189 |
| rno-miR-330-3p | down | ENSRNOT00000093381 | ENSRNOG00000045569 | Nol8 | nucleolar protein 8 |
| rno-miR-503-5p | down | ENSRNOT00000027875 | ENSRNOG00000020532 | Kcnq1 | potassium voltage-gated channel subfamily Q member 1 |
| rno-novel-106-mature | down | ENSRNOT00000010613 | ENSRNOG00000007951 | Galnt14 | polypeptide N-acetylgalactosaminyltransferase 14 |
| rno-miR-150-5p | down | ENSRNOT00000077186 | ENSRNOG00000027811 | Lilrb4 | leukocyte immunoglobulin like receptor B4 |
| rno-novel-34-mature | down | ENSRNOT00000005006 | ENSRNOG00000003769 | Tmem163 | transmembrane protein 163 |
| rno-novel-106-mature | down | ENSRNOT00000013569 | ENSRNOG00000010133 | Bpgm | bisphosphoglycerate mutase |
| rno-miR-150-3p | down | ENSRNOT00000066001 | ENSRNOG00000008922 | Trim14 | tripartite motif-containing 14 |
| rno-miR-449c-5p | down | ENSRNOT00000087474 | ENSRNOG00000018033 | Ddx19a | DEAD-box helicase 19A |
| rno-miR-150-3p | down | ENSRNOT00000022563 | ENSRNOG00000016804 | Il25 | interleukin 25 |
| rno-miR-17-1-3p | down | ENSRNOT00000091927 | ENSRNOG00000058202 | Ppp2r2c | protein phosphatase 2, regulatory subunit B, gamma |
| rno-miR-449c-5p | down | ENSRNOT00000084462 | ENSRNOG00000018033 | Ddx19a | DEAD-box helicase 19A |
| rno-miR-330-3p | down | ENSRNOT00000016919 | ENSRNOG00000012344 | Slc7a9 | solute carrier family 7 member 9 |
| rno-novel-34-mature | down | ENSRNOT00000010433 | ENSRNOG00000007763 | Plod1 | procollagen-lysine, 2-oxoglutarate 5-dioxygenase 1 |
| rno-novel-34-mature | down | ENSRNOT00000089981 | ENSRNOG00000014197 | Tmem51 | transmembrane protein 51 |
| rno-miR-150-5p | down | ENSRNOT00000039438 | ENSRNOG00000034184 | Zfp583 | zinc finger protein 583 |
| rno-miR-652-3p | down | ENSRNOT00000084911 | ENSRNOG00000051970 | Aqp5 | aquaporin 5 |
| rno-miR-322-5p | down | ENSRNOT00000067840 | ENSRNOG00000018666 | Gpsm1 | G-protein signaling modulator 1 |
| rno-miR-503-5p | down | ENSRNOT00000032355 | ENSRNOG00000023640 | Rab25 | RAB25, member RAS oncogene family |
| rno-novel-34-mature | down | ENSRNOT00000077275 | ENSRNOG00000059579 | Gpt2 | glutamic--pyruvic transaminase 2 |
| rno-novel-106-mature | down | ENSRNOT00000013193 | ENSRNOG00000009906 | Slfnl1 | schlafen-like 1 |
| rno-miR-351-3p | down | ENSRNOT00000036103 | ENSRNOG00000025670 | Shisa3 | shisa family member 3 |
| rno-miR-17-1-3p | down | ENSRNOT00000020499 | ENSRNOG00000015249 | Taf8 | TATA-box binding protein associated factor 8 |
| rno-miR-201-3p | down | ENSRNOT00000019370 | ENSRNOG00000014251 | Capn5 | calpain 5 [Source:RGD Symbol;Acc:620084] |
| rno-miR-503-5p | down | ENSRNOT00000064041 | ENSRNOG00000012420 | Bcl9l | B-cell CLL/lymphoma 9-like |
| rno-novel-106-mature | down | ENSRNOT00000011032 | ENSRNOG00000008239 | Repin1 | replication initiator 1 |
| rno-novel-53-mature | down | ENSRNOT00000027135 | ENSRNOG00000019985 | Asic4 | acid sensing ion channel subunit family member 4 |
| rno-miR-503-5p | down | ENSRNOT00000077363 | ENSRNOG00000055917 | Trim27 | tripartite motif-containing 27 |
| rno-novel-106-mature | down | ENSRNOT00000067562 | ENSRNOG00000019424 | Aspdh | aspartate dehydrogenase domain containing |
| rno-novel-106-mature | down | ENSRNOT00000083414 | ENSRNOG00000016879 | Ldlrad4 | low density lipoprotein receptor class A domain containing 4 |
| rno-novel-34-mature | down | ENSRNOT00000008940 | ENSRNOG00000006327 | Rcc2 | regulator of chromosome condensation 2 |
| rno-novel-34-mature | down | ENSRNOT00000029629 | ENSRNOG00000026408 | Rnf169 | ring finger protein 169 |
| rno-novel-106-mature | down | ENSRNOT00000013244 | ENSRNOG00000009715 | Me1 | malic enzyme 1 |
| rno-miR-150-3p | down | ENSRNOT00000078067 | ENSRNOG00000052134 | Pigy | phosphatidylinositol glycan anchor biosynthesis, class Y |
| rno-miR-351-3p | down | ENSRNOT00000034289 | ENSRNOG00000025648 | Dhrs7l1 | dehydrogenase/reductase (SDR family) member 7-like 1 |
| rno-miR-330-3p | down | ENSRNOT00000004117 | ENSRNOG00000003018 | Olfml2b | olfactomedin-like 2B |
| rno-miR-330-3p | down | ENSRNOT00000086633 | ENSRNOG00000059947 | Sdc1 | syndecan 1 |
| rno-novel-106-mature | down | ENSRNOT00000023663 | ENSRNOG00000027607 | Ceacam3 | carcinoembryonic antigen-related cell adhesion molecule 3 |
| rno-miR-351-5p | down | ENSRNOT00000014573 | ENSRNOG00000010803 | Gabra5 | gamma-aminobutyric acid type A receptor alpha 5 subunit |
| rno-miR-17-1-3p | down | ENSRNOT00000088469 | ENSRNOG00000058202 | Ppp2r2c | protein phosphatase 2, regulatory subunit B, gamma |
| rno-miR-509-5p | down | ENSRNOT00000075040 | ENSRNOG00000045616 | LOC100911398 | olfactory receptor 2AK2-like |
| rno-miR-509-5p | down | ENSRNOT00000071262 | ENSRNOG00000047820 | Olr1434 | olfactory receptor 1434 |
| rno-miR-150-3p | down | ENSRNOT00000026883 | ENSRNOG00000019780 | Sypl2 | synaptophysin-like 2 |
| rno-miR-449c-5p | down | ENSRNOT00000091788 | ENSRNOG00000018033 | Ddx19a | DEAD-box helicase 19A |
| rno-novel-34-mature | down | ENSRNOT00000007993 | ENSRNOG00000006093 | LRRTM1 | leucine rich repeat transmembrane neuronal 1 |
| rno-miR-150-5p | down | ENSRNOT00000030794 | ENSRNOG00000022387 | Olr1589 | olfactory receptor 1589 |
| rno-novel-34-mature | down | ENSRNOT00000075777 | ENSRNOG00000047307 | Cntfr | ciliary neurotrophic factor receptor |
| rno-miR-351-3p | down | ENSRNOT00000061185 | ENSRNOG00000039856 | Lrrc73 | leucine rich repeat containing 73 [ |
| rno-novel-34-mature | down | ENSRNOT00000045756 | ENSRNOG00000046297 | Klk1c10 | kallikrein 1-related peptidase C10 |
| rno-novel-34-mature | down | ENSRNOT00000041899 | ENSRNOG00000046670 | LOC100911689 | glandular kallikrein-10-like |
| rno-novel-106-mature | down | ENSRNOT00000020892 | ENSRNOG00000015508 | RGD1307235 | similar to RIKEN cDNA 2310035C23 |
| rno-miR-150-3p | down | ENSRNOT00000084085 | ENSRNOG00000009712 | Gale | UDP-galactose-4-epimerase |
| rno-miR-449c-5p | down | ENSRNOT00000082566 | ENSRNOG00000018033 | Ddx19a | DEAD-box helicase 19A |
| rno-novel-79-star | down | ENSRNOT00000076927 | ENSRNOG00000037251 | Zfp248 | zinc finger protein 248 |
| rno-miR-330-3p | down | ENSRNOT00000073035 | ENSRNOG00000046968 | Nol8 | nucleolar protein 8 |
| rno-miR-652-3p | down | ENSRNOT00000038312 | ENSRNOG00000021420 | Tirap | TIR domain containing adaptor protein |
| rno-miR-503-5p | down | ENSRNOT00000023592 | ENSRNOG00000017557 | LOC100362216 | hypothetical protein LOC100362216 |
| rno-miR-322-5p | down | ENSRNOT00000014749 | ENSRNOG00000011009 | Cmtm4 | CKLF-like MARVEL transmembrane domain containing 4 |
| rno-novel-53-mature | down | ENSRNOT00000017833 | ENSRNOG00000012620 | Syngr3 | synaptogyrin 3 |
| rno-novel-53-mature | down | ENSRNOT00000001882 | ENSRNOG00000001391 | Sdsl | serine dehydratase-like |
| rno-novel-106-mature | down | ENSRNOT00000006876 | ENSRNOG00000005111 | Nutm1 | NUT midline carcinoma, family member 1 |
| rno-novel-34-mature | down | ENSRNOT00000093277 | ENSRNOG00000062295 | Gm9918 | predicted gene 9918 |
| rno-novel-34-mature | down | ENSRNOT00000030399 | ENSRNOG00000026994 | Afg3l1 | AFG3(ATPase family gene 3)-like 1 (S. cerevisiae) |
| rno-miR-342-5p | down | ENSRNOT00000088917 | ENSRNOG00000007646 | Sipa1l1 | signal-induced proliferation-associated 1 like 1 |
| rno-miR-652-3p | down | ENSRNOT00000050980 | ENSRNOG00000016050 | Fgfr1 | Fibroblast growth factor receptor 1 |
| rno-novel-106-mature | down | ENSRNOT00000057986 | ENSRNOG00000038101 | LOC102557319 | carcinoembryonic antigen-related cell adhesion molecule 3-like |
| rno-miR-150-3p | down | ENSRNOT00000086962 | ENSRNOG00000008922 | Trim14 | tripartite motif-containing 14 |
| rno-novel-106-mature | down | ENSRNOT00000002712 | ENSRNOG00000001980 | Ugt2b35 | UDP glucuronosyltransferase 2 family, polypeptide B35 |
| rno-miR-503-5p | down | ENSRNOT00000078299 | ENSRNOG00000052205 | Fgf23 | fibroblast growth factor 23 |
| rno-miR-201-3p | down | ENSRNOT00000036025 | ENSRNOG00000021285 | Celsr1 | cadherin, EGF LAG seven-pass G-type receptor 1 |
| rno-miR-150-3p | down | ENSRNOT00000000337 | ENSRNOG00000000307 | Mical1 | microtubule associated monooxygenase, calponin and LIM domain containing 1 |
| rno-novel-106-mature | down | ENSRNOT00000092201 | ENSRNOG00000001980 | Ugt2b35 | UDP glucuronosyltransferase 2 family, polypeptide B35 |
| rno-novel-34-mature | down | ENSRNOT00000025831 | ENSRNOG00000032857 | Klk1 | kallikrein 1 |
| rno-miR-351-5p | down | ENSRNOT00000040850 | ENSRNOG00000020484 | Trpm5 | transient receptor potential cation channel, subfamily M, member 5 |
| rno-miR-17-1-3p | down | ENSRNOT00000067646 | ENSRNOG00000043037 | Zfp770 | zinc finger protein 770 |
| rno-novel-106-mature | down | ENSRNOT00000078977 | ENSRNOG00000009715 | Me1 | malic enzyme 1 |
| rno-novel-106-mature | down | ENSRNOT00000057988 | ENSRNOG00000038101 | LOC102557319 | carcinoembryonic antigen-related cell adhesion molecule 3-like |
| rno-novel-106-mature | down | ENSRNOT00000088393 | ENSRNOG00000038101 | LOC102557319 | carcinoembryonic antigen-related cell adhesion molecule 3-like |
| rno-miR-150-5p | down | ENSRNOT00000004279 | ENSRNOG00000003171 | Mpz | myelin protein zero |
| rno-novel-106-mature | down | ENSRNOT00000027427 | ENSRNOG00000020251 | Art1 | ADP-ribosyltransferase 1 |
| rno-novel-34-mature | down | ENSRNOT00000011940 | ENSRNOG00000008966 | Slco5a1 | solute carrier organic anion transporter family, member 5A1 |
| rno-novel-106-mature | down | ENSRNOT00000084075 | ENSRNOG00000059765 | Asic1 | acid sensing ion channel subunit 1 |
| rno-miR-351-5p | down | ENSRNOT00000019368 | ENSRNOG00000014373 | Trim66 | tripartite motif-containing 66 |
| rno-miR-322-5p | down | ENSRNOT00000014269 | ENSRNOG00000009667 | Mlst8 | MTOR associated protein, LST8 homolog |
| rno-novel-53-mature | down | ENSRNOT00000019368 | ENSRNOG00000014373 | Trim66 | tripartite motif-containing 66 |
| rno-novel-53-mature | down | ENSRNOT00000036178 | ENSRNOG00000027392 | Ccdc187 | coiled-coil domain containing 187 |
| rno-miR-201-3p | down | ENSRNOT00000001389 | ENSRNOG00000001052 | Slc25a30 | solute carrier family 25, member 30 |
| rno-miR-150-3p | down | ENSRNOT00000002231 | ENSRNOG00000001632 | Brwd1 | bromodomain and WD repeat domain containing 1 |
| rno-novel-53-mature | down | ENSRNOT00000091167 | ENSRNOG00000031792 | Mcpt1l4 | mast cell protease 1-like 4 |
| rno-miR-17-1-3p | down | ENSRNOT00000081834 | ENSRNOG00000054203 | Sim2 | single-minded family bHLH transcription factor 2 |
| rno-miR-330-3p | down | ENSRNOT00000064788 | ENSRNOG00000010189 | Rps24 | ribosomal protein S24 |
| rno-miR-342-5p | down | ENSRNOT00000040169 | ENSRNOG00000019557 | Asb18 | ankyrin repeat and SOCS box-containing 18 |
| rno-miR-503-5p | down | ENSRNOT00000004848 | ENSRNOG00000003654 | Cldn9 | claudin 9 [Source:RGD Symbol;Acc:1308999] |
| rno-miR-351-5p | down | ENSRNOT00000024765 | ENSRNOG00000018337 | Caly | calcyon neuron-specific vesicular protein |
| rno-miR-150-3p | down | ENSRNOT00000021103 | ENSRNOG00000015763 | Nat8f3 | N-acetyltransferase 8 (GCN5-related) family member 3 |
| rno-miR-708-3p | down | ENSRNOT00000022986 | ENSRNOG00000016955 | Fam188a | family with sequence similarity 188, member A |
| rno-miR-330-3p | down | ENSRNOT00000022661 | ENSRNOG00000016873 | Msrb2 | methionine sulfoxide reductase B2 |
| rno-miR-150-3p | down | ENSRNOT00000016495 | ENSRNOG00000011905 | Atp6v1e1 | ATPase H+ transporting V1 subunit E1 |
| rno-miR-150-3p | down | ENSRNOT00000088514 | ENSRNOG00000052361 | LOC100909966 | olfactory receptor 6F1-like |
| rno-miR-150-3p | down | ENSRNOT00000045582 | ENSRNOG00000032371 | Olr13 | olfactory receptor 13 |
| rno-miR-547-5p | down | ENSRNOT00000082863 | ENSRNOG00000009563 | Krt2 | keratin 2 |
| rno-miR-150-3p | down | ENSRNOT00000013460 | ENSRNOG00000010111 | Exoc3l4 | exocyst complex component 3-like 4 |
| rno-novel-53-mature | down | ENSRNOT00000081912 | ENSRNOG00000009440 | Gucy1b2 | guanylate cyclase 1 soluble subunit beta 2 |
| rno-novel-106-mature | down | ENSRNOT00000027093 | ENSRNOG00000019973 | Arl3 | ADP ribosylation factor like GTPase 3 |
| rno-miR-503-5p | down | ENSRNOT00000001373 | ENSRNOG00000001039 | Eif2b1 | eukaryotic translation initiation factor 2B subunit 1 alpha |
| rno-miR-503-5p | down | ENSRNOT00000092416 | ENSRNOG00000001039 | Eif2b1 | eukaryotic translation initiation factor 2B subunit 1 alpha |
| rno-novel-79-star | down | ENSRNOT00000082639 | ENSRNOG00000011427 | Hr | HR, lysine demethylase and nuclear receptor corepressor |
| rno-novel-53-mature | down | ENSRNOT00000079505 | ENSRNOG00000049991 | Mcpt8 | mast cell protease 8 |
| rno-novel-34-mature | down | ENSRNOT00000038972 | ENSRNOG00000024093 | Dmrta1 | DMRT-like family A1 |
| rno-miR-150-3p | down | ENSRNOT00000006366 | ENSRNOG00000004637 | Fbxo7 | F-box protein 7 |
| rno-novel-34-mature | down | ENSRNOT00000007404 | ENSRNOG00000005334 | Acvr2a | activin A receptor type 2A |
| rno-miR-503-5p | down | ENSRNOT00000092699 | ENSRNOG00000001039 | Eif2b1 | eukaryotic translation initiation factor 2B subunit 1 alpha |
| rno-miR-330-3p | down | ENSRNOT00000020838 | ENSRNOG00000015173 | Mbtps1 | membrane-bound transcription factor peptidase, site 1 |
| rno-novel-53-mature | down | ENSRNOT00000049315 | ENSRNOG00000049991 | Mcpt8 | mast cell protease 8 |
| rno-novel-34-mature | down | ENSRNOT00000045180 | ENSRNOG00000006747 | Cc2d1a | coiled-coil and C2 domain containing 1A |
| rno-miR-652-3p | down | ENSRNOT00000022182 | ENSRNOG00000016477 | Vangl1 | VANGL planar cell polarity protein 1 |
| rno-novel-106-mature | down | ENSRNOT00000024369 | ENSRNOG00000017897 | Adam8 | ADAM metallopeptidase domain 8 |
| rno-novel-79-star | down | ENSRNOT00000063787 | ENSRNOG00000019007 | Rpl14 | ribosomal protein L14 |
| rno-novel-106-mature | down | ENSRNOT00000073277 | ENSRNOG00000010415 | Atxn7l1 | ataxin 7-like 1 |
| rno-miR-500-5p | down | ENSRNOT00000060109 | ENSRNOG00000029964 | Tmprss11g | transmembrane protease, serine 11G |
| rno-miR-330-3p | down | ENSRNOT00000036447 | ENSRNOG00000027852 | RGD1311343 | similar to RIKEN cDNA 4930524B15 |
| rno-novel-53-mature | down | ENSRNOT00000009323 | ENSRNOG00000007017 | Tmc2 | transmembrane channel-like 2 |
| rno-novel-106-mature | down | ENSRNOT00000001918 | ENSRNOG00000001415 | Ap1s1 | adaptor-related protein complex 1, sigma 1 subunit |
| rno-novel-18-mature | down | ENSRNOT00000021868 | ENSRNOG00000016315 | Cnpy3 | canopy FGF signaling regulator 3 |
| rno-novel-106-mature | down | ENSRNOT00000087067 | ENSRNOG00000017311 | Me3 | malic enzyme 3 |
| rno-miR-503-5p | down | ENSRNOT00000009135 | ENSRNOG00000006978 | Mterf2 | mitochondrial transcription termination factor 2 |
| rno-miR-503-5p | down | ENSRNOT00000021592 | ENSRNOG00000016032 | Cnnm3 | cyclin and CBS domain divalent metal cation transport mediator 3 |
| rno-miR-500-5p | down | ENSRNOT00000055200 | ENSRNOG00000036802 | Snhg11 | small nucleolar RNA host gene 11 [Source:RGD Symbol;Acc:1563912] |
| rno-novel-79-star | down | ENSRNOT00000005930 | ENSRNOG00000004494 | Lta4h | leukotriene A4 hydrolase |
| rno-novel-106-mature | down | ENSRNOT00000034075 | ENSRNOG00000024536 | Ccbe1 | collagen and calcium binding EGF domains 1 |
| rno-novel-79-star | down | ENSRNOT00000083427 | ENSRNOG00000019007 | Rpl14 | ribosomal protein L14 |
| rno-miR-150-5p | down | ENSRNOT00000067530 | ENSRNOG00000034184 | Zfp583 | zinc finger protein 583 |
| rno-miR-503-5p | down | ENSRNOT00000018473 | ENSRNOG00000013721 | Nepro | nucleolus and neural progenitor protein |
| rno-novel-106-mature | down | ENSRNOT00000034069 | ENSRNOG00000024536 | Ccbe1 | collagen and calcium binding EGF domains 1 |
| rno-miR-503-5p | down | ENSRNOT00000077380 | ENSRNOG00000001039 | Eif2b1 | eukaryotic translation initiation factor 2B subunit 1 alpha |
| rno-novel-106-mature | down | ENSRNOT00000011925 | ENSRNOG00000008941 | Ets1 | ETS proto-oncogene 1, transcription factor |
| rno-novel-106-mature | down | ENSRNOT00000024380 | ENSRNOG00000018057 | Mrpl2 | mitochondrial ribosomal protein L2 |
| rno-novel-106-mature | down | ENSRNOT00000087632 | ENSRNOG00000032395 | Tfcp2 | transcription factor CP2 |
| rno-novel-34-mature | down | ENSRNOT00000019171 | ENSRNOG00000014197 | Tmem51 | transmembrane protein 51 |
| rno-miR-500-5p | down | ENSRNOT00000056935 | ENSRNOG00000037567 | RGD1309730 | similar to RIKEN cDNA B230118H07 |
| rno-novel-106-mature | down | ENSRNOT00000013474 | ENSRNOG00000010415 | Atxn7l1 | ataxin 7-like 1 |
| rno-novel-18-mature | down | ENSRNOT00000082949 | ENSRNOG00000051904 | Neu4 | neuraminidase 4 |
| rno-miR-330-3p | down | ENSRNOT00000011906 | ENSRNOG00000008915 | Prima1 | proline rich membrane anchor 1 |
| rno-novel-106-mature | down | ENSRNOT00000025260 | ENSRNOG00000018536 | Pck2 | phosphoenolpyruvate carboxykinase 2 (mitochondrial) |
| rno-novel-106-mature | down | ENSRNOT00000026263 | ENSRNOG00000019416 | Zfp94 | zinc finger protein 94 |
| rno-novel-106-mature | down | ENSRNOT00000077305 | ENSRNOG00000019416 | Zfp94 | zinc finger protein 94 |
| rno-novel-34-mature | down | ENSRNOT00000001856 | ENSRNOG00000001374 | Rasal1 | RAS protein activator like 1 (GAP1 like) |
| rno-novel-106-mature | down | ENSRNOT00000023329 | ENSRNOG00000017311 | Me3 | malic enzyme 3 |
| rno-novel-34-mature | down | ENSRNOT00000021304 | ENSRNOG00000015919 | Olr109 | olfactory receptor 109 |
| rno-novel-106-mature | down | ENSRNOT00000079256 | ENSRNOG00000000661 | Hps4 | Hermansky-Pudlak syndrome 4 |
| rno-novel-34-mature | down | ENSRNOT00000037998 | ENSRNOG00000025378 | Ccdc155 | coiled-coil domain containing 155 |
| rno-miR-503-5p | down | ENSRNOT00000028065 | ENSRNOG00000020675 | Ccdc97 | coiled-coil domain containing 97 |
| rno-novel-34-mature | down | ENSRNOT00000065217 | ENSRNOG00000042749 | RGD1562024 | RGD1562024 |
| rno-miR-150-5p | down | ENSRNOT00000022486 | ENSRNOG00000016790 | Kmt5b | lysine methyltransferase 5B |
| rno-miR-342-5p | down | ENSRNOT00000092099 | ENSRNOG00000058003 | Spon1 | spondin 1 |
| rno-novel-106-mature | down | ENSRNOT00000022003 | ENSRNOG00000016381 | Ust | uronyl-2-sulfotransferase |
| rno-miR-351-5p | down | ENSRNOT00000077776 | ENSRNOG00000049944 | LOC100911440 | mitochondrial glutamate carrier 1-like |
| rno-miR-509-5p | down | ENSRNOT00000042003 | ENSRNOG00000030102 | Mboat4 | membrane bound O-acyltransferase domain containing 4 |
| rno-miR-17-1-3p | down | ENSRNOT00000013225 | ENSRNOG00000009948 | Tmem55b | transmembrane protein 55B |
| rno-miR-449c-5p | down | ENSRNOT00000082042 | ENSRNOG00000016867 | Zfp346 | zinc finger protein 346 |
| rno-novel-53-mature | down | ENSRNOT00000085356 | ENSRNOG00000016075 | Dmrt1 | doublesex and mab-3 related transcription factor 1 |
| rno-miR-150-3p | down | ENSRNOT00000077577 | ENSRNOG00000004637 | Fbxo7 | F-box protein 7 |
| rno-novel-34-mature | down | ENSRNOT00000015498 | ENSRNOG00000011417 | Pde3b | phosphodiesterase 3B |
| rno-miR-351-3p | down | ENSRNOT00000000444 | ENSRNOG00000000395 | Kif1bp | KIF1 binding protein |
| rno-miR-503-5p | down | ENSRNOT00000068482 | ENSRNOG00000008984 | Irak1bp1 | interleukin-1 receptor-associated kinase 1 binding protein 1 |
| rno-miR-17-1-3p | down | ENSRNOT00000046384 | ENSRNOG00000031648 | Olr340 | olfactory receptor 340 |
| rno-novel-34-mature | down | ENSRNOT00000068497 | ENSRNOG00000020412 | Sf3b2 | splicing factor 3b, subunit 2 |
| rno-novel-34-mature | down | ENSRNOT00000002928 | ENSRNOG00000002149 | Nkx6-1 | NK6 homeobox 1 |
| rno-miR-17-1-3p | down | ENSRNOT00000066512 | ENSRNOG00000043313 | LOC687097 | similar to Olfactory receptor 5B2 (OST073) (Olfactory receptor OR11-240) |
| rno-miR-449c-5p | down | ENSRNOT00000022646 | ENSRNOG00000016867 | Zfp346 | zinc finger protein 346 |
| rno-miR-17-1-3p | down | ENSRNOT00000089570 | ENSRNOG00000054146 | Olr264 | olfactory receptor 264 |
| rno-miR-509-5p | down | ENSRNOT00000018882 | ENSRNOG00000037695 | Sgpp2 | sphingosine-1-phosphate phosphatase 2 |
| rno-miR-330-3p | down | ENSRNOT00000043358 | ENSRNOG00000048395 | Olr1515 | olfactory receptor 1515 |
| rno-miR-150-3p | down | ENSRNOT00000088687 | ENSRNOG00000004084 | Fam84a | family with sequence similarity 84, member A |
| rno-novel-106-mature | down | ENSRNOT00000086917 | ENSRNOG00000019424 | Aspdh | aspartate dehydrogenase domain containing |
| rno-miR-503-5p | down | ENSRNOT00000072342 | ENSRNOG00000049743 | NEWGENE_620381 | glutathione S-transferase mu 3 |
| rno-novel-34-mature | down | ENSRNOT00000009555 | ENSRNOG00000006916 | Sardh | sarcosine dehydrogenase |
| rno-novel-106-mature | down | ENSRNOT00000001610 | ENSRNOG00000001212 | Dnmt3l | DNA methyltransferase 3 like |
| rno-novel-106-mature | down | ENSRNOT00000051265 | ENSRNOG00000016917 | Clcn1 | chloride voltage-gated channel 1 |
| rno-novel-106-mature | down | ENSRNOT00000015944 | ENSRNOG00000011824 | Trh | thyrotropin releasing hormone |
| rno-novel-34-mature | down | ENSRNOT00000009806 | ENSRNOG00000006747 | Cc2d1a | coiled-coil and C2 domain containing 1A |
| rno-novel-79-star | down | ENSRNOT00000034889 | ENSRNOG00000027139 | Krt20 | keratin 20 |
| rno-miR-351-5p | down | ENSRNOT00000086238 | ENSRNOG00000018450 | Slc25a22 | solute carrier family 25 member 22 |
| rno-miR-351-5p | down | ENSRNOT00000027727 | ENSRNOG00000020455 | Cst6 | cystatin E/M |
| rno-novel-106-mature | down | ENSRNOT00000091901 | ENSRNOG00000008825 | Ssrp1 | structure specific recognition protein 1 |
| rno-miR-150-3p | down | ENSRNOT00000084611 | ENSRNOG00000004657 | Sec23a | Sec23 homolog A, coat complex II component |
| rno-novel-53-mature | down | ENSRNOT00000074160 | ENSRNOG00000045623 | Capn10 | calpain 10 |
| rno-novel-53-mature | down | ENSRNOT00000089602 | ENSRNOG00000054008 | Scamp5 | secretory carrier membrane protein 5 |
| rno-novel-106-mature | down | ENSRNOT00000082120 | ENSRNOG00000037340 | Epha10 | EPH receptor A10 |
| rno-novel-106-mature | down | ENSRNOT00000056496 | ENSRNOG00000037340 | Epha10 | EPH receptor A10 |
| rno-miR-342-5p | down | ENSRNOT00000010626 | ENSRNOG00000007646 | Sipa1l1 | signal-induced proliferation-associated 1 like 1 |
| rno-miR-342-5p | down | ENSRNOT00000092169 | ENSRNOG00000051531 | Ctu2 | cytosolic thiouridylase subunit 2 |
| rno-miR-351-5p | down | ENSRNOT00000014764 | ENSRNOG00000011074 | Calcb | calcitonin-related polypeptide, beta |
| rno-novel-34-mature | down | ENSRNOT00000083283 | ENSRNOG00000021086 | Dtx4 | deltex E3 ubiquitin ligase 4 |
| rno-novel-106-mature | down | ENSRNOT00000029284 | ENSRNOG00000016050 | Fgfr1 | Fibroblast growth factor receptor 1 |
| rno-novel-53-mature | down | ENSRNOT00000077672 | ENSRNOG00000055738 | LOC103692976 | cyclin-T1-like |
| rno-novel-79-star | down | ENSRNOT00000020822 | ENSRNOG00000015415 | Rhoq | ras homolog family member Q |
| rno-novel-34-mature | down | ENSRNOT00000037068 | ENSRNOG00000026953 | Gpr88 | G-protein coupled receptor 88 |
| rno-miR-503-5p | down | ENSRNOT00000022786 | ENSRNOG00000016989 | Dolk | dolichol kinase |
| rno-novel-53-mature | down | ENSRNOT00000021779 | ENSRNOG00000016075 | Dmrt1 | doublesex and mab-3 related transcription factor 1 |
| rno-novel-106-mature | down | ENSRNOT00000012022 | ENSRNOG00000008825 | Ssrp1 | structure specific recognition protein 1 |
| rno-miR-17-1-3p | down | ENSRNOT00000090368 | ENSRNOG00000033343 | Prss36 | protease, serine, 36 |
| rno-novel-34-mature | down | ENSRNOT00000083434 | ENSRNOG00000053706 | Lonrf1 | LON peptidase N-terminal domain and ring finger 1 |
| rno-novel-53-mature | down | ENSRNOT00000012948 | ENSRNOG00000009440 | Gucy1b2 | guanylate cyclase 1 soluble subunit beta 2 |
| rno-novel-18-mature | down | ENSRNOT00000065533 | ENSRNOG00000019031 | NEWGENE_1308624 | sialidase 4 |
| rno-miR-17-1-3p | down | ENSRNOT00000026587 | ENSRNOG00000033343 | Prss36 | protease, serine, 36 |
| rno-novel-34-mature | down | ENSRNOT00000085191 | ENSRNOG00000052296 | Shank3 | SH3 and multiple ankyrin repeat domains 3 |
| rno-miR-351-5p | down | ENSRNOT00000055016 | ENSRNOG00000027213 | Asphd1 | aspartate beta-hydroxylase domain containing 1 |
| rno-novel-106-mature | down | ENSRNOT00000079305 | ENSRNOG00000023856 | Agxt | alanine-glyoxylate aminotransferase |
| rno-miR-330-3p | down | ENSRNOT00000035125 | ENSRNOG00000022921 | Dact2 | dishevelled-binding antagonist of beta-catenin 2 |
| rno-novel-106-mature | down | ENSRNOT00000034672 | ENSRNOG00000024824 | Col22a1 | collagen type XXII alpha 1 chain |
| rno-miR-150-5p | down | ENSRNOT00000026318 | ENSRNOG00000019388 | Egfl7 | EGF-like-domain, multiple 7 |
| rno-novel-106-mature | down | ENSRNOT00000079909 | ENSRNOG00000057706 | Kdm5c | lysine demethylase 5C |
| rno-novel-106-mature | down | ENSRNOT00000087945 | ENSRNOG00000057706 | Kdm5c | lysine demethylase 5C |
| rno-novel-106-mature | down | ENSRNOT00000077528 | ENSRNOG00000057706 | Kdm5c | lysine demethylase 5C |
| rno-novel-106-mature | down | ENSRNOT00000037447 | ENSRNOG00000032395 | Tfcp2 | transcription factor CP2 |
| rno-novel-53-mature | down | ENSRNOT00000088999 | ENSRNOG00000053054 | Ccnt1 | cyclin T1 |
| rno-miR-150-3p | down | ENSRNOT00000006369 | ENSRNOG00000004657 | Sec23a | Sec23 homolog A, coat complex II component |
| rno-novel-34-mature | down | ENSRNOT00000037177 | ENSRNOG00000020225 | Dlgap4 | DLG associated protein 4 |
| rno-miR-503-5p | down | ENSRNOT00000032859 | ENSRNOG00000025034 | RGD1304567 | similar to RIKEN cDNA A430005L14 |
| rno-novel-106-mature | down | ENSRNOT00000001822 | ENSRNOG00000001348 | Erp29 | endoplasmic reticulum protein 29 |
| rno-miR-330-3p | down | ENSRNOT00000010283 | ENSRNOG00000007821 | Dyrk2 | dual specificity tyrosine phosphorylation regulated kinase 2 |
| rno-novel-53-mature | down | ENSRNOT00000009313 | ENSRNOG00000007002 | Lif | leukemia inhibitory factor |
| rno-novel-34-mature | down | ENSRNOT00000033132 | ENSRNOG00000028052 | RGD1563620 | similar to retinoblastoma binding protein 4 |
| rno-miR-351-3p | down | ENSRNOT00000027372 | ENSRNOG00000020119 | Pcdha4 | protocadherin alpha 4 |
| rno-novel-53-mature | down | ENSRNOT00000018303 | ENSRNOG00000013573 | Atp6v1b1 | ATPase H+ transporting V1 subunit B1 |
| rno-miR-150-3p | down | ENSRNOT00000027639 | ENSRNOG00000020390 | Tmem161a | transmembrane protein 161A |
| rno-miR-450b-3p | down | ENSRNOT00000006191 | ENSRNOG00000000504 | Fance | Fanconi anemia, complementation group E |
| rno-novel-53-mature | down | ENSRNOT00000003452 | ENSRNOG00000002548 | Tnn | tenascin N |
| rno-miR-351-5p | down | ENSRNOT00000038198 | ENSRNOG00000027564 | Tsga10ip | testis specific 10 interacting protein |
| rno-novel-106-mature | down | ENSRNOT00000051853 | ENSRNOG00000015902 | Cpxm2 | carboxypeptidase X (M14 family), member 2 |
| rno-miR-150-3p | down | ENSRNOT00000035252 | ENSRNOG00000023601 | Elavl4 | ELAV like RNA binding protein 4 |
| rno-novel-34-mature | down | ENSRNOT00000080166 | ENSRNOG00000013179 | Tinagl1 | tubulointerstitial nephritis antigen-like 1 |
| rno-miR-503-5p | down | ENSRNOT00000021589 | ENSRNOG00000016141 | Hoxc11 | homeobox C11 |
| rno-novel-79-star | down | ENSRNOT00000038767 | ENSRNOG00000017215 | RGD1308601 | similar to hypothetical protein |
| rno-miR-150-5p | down | ENSRNOT00000059869 | ENSRNOG00000006087 | Rnf20 | ring finger protein 20 |
| rno-novel-53-mature | down | ENSRNOT00000080012 | ENSRNOG00000054008 | Scamp5 | secretory carrier membrane protein 5 |
| rno-miR-17-1-3p | down | ENSRNOT00000013271 | ENSRNOG00000009184 | Foxp1 | forkhead box P1 |
| rno-miR-150-3p | down | ENSRNOT00000037068 | ENSRNOG00000026953 | Gpr88 | G-protein coupled receptor 88 |
| rno-novel-53-mature | down | ENSRNOT00000084970 | ENSRNOG00000017746 | Pard6a | par-6 family cell polarity regulator alpha |
| rno-miR-330-3p | down | ENSRNOT00000079196 | ENSRNOG00000048617 | Ensa | endosulfine alpha |
| rno-novel-34-mature | down | ENSRNOT00000016121 | ENSRNOG00000011815 | Sgk1 | serum/glucocorticoid regulated kinase 1 |
| rno-miR-342-5p | down | ENSRNOT00000020781 | ENSRNOG00000015458 | Hemk1 | HemK methyltransferase family member 1 |
| rno-novel-106-mature | down | ENSRNOT00000020152 | ENSRNOG00000014882 | Fgf11 | fibroblast growth factor 11 |
| rno-novel-34-mature | down | ENSRNOT00000046804 | ENSRNOG00000019086 | Gucy2f | guanylate cyclase 2F |
| rno-miR-351-3p | down | ENSRNOT00000027383 | ENSRNOG00000020119 | Pcdha4 | protocadherin alpha 4 |
| rno-novel-106-mature | down | ENSRNOT00000089789 | ENSRNOG00000008901 | Coro2a | coronin 2A |
| rno-novel-34-mature | down | ENSRNOT00000040736 | ENSRNOG00000011815 | Sgk1 | serum/glucocorticoid regulated kinase 1 |
| rno-miR-150-3p | down | ENSRNOT00000008302 | ENSRNOG00000005964 | Nr4a3 | nuclear receptor subfamily 4, group A, member 3 |
| rno-novel-34-mature | down | ENSRNOT00000029721 | ENSRNOG00000026091 | Slc10a4 | solute carrier family 10, member 4 |
| rno-miR-150-3p | down | ENSRNOT00000089179 | ENSRNOG00000013603 | Dffa | DNA fragmentation factor subunit alpha |
| rno-miR-500-5p | down | ENSRNOT00000037890 | ENSRNOG00000027408 | Ppid | peptidylprolyl isomerase D |
| rno-novel-34-mature | down | ENSRNOT00000049798 | ENSRNOG00000008474 | Acox3 | acyl-CoA oxidase 3, pristanoyl |
| rno-novel-34-mature | down | ENSRNOT00000085043 | ENSRNOG00000055103 | Cftr | cystic fibrosis transmembrane conductance regulator |
| rno-novel-6-mature | down | ENSRNOT00000031208 | ENSRNOG00000023768 | Rundc1 | RUN domain containing 1 |
| rno-novel-34-mature | down | ENSRNOT00000025627 | ENSRNOG00000018697 | Abcb6 | ATP-binding cassette, subfamily B (MDR/TAP), member 6 |
| rno-novel-53-mature | down | ENSRNOT00000044971 | ENSRNOG00000017746 | Pard6a | par-6 family cell polarity regulator alpha |
| rno-novel-106-mature | down | ENSRNOT00000027305 | ENSRNOG00000020075 | Eef1g | eukaryotic translation elongation factor 1 gamma |
| rno-novel-53-mature | down | ENSRNOT00000030164 | ENSRNOG00000028214 | Zscan4f | zinc finger and SCAN domain containing 4F |
| rno-miR-330-3p | down | ENSRNOT00000075035 | ENSRNOG00000046181 | Anks4b | ankyrin repeat and sterile alpha motif domain containing 4B |
| rno-miR-330-3p | down | ENSRNOT00000021865 | ENSRNOG00000016097 | Kyat1 | kynurenine aminotransferase 1 |
| rno-miR-351-5p | down | ENSRNOT00000051425 | ENSRNOG00000020332 | Tnnt3 | troponin T3, fast skeletal type |
| rno-miR-330-3p | down | ENSRNOT00000022563 | ENSRNOG00000016804 | Il25 | interleukin 25 |
| rno-miR-330-3p | down | ENSRNOT00000080065 | ENSRNOG00000022921 | Dact2 | dishevelled-binding antagonist of beta-catenin 2 |
| rno-miR-351-5p | down | ENSRNOT00000080339 | ENSRNOG00000020332 | Tnnt3 | troponin T3, fast skeletal type |
| rno-miR-449c-5p | down | ENSRNOT00000068444 | ENSRNOG00000011208 | Rapsn | receptor-associated protein of the synapse |
| rno-miR-351-5p | down | ENSRNOT00000066896 | ENSRNOG00000020332 | Tnnt3 | troponin T3, fast skeletal type |
| rno-miR-330-3p | down | ENSRNOT00000016112 | ENSRNOG00000011853 | Mbd2 | methyl-CpG binding domain protein 2 |
| rno-miR-322-5p | down | ENSRNOT00000004147 | ENSRNOG00000046276 | Myh3 | myosin heavy chain 3 |
| rno-miR-351-5p | down | ENSRNOT00000063918 | ENSRNOG00000020332 | Tnnt3 | troponin T3, fast skeletal type |
| rno-miR-330-3p | down | ENSRNOT00000090919 | ENSRNOG00000010086 | Plagl2 | PLAG1 like zinc finger 2 |
| rno-novel-34-mature | down | ENSRNOT00000061157 | ENSRNOG00000011815 | Sgk1 | serum/glucocorticoid regulated kinase 1 |
| rno-miR-150-3p | down | ENSRNOT00000055335 | ENSRNOG00000036864 | Actl10 | actin-like 10 |
| rno-novel-6-mature | down | ENSRNOT00000002554 | ENSRNOG00000001870 | Lztr1 | leucine-zipper-like transcription regulator 1 |
| rno-miR-351-5p | down | ENSRNOT00000040598 | ENSRNOG00000020332 | Tnnt3 | troponin T3, fast skeletal type |
| rno-miR-351-5p | down | ENSRNOT00000066135 | ENSRNOG00000020332 | Tnnt3 | troponin T3, fast skeletal type |
| rno-miR-330-3p | down | ENSRNOT00000011983 | ENSRNOG00000008890 | Slc18a2 | solute carrier family 18 member A2 |
| rno-novel-18-mature | down | ENSRNOT00000016362 | ENSRNOG00000012215 | Baiap2l2 | BAI1-associated protein 2-like 2 |
| rno-miR-342-5p | down | ENSRNOT00000008447 | ENSRNOG00000006281 | Hoxa3 | homeobox A3 |
| rno-novel-34-mature | down | ENSRNOT00000010084 | ENSRNOG00000007668 | Mafa | MAF bZIP transcription factor A |
| rno-miR-330-3p | down | ENSRNOT00000087886 | ENSRNOG00000015173 | Mbtps1 | membrane-bound transcription factor peptidase, site 1 |
| rno-novel-34-mature | down | ENSRNOT00000020669 | ENSRNOG00000015365 | Col4a3 | collagen type IV alpha 3 chain |
| rno-novel-34-mature | down | ENSRNOT00000067675 | ENSRNOG00000042163 | Btbd19 | BTB domain containing 19 |
| rno-miR-150-3p | down | ENSRNOT00000026392 | ENSRNOG00000062013 | Adprhl1 | ADP-ribosylhydrolase like 1 |
| rno-miR-503-5p | down | ENSRNOT00000015467 | ENSRNOG00000011237 | Zc3h13 | zinc finger CCCH type containing 13 |
| rno-novel-79-star | down | ENSRNOT00000040851 | ENSRNOG00000031061 | LOC306079 | similar to RIKEN cDNA 3100001N19 |
| rno-miR-17-1-3p | down | ENSRNOT00000037336 | ENSRNOG00000028749 | Atxn7l2 | ataxin 7-like 2 |
| rno-novel-106-mature | down | ENSRNOT00000027494 | ENSRNOG00000020235 | Hnrnpl | heterogeneous nuclear ribonucleoprotein L |
| rno-miR-150-3p | down | ENSRNOT00000023535 | ENSRNOG00000017359 | Itm2c | integral membrane protein 2C |
| rno-miR-449c-5p | down | ENSRNOT00000001479 | ENSRNOG00000001117 | Fbxl18 | F-box and leucine-rich repeat protein 18 |
| rno-miR-150-5p | down | ENSRNOT00000007535 | ENSRNOG00000005729 | Palm3 | paralemmin 3 |
| rno-novel-53-mature | down | ENSRNOT00000061821 | ENSRNOG00000001047 | Map2k7 | mitogen activated protein kinase kinase 7 |
| rno-miR-150-5p | down | ENSRNOT00000035427 | ENSRNOG00000028108 | Cytl1 | cytokine like 1 |
| rno-novel-79-star | down | ENSRNOT00000075047 | ENSRNOG00000046086 | Tmc8 | transmembrane channel-like 8 |
| rno-novel-34-mature | down | ENSRNOT00000092388 | ENSRNOG00000052296 | Shank3 | SH3 and multiple ankyrin repeat domains 3 |
| rno-miR-330-3p | down | ENSRNOT00000084045 | ENSRNOG00000051699 | Zdhhc4 | zinc finger, DHHC-type containing 4 |
| rno-miR-330-3p | down | ENSRNOT00000032992 | ENSRNOG00000025868 | Klhl33 | kelch-like family member 33 |
| rno-novel-34-mature | down | ENSRNOT00000084049 | ENSRNOG00000033202 | Fbxo41 | F-box protein 41 |
| rno-miR-322-5p | down | ENSRNOT00000022688 | ENSRNOG00000016834 | Wdr37 | WD repeat domain 37 |
| rno-novel-106-mature | down | ENSRNOT00000011787 | ENSRNOG00000008901 | Coro2a | coronin 2A |
| rno-miR-150-3p | down | ENSRNOT00000090256 | ENSRNOG00000061768 | Slc43a3 | solute carrier family 43, member 3 |
| rno-miR-150-3p | down | ENSRNOT00000021968 | ENSRNOG00000016167 | Spata2L | spermatogenesis associated 2-like |
| rno-miR-201-3p | down | ENSRNOT00000032620 | ENSRNOG00000007818 | Slc45a4 | solute carrier family 45, member 4 |
| rno-miR-150-3p | down | ENSRNOT00000055427 | ENSRNOG00000029245 | Gpr179 | G protein-coupled receptor 179 |
| rno-novel-53-mature | down | ENSRNOT00000084460 | ENSRNOG00000001047 | Map2k7 | mitogen activated protein kinase kinase 7 |
| rno-miR-150-3p | down | ENSRNOT00000065531 | ENSRNOG00000004084 | Fam84a | family with sequence similarity 84, member A |
| rno-miR-500-5p | down | ENSRNOT00000056255 | ENSRNOG00000037230 | Ppidl1 | peptidylprolyl isomerase D-like 1 |
| rno-novel-106-mature | down | ENSRNOT00000067414 | ENSRNOG00000003121 | Rtn4rl1 | reticulon 4 receptor-like 1 |
| rno-novel-106-mature | down | ENSRNOT00000071130 | ENSRNOG00000045626 | Pkd1l2 | polycystic kidney disease 1-like 2 |
| rno-novel-53-mature | down | ENSRNOT00000045950 | ENSRNOG00000038957 | RGD1305184 | similar to CDNA sequence BC023105 |
| rno-miR-92a-3p | down | ENSRNOT00000057190 | ENSRNOG00000037690 | Sertad3 | SERTA domain containing 3 |
| rno-miR-150-3p | down | ENSRNOT00000068381 | ENSRNOG00000012645 | Mecom | MDS1 and EVI1 complex locus |
| rno-miR-449c-5p | down | ENSRNOT00000032056 | ENSRNOG00000024112 | Dgkq | diacylglycerol kinase, theta |
| rno-miR-17-1-3p | down | ENSRNOT00000016020 | ENSRNOG00000011646 | Rem2 | RRAD and GEM like GTPase 2 |
| rno-novel-106-mature | down | ENSRNOT00000027507 | ENSRNOG00000020293 | Chrna10 | cholinergic receptor nicotinic alpha 10 subunit |
| rno-novel-18-mature | down | ENSRNOT00000081850 | ENSRNOG00000060410 | Pcdh1 | protocadherin 1 |
| rno-novel-106-mature | down | ENSRNOT00000027425 | ENSRNOG00000020235 | Hnrnpl | heterogeneous nuclear ribonucleoprotein L |
| rno-miR-17-1-3p | down | ENSRNOT00000049259 | ENSRNOG00000010188 | Satb2 | SATB homeobox 2 |
| rno-miR-652-3p | down | ENSRNOT00000029284 | ENSRNOG00000016050 | Fgfr1 | Fibroblast growth factor receptor 1 |
| rno-miR-351-5p | down | ENSRNOT00000089723 | ENSRNOG00000047756 | Mef2a | myocyte enhancer factor 2a |
| rno-novel-53-mature | down | ENSRNOT00000057386 | ENSRNOG00000016281 | Col4a1 | collagen type IV alpha 1 chain |
| rno-miR-351-5p | down | ENSRNOT00000084946 | ENSRNOG00000047756 | Mef2a | myocyte enhancer factor 2a |
| rno-miR-330-3p | down | ENSRNOT00000035838 | ENSRNOG00000025806 | Prr3 |  |
| rno-miR-150-3p | down | ENSRNOT00000032467 | ENSRNOG00000023494 | Tti2 | TELO2 interacting protein 2 |
| rno-novel-34-mature | down | ENSRNOT00000089057 | ENSRNOG00000060806 | Drd3 | dopamine receptor D3 |
| rno-miR-503-5p | down | ENSRNOT00000081920 | ENSRNOG00000015139 | F12 | coagulation factor XII |
| rno-miR-449c-5p | down | ENSRNOT00000081160 | ENSRNOG00000011208 | Rapsn | receptor-associated protein of the synapse |
| rno-novel-106-mature | down | ENSRNOT00000017829 | ENSRNOG00000013102 | Entpd2 | ectonucleoside triphosphate diphosphohydrolase 2 |
| rno-miR-351-3p | down | ENSRNOT00000019408 | ENSRNOG00000014452 | Zfhx3 | zinc finger homeobox 3 |
| rno-miR-351-3p | down | ENSRNOT00000068594 | ENSRNOG00000014452 | Zfhx3 | zinc finger homeobox 3 |
| rno-novel-106-mature | down | ENSRNOT00000011466 | ENSRNOG00000008445 | Dact1 | dishevelled-binding antagonist of beta-catenin 1 |
| rno-novel-79-star | down | ENSRNOT00000029728 | ENSRNOG00000021781 | Camk1 | calcium/calmodulin-dependent protein kinase I |
| rno-miR-150-3p | down | ENSRNOT00000025399 | ENSRNOG00000018687 | Fbxw2 | F-box and WD repeat domain containing 2 |
| rno-novel-106-mature | down | ENSRNOT00000021027 | ENSRNOG00000015557 | Umod | uromodulin [Source:RGD Symbol;Acc:3940] |
| rno-novel-53-mature | down | ENSRNOT00000049459 | ENSRNOG00000033984 | Ifnlr1 | interferon, lambda receptor 1 |
| rno-novel-34-mature | down | ENSRNOT00000033080 | ENSRNOG00000028350 | Arse | arylsulfatase E |
| rno-miR-330-3p | down | ENSRNOT00000034261 | ENSRNOG00000024972 | Cox10 | COX10 heme A:farnesyltransferase cytochrome c oxidase assembly factor |
| rno-miR-503-5p | down | ENSRNOT00000051991 | ENSRNOG00000030452 | Rimbp3 | RIMS binding protein 3 |
| rno-novel-53-mature | down | ENSRNOT00000004965 | ENSRNOG00000003722 | Dusp27 | dual specificity phosphatase 27 (putative) |
| rno-miR-330-3p | down | ENSRNOT00000086131 | ENSRNOG00000055344 | Trnau1ap | tRNA selenocysteine 1 associated protein 1 |
| rno-novel-6-mature | down | ENSRNOT00000003369 | ENSRNOG00000002474 | Tom1l1 | target of myb1 like 1 membrane trafficking protein |
| rno-novel-106-mature | down | ENSRNOT00000063870 | ENSRNOG00000002979 | Tbx19 | T-box 19 |
| rno-miR-449c-5p | down | ENSRNOT00000018240 | ENSRNOG00000013514 | Maf1 | MAF1 homolog, negative regulator of RNA polymerase III |
| rno-miR-449c-5p | down | ENSRNOT00000080604 | ENSRNOG00000005094 | C1qtnf7 | C1q and tumor necrosis factor related protein 7 |
| rno-miR-330-3p | down | ENSRNOT00000089869 | ENSRNOG00000025806 | Prr3 | proline rich 3 |
| rno-novel-106-mature | down | ENSRNOT00000042366 | ENSRNOG00000043267 | Mrm2 | mitochondrial rRNA methyltransferase 2 |
| rno-miR-17-1-3p | down | ENSRNOT00000013284 | ENSRNOG00000009871 | Piwil2 | piwi-like RNA-mediated gene silencing 2 |
| rno-novel-106-mature | down | ENSRNOT00000028752 | ENSRNOG00000021175 | Otub1 | OTU deubiquitinase, ubiquitin aldehyde binding 1 |
| rno-miR-150-3p | down | ENSRNOT00000025885 | ENSRNOG00000038459 | Ulk3 | unc-51 like kinase 3 [Source:RGD Symbol;Acc:1587417] |
| rno-miR-150-3p | down | ENSRNOT00000088306 | ENSRNOG00000037480 | Ddx51 | DEAD-box helicase 51 |
| rno-miR-150-3p | down | ENSRNOT00000068621 | ENSRNOG00000037480 | Ddx51 | DEAD-box helicase 51 |
| rno-miR-500-5p | down | ENSRNOT00000009544 | ENSRNOG00000006940 | Ncf4 | neutrophil cytosolic factor 4 |
| rno-miR-547-5p | down | ENSRNOT00000028650 | ENSRNOG00000021096 | Tmem143 | transmembrane protein 143 |
| rno-novel-106-mature | down | ENSRNOT00000078005 | ENSRNOG00000052111 | Sugp1 | SURP and G patch domain containing 1 |
| rno-novel-18-mature | down | ENSRNOT00000020910 | ENSRNOG00000015594 | Rftn2 | raftlin family member 2 |
| rno-miR-150-3p | down | ENSRNOT00000018890 | ENSRNOG00000013840 | Ankrd2 | ankyrin repeat domain 2 |
| rno-novel-6-mature | down | ENSRNOT00000016236 | ENSRNOG00000012199 | Sox2 | SRY box 2 |
| rno-novel-34-mature | down | ENSRNOT00000077651 | ENSRNOG00000055371 | Sptbn4 | spectrin, beta, non-erythrocytic 4 |
| rno-novel-34-mature | down | ENSRNOT00000033396 | ENSRNOG00000022393 | Faap24 | Fanconi anemia core complex associated protein 24 |
| rno-miR-150-5p | down | ENSRNOT00000004223 | ENSRNOG00000003132 | Mip | major intrinsic protein of lens fiber |
| rno-miR-201-3p | down | ENSRNOT00000007174 | ENSRNOG00000005250 | Abcg5 | ATP binding cassette subfamily G member 5 |
| rno-novel-6-mature | down | ENSRNOT00000006969 | ENSRNOG00000005093 | Lgr6 | leucine-rich repeat-containing G protein-coupled receptor 6 |
| rno-miR-17-1-3p | down | ENSRNOT00000082704 | ENSRNOG00000006400 | Tbc1d14 | TBC1 domain family, member 14 |
| rno-novel-1-star | down | ENSRNOT00000076680 | ENSRNOG00000005753 | Alg13 | ALG13, UDP-N-acetylglucosaminyltransferase subunit |
| rno-novel-53-mature | down | ENSRNOT00000090155 | ENSRNOG00000059604 | AABR07012274.1 |  |
| rno-novel-106-mature | down | ENSRNOT00000027973 | ENSRNOG00000020608 | Ppan | peter pan homolog (Drosophila) |
| rno-novel-106-mature | down | ENSRNOT00000007726 | ENSRNOG00000005747 | Il27ra | interleukin 27 receptor subunit alpha |
| rno-miR-150-3p | down | ENSRNOT00000026518 | ENSRNOG00000019522 | Narfl | nuclear prelamin A recognition factor-like |
| rno-miR-351-5p | down | ENSRNOT00000078244 | ENSRNOG00000020497 | Plekha1 | pleckstrin homology domain containing A1 |
| rno-novel-106-mature | down | ENSRNOT00000001162 | ENSRNOG00000000871 | Cd40lg | CD40 ligand |
| rno-miR-547-5p | down | ENSRNOT00000082917 | ENSRNOG00000021096 | Tmem143 | transmembrane protein 143 |
| rno-novel-34-mature | down | ENSRNOT00000060097 | ENSRNOG00000020030 | Crlf1 | cytokine receptor-like factor 1 |
| rno-novel-53-mature | down | ENSRNOT00000039057 | ENSRNOG00000001271 | Card6 | caspase recruitment domain family, member 6 |
| rno-novel-34-mature | down | ENSRNOT00000011486 | ENSRNOG00000008638 | Angptl3 | angiopoietin-like 3 |
| rno-novel-106-mature | down | ENSRNOT00000004834 | ENSRNOG00000003635 | Disp1 | dispatched RND transporter family member 1 |
| rno-novel-34-mature | down | ENSRNOT00000019389 | ENSRNOG00000014460 | Hivep1 | human immunodeficiency virus type I enhancer binding protein 1 |
| rno-miR-449c-5p | down | ENSRNOT00000092736 | ENSRNOG00000018899 | C5 | complement C5 |
| rno-miR-449c-5p | down | ENSRNOT00000019258 | ENSRNOG00000014340 | Pdcd7 | programmed cell death 7 |
| rno-novel-34-mature | down | ENSRNOT00000012379 | ENSRNOG00000009227 | Aplnr | apelin receptor |
| rno-miR-201-3p | down | ENSRNOT00000017758 | ENSRNOG00000013269 | Tnfsf10 | tumor necrosis factor superfamily member 10 |
| rno-miR-150-3p | down | ENSRNOT00000081206 | ENSRNOG00000054274 | Cacnb3 | calcium voltage-gated channel auxiliary subunit beta 3 |
| rno-miR-449c-5p | down | ENSRNOT00000090368 | ENSRNOG00000033343 | Prss36 | protease, serine, 36 |
| rno-novel-53-mature | down | ENSRNOT00000079333 | ENSRNOG00000001271 | Card6 | caspase recruitment domain family, member 6 |
| rno-novel-106-mature | down | ENSRNOT00000081036 | ENSRNOG00000006314 | Zbp1 | Z-DNA binding protein 1 |
| rno-miR-351-5p | down | ENSRNOT00000070864 | ENSRNOG00000047756 | Mef2a | myocyte enhancer factor 2a |
| rno-miR-503-5p | down | ENSRNOT00000066586 | ENSRNOG00000015139 | F12 | coagulation factor XII |
| rno-miR-351-5p | down | ENSRNOT00000078300 | ENSRNOG00000011476 | Nars2 | asparaginyl-tRNA synthetase 2 (mitochondrial)(putative) |
| rno-miR-150-5p | down | ENSRNOT00000092294 | ENSRNOG00000012297 | 8-Mar | membrane associated ring-CH-type finger 8 |
| rno-miR-449c-5p | down | ENSRNOT00000026587 | ENSRNOG00000033343 | Prss36 | protease, serine, 36 |
| rno-novel-34-mature | down | ENSRNOT00000024891 | ENSRNOG00000018000 | Ranbp10 | RAN binding protein 10 |
| rno-novel-34-mature | down | ENSRNOT00000090456 | ENSRNOG00000059683 | Mpp2 | membrane palmitoylated protein 2 |
| rno-novel-6-mature | down | ENSRNOT00000026259 | ENSRNOG00000019418 | Lrrc4b | leucine rich repeat containing 4B |
| rno-miR-351-5p | down | ENSRNOT00000027836 | ENSRNOG00000020497 | Plekha1 | pleckstrin homology domain containing A1 |
| rno-miR-351-5p | down | ENSRNOT00000072915 | ENSRNOG00000047756 | Mef2a | myocyte enhancer factor 2a |
| rno-novel-53-mature | down | ENSRNOT00000058219 | ENSRNOG00000038241 | LOC100359752 | hypothetical protein LOC100359752 |
| rno-miR-150-5p | down | ENSRNOT00000010035 | ENSRNOG00000007610 | Gdf11 | growth differentiation factor 11 |
| rno-novel-34-mature | down | ENSRNOT00000039852 | ENSRNOG00000028512 | Ilvbl | ilvB acetolactate synthase like |
| rno-novel-18-mature | down | ENSRNOT00000055279 | ENSRNOG00000036834 | Gpr84 | G protein-coupled receptor 84 |
| rno-novel-6-mature | down | ENSRNOT00000001059 | ENSRNOG00000000808 | Hsf2 | heat shock transcription factor 2 |
| rno-miR-150-3p | down | ENSRNOT00000008764 | ENSRNOG00000006420 | Rbm38 | RNA binding motif protein 38 |
| rno-novel-34-mature | down | ENSRNOT00000028585 | ENSRNOG00000021053 | Lsr | lipolysis stimulated lipoprotein receptor |
| rno-novel-6-mature | down | ENSRNOT00000044543 | ENSRNOG00000027311 | Nutm2f | NUT family member 2F |
| rno-novel-106-mature | down | ENSRNOT00000057058 | ENSRNOG00000009722 | Chd3 | chromodomain helicase DNA binding protein 3 |
| rno-miR-351-5p | down | ENSRNOT00000077526 | ENSRNOG00000056038 | Ehbp1l1 | EH domain binding protein 1-like 1 |
| rno-miR-503-5p | down | ENSRNOT00000005050 | ENSRNOG00000003777 | Chrne | cholinergic receptor nicotinic epsilon subunit |
| rno-novel-53-mature | down | ENSRNOT00000077526 | ENSRNOG00000056038 | Ehbp1l1 | EH domain binding protein 1-like 1 |
| rno-miR-150-5p | down | ENSRNOT00000024077 | ENSRNOG00000017679 | Cckbr | cholecystokinin B receptor |
| rno-miR-503-5p | down | ENSRNOT00000085517 | ENSRNOG00000051619 | Asb2 | ankyrin repeat and SOCS box-containing 2 |
| rno-miR-351-3p | down | ENSRNOT00000049044 | ENSRNOG00000029366 | Prrt2 | proline-rich transmembrane protein 2 |
| rno-miR-351-3p | down | ENSRNOT00000060355 | ENSRNOG00000023403 | Gtpbp3 | GTP binding protein 3 |
| rno-novel-53-mature | down | ENSRNOT00000050330 | ENSRNOG00000029071 | Unc5c | unc-5 netrin receptor C |
| rno-novel-34-mature | down | ENSRNOT00000092150 | ENSRNOG00000059683 | Mpp2 | membrane palmitoylated protein 2 |
| rno-miR-201-3p | down | ENSRNOT00000009496 | ENSRNOG00000006490 | Neo1 | neogenin 1 |
| rno-miR-342-5p | down | ENSRNOT00000066683 | ENSRNOG00000010409 | Nol6 | nucleolar protein 6 |
| rno-novel-106-mature | down | ENSRNOT00000068774 | ENSRNOG00000021833 | Myrfl | myelin regulatory factor-like |
| rno-novel-79-star | down | ENSRNOT00000024020 | ENSRNOG00000017612 | Vps35 | VPS35 retromer complex component |
| rno-novel-106-mature | down | ENSRNOT00000029127 | ENSRNOG00000023856 | Agxt | alanine-glyoxylate aminotransferase |
| rno-miR-351-5p | down | ENSRNOT00000083656 | ENSRNOG00000027564 | Tsga10ip | testis specific 10 interacting protein |
| rno-novel-34-mature | down | ENSRNOT00000044678 | ENSRNOG00000021053 | Lsr | lipolysis stimulated lipoprotein receptor |
| rno-miR-330-3p | down | ENSRNOT00000018897 | ENSRNOG00000013968 | Comtd1 | catechol-O-methyltransferase domain containing 1 |
| rno-novel-34-mature | down | ENSRNOT00000020955 | ENSRNOG00000015417 | Kansl3 | KAT8 regulatory NSL complex subunit 3 |
| rno-miR-547-5p | down | ENSRNOT00000037203 | ENSRNOG00000025430 | Ddx18 | DEAD-box helicase 18 |
| rno-novel-34-mature | down | ENSRNOT00000006519 | ENSRNOG00000004834 | Llgl2 | LLGL2, scribble cell polarity complex component |
| rno-novel-106-mature | down | ENSRNOT00000011824 | ENSRNOG00000008898 | Trmt44 | tRNA methyltransferase 44 homolog (S. cerevisiae) |
| rno-miR-17-1-3p | down | ENSRNOT00000004939 | ENSRNOG00000003717 | Cnih4 | cornichon family AMPA receptor auxiliary protein 4 |
| rno-miR-351-3p | down | ENSRNOT00000061498 | ENSRNOG00000012007 | Slc38a7 | solute carrier family 38, member 7 |
| rno-miR-150-5p | down | ENSRNOT00000001849 | ENSRNOG00000001367 | Gpc2 | glypican 2 |
| rno-miR-547-5p | down | ENSRNOT00000049538 | ENSRNOG00000030633 | Lmf2 | lipase maturation factor 2 |
| rno-miR-351-5p | down | ENSRNOT00000028081 | ENSRNOG00000020669 | Ovol1 | ovo like transcriptional repressor 1 |
| rno-novel-106-mature | down | ENSRNOT00000019500 | ENSRNOG00000014369 | Slc27a4 | solute carrier family 27 member 4 |
| rno-novel-53-mature | down | ENSRNOT00000087678 | ENSRNOG00000009440 | Gucy1b2 | guanylate cyclase 1 soluble subunit beta 2 |
| rno-novel-34-mature | down | ENSRNOT00000043971 | ENSRNOG00000029134 | Adgrl1 | adhesion G protein-coupled receptor L1 |
| rno-miR-150-3p | down | ENSRNOT00000057740 | ENSRNOG00000019504 | Grtp1 | growth hormone regulated TBC protein 1 |
| rno-miR-351-5p | down | ENSRNOT00000016376 | ENSRNOG00000011945 | Cyfip1 | cytoplasmic FMR1 interacting protein 1 |
| rno-novel-53-mature | down | ENSRNOT00000082287 | ENSRNOG00000029071 | Unc5c | unc-5 netrin receptor C |
| rno-novel-34-mature | down | ENSRNOT00000085260 | ENSRNOG00000015417 | Kansl3 | KAT8 regulatory NSL complex subunit 3 |
| rno-miR-150-5p | down | ENSRNOT00000085073 | ENSRNOG00000004483 | Ptprr | protein tyrosine phosphatase, receptor type, R |
| rno-miR-150-3p | down | ENSRNOT00000021729 | ENSRNOG00000016239 | Zadh2 | zinc binding alcohol dehydrogenase, domain containing 2 |
| rno-novel-106-mature | down | ENSRNOT00000014132 | ENSRNOG00000010646 | Tmem229b | transmembrane protein 229B |
| rno-novel-34-mature | down | ENSRNOT00000083452 | ENSRNOG00000011815 | Sgk1 | serum/glucocorticoid regulated kinase 1 |
| rno-miR-150-5p | down | ENSRNOT00000036940 | ENSRNOG00000022725 | Iba57 | IBA57 homolog, iron-sulfur cluster assembly |
| rno-miR-503-5p | down | ENSRNOT00000034157 | ENSRNOG00000022249 | Mllt10 | myeloid/lymphoid or mixed-lineage leukemia; translocated to, 10 |
| rno-miR-342-5p | down | ENSRNOT00000045107 | ENSRNOG00000029570 | Fam222b | family with sequence similarity 222, member B |
| rno-miR-330-3p | down | ENSRNOT00000078538 | ENSRNOG00000025806 | Prr3 | proline rich 3 |
| rno-miR-322-5p | down | ENSRNOT00000016420 | ENSRNOG00000012333 | Kbtbd11 | kelch repeat and BTB domain containing 11 |
| rno-miR-652-3p | down | ENSRNOT00000019350 | ENSRNOG00000014454 | Ap1m1 | adaptor-related protein complex 1, mu 1 subunit |
| rno-miR-150-5p | down | ENSRNOT00000017294 | ENSRNOG00000012297 | 8-Mar | membrane associated ring-CH-type finger 8 |
| rno-miR-342-5p | down | ENSRNOT00000019406 | ENSRNOG00000014296 | Syt10 | synaptotagmin 10 |
| rno-novel-106-mature | down | ENSRNOT00000058495 | ENSRNOG00000025371 | Spry1 | sprouty RTK signaling antagonist 1 |
| rno-novel-34-mature | down | ENSRNOT00000089123 | ENSRNOG00000002303 | Kcnj12 | potassium voltage-gated channel subfamily J member 12 |
| rno-miR-17-1-3p | down | ENSRNOT00000024430 | ENSRNOG00000018087 | Vim | vimentin |
| rno-miR-449c-5p | down | ENSRNOT00000066961 | ENSRNOG00000017925 | Gapvd1 | GTPase activating protein and VPS9 domains 1 |
| rno-novel-6-mature | down | ENSRNOT00000066961 | ENSRNOG00000017925 | Gapvd1 | GTPase activating protein and VPS9 domains 1 |
| rno-novel-53-mature | down | ENSRNOT00000074193 | ENSRNOG00000046482 | Znf750 | zinc finger protein 750 |
| rno-miR-503-5p | down | ENSRNOT00000027995 | ENSRNOG00000020607 | Bckdha | branched chain ketoacid dehydrogenase E1, alpha polypeptide |
| rno-miR-150-5p | down | ENSRNOT00000089941 | ENSRNOG00000016253 | Slc6a18 | solute carrier family 6 member 18 |
| rno-miR-351-5p | down | ENSRNOT00000088459 | ENSRNOG00000011945 | Cyfip1 | cytoplasmic FMR1 interacting protein 1 |
| rno-miR-150-3p | down | ENSRNOT00000017226 | ENSRNOG00000012860 | Tmem184c | transmembrane protein 184C |
| rno-novel-79-star | down | ENSRNOT00000088712 | ENSRNOG00000010653 | Elmsan1 | ELM2 and Myb/SANT domain containing 1 |
| rno-miR-351-5p | down | ENSRNOT00000045233 | ENSRNOG00000030888 | Rela | RELA proto-oncogene, NF-kB subunit |
| rno-miR-150-3p | down | ENSRNOT00000035238 | ENSRNOG00000022196 | Bmpr2 | bone morphogenetic protein receptor type 2 |
| rno-miR-351-3p | down | ENSRNOT00000001016 | ENSRNOG00000000782 | Trim10 | tripartite motif-containing 10 |
| rno-miR-503-5p | down | ENSRNOT00000032792 | ENSRNOG00000023861 | Snap91 | synaptosomal-associated protein 91 |
| rno-miR-330-3p | down | ENSRNOT00000059246 | ENSRNOG00000005504 | Pkp4 | plakophilin 4 |
| rno-miR-150-5p | down | ENSRNOT00000005901 | ENSRNOG00000004466 | Fam210b | family with sequence similarity 210, member B |
| rno-miR-330-3p | down | ENSRNOT00000036151 | ENSRNOG00000028415 | Cdc20 | cell division cycle 20 |
| rno-miR-449c-5p | down | ENSRNOT00000007978 | ENSRNOG00000006098 | Sox30 | SRY box 30 |
| rno-novel-106-mature | down | ENSRNOT00000032263 | ENSRNOG00000021899 | Tmem115 | transmembrane protein 115 |
| rno-miR-449c-5p | down | ENSRNOT00000064495 | ENSRNOG00000005094 | C1qtnf7 | C1q and tumor necrosis factor related protein 7 |
| rno-novel-106-mature | down | ENSRNOT00000016997 | ENSRNOG00000012655 | Adamts6 | ADAM metallopeptidase with thrombospondin type 1 motif, 6 |
| rno-miR-150-5p | down | ENSRNOT00000092477 | ENSRNOG00000012297 | 8-Mar | membrane associated ring-CH-type finger 8 |
| rno-novel-106-mature | down | ENSRNOT00000007312 | ENSRNOG00000005515 | Rhbdl3 | rhomboid like 3 |
| rno-novel-34-mature | down | ENSRNOT00000065426 | ENSRNOG00000001580 | Hoxd9 | homeo box D9 |
| rno-novel-106-mature | down | ENSRNOT00000015469 | ENSRNOG00000011530 | Fam188b | family with sequence similarity 188, member B |
| rno-miR-351-5p | down | ENSRNOT00000051853 | ENSRNOG00000015902 | Cpxm2 | carboxypeptidase X (M14 family), member 2 |
| rno-novel-53-mature | down | ENSRNOT00000022020 | ENSRNOG00000016177 | Scara3 | scavenger receptor class A, member 3 |
| rno-novel-106-mature | down | ENSRNOT00000085630 | ENSRNOG00000005747 | Il27ra | interleukin 27 receptor subunit alpha |
| rno-miR-150-3p | down | ENSRNOT00000085210 | ENSRNOG00000047296 | Bptf | bromodomain PHD finger transcription factor |
| rno-novel-53-mature | down | ENSRNOT00000015198 | ENSRNOG00000011054 | Laptm5 | lysosomal protein transmembrane 5 |
| rno-novel-34-mature | down | ENSRNOT00000001807 | ENSRNOG00000001337 | Setd1b | SET domain containing 1B |
| rno-miR-503-5p | down | ENSRNOT00000084437 | ENSRNOG00000023861 | Snap91 | synaptosomal-associated protein 91 |
| rno-novel-18-mature | down | ENSRNOT00000042227 | ENSRNOG00000018830 | Aff3 | AF4/FMR2 family, member 3 |
| rno-miR-330-3p | down | ENSRNOT00000026718 | ENSRNOG00000019708 | Ctsf | cathepsin F |
| rno-miR-351-3p | down | ENSRNOT00000093039 | ENSRNOG00000007467 | Ace3 | angiotensin I converting enzyme (peptidyl-dipeptidase A) 3 |
| rno-miR-150-3p | down | ENSRNOT00000021979 | ENSRNOG00000016242 | Fzd1 | frizzled class receptor 1 |
| rno-novel-106-mature | down | ENSRNOT00000077871 | ENSRNOG00000021725 | Unc119b | unc-119 lipid binding chaperone B |
| rno-novel-34-mature | down | ENSRNOT00000065494 | ENSRNOG00000016366 | Colec12 | collectin sub-family member 12 |
| rno-miR-351-5p | down | ENSRNOT00000075588 | ENSRNOG00000049324 | LOC100911766 | tetraspanin-4-like |
| rno-novel-106-mature | down | ENSRNOT00000000205 | ENSRNOG00000024631 | Chadl | chondroadherin-like |
| rno-miR-150-3p | down | ENSRNOT00000065270 | ENSRNOG00000036835 | Copz1 | coatomer protein complex, subunit zeta 1 |
| rno-miR-330-3p | down | ENSRNOT00000052087 | ENSRNOG00000029095 | Trabd | TraB domain containing |
| rno-miR-351-3p | down | ENSRNOT00000074007 | ENSRNOG00000007467 | Ace3 | angiotensin I converting enzyme (peptidyl-dipeptidase A) 3 |
| rno-novel-53-mature | down | ENSRNOT00000042681 | ENSRNOG00000033608 | Cd276 | Cd276 molecule |
| rno-miR-17-1-3p | down | ENSRNOT00000087684 | ENSRNOG00000010188 | Satb2 | SATB homeobox 2 |
| rno-novel-106-mature | down | ENSRNOT00000085444 | ENSRNOG00000057794 | Adamts5 | ADAM metallopeptidase with thrombospondin type 1 motif, 5 |
| rno-miR-351-3p | down | ENSRNOT00000072241 | ENSRNOG00000050204 | Naif1 | nuclear apoptosis inducing factor 1 |
| rno-miR-500-5p | down | ENSRNOT00000056872 | ENSRNOG00000015430 | Nlgn2 | neuroligin 2 |
| rno-miR-500-5p | down | ENSRNOT00000092662 | ENSRNOG00000015430 | Nlgn2 | neuroligin 2 |
| rno-miR-449c-5p | down | ENSRNOT00000086096 | ENSRNOG00000036677 | Slc16a3 | solute carrier family 16 member 3 |
| rno-miR-17-1-3p | down | ENSRNOT00000008888 | ENSRNOG00000005390 | Nup210 | nucleoporin 210 |
| rno-miR-17-1-3p | down | ENSRNOT00000019214 | ENSRNOG00000014302 | Dlgap3 | DLG associated protein 3 |
| rno-miR-503-5p | down | ENSRNOT00000005366 | ENSRNOG00000003948 | Llgl1 | LLGL1, scribble cell polarity complex component |
| rno-miR-150-3p | down | ENSRNOT00000050528 | ENSRNOG00000025892 | Set | SET nuclear proto-oncogene |
| rno-novel-34-mature | down | ENSRNOT00000045699 | ENSRNOG00000029134 | Adgrl1 | adhesion G protein-coupled receptor L1 |
| rno-miR-503-5p | down | ENSRNOT00000057084 | ENSRNOG00000023453 | Lrba | LPS responsive beige-like anchor protein |
| rno-novel-34-mature | down | ENSRNOT00000015307 | ENSRNOG00000011423 | Pitx1 | paired-like homeodomain 1 |
| rno-miR-201-3p | down | ENSRNOT00000089009 | ENSRNOG00000026435 | Arid3a | AT-rich interaction domain 3A |
| rno-miR-450b-3p | down | ENSRNOT00000028434 | ENSRNOG00000020947 | Egln2 | egl-9 family hypoxia-inducible factor 2 |
| rno-miR-449c-5p | down | ENSRNOT00000054939 | ENSRNOG00000036677 | Slc16a3 | solute carrier family 16 member 3 |
| rno-novel-34-mature | down | ENSRNOT00000085940 | ENSRNOG00000058662 | Ano8 | anoctamin 8 |
| rno-miR-150-3p | down | ENSRNOT00000059522 | ENSRNOG00000013603 | Dffa | DNA fragmentation factor subunit alpha |
| rno-novel-34-mature | down | ENSRNOT00000057953 | ENSRNOG00000002303 | Kcnj12 | potassium voltage-gated channel subfamily J member 12 |
| rno-novel-106-mature | down | ENSRNOT00000005839 | ENSRNOG00000004392 | Aatk | apoptosis-associated tyrosine kinase |
| rno-novel-106-mature | down | ENSRNOT00000014073 | ENSRNOG00000010478 | LOC500712 | Ab1-233 |
| rno-miR-150-3p | down | ENSRNOT00000077797 | ENSRNOG00000025892 | Set | SET nuclear proto-oncogene |
| rno-novel-34-mature | down | ENSRNOT00000040898 | ENSRNOG00000033527 | Pappa | pregnancy-associated plasma protein A |
| rno-miR-201-3p | down | ENSRNOT00000026708 | ENSRNOG00000026435 | Arid3a | AT-rich interaction domain 3A |
| rno-miR-150-3p | down | ENSRNOT00000083851 | ENSRNOG00000001227 | Adarb1 | adenosine deaminase, RNA-specific, B1 |
| rno-miR-351-5p | down | ENSRNOT00000081567 | ENSRNOG00000020669 | Ovol1 | ovo like transcriptional repressor 1 |
| rno-novel-53-mature | down | ENSRNOT00000020855 | ENSRNOG00000015376 | Npas1 | neuronal PAS domain protein 1 |
| rno-novel-18-mature | down | ENSRNOT00000083256 | ENSRNOG00000018830 | Aff3 | AF4/FMR2 family, member 3 |
| rno-miR-330-3p | down | ENSRNOT00000092123 | ENSRNOG00000057620 | Slc6a8 | solute carrier family 6 member 8 |
| rno-miR-503-5p | down | ENSRNOT00000020428 | ENSRNOG00000015085 | Dmpk | dystrophia myotonica-protein kinase |
| rno-novel-106-mature | down | ENSRNOT00000026960 | ENSRNOG00000019891 | Sgta | small glutamine rich tetratricopeptide repeat containing alpha |
| rno-miR-351-5p | down | ENSRNOT00000016770 | ENSRNOG00000012552 | Ints4 | integrator complex subunit 4 |
| rno-novel-106-mature | down | ENSRNOT00000079769 | ENSRNOG00000007882 | Ablim2 | actin binding LIM protein family, member 2 |
| rno-miR-150-3p | down | ENSRNOT00000032158 | ENSRNOG00000025209 | Plxnd1 | plexin D1 |
| rno-miR-3585-5p | down | ENSRNOT00000037685 | ENSRNOG00000010748 | Mtus1 | microtubule associated tumor suppressor 1 |
| rno-miR-503-5p | down | ENSRNOT00000026700 | ENSRNOG00000049913 | Rbm12 | RNA binding motif protein 12 |
| rno-novel-34-mature | down | ENSRNOT00000076467 | ENSRNOG00000033527 | Pappa | pregnancy-associated plasma protein A |
| rno-miR-330-3p | down | ENSRNOT00000046691 | ENSRNOG00000006999 | Wdr78 | WD repeat domain 78 |
| rno-novel-106-mature | down | ENSRNOT00000056272 | ENSRNOG00000014837 | Emilin2 | elastin microfibril interfacer 2 |
| rno-novel-34-mature | down | ENSRNOT00000020388 | ENSRNOG00000015180 | Kdm8 | lysine demethylase 8 |
| rno-miR-150-5p | down | ENSRNOT00000018860 | ENSRNOG00000013981 | Ptpn5 | protein tyrosine phosphatase, non-receptor type 5 |
| rno-miR-547-5p | down | ENSRNOT00000005257 | ENSRNOG00000003931 | Arsg | arylsulfatase G |
| rno-miR-1912-3p | down | ENSRNOT00000090152 | ENSRNOG00000027833 | Fnip2 | folliculin interacting protein 2 |
| rno-miR-150-5p | down | ENSRNOT00000009377 | ENSRNOG00000006178 | Dync1h1 | dynein cytoplasmic 1 heavy chain 1 |
| rno-novel-106-mature | down | ENSRNOT00000024111 | ENSRNOG00000017930 | Lpcat1 | lysophosphatidylcholine acyltransferase 1 |
| rno-miR-503-5p | down | ENSRNOT00000057945 | ENSRNOG00000019779 | Disc1 | disrupted in schizophrenia 1 |
| rno-novel-106-mature | down | ENSRNOT00000087062 | ENSRNOG00000051854 | Enpep | glutamyl aminopeptidase |
| rno-miR-330-3p | down | ENSRNOT00000015452 | ENSRNOG00000011560 | Mtmr9 | myotubularin related protein 9 |
| rno-miR-150-3p | down | ENSRNOT00000004361 | ENSRNOG00000003268 | Maml1 | mastermind-like transcriptional coactivator 1 |
| rno-miR-150-5p | down | ENSRNOT00000022346 | ENSRNOG00000016253 | Slc6a18 | solute carrier family 6 member 18 |
| rno-miR-652-3p | down | ENSRNOT00000057978 | ENSRNOG00000014155 | Spg20 | spastic paraplegia 20 (Troyer syndrome) |
| rno-novel-106-mature | down | ENSRNOT00000044532 | ENSRNOG00000021023 | Mag | myelin-associated glycoprotein |
| rno-miR-330-3p | down | ENSRNOT00000079976 | ENSRNOG00000032311 | AABR07058658.1 |  |
| rno-novel-53-mature | down | ENSRNOT00000010043 | ENSRNOG00000007364 | Rab15 | RAB15, member RAS oncogene family |
| rno-miR-500-5p | down | ENSRNOT00000051970 | ENSRNOG00000028783 | Wdr35 | WD repeat domain 35 |
| rno-miR-150-3p | down | ENSRNOT00000020003 | ENSRNOG00000014668 | RGD621098 | similar to RIKEN cDNA D230025D16Rik |
| rno-novel-53-mature | down | ENSRNOT00000015104 | ENSRNOG00000011323 | Lgi3 | leucine-rich repeat LGI family, member 3 |
| rno-novel-34-mature | down | ENSRNOT00000076111 | ENSRNOG00000033527 | Pappa | pregnancy-associated plasma protein A |
| rno-novel-106-mature | down | ENSRNOT00000028544 | ENSRNOG00000021023 | Mag | myelin-associated glycoprotein |
| rno-miR-17-1-3p | down | ENSRNOT00000066923 | ENSRNOG00000019136 | Scamp2 | secretory carrier membrane protein 2 |
| rno-novel-106-mature | down | ENSRNOT00000084202 | ENSRNOG00000058461 | Sfpq | splicing factor proline and glutamine rich |
| rno-novel-106-mature | down | ENSRNOT00000089517 | ENSRNOG00000037851 | Spidr | scaffolding protein involved in DNA repair |
| rno-miR-322-5p | down | ENSRNOT00000006152 | ENSRNOG00000004489 | Adgre5 | adhesion G protein-coupled receptor E5 |
| rno-miR-351-5p | down | ENSRNOT00000028283 | ENSRNOG00000050994 | Cttn | cortactin |
| rno-miR-351-5p | down | ENSRNOT00000092916 | ENSRNOG00000047280 | Cttn | cortactin |
| rno-miR-503-5p | down | ENSRNOT00000088188 | ENSRNOG00000023453 | Lrba | LPS responsive beige-like anchor protein |
| rno-miR-503-5p | down | ENSRNOT00000088807 | ENSRNOG00000023453 | Lrba | LPS responsive beige-like anchor protein |
| rno-novel-6-mature | down | ENSRNOT00000088870 | ENSRNOG00000012811 | Spint1 | serine peptidase inhibitor, Kunitz type 1 |
| rno-novel-34-mature | down | ENSRNOT00000010157 | ENSRNOG00000007583 | Pygb | glycogen phosphorylase B |
| rno-novel-106-mature | down | ENSRNOT00000084685 | ENSRNOG00000010478 | LOC500712 | Ab1-233 |
| rno-miR-351-5p | down | ENSRNOT00000022852 | ENSRNOG00000017016 | Ubqlnl | ubiquilin-like |
| rno-miR-708-3p | down | ENSRNOT00000027507 | ENSRNOG00000020293 | Chrna10 | cholinergic receptor nicotinic alpha 10 subunit |
| rno-novel-6-mature | down | ENSRNOT00000017223 | ENSRNOG00000012811 | Spint1 | serine peptidase inhibitor, Kunitz type 1 |
| rno-novel-34-mature | down | ENSRNOT00000080170 | ENSRNOG00000059202 | Mia3 | MIA family member 3, ER export factor |
| rno-miR-150-5p | down | ENSRNOT00000003061 | ENSRNOG00000002248 | Fryl | FRY like transcription coactivator |
| rno-novel-34-mature | down | ENSRNOT00000080714 | ENSRNOG00000004834 | Llgl2 | LLGL2, scribble cell polarity complex component |
| rno-miR-351-5p | down | ENSRNOT00000031973 | ENSRNOG00000024429 | Peg12 | paternally expressed 12 |
| rno-miR-150-3p | down | ENSRNOT00000080170 | ENSRNOG00000059202 | Mia3 | MIA family member 3, ER export factor |
| rno-novel-106-mature | down | ENSRNOT00000031549 | ENSRNOG00000025808 | Aars2 | alanyl-tRNA synthetase 2, mitochondrial |
| rno-miR-150-3p | down | ENSRNOT00000001642 | ENSRNOG00000001227 | Adarb1 | adenosine deaminase, RNA-specific, B1 |
| rno-miR-150-5p | down | ENSRNOT00000073950 | ENSRNOG00000006178 | Dync1h1 | dynein cytoplasmic 1 heavy chain 1 |
| rno-novel-53-mature | down | ENSRNOT00000010134 | ENSRNOG00000007720 | Rnf26 | ring finger protein 26 |
| rno-novel-106-mature | down | ENSRNOT00000071381 | ENSRNOG00000047867 | Klhdc8b | kelch domain containing 8B |
| rno-novel-106-mature | down | ENSRNOT00000057136 | ENSRNOG00000007387 | Per1 | period circadian clock 1 |
| rno-miR-351-5p | down | ENSRNOT00000054859 | ENSRNOG00000047280 | Cttn | cortactin |
| rno-novel-53-mature | down | ENSRNOT00000001532 | ENSRNOG00000001156 | Msi1 | musashi RNA-binding protein 1 |
| rno-miR-17-1-3p | down | ENSRNOT00000016233 | ENSRNOG00000011636 | Dennd6a | DENN domain containing 6A |
| rno-novel-79-star | down | ENSRNOT00000014158 | ENSRNOG00000010653 | Elmsan1 | ELM2 and Myb/SANT domain containing 1 |
| rno-miR-17-1-3p | down | ENSRNOT00000085534 | ENSRNOG00000006400 | Tbc1d14 | TBC1 domain family, member 14 |
| rno-miR-449c-5p | down | ENSRNOT00000068026 | ENSRNOG00000011459 | Rhbdf2 | rhomboid 5 homolog 2 |
| rno-miR-509-5p | down | ENSRNOT00000011459 | ENSRNOG00000008000 | Syt13 | synaptotagmin 13 |
| rno-miR-17-1-3p | down | ENSRNOT00000077846 | ENSRNOG00000006400 | Tbc1d14 | TBC1 domain family, member 14 |
| rno-miR-503-5p | down | ENSRNOT00000047246 | ENSRNOG00000019723 | LOC100910990 | copine-1-like |
| rno-miR-17-1-3p | down | ENSRNOT00000088089 | ENSRNOG00000006729 | Slc24a4 | solute carrier family 24 member 4 |
| rno-miR-351-5p | down | ENSRNOT00000047669 | ENSRNOG00000050994 | Cttn | cortactin |
| rno-miR-547-5p | down | ENSRNOT00000005157 | ENSRNOG00000003880 | Tph2 | tryptophan hydroxylase 2 |
| rno-miR-150-3p | down | ENSRNOT00000012056 | ENSRNOG00000008829 | Sorbs3 | sorbin and SH3 domain containing 3 |
| rno-miR-351-3p | down | ENSRNOT00000015779 | ENSRNOG00000011859 | Eif5a2 | eukaryotic translation initiation factor 5A2 |
| rno-miR-503-5p | down | ENSRNOT00000060690 | ENSRNOG00000024349 | Cbarp | CACN beta subunit associated regulatory protein |
| rno-novel-34-mature | down | ENSRNOT00000018328 | ENSRNOG00000012956 | Tgm2 | transglutaminase 2 |
| rno-novel-106-mature | down | ENSRNOT00000015296 | ENSRNOG00000011387 | Tet3 | tet methylcytosine dioxygenase 3 |
| rno-miR-322-5p | down | ENSRNOT00000014444 | ENSRNOG00000010718 | Gpr153 | G protein-coupled receptor 153 |
| rno-miR-150-3p | down | ENSRNOT00000081995 | ENSRNOG00000052204 | Tbc1d24 | TBC1 domain family, member 24 |
| rno-miR-150-5p | down | ENSRNOT00000006401 | ENSRNOG00000004483 | Ptprr | protein tyrosine phosphatase, receptor type, R |
| rno-miR-150-5p | down | ENSRNOT00000023205 | ENSRNOG00000016848 | Fzd4 | frizzled class receptor 4 |
| rno-miR-351-5p | down | ENSRNOT00000000474 | ENSRNOG00000000417 | Numa1 | nuclear mitotic apparatus protein 1 |
| rno-novel-106-mature | down | ENSRNOT00000009497 | ENSRNOG00000007177 | Kdelc2 | KDEL motif containing 2 |
| rno-miR-150-5p | down | ENSRNOT00000074401 | ENSRNOG00000046984 | St6galnac6 | ST6 N-acetylgalactosaminide alpha-2,6-sialyltransferase 6 |
| rno-novel-34-mature | down | ENSRNOT00000035905 | ENSRNOG00000025926 | Heatr5b | HEAT repeat containing 5B |
| rno-miR-17-1-3p | down | ENSRNOT00000016273 | ENSRNOG00000011058 | Utrn | utrophin |
| rno-novel-34-mature | down | ENSRNOT00000013213 | ENSRNOG00000009930 | Pigo | phosphatidylinositol glycan anchor biosynthesis, class O |
| rno-miR-150-3p | down | ENSRNOT00000079955 | ENSRNOG00000001658 | Kcnj6 | potassium voltage-gated channel subfamily J member 6 |
| rno-miR-1912-3p | down | ENSRNOT00000038543 | ENSRNOG00000027833 | Fnip2 | folliculin interacting protein 2 |
| rno-novel-106-mature | down | ENSRNOT00000022428 | ENSRNOG00000016708 | E2f1 | E2F transcription factor 1 |
| rno-miR-17-1-3p | down | ENSRNOT00000086574 | ENSRNOG00000011058 | Utrn | utrophin |
| rno-miR-150-3p | down | ENSRNOT00000018970 | ENSRNOG00000014011 | Dll4 | delta like canonical Notch ligand 4 |
| rno-miR-150-3p | down | ENSRNOT00000015384 | ENSRNOG00000011451 | Lrp3 | LDL receptor related protein 3 |
| rno-miR-449c-5p | down | ENSRNOT00000036848 | ENSRNOG00000018412 | Sfi1 | SFI1 centrin binding protein |
| rno-novel-106-mature | down | ENSRNOT00000057502 | ENSRNOG00000037851 | Spidr | scaffolding protein involved in DNA repair |
| rno-novel-106-mature | down | ENSRNOT00000086350 | ENSRNOG00000060237 | Inhbb | inhibin beta B subunit |
| rno-novel-106-mature | down | ENSRNOT00000008055 | ENSRNOG00000005342 | Rassf5 | Ras association domain family member 5 |
| rno-miR-351-3p | down | ENSRNOT00000076729 | ENSRNOG00000011613 | Ppip5k2 | diphosphoinositol pentakisphosphate kinase 2 |
| rno-miR-330-3p | down | ENSRNOT00000050620 | ENSRNOG00000031093 | Mov10l1 | Mov10 RISC complex RNA helicase like 1 |
| rno-novel-53-mature | down | ENSRNOT00000010763 | ENSRNOG00000008024 | Pxk | PX domain containing serine/threonine kinase |
| rno-novel-106-mature | down | ENSRNOT00000077546 | ENSRNOG00000059260 | AABR07053509.2 |  |
| rno-miR-449c-5p | down | ENSRNOT00000025534 | ENSRNOG00000018899 | C5 | complement C5 |
| rno-novel-106-mature | down | ENSRNOT00000021141 | ENSRNOG00000015609 | Wdr27 | WD repeat domain 27 |
| rno-novel-53-mature | down | ENSRNOT00000012161 | ENSRNOG00000008944 | Ext2 | exostosin glycosyltransferase 2 |
| rno-novel-53-mature | down | ENSRNOT00000028963 | ENSRNOG00000027799 | Tmie | transmembrane inner ear |
| rno-miR-150-5p | down | ENSRNOT00000034096 | ENSRNOG00000024089 | Fndc3b | fibronectin type III domain containing 3B |
| rno-miR-351-5p | down | ENSRNOT00000055249 | ENSRNOG00000000417 | Numa1 | nuclear mitotic apparatus protein 1 |
| rno-miR-17-1-3p | down | ENSRNOT00000058414 | ENSRNOG00000006400 | Tbc1d14 | TBC1 domain family, member 14 |
| rno-miR-17-1-3p | down | ENSRNOT00000086761 | ENSRNOG00000061153 | Hrh3 | histamine receptor H3 |
| rno-novel-34-mature | down | ENSRNOT00000015499 | ENSRNOG00000011566 | Cecr2 | CECR2, histone acetyl-lysine reader |
| rno-miR-330-3p | down | ENSRNOT00000000653 | ENSRNOG00000000543 | Frk | fyn-related Src family tyrosine kinase |
| rno-miR-351-5p | down | ENSRNOT00000022176 | ENSRNOG00000016206 | Uvrag | UV radiation resistance associated |
| rno-novel-34-mature | down | ENSRNOT00000087834 | ENSRNOG00000054360 | Tspan11 | tetraspanin 11 |
| rno-miR-150-3p | down | ENSRNOT00000027135 | ENSRNOG00000019985 | Asic4 | acid sensing ion channel subunit family member 4 |
| rno-novel-106-mature | down | ENSRNOT00000014489 | ENSRNOG00000010896 | Tprn | taperin |
| rno-miR-17-1-3p | down | ENSRNOT00000008886 | ENSRNOG00000006729 | Slc24a4 | solute carrier family 24 member 4 |
| rno-miR-351-5p | down | ENSRNOT00000022486 | ENSRNOG00000016790 | Kmt5b | lysine methyltransferase 5B |
| rno-novel-53-mature | down | ENSRNOT00000090597 | ENSRNOG00000060665 | Afap1 | actin filament associated protein 1 |
| rno-miR-330-3p | down | ENSRNOT00000021188 | ENSRNOG00000015718 | RGD1307461 | similar to RIKEN cDNA 6430571L13 gene; similar to g20 protein |
| rno-novel-106-mature | down | ENSRNOT00000058301 | ENSRNOG00000038297 | Plekhd1 | pleckstrin homology and coiled-coil domain containing D1 |
| rno-miR-351-3p | down | ENSRNOT00000083144 | ENSRNOG00000060052 | Tdrd15 | tudor domain containing 15 |
| rno-novel-18-mature | down | ENSRNOT00000087434 | ENSRNOG00000027869 | Sox5 | SRY box 5 |
| rno-novel-106-mature | down | ENSRNOT00000000737 | ENSRNOG00000000599 | Lama4 | laminin subunit alpha 4 |
| rno-novel-53-mature | down | ENSRNOT00000083743 | ENSRNOG00000008944 | Ext2 | exostosin glycosyltransferase 2 |
| rno-miR-351-5p | down | ENSRNOT00000028967 | ENSRNOG00000019846 | Muc5b | mucin 5B, oligomeric mucus/gel-forming |
| rno-miR-351-3p | down | ENSRNOT00000068299 | ENSRNOG00000028844 | Slc9a5 | solute carrier family 9 member A5 |
| rno-novel-106-mature | down | ENSRNOT00000082725 | ENSRNOG00000057706 | Kdm5c | lysine demethylase 5C |
| rno-miR-330-3p | down | ENSRNOT00000042686 | ENSRNOG00000031093 | Mov10l1 | Mov10 RISC complex RNA helicase like 1 |
| rno-novel-6-mature | down | ENSRNOT00000090468 | ENSRNOG00000053869 | Ppp1r3d | protein phosphatase 1, regulatory subunit 3D |
| rno-miR-503-5p | down | ENSRNOT00000017363 | ENSRNOG00000012862 | Spsb4 | splA/ryanodine receptor domain and SOCS box containing 4 |
| rno-miR-150-3p | down | ENSRNOT00000012757 | ENSRNOG00000009448 | Papln | papilin, proteoglycan-like sulfated glycoprotein |
| rno-novel-79-star | down | ENSRNOT00000086276 | ENSRNOG00000056678 | Nckap5l | NCK-associated protein 5-like |
| rno-miR-503-5p | down | ENSRNOT00000081127 | ENSRNOG00000008598 | Btbd7 | BTB domain containing 7 |
| rno-novel-53-mature | down | ENSRNOT00000080945 | ENSRNOG00000061230 | L1cam | L1 cell adhesion molecule |
| rno-novel-106-mature | down | ENSRNOT00000023478 | ENSRNOG00000017431 | RGD1304884 | similar to RIKEN cDNA 6430548M08 |
| rno-miR-503-5p | down | ENSRNOT00000024632 | ENSRNOG00000017965 | Afg3l2 | AFG3 like matrix AAA peptidase subunit 2 |
| rno-novel-106-mature | down | ENSRNOT00000007828 | ENSRNOG00000005783 | Dlg5 | discs large MAGUK scaffold protein 5 |
| rno-miR-351-5p | down | ENSRNOT00000016289 | ENSRNOG00000011680 | Il16 | interleukin 16 |
| rno-miR-503-3p | down | ENSRNOT00000023418 | ENSRNOG00000023453 | Lrba | LPS responsive beige-like anchor protein |
| rno-novel-53-mature | down | ENSRNOT00000056272 | ENSRNOG00000014837 | Emilin2 | elastin microfibril interfacer 2 |
| rno-novel-106-mature | down | ENSRNOT00000025043 | ENSRNOG00000018462 | Rabep2 | rabaptin, RAB GTPase binding effector protein 2 |
| rno-miR-150-5p | down | ENSRNOT00000023555 | ENSRNOG00000017403 | Apobr | apolipoprotein B receptor |
| rno-novel-106-mature | down | ENSRNOT00000061735 | ENSRNOG00000018574 | Qsox2 | quiescin sulfhydryl oxidase 2 |
| rno-novel-79-star | down | ENSRNOT00000081434 | ENSRNOG00000056678 | Nckap5l | NCK-associated protein 5-like |
| rno-novel-106-mature | down | ENSRNOT00000003615 | ENSRNOG00000002664 | Emp2 | epithelial membrane protein 2 |
| rno-miR-449c-5p | down | ENSRNOT00000027493 | ENSRNOG00000020272 | RGD1310209 | similar to KIAA1324 protein |
| rno-novel-1-star | down | ENSRNOT00000076472 | ENSRNOG00000014851 | Col4a4 | collagen type IV alpha 4 chain |
| rno-novel-18-mature | down | ENSRNOT00000055542 | ENSRNOG00000027869 | Sox5 | SRY box 5 |
| rno-novel-106-mature | down | ENSRNOT00000027312 | ENSRNOG00000027924 | Fam83g | family with sequence similarity 83, member G |
| rno-miR-150-3p | down | ENSRNOT00000089447 | ENSRNOG00000017154 | Atp11a | ATPase phospholipid transporting 11A |
| rno-miR-450b-3p | down | ENSRNOT00000059806 | ENSRNOG00000024201 | Fer1l5 | fer-1-like family member 5 |
| rno-miR-150-5p | down | ENSRNOT00000033491 | ENSRNOG00000026857 | Kif7 | kinesin family member 7 |
| rno-novel-106-mature | down | ENSRNOT00000043872 | ENSRNOG00000033883 | Stard8 | StAR-related lipid transfer domain containing 8 |
| rno-miR-330-3p | down | ENSRNOT00000072386 | ENSRNOG00000049895 | LOC100910143 | GPI ethanolamine phosphate transferase 2-like |
| rno-miR-330-3p | down | ENSRNOT00000075415 | ENSRNOG00000050374 | Pigg | phosphatidylinositol glycan anchor biosynthesis, class G |
| rno-novel-106-mature | down | ENSRNOT00000013552 | ENSRNOG00000009891 | Pcf11 | PCF11 cleavage and polyadenylation factor subunit |
| rno-novel-106-mature | down | ENSRNOT00000081662 | ENSRNOG00000033883 | Stard8 | StAR-related lipid transfer domain containing 8 |
| rno-miR-351-5p | down | ENSRNOT00000025699 | ENSRNOG00000018974 | Ern2 | endoplasmic reticulum to nucleus signaling 2 |
| rno-novel-106-mature | down | ENSRNOT00000020347 | ENSRNOG00000015109 | Ubxn8 | UBX domain protein 8 |
| rno-miR-351-3p | down | ENSRNOT00000080759 | ENSRNOG00000057855 | F5 | coagulation factor V |
| rno-miR-351-3p | down | ENSRNOT00000026844 | ENSRNOG00000019758 | Ipo13 | importin 13 |
| rno-novel-53-mature | down | ENSRNOT00000013244 | ENSRNOG00000009715 | Me1 | malic enzyme 1 |
| rno-miR-150-5p | down | ENSRNOT00000049973 | ENSRNOG00000031824 | Slc44a2 | solute carrier family 44 member 2 |
| rno-novel-106-mature | down | ENSRNOT00000085547 | ENSRNOG00000000463 | Col11a2 | collagen type XI alpha 2 chain |
| rno-miR-150-3p | down | ENSRNOT00000089359 | ENSRNOG00000053502 | Arhgef17 | Rho guanine nucleotide exchange factor (GEF) 17 |
| rno-miR-330-3p | down | ENSRNOT00000034267 | ENSRNOG00000022164 | Zscan2 | zinc finger and SCAN domain containing 2 |
| rno-miR-150-3p | down | ENSRNOT00000025585 | ENSRNOG00000018905 | Armc4 | armadillo repeat containing 4 |
| rno-novel-53-mature | down | ENSRNOT00000072618 | ENSRNOG00000047741 | E2f2 | E2F transcription factor 2 |
| rno-novel-106-mature | down | ENSRNOT00000077039 | ENSRNOG00000033883 | Stard8 | StAR-related lipid transfer domain containing 8 |
| rno-miR-449c-5p | down | ENSRNOT00000018431 | ENSRNOG00000013620 | Cdk13 | cyclin-dependent kinase 13 [Source:RGD Symbol;Acc:1311226] |
| rno-miR-150-3p | down | ENSRNOT00000089005 | ENSRNOG00000033389 | Susd2 | sushi domain containing 2 |
| rno-novel-53-mature | down | ENSRNOT00000009634 | ENSRNOG00000007081 | Xdh | xanthine dehydrogenase |
| rno-miR-150-3p | down | ENSRNOT00000023163 | ENSRNOG00000017154 | Atp11a | ATPase phospholipid transporting 11A |
| rno-miR-150-3p | down | ENSRNOT00000024412 | ENSRNOG00000018012 | Tulp4 | tubby like protein 4 |
| rno-miR-351-3p | down | ENSRNOT00000085853 | ENSRNOG00000039214 | RGD1305455 | similar to hypothetical protein FLJ10925 |
| rno-novel-106-mature | down | ENSRNOT00000037405 | ENSRNOG00000027098 | Sez6l2 | seizure related 6 homolog like 2 |
| rno-miR-150-5p | down | ENSRNOT00000087934 | ENSRNOG00000059903 | Thbs3 | thrombospondin 3 |
| rno-miR-150-3p | down | ENSRNOT00000066773 | ENSRNOG00000033389 | Susd2 | sushi domain containing 2 |
| rno-miR-351-5p | down | ENSRNOT00000027032 | ENSRNOG00000019935 | Armc5 | armadillo repeat containing 5 |
| rno-miR-201-3p | down | ENSRNOT00000060818 | ENSRNOG00000039656 | Abca17 | ATP-binding cassette, subfamily A (ABC1), member 17 |
| rno-novel-106-mature | down | ENSRNOT00000027897 | ENSRNOG00000020525 | Col5a3 | collagen type V alpha 3 chain |
| rno-novel-34-mature | down | ENSRNOT00000054937 | ENSRNOG00000020457 | Tacc2 | transforming, acidic coiled-coil containing protein 2 |
| rno-novel-53-mature | down | ENSRNOT00000029458 | ENSRNOG00000028302 | LOC103694876 | SWI/SNF-related matrix-associated actin-dependent regulator of chromatin subfamily B member 1 |
| rno-novel-106-mature | down | ENSRNOT00000016333 | ENSRNOG00000012163 | Plekhm2 | pleckstrin homology and RUN domain containing M2 |
| rno-miR-351-3p | down | ENSRNOT00000045212 | ENSRNOG00000020873 | Nphs1 | NPHS1 nephrin |
| rno-novel-106-mature | down | ENSRNOT00000045533 | ENSRNOG00000000463 | Col11a2 | collagen type XI alpha 2 chain |
| rno-miR-351-5p | down | ENSRNOT00000020002 | ENSRNOG00000014610 | Anpep | alanyl aminopeptidase, membrane |
| rno-miR-150-3p | down | ENSRNOT00000070961 | ENSRNOG00000048430 | Myo18b | myosin XVIIIb |
| rno-novel-34-mature | down | ENSRNOT00000080945 | ENSRNOG00000061230 | L1cam | L1 cell adhesion molecule |
| rno-miR-351-5p | down | ENSRNOT00000024933 | ENSRNOG00000018262 | Ampd3 | adenosine monophosphate deaminase 3 |
| rno-miR-351-5p | down | ENSRNOT00000017636 | ENSRNOG00000013017 | Arnt2 | aryl hydrocarbon receptor nuclear translocator 2 |
| rno-miR-503-5p | down | ENSRNOT00000013570 | ENSRNOG00000009977 | Thrap3 | thyroid hormone receptor associated protein 3 |
| rno-novel-34-mature | down | ENSRNOT00000077908 | ENSRNOG00000020457 | Tacc2 | transforming, acidic coiled-coil containing protein 2 |
| rno-novel-106-mature | down | ENSRNOT00000064776 | ENSRNOG00000027057 | Gga3 | golgi associated, gamma adaptin ear containing, ARF binding protein 3 |
| rno-novel-106-mature | down | ENSRNOT00000021999 | ENSRNOG00000016446 | Gpr45 | G protein-coupled receptor 45 |
| rno-novel-106-mature | down | ENSRNOT00000079893 | ENSRNOG00000053240 | Soga1 | suppressor of glucose, autophagy associated 1 |
| rno-miR-503-5p | down | ENSRNOT00000036187 | ENSRNOG00000026115 | Zfp646 | zinc finger protein 646 |
| rno-miR-150-3p | down | ENSRNOT00000011513 | ENSRNOG00000008409 | Myo1f | myosin IF |
| rno-miR-500-5p | down | ENSRNOT00000061693 | ENSRNOG00000040110 | Foxn2 | forkhead box N2 |
| rno-novel-106-mature | down | ENSRNOT00000078644 | ENSRNOG00000059961 | Rapgef3 | Rap guanine nucleotide exchange factor 3 |
| rno-miR-547-5p | down | ENSRNOT00000076451 | ENSRNOG00000015921 | Esco2 | establishment of sister chromatid cohesion N-acetyltransferase 2 |
| rno-miR-330-3p | down | ENSRNOT00000047482 | ENSRNOG00000031816 | Nckipsd | NCK interacting protein with SH3 domain |
| rno-novel-53-mature | down | ENSRNOT00000001957 | ENSRNOG00000001439 | Srrm3 | serine/arginine repetitive matrix 3 |
| rno-miR-150-3p | down | ENSRNOT00000086385 | ENSRNOG00000013456 | Ighmbp2 | immunoglobulin mu binding protein 2 |
| rno-miR-351-3p | down | ENSRNOT00000089497 | ENSRNOG00000052113 | Ppp1r9b | protein phosphatase 1, regulatory subunit 9B |
| rno-novel-53-mature | down | ENSRNOT00000084390 | ENSRNOG00000061230 | L1cam | L1 cell adhesion molecule |
| rno-novel-34-mature | down | ENSRNOT00000001347 | ENSRNOG00000001010 | Tecpr1 | tectonin beta-propeller repeat containing 1 |
| rno-miR-150-5p | down | ENSRNOT00000021689 | ENSRNOG00000015845 | Fam129b | family with sequence similarity 129, member B |
| rno-miR-547-5p | down | ENSRNOT00000009803 | ENSRNOG00000025120 | Gli1 | GLI family zinc finger 1 |
| rno-miR-150-5p | down | ENSRNOT00000068214 | ENSRNOG00000012950 | Efr3b | EFR3 homolog B |
| rno-novel-106-mature | down | ENSRNOT00000077313 | ENSRNOG00000052687 | Megf8 | multiple EGF-like-domains 8 |
| rno-miR-503-3p | down | ENSRNOT00000021554 | ENSRNOG00000015977 | Zfp609 | zinc finger protein 609 |
| rno-miR-150-5p | down | ENSRNOT00000091065 | ENSRNOG00000017403 | Apobr | apolipoprotein B receptor |
| rno-miR-150-5p | down | ENSRNOT00000065246 | ENSRNOG00000007062 | Rin3 | Ras and Rab interactor 3 |
| rno-miR-150-3p | down | ENSRNOT00000018487 | ENSRNOG00000013456 | Ighmbp2 | immunoglobulin mu binding protein 2 |
| rno-novel-53-mature | down | ENSRNOT00000080963 | ENSRNOG00000017671 | Rasa3 | RAS p21 protein activator 3 |
| rno-novel-106-mature | down | ENSRNOT00000004365 | ENSRNOG00000003243 | Wdr81 | WD repeat domain 81 |
| rno-miR-351-5p | down | ENSRNOT00000092125 | ENSRNOG00000014610 | Anpep | alanyl aminopeptidase, membrane |
| rno-novel-34-mature | down | ENSRNOT00000010516 | ENSRNOG00000008005 | Akna | AT-hook transcription factor |
| rno-novel-34-mature | down | ENSRNOT00000011662 | ENSRNOG00000008614 | Zfyve1 | zinc finger FYVE-type containing 1 |
| rno-novel-106-mature | down | ENSRNOT00000068413 | ENSRNOG00000023148 | Col11a1 | collagen type XI alpha 1 chain |
| rno-novel-106-mature | down | ENSRNOT00000064902 | ENSRNOG00000013934 | St5 | suppression of tumorigenicity 5 |
| rno-miR-503-5p | down | ENSRNOT00000051893 | ENSRNOG00000032700 | Zbtb34 | zinc finger and BTB domain containing 34 |
| rno-novel-34-mature | down | ENSRNOT00000022282 | ENSRNOG00000016553 | Vwa5b1 | von Willebrand factor A domain containing 5B1 |
| rno-novel-53-mature | down | ENSRNOT00000019579 | ENSRNOG00000014597 | Irs1 | insulin receptor substrate 1 |
| rno-miR-351-3p | down | ENSRNOT00000079872 | ENSRNOG00000057855 | F5 | coagulation factor V |
| rno-miR-351-3p | down | ENSRNOT00000086830 | ENSRNOG00000061348 | Fam53b | family with sequence similarity 53, member B |
| rno-novel-79-star | down | ENSRNOT00000001807 | ENSRNOG00000001337 | Setd1b | SET domain containing 1B |
| rno-novel-106-mature | down | ENSRNOT00000051846 | ENSRNOG00000007090 | Cacna1c | calcium voltage-gated channel subunit alpha1 C |
| rno-novel-34-mature | down | ENSRNOT00000082113 | ENSRNOG00000030714 | Bsn | bassoon (presynaptic cytomatrix protein) |
| rno-novel-34-mature | down | ENSRNOT00000085600 | ENSRNOG00000059202 | Mia3 | MIA family member 3, ER export factor |
| rno-miR-150-3p | down | ENSRNOT00000085600 | ENSRNOG00000059202 | Mia3 | MIA family member 3, ER export factor |
| rno-miR-503-5p | down | ENSRNOT00000021762 | ENSRNOG00000016229 | Gltscr1l | GLTSCR1-like |
| rno-novel-106-mature | down | ENSRNOT00000000713 | ENSRNOG00000000583 | Cdk19 | cyclin-dependent kinase 19 |
| rno-novel-106-mature | down | ENSRNOT00000052017 | ENSRNOG00000007090 | Cacna1c | calcium voltage-gated channel subunit alpha1 C |
| rno-novel-106-mature | down | ENSRNOT00000010688 | ENSRNOG00000008113 | RGD1561149 | similar to mKIAA1522 protein |
| rno-miR-503-5p | down | ENSRNOT00000091809 | ENSRNOG00000016229 | Gltscr1l | GLTSCR1-like |
| rno-novel-34-mature | down | ENSRNOT00000084390 | ENSRNOG00000061230 | L1cam | L1 cell adhesion molecule |
| rno-novel-106-mature | down | ENSRNOT00000001891 | ENSRNOG00000001397 | Rbm19 | RNA binding motif protein 19 |
| rno-miR-351-3p | down | ENSRNOT00000073321 | ENSRNOG00000045636 | Fasn | fatty acid synthase |
| rno-miR-330-3p | down | ENSRNOT00000091473 | ENSRNOG00000009264 | Erc1 | ELKS/RAB6-interacting/CAST family member 1 |
| rno-novel-106-mature | down | ENSRNOT00000010333 | ENSRNOG00000007657 | Col27a1 | collagen type XXVII alpha 1 chain |
| rno-miR-17-1-3p | down | ENSRNOT00000081007 | ENSRNOG00000008924 | Arhgef12 | Rho guanine nucleotide exchange factor 12 |
| rno-miR-150-3p | down | ENSRNOT00000004477 | ENSRNOG00000003280 | Grin2c | glutamate ionotropic receptor NMDA type subunit 2C |
| rno-novel-106-mature | down | ENSRNOT00000041571 | ENSRNOG00000007090 | Cacna1c | calcium voltage-gated channel subunit alpha1 C |
| rno-miR-150-3p | down | ENSRNOT00000023149 | ENSRNOG00000017210 | Slc22a23 | solute carrier family 22, member 23 |
| rno-miR-351-5p | down | ENSRNOT00000080907 | ENSRNOG00000056228 | Atp10a | ATPase phospholipid transporting 10A (putative) |
| rno-novel-106-mature | down | ENSRNOT00000079682 | ENSRNOG00000016874 | Zfp521 | zinc finger protein 521 |
| rno-novel-106-mature | down | ENSRNOT00000018634 | ENSRNOG00000013886 | Fyb | FYN binding protein |
| rno-miR-342-5p | down | ENSRNOT00000001466 | ENSRNOG00000001104 | Foxk1 | forkhead box K1 |
| rno-novel-53-mature | down | ENSRNOT00000078175 | ENSRNOG00000010048 | Dctn1 | dynactin subunit 1 |
| rno-miR-150-3p | down | ENSRNOT00000092846 | ENSRNOG00000011300 | Lama3 | laminin subunit alpha 3 |
| rno-novel-53-mature | down | ENSRNOT00000068102 | ENSRNOG00000010048 | Dctn1 | dynactin subunit 1 |
| rno-miR-351-5p | down | ENSRNOT00000065950 | ENSRNOG00000015540 | Ppp6r3 | protein phosphatase 6, regulatory subunit 3 |
| rno-miR-150-3p | down | ENSRNOT00000083366 | ENSRNOG00000002369 | Rgs8 | regulator of G-protein signaling 8 |
| rno-miR-17-1-3p | down | ENSRNOT00000025325 | ENSRNOG00000018387 | Wdr7 | WD repeat domain 7 |
| rno-novel-106-mature | down | ENSRNOT00000066281 | ENSRNOG00000028090 | Arhgef18 | Rho/Rac guanine nucleotide exchange factor 18 |
| rno-miR-342-5p | down | ENSRNOT00000014211 | ENSRNOG00000010409 | Nol6 | nucleolar protein 6 |
| rno-miR-503-5p | down | ENSRNOT00000089644 | ENSRNOG00000011334 | Tmem63c | transmembrane protein 63c |
| rno-novel-106-mature | down | ENSRNOT00000080088 | ENSRNOG00000061731 | Plxnb3 | plexin B3 |
| rno-miR-150-3p | down | ENSRNOT00000055137 | ENSRNOG00000006548 | Mrc2 | mannose receptor, C type 2 |
| rno-novel-34-mature | down | ENSRNOT00000001466 | ENSRNOG00000001104 | Foxk1 | forkhead box K1 |
| rno-novel-106-mature | down | ENSRNOT00000002180 | ENSRNOG00000001602 | Ltn1 | listerin E3 ubiquitin protein ligase 1 |
| rno-miR-17-1-3p | down | ENSRNOT00000046744 | ENSRNOG00000014844 | Kif21a | kinesin family member 21A |
| rno-miR-17-1-3p | down | ENSRNOT00000059868 | ENSRNOG00000008924 | Arhgef12 | Rho guanine nucleotide exchange factor 12 |
| rno-miR-330-3p | down | ENSRNOT00000023149 | ENSRNOG00000017210 | Slc22a23 | solute carrier family 22, member 23 |
| rno-miR-17-1-3p | down | ENSRNOT00000045553 | ENSRNOG00000014844 | Kif21a | kinesin family member 21A |
| rno-miR-449c-5p | down | ENSRNOT00000005059 | ENSRNOG00000003723 | Wdr26 | WD repeat domain 26 |
| rno-miR-17-1-3p | down | ENSRNOT00000066528 | ENSRNOG00000014844 | Kif21a | kinesin family member 21A |
| rno-miR-330-3p | down | ENSRNOT00000012273 | ENSRNOG00000008996 | Dpysl5 | dihydropyrimidinase-like 5 |
| rno-miR-322-5p | down | ENSRNOT00000044413 | ENSRNOG00000034134 | Cpm | carboxypeptidase M |
| rno-novel-106-mature | down | ENSRNOT00000089374 | ENSRNOG00000049221 | Kdm4b | lysine demethylase 4B |
| rno-miR-150-3p | down | ENSRNOT00000071442 | ENSRNOG00000020893 | Snx27 | sorting nexin family member 27 |
| rno-miR-351-5p | down | ENSRNOT00000081667 | ENSRNOG00000051291 | Dnhd1 | dynein heavy chain domain 1 |
| rno-miR-17-1-3p | down | ENSRNOT00000038946 | ENSRNOG00000014844 | Kif21a | kinesin family member 21A |
| rno-miR-17-1-3p | down | ENSRNOT00000044092 | ENSRNOG00000014844 | Kif21a | kinesin family member 21A |
| rno-novel-106-mature | down | ENSRNOT00000078909 | ENSRNOG00000051490 | Plcg1 | phospholipase C, gamma 1 |
| rno-miR-150-5p | down | ENSRNOT00000018853 | ENSRNOG00000014034 | Olfml2a | olfactomedin-like 2A |
| rno-novel-53-mature | down | ENSRNOT00000084220 | ENSRNOG00000053889 | Celsr3 | cadherin, EGF LAG seven-pass G-type receptor 3 |
| rno-miR-150-3p | down | ENSRNOT00000076776 | ENSRNOG00000028627 | Hmcn1 | hemicentin 1 |
| rno-novel-106-mature | down | ENSRNOT00000041140 | ENSRNOG00000034013 | Acaca | acetyl-CoA carboxylase alpha |
| rno-miR-351-5p | down | ENSRNOT00000076783 | ENSRNOG00000051291 | Dnhd1 | dynein heavy chain domain 1 |
| rno-novel-34-mature | down | ENSRNOT00000024732 | ENSRNOG00000011268 | Chd5 | chromodomain helicase DNA binding protein 5 |
| rno-novel-106-mature | down | ENSRNOT00000091637 | ENSRNOG00000013515 | Ptpru | protein tyrosine phosphatase, receptor type, U |
| rno-miR-330-3p | down | ENSRNOT00000006425 | ENSRNOG00000004826 | Sos2 | SOS Ras/Rho guanine nucleotide exchange factor 2 |
| rno-novel-106-mature | down | ENSRNOT00000081037 | ENSRNOG00000061262 | Huwe1 | HECT, UBA and WWE domain containing 1, E3 ubiquitin protein ligase |
| rno-novel-106-mature | down | ENSRNOT00000072955 | ENSRNOG00000008392 | Sbf1 | SET binding factor 1 |
| rno-novel-34-mature | down | ENSRNOT00000015181 | ENSRNOG00000011151 | Tenm4 | teneurin transmembrane protein 4 |
| rno-miR-150-5p | down | ENSRNOT00000055251 | ENSRNOG00000019340 | Cep250 | centrosomal protein 250 |
| rno-miR-150-3p | down | ENSRNOT00000065145 | ENSRNOG00000024711 | Sdk2 | sidekick cell adhesion molecule 2 |
| rno-miR-17-1-3p | down | ENSRNOT00000009359 | ENSRNOG00000007106 | Sos1 | SOS Ras/Rac guanine nucleotide exchange factor 1 |
| rno-miR-330-3p | down | ENSRNOT00000018023 | ENSRNOG00000013236 | Samd8 | sterile alpha motif domain containing 8 |
| rno-novel-106-mature | down | ENSRNOT00000031005 | ENSRNOG00000025053 | Lrp1 | LDL receptor related protein 1 |
| rno-novel-106-mature | down | ENSRNOT00000088138 | ENSRNOG00000034013 | Acaca | acetyl-CoA carboxylase alpha |
| rno-novel-6-mature | down | ENSRNOT00000008888 | ENSRNOG00000005390 | Nup210 | nucleoporin 210 |
| rno-novel-79-star | down | ENSRNOT00000080088 | ENSRNOG00000061731 | Plxnb3 | plexin B3 |
| rno-miR-351-3p | down | ENSRNOT00000009763 | ENSRNOG00000007027 | Hgf | hepatocyte growth factor |
| rno-miR-150-3p | down | ENSRNOT00000028389 | ENSRNOG00000020893 | Snx27 | sorting nexin family member 27 |
| rno-miR-150-3p | down | ENSRNOT00000030971 | ENSRNOG00000028627 | Hmcn1 | hemicentin 1 |
| rno-novel-106-mature | down | ENSRNOT00000077015 | ENSRNOG00000045913 | Prdm16 | PR/SET domain 16 |
| rno-novel-106-mature | down | ENSRNOT00000047854 | ENSRNOG00000020205 | Agrn | agrin |
| rno-novel-34-mature | down | ENSRNOT00000067892 | ENSRNOG00000011268 | Chd5 | chromodomain helicase DNA binding protein 5 |
| rno-novel-106-mature | down | ENSRNOT00000045678 | ENSRNOG00000020205 | Agrn | agrin |
| rno-novel-106-mature | down | ENSRNOT00000007079 | ENSRNOG00000005330 | Crebbp | CREB binding protein |
| rno-miR-330-3p | down | ENSRNOT00000046381 | ENSRNOG00000025155 | Lmtk2 | lemur tyrosine kinase 2 |
| rno-miR-150-3p | down | ENSRNOT00000091129 | ENSRNOG00000024711 | Sdk2 | sidekick cell adhesion molecule 2 |
| rno-miR-150-5p | down | ENSRNOT00000018149 | ENSRNOG00000013186 | G3bp1 | G3BP stress granule assembly factor 1 |
| rno-novel-34-mature | down | ENSRNOT00000042865 | ENSRNOG00000031643 | Dchs1 | dachsous cadherin-related 1 |
| rno-miR-330-3p | down | ENSRNOT00000013528 | ENSRNOG00000009971 | Plekha8 | pleckstrin homology domain containing A8 |
| rno-novel-106-mature | down | ENSRNOT00000032430 | ENSRNOG00000028362 | Unc80 | unc-80 homolog, NALCN activator |
| rno-novel-106-mature | down | ENSRNOT00000066546 | ENSRNOG00000009470 | Flnb | filamin B |
| rno-novel-106-mature | down | ENSRNOT00000039235 | ENSRNOG00000028659 | Szt2 | seizure threshold 2 homolog (mouse) |
| rno-novel-53-mature | down | ENSRNOT00000068658 | ENSRNOG00000042519 | Peak1 | pseudopodium-enriched atypical kinase 1 |
| rno-miR-150-3p | down | ENSRNOT00000014004 | ENSRNOG00000010488 | Zmiz1 | zinc finger, MIZ-type containing 1 |
| rno-miR-449c-5p | down | ENSRNOT00000004784 | ENSRNOG00000003578 | Fem1c | fem-1 homolog C |
| rno-miR-449c-5p | down | ENSRNOT00000026212 | ENSRNOG00000019322 | Notch1 | notch 1 |
| rno-novel-106-mature | down | ENSRNOT00000088264 | ENSRNOG00000009470 | Flnb | filamin B |
| rno-novel-34-mature | down | ENSRNOT00000042528 | ENSRNOG00000030714 | Bsn | bassoon (presynaptic cytomatrix protein) |
| rno-novel-106-mature | down | ENSRNOT00000088919 | ENSRNOG00000033101 | Myo18a | myosin XVIIIa |
| rno-novel-106-mature | down | ENSRNOT00000044452 | ENSRNOG00000012207 | Dst | dystonin |
| rno-novel-106-mature | down | ENSRNOT00000079473 | ENSRNOG00000020557 | Ryr1 | ryanodine receptor 1 |
| rno-novel-106-mature | down | ENSRNOT00000027893 | ENSRNOG00000020557 | Ryr1 | ryanodine receptor 1 |
| rno-miR-330-3p | down | ENSRNOT00000077943 | ENSRNOG00000034025 | Ptprj | protein tyrosine phosphatase, receptor type, J |
| rno-novel-106-mature | down | ENSRNOT00000091139 | ENSRNOG00000056786 | Piezo1 | piezo-type mechanosensitive ion channel component 1 |
| rno-miR-503-5p | down | ENSRNOT00000046381 | ENSRNOG00000025155 | Lmtk2 | lemur tyrosine kinase 2 |
| rno-novel-106-mature | down | ENSRNOT00000090809 | ENSRNOG00000052688 | Dnah2 | dynein, axonemal, heavy chain 2 |
| rno-miR-17-1-3p | down | ENSRNOT00000031389 | ENSRNOG00000027906 | Ankrd11 | ankyrin repeat domain 11 |
| rno-miR-449c-5p | down | ENSRNOT00000006562 | ENSRNOG00000004841 | Akap6 | A-kinase anchoring protein 6 |
| rno-novel-106-mature | down | ENSRNOT00000086550 | ENSRNOG00000023781 | Plec | plectin |
| rno-novel-106-mature | down | ENSRNOT00000091285 | ENSRNOG00000023781 | Plec | plectin |
| rno-novel-106-mature | down | ENSRNOT00000088945 | ENSRNOG00000023781 | Plec | plectin |
| rno-novel-106-mature | down | ENSRNOT00000091840 | ENSRNOG00000023781 | Plec | plectin |
| rno-novel-106-mature | down | ENSRNOT00000042642 | ENSRNOG00000023781 | Plec | plectin |
| rno-novel-106-mature | down | ENSRNOT00000081021 | ENSRNOG00000023781 | Plec | plectin |
| rno-novel-106-mature | down | ENSRNOT00000082271 | ENSRNOG00000023781 | Plec | plectin |
| rno-novel-106-mature | down | ENSRNOT00000006311 | ENSRNOG00000023781 | Plec | plectin |
| rno-novel-106-mature | down | ENSRNOT00000040762 | ENSRNOG00000023781 | Plec | plectin |
| rno-miR-150-3p | down | ENSRNOT00000035247 | ENSRNOG00000025028 | Prkdc | protein kinase, DNA activated, catalytic polypeptide |
| rno-novel-106-mature | down | ENSRNOT00000083429 | ENSRNOG00000023781 | Plec | plectin |
| rno-novel-53-mature | down | ENSRNOT00000086550 | ENSRNOG00000023781 | Plec | plectin |
| rno-novel-53-mature | down | ENSRNOT00000091285 | ENSRNOG00000023781 | Plec | plectin |
| rno-novel-53-mature | down | ENSRNOT00000088945 | ENSRNOG00000023781 | Plec | plectin |
| rno-novel-53-mature | down | ENSRNOT00000091840 | ENSRNOG00000023781 | Plec | plectin |
| rno-novel-53-mature | down | ENSRNOT00000042642 | ENSRNOG00000023781 | Plec | plectin |
| rno-novel-53-mature | down | ENSRNOT00000081021 | ENSRNOG00000023781 | Plec | plectin |
| rno-novel-53-mature | down | ENSRNOT00000082271 | ENSRNOG00000023781 | Plec | plectin |
| rno-novel-53-mature | down | ENSRNOT00000006311 | ENSRNOG00000023781 | Plec | plectin |
| rno-novel-53-mature | down | ENSRNOT00000040762 | ENSRNOG00000023781 | Plec | plectin |
| rno-novel-53-mature | down | ENSRNOT00000083429 | ENSRNOG00000023781 | Plec | plectin |
| rno-miR-150-5p | down | ENSRNOT00000085698 | ENSRNOG00000033110 | Svep1 | sushi, von Willebrand factor type A, EGF and pentraxin domain containing 1 |
| rno-novel-34-mature | down | ENSRNOT00000092720 | ENSRNOG00000039832 | Gpr12 | G protein-coupled receptor 12 |
| rno-novel-34-mature | down | ENSRNOT00000078180 | ENSRNOG00000013287 | Sall2 | spalt-like transcription factor 2 |
| rno-novel-106-mature | down | ENSRNOT00000001733 | ENSRNOG00000001288 | Gpr146 | G protein-coupled receptor 146 |
| rno-novel-34-mature | down | ENSRNOT00000017730 | ENSRNOG00000013287 | Sall2 | spalt-like transcription factor 2 |
| rno-miR-351-5p | down | ENSRNOT00000030037 | ENSRNOG00000019555 | Arap1 | ArfGAP with RhoGAP domain, ankyrin repeat and PH domain 1 |
| rno-novel-34-mature | down | ENSRNOT00000010333 | ENSRNOG00000007657 | Col27a1 | collagen type XXVII alpha 1 chain |
| rno-miR-500-5p | down | ENSRNOT00000057461 | ENSRNOG00000023972 | Col4a2 | collagen type IV alpha 2 chain |
| rno-novel-106-mature | down | ENSRNOT00000024037 | ENSRNOG00000017525 | Epha1 | Eph receptor A1 |
