# Supplementary material for: Differential Expression Profile of microRNAs and Tight Junction in the Lung Tissues of Rat With Mitomycin-C-Induced Pulmonary Veno-Occlusive Disease
Source: Front Cardiovasc Med. 2022 Feb 16;9:746888. doi: 10.3389/fcvm.2022.746888 (PMC8889576; doi:10.3389/fcvm.2022.746888)
Supplement: Supplementary file 5 [file Table_5.docx]

**Supplement table 5.** The detail target genes of up-regulated miRNAs with differential expression.

| **miRNA** | **Regulated** | **Transcript** | **Gene** | **Name** | **Description** | |
| --- | --- | --- | --- | --- | --- | --- |
| rno-miR-665 | up | ENSRNOT00000089073 | ENSRNOG00000011599 | Gldc | glycine decarboxylase | |
| rno-miR-665 | up | ENSRNOT00000045318 | ENSRNOG00000049845 | Olr1423 | olfactory receptor 1423 | |
| rno-miR-485-5p | up | ENSRNOT00000085380 | ENSRNOG00000057125 | Ddr1 | discoidin domain receptor tyrosine kinase 1 | |
| rno-miR-370-3p | up | ENSRNOT00000037890 | ENSRNOG00000027408 | Ppid | peptidylprolyl isomerase D | |
| rno-miR-673-3p | up | ENSRNOT00000000092 | ENSRNOG00000000081 | Antxr2 | anthrax toxin receptor 2 | |
| rno-miR-127-3p | up | ENSRNOT00000074645 | ENSRNOG00000047631 | Mettl21b | methyltransferase like 21B | |
| rno-miR-665 | up | ENSRNOT00000077711 | ENSRNOG00000001404 | Agfg2 | ArfGAP with FG repeats 2 | |
| rno-miR-673-3p | up | ENSRNOT00000080900 | ENSRNOG00000038999 | RT1-A1 | RT1 class Ia, locus A1 | |
| rno-miR-673-3p | up | ENSRNOT00000022830 | ENSRNOG00000016915 | Barx1 | BARX homeobox 1 | |
| rno-miR-370-3p | up | ENSRNOT00000026665 | ENSRNOG00000019693 | Clpb | ClpB homolog, mitochondrial AAA ATPase chaperonin | |
| rno-miR-665 | up | ENSRNOT00000078299 | ENSRNOG00000052205 | Fgf23 | fibroblast growth factor 23 | |
| rno-miR-337-5p | up | ENSRNOT00000023613 | ENSRNOG00000017250 | Gmpr | guanosine monophosphate reductase | |
| rno-miR-673-3p | up | ENSRNOT00000014645 | ENSRNOG00000011026 | Irf2bpl | interferon regulatory factor 2 binding protein-like | |
| rno-miR-665 | up | ENSRNOT00000022863 | ENSRNOG00000016890 | Tppp3 | tubulin polymerization-promoting protein family member 3 | |
| rno-miR-127-5p | up | ENSRNOT00000022280 | ENSRNOG00000016516 | Mbp | myelin basic protein | |
| rno-miR-127-5p | up | ENSRNOT00000022303 | ENSRNOG00000016516 | Mbp | myelin basic protein | |
| rno-miR-127-5p | up | ENSRNOT00000058295 | ENSRNOG00000016516 | Mbp | myelin basic protein | |
| rno-miR-127-5p | up | ENSRNOT00000058296 | ENSRNOG00000016516 | Mbp | myelin basic protein | |
| rno-miR-214-3p | up | ENSRNOT00000080170 | ENSRNOG00000059202 | Mia3 | MIA family member 3, ER export factor | |
| rno-miR-370-3p | up | ENSRNOT00000018449 | ENSRNOG00000013673 | Msh3 | mutS homolog 3 | |
| rno-miR-370-3p | up | ENSRNOT00000044020 | ENSRNOG00000012785 | Armc10 | armadillo repeat containing 10 | |
| rno-miR-673-3p | up | ENSRNOT00000075206 | ENSRNOG00000047517 | Oxld1 | oxidoreductase-like domain containing 1 | |
| rno-miR-214-3p | up | ENSRNOT00000028892 | ENSRNOG00000021269 | Chgb | chromogranin B | |
| rno-miR-140-3p | up | ENSRNOT00000028898 | ENSRNOG00000021272 | Mcm8 | minichromosome maintenance 8 homologous recombination repair factor | |
| rno-miR-673-3p | up | ENSRNOT00000016871 | ENSRNOG00000012156 | Ostf1 | osteoclast stimulating factor 1 | |
| rno-miR-673-3p | up | ENSRNOT00000015564 | ENSRNOG00000011589 | Camk2d | calcium/calmodulin-dependent protein kinase II delta | |
| rno-miR-673-3p | up | ENSRNOT00000078972 | ENSRNOG00000038999 | RT1-A1 | RT1 class Ia, locus A1 | |
| rno-miR-673-3p | up | ENSRNOT00000014934 | ENSRNOG00000011078 | Srm | spermidine synthase | |
| rno-miR-485-5p | up | ENSRNOT00000066952 | ENSRNOG00000001294 | Ift81 | intraflagellar transport 81 | |
| rno-miR-665 | up | ENSRNOT00000087888 | ENSRNOG00000013577 | Kdelr3 | KDEL endoplasmic reticulum protein retention receptor 3 | |
| rno-miR-136-3p | up | ENSRNOT00000072399 | ENSRNOG00000048751 | Rtl1 | retrotransposon-like 1 | |
| rno-miR-370-3p | up | ENSRNOT00000044726 | ENSRNOG00000004290 | Grb10 | growth factor receptor bound protein 10 | |
| rno-miR-370-3p | up | ENSRNOT00000016000 | ENSRNOG00000010967 | Cdc37l1 | cell division cycle 37-like 1 | |
| rno-miR-673-3p | up | ENSRNOT00000002188 | ENSRNOG00000001608 | Nxpe3 | neurexophilin and PC-esterase domain family, member 3 | |
| rno-miR-665 | up | ENSRNOT00000071885 | ENSRNOG00000049033 | Racgap1 | Rac GTPase-activating protein 1 | |
| rno-miR-127-3p | up | ENSRNOT00000041238 | ENSRNOG00000020299 | Klc2 | kinesin light chain 2 | |
| rno-miR-665 | up | ENSRNOT00000067584 | ENSRNOG00000021988 | Olfr225 | olfactory receptor 225 | |
| rno-miR-370-3p | up | ENSRNOT00000016321 | ENSRNOG00000012001 | Vps37a | VPS37A, ESCRT-I subunit | |
| rno-miR-127-3p | up | ENSRNOT00000010101 | ENSRNOG00000007089 | Lgmn | legumain | |
| rno-miR-341 | up | ENSRNOT00000060143 | ENSRNOG00000017093 | Pxdc1 | PX domain containing 1 | |
| rno-miR-665 | up | ENSRNOT00000089309 | ENSRNOG00000058805 | Aida | axin interactor, dorsalization associated | |
| rno-miR-665 | up | ENSRNOT00000047158 | ENSRNOG00000014152 | Kcnip3 | potassium voltage-gated channel interacting protein 3 | |
| rno-miR-370-3p | up | ENSRNOT00000007773 | ENSRNOG00000005929 | Them6 | thioesterase superfamily member 6 | |
| rno-miR-665 | up | ENSRNOT00000067327 | ENSRNOG00000027569 | Trappc9 | trafficking protein particle complex 9 | |
| rno-miR-665 | up | ENSRNOT00000038172 | ENSRNOG00000027569 | Trappc9 | trafficking protein particle complex 9 | |
| rno-miR-370-3p | up | ENSRNOT00000075513 | ENSRNOG00000047455 | Cdhr3 | cadherin-related family member 3 | |
| rno-miR-665 | up | ENSRNOT00000002299 | ENSRNOG00000001692 | Chaf1b | chromatin assembly factor 1 subunit B | |
| rno-miR-370-3p | up | ENSRNOT00000089891 | ENSRNOG00000004826 | Sos2 | SOS Ras/Rho guanine nucleotide exchange factor 2 | |
| rno-miR-485-5p | up | ENSRNOT00000064557 | ENSRNOG00000017663 | Dolpp1 | dolichyldiphosphatase 1 | |
| rno-miR-673-3p | up | ENSRNOT00000077462 | ENSRNOG00000021097 | Hpn | hepsin | |
| rno-miR-673-3p | up | ENSRNOT00000028644 | ENSRNOG00000021097 | Hpn | hepsin | |
| rno-miR-673-3p | up | ENSRNOT00000000673 | ENSRNOG00000000557 | Ppa1 | pyrophosphatase (inorganic) 1 | |
| rno-miR-665 | up | ENSRNOT00000000499 | ENSRNOG00000000436 | Egfl8 | EGF-like-domain, multiple 8 [Source:RGD Symbol;Acc:1549706] | |
| rno-miR-370-3p | up | ENSRNOT00000060767 | ENSRNOG00000019549 | Akap12 | A-kinase anchoring protein 12 | |
| rno-miR-212-5p | up | ENSRNOT00000080271 | ENSRNOG00000057713 | Cav2 | caveolin 2 | |
| rno-miR-370-3p | up | ENSRNOT00000088558 | ENSRNOG00000015473 | Phactr2 | phosphatase and actin regulator 2 | |
| rno-miR-433-3p | up | ENSRNOT00000024375 | ENSRNOG00000017887 | Mutyh | mutY DNA glycosylase | |
| rno-miR-673-3p | up | ENSRNOT00000025913 | ENSRNOG00000043416 | Bcl3 | B-cell CLL/lymphoma 3 | |
| rno-miR-212-5p | up | ENSRNOT00000008969 | ENSRNOG00000006766 | Laptm4b | lysosomal protein transmembrane 4 beta | |
| rno-miR-665 | up | ENSRNOT00000028893 | ENSRNOG00000021270 | Trmt6 | tRNA methyltransferase 6 | |
| rno-miR-665 | up | ENSRNOT00000001625 | ENSRNOG00000001214 | Pfkl | phosphofructokinase, liver type | |
| rno-miR-665 | up | ENSRNOT00000082899 | ENSRNOG00000059862 | Avpr2 | arginine vasopressin receptor 2 | |
| rno-miR-665 | up | ENSRNOT00000028494 | ENSRNOG00000020995 | Fut1 | fucosyltransferase 1 | |
| rno-miR-370-3p | up | ENSRNOT00000039000 | ENSRNOG00000022939 | Gpkow | G patch domain and KOW motifs | |
| rno-miR-431 | up | ENSRNOT00000033687 | ENSRNOG00000027722 | H1fx | H1 histone family, member X | |
| rno-miR-212-5p | up | ENSRNOT00000013927 | ENSRNOG00000010480 | Vstm5 | V-set and transmembrane domain containing 5 | |
| rno-miR-214-3p | up | ENSRNOT00000064064 | ENSRNOG00000030726 | Asgr2 | asialoglycoprotein receptor 2 | |
| rno-miR-370-3p | up | ENSRNOT00000023404 | ENSRNOG00000017326 | Ctbp2 | C-terminal binding protein 2 | |
| rno-miR-370-3p | up | ENSRNOT00000078222 | ENSRNOG00000019012 | D2hgdh | D-2-hydroxyglutarate dehydrogenase | |
| rno-miR-214-3p | up | ENSRNOT00000083703 | ENSRNOG00000058157 | Ldlrad3 | low density lipoprotein receptor class A domain containing 3 | |
| rno-miR-127-5p | up | ENSRNOT00000010723 | ENSRNOG00000007881 | Mtmr14 | myotubularin related protein 14 | |
| rno-miR-665 | up | ENSRNOT00000060977 | ENSRNOG00000014316 | Rapgef1 | Rap guanine nucleotide exchange factor 1 | |
| rno-miR-370-3p | up | ENSRNOT00000002169 | ENSRNOG00000001592 | Cct8 | chaperonin containing TCP1 subunit 8 | |
| rno-miR-370-3p | up | ENSRNOT00000005050 | ENSRNOG00000003777 | Chrne | cholinergic receptor nicotinic epsilon subunit | |
| rno-miR-370-3p | up | ENSRNOT00000008660 | ENSRNOG00000006591 | Fam163b | family with sequence similarity 163, member B | |
| rno-miR-665 | up | ENSRNOT00000040323 | ENSRNOG00000032086 | AABR07066753.1 |  | |
| rno-miR-665 | up | ENSRNOT00000066779 | ENSRNOG00000042468 | LOC680920 | similar to natural cytotoxicity triggering receptor 2 | |
| rno-miR-665 | up | ENSRNOT00000081157 | ENSRNOG00000058580 | Paip1 | poly(A) binding protein interacting protein 1 | |
| rno-miR-665 | up | ENSRNOT00000075558 | ENSRNOG00000048060 | RGD1563072 | similar to hypothetical protein FLJ38984 | |
| rno-miR-665 | up | ENSRNOT00000016995 | ENSRNOG00000012654 | Vps36 | vacuolar protein sorting 36 | |
| rno-miR-127-3p | up | ENSRNOT00000087451 | ENSRNOG00000007304 | Herc3 | HECT and RLD domain containing E3 ubiquitin protein ligase 3 | |
| rno-miR-665 | up | ENSRNOT00000046956 | ENSRNOG00000034087 | Krt42 | keratin 42 | |
| rno-miR-665 | up | ENSRNOT00000004359 | ENSRNOG00000003244 | Ltc4s | leukotriene C4 synthase | |
| rno-miR-370-3p | up | ENSRNOT00000083196 | ENSRNOG00000058142 | 1700069L16Rik | RIKEN cDNA 1700069L16 gene | |
| rno-miR-665 | up | ENSRNOT00000005575 | ENSRNOG00000004177 | Myo1a | myosin IA | |
| rno-miR-127-5p | up | ENSRNOT00000073565 | ENSRNOG00000049778 | LOC687119 | similar to olfactory receptor 513 | |
| rno-miR-6331 | up | ENSRNOT00000072019 | ENSRNOG00000046144 | Hist1h3a | histone cluster 1, H3a | |
| rno-miR-673-3p | up | ENSRNOT00000017968 | ENSRNOG00000013177 | LOC100912399 | mitogen-activated protein kinase kinase kinase 1-like | |
| rno-miR-127-3p | up | ENSRNOT00000020524 | ENSRNOG00000015283 | Nt5c1a | 5'-nucleotidase, cytosolic IA | |
| rno-miR-370-3p | up | ENSRNOT00000005770 | ENSRNOG00000004131 | Pkn1 | protein kinase N1 | |
| rno-miR-127-5p | up | ENSRNOT00000075227 | ENSRNOG00000053834 | LOC100909796 | olfactory receptor 8K5-like | |
| rno-miR-127-5p | up | ENSRNOT00000072178 | ENSRNOG00000049125 | LOC684539 | similar to olfactory receptor Olr490 | |
| rno-miR-665 | up | ENSRNOT00000013994 | ENSRNOG00000010539 | Mcat | malonyl-CoA-acyl carrier protein transacylase | |
| rno-miR-127-5p | up | ENSRNOT00000012938 | ENSRNOG00000009770 | Olr490 | olfactory receptor 490 | |
| rno-miR-370-3p | up | ENSRNOT00000074212 | ENSRNOG00000010588 | Tns2 | tensin 2 | |
| rno-miR-673-3p | up | ENSRNOT00000037500 | ENSRNOG00000027161 | RGD1359158 | similar to RIKEN cDNA 1110059E24 | |
| rno-miR-214-3p | up | ENSRNOT00000065370 | ENSRNOG00000030726 | Asgr2 | asialoglycoprotein receptor 2 | |
| rno-miR-127-3p | up | ENSRNOT00000087167 | ENSRNOG00000061845 | Cttnbp2 | cortactin binding protein 2 | |
| rno-miR-431 | up | ENSRNOT00000052237 | ENSRNOG00000033736 | Diras3 | DIRAS family GTPase 3 | |
| rno-miR-214-3p | up | ENSRNOT00000028490 | ENSRNOG00000020990 | Fgf21 | fibroblast growth factor 21 | |
| rno-miR-370-3p | up | ENSRNOT00000024112 | ENSRNOG00000017901 | Acy3 | aminoacylase 3 | |
| rno-miR-127-5p | up | ENSRNOT00000072782 | ENSRNOG00000046807 | LOC100910863 | olfactory receptor 143-like | |
| rno-miR-127-5p | up | ENSRNOT00000071307 | ENSRNOG00000049658 | LOC100911801 | olfactory receptor 143-like | |
| rno-miR-127-5p | up | ENSRNOT00000071964 | ENSRNOG00000046963 | LOC100911801 | olfactory receptor 143-like | |
| rno-miR-127-5p | up | ENSRNOT00000072741 | ENSRNOG00000047639 | LOC100912605 | olfactory receptor 5D14-like | |
| rno-miR-127-5p | up | ENSRNOT00000007782 | ENSRNOG00000050737 | Olr607 | olfactory receptor 607 | |
| rno-miR-673-3p | up | ENSRNOT00000014821 | ENSRNOG00000011039 | Gch1 | GTP cyclohydrolase 1 | |
| rno-miR-370-3p | up | ENSRNOT00000084372 | ENSRNOG00000061695 | Slc23a1 | solute carrier family 23 member 1 | |
| rno-miR-370-3p | up | ENSRNOT00000023605 | ENSRNOG00000017441 | Tpm3 | tropomyosin 3 | |
| rno-miR-370-3p | up | ENSRNOT00000013753 | ENSRNOG00000046864 | Acot4 | acyl-CoA thioesterase 4 | |
| rno-miR-673-3p | up | ENSRNOT00000016132 | ENSRNOG00000011830 | Plekha3 | pleckstrin homology domain containing A3 | |
| rno-miR-665 | up | ENSRNOT00000084045 | ENSRNOG00000051699 | Zdhhc4 | zinc finger, DHHC-type containing 4 | |
| rno-miR-673-3p | up | ENSRNOT00000018411 | ENSRNOG00000013585 | Nmnat3 | nicotinamide nucleotide adenylyltransferase 3 | |
| rno-miR-673-3p | up | ENSRNOT00000087788 | ENSRNOG00000055188 | Tlx2 | T-cell leukemia homeobox 2 | |
| rno-miR-665 | up | ENSRNOT00000082388 | ENSRNOG00000019372 | Pc | pyruvate carboxylase | |
| rno-miR-127-3p | up | ENSRNOT00000041960 | ENSRNOG00000015522 | Tfap2a | transcription factor AP-2 alpha | |
| rno-miR-485-5p | up | ENSRNOT00000086131 | ENSRNOG00000055344 | Trnau1ap | tRNA selenocysteine 1 associated protein 1 | |
| rno-miR-673-3p | up | ENSRNOT00000015545 | ENSRNOG00000011419 | Aldh6a1 | aldehyde dehydrogenase 6 family, member A1 | |
| rno-miR-370-3p | up | ENSRNOT00000089056 | ENSRNOG00000015496 | Tpm4 | tropomyosin 4 | |
| rno-miR-214-3p | up | ENSRNOT00000025652 | ENSRNOG00000018740 | Ugt1a5 | UDP glucuronosyltransferase family 1 member A5 | |
| rno-miR-214-3p | up | ENSRNOT00000071003 | ENSRNOG00000018740 | Ugt1a5 | UDP glucuronosyltransferase family 1 member A5 | |
| rno-miR-673-3p | up | ENSRNOT00000084003 | ENSRNOG00000056275 | AABR07052298.1 |  | |
| rno-miR-370-3p | up | ENSRNOT00000040778 | ENSRNOG00000024345 | Pard3b | par-3 family cell polarity regulator beta | |
| rno-miR-665 | up | ENSRNOT00000004999 | ENSRNOG00000003328 | Cdip1 | cell death-inducing p53 target 1 | |
| rno-miR-214-3p | up | ENSRNOT00000018616 | ENSRNOG00000013832 | Fam193a | family with sequence similarity 193, member A | |
| rno-miR-127-5p | up | ENSRNOT00000006152 | ENSRNOG00000004489 | Adgre5 | adhesion G protein-coupled receptor E5 | |
| rno-miR-665 | up | ENSRNOT00000041399 | ENSRNOG00000033020 | Vegp2 | von Ebners gland protein 2 | |
| rno-miR-370-3p | up | ENSRNOT00000084950 | ENSRNOG00000018047 | Gorasp1 | golgi reassembly stacking protein 1 | |
| rno-miR-665 | up | ENSRNOT00000013410 | ENSRNOG00000010094 | Kcnmb2 | potassium calcium-activated channel subfamily M regulatory beta subunit 2 | |
| rno-miR-673-3p | up | ENSRNOT00000082863 | ENSRNOG00000009563 | Krt2 | keratin 2 | |
| rno-miR-485-5p | up | ENSRNOT00000086789 | ENSRNOG00000017663 | Dolpp1 | dolichyldiphosphatase 1 | |
| rno-miR-665 | up | ENSRNOT00000042910 | ENSRNOG00000033761 | Lcn1 | lipocalin 1 | |
| rno-miR-370-3p | up | ENSRNOT00000033267 | ENSRNOG00000027971 | Lrrc31 | leucine rich repeat containing 31 | |
| rno-miR-665 | up | ENSRNOT00000012433 | ENSRNOG00000009346 | Zbtb26 | zinc finger and BTB domain containing 26 | |
| rno-miR-673-3p | up | ENSRNOT00000008913 | ENSRNOG00000006369 | Alg2 | ALG2, alpha-1,3/1,6-mannosyltransferase | |
| rno-miR-370-3p | up | ENSRNOT00000084494 | ENSRNOG00000059810 | Txnrd3 | thioredoxin reductase 3 | |
| rno-miR-370-3p | up | ENSRNOT00000057055 | ENSRNOG00000010835 | Dmbx1 | diencephalon/mesencephalon homeobox 1 | |
| rno-miR-665 | up | ENSRNOT00000028284 | ENSRNOG00000020845 | Tyrobp | Tyro protein tyrosine kinase binding protein | |
| rno-miR-665 | up | ENSRNOT00000088114 | ENSRNOG00000002767 | Dlg3 | discs large MAGUK scaffold protein 3 | |
| rno-miR-214-3p | up | ENSRNOT00000054757 | ENSRNOG00000036604 | Ifit2 | interferon-induced protein with tetratricopeptide repeats 2 | |
| rno-miR-665 | up | ENSRNOT00000084230 | ENSRNOG00000020817 | Cyp2a1 | cytochrome P450, family 2, subfamily a, polypeptide 1 | |
| rno-miR-665 | up | ENSRNOT00000090071 | ENSRNOG00000020817 | Cyp2a1 | cytochrome P450, family 2, subfamily a, polypeptide 1 | |
| rno-miR-31a-5p | up | ENSRNOT00000018853 | ENSRNOG00000014034 | Olfml2a | olfactomedin-like 2A | |
| rno-miR-370-3p | up | ENSRNOT00000000785 | ENSRNOG00000000634 | RGD1306739 | similar to RIKEN cDNA 1700040L02 | |
| rno-miR-370-3p | up | ENSRNOT00000060527 | ENSRNOG00000017889 | Serac1 | serine active site containing 1 | |
| rno-miR-370-3p | up | ENSRNOT00000038439 | ENSRNOG00000027260 | Adprh | ADP-ribosylarginine hydrolase | |
| rno-miR-370-3p | up | ENSRNOT00000006680 | ENSRNOG00000004766 | Glis2 | GLIS family zinc finger 2 | |
| rno-miR-665 | up | ENSRNOT00000026316 | ENSRNOG00000019372 | Pc | pyruvate carboxylase | |
| rno-miR-665 | up | ENSRNOT00000088242 | ENSRNOG00000006947 | Pdhx | pyruvate dehydrogenase complex, component X | |
| rno-miR-665 | up | ENSRNOT00000082205 | ENSRNOG00000016412 | Fxyd6 | FXYD domain-containing ion transport regulator 6 | |
| rno-miR-214-3p | up | ENSRNOT00000079290 | ENSRNOG00000020630 | Il9r | interleukin 9 receptor | |
| rno-miR-214-3p | up | ENSRNOT00000093711 | ENSRNOG00000025864 | Lemd2 | LEM domain containing 2 | |
| rno-miR-665 | up | ENSRNOT00000064308 | ENSRNOG00000048036 | Ncan | neurocan | |
| rno-miR-485-5p | up | ENSRNOT00000051670 | ENSRNOG00000047710 | Olr962 | olfactory receptor 962 | |
| rno-miR-370-3p | up | ENSRNOT00000002114 | ENSRNOG00000001548 | Nfe2l2 | nuclear factor, erythroid 2-like 2 | |
| rno-miR-370-3p | up | ENSRNOT00000016909 | ENSRNOG00000012392 | Dnajc2 | DnaJ heat shock protein family (Hsp40) member C2 ] | |
| rno-miR-214-3p | up | ENSRNOT00000079725 | ENSRNOG00000057487 | Krtap12-2 | keratin associated protein 12-2 | |
| rno-miR-214-3p | up | ENSRNOT00000067800 | ENSRNOG00000042956 | Krtap12-2 | keratin associated protein 12-2 | |
| rno-miR-370-3p | up | ENSRNOT00000024408 | ENSRNOG00000018047 | Gorasp1 | golgi reassembly stacking protein 1 | |
| rno-miR-370-3p | up | ENSRNOT00000070897 | ENSRNOG00000050655 | P4ha1 | prolyl 4-hydroxylase subunit alpha 1 | |
| rno-miR-370-3p | up | ENSRNOT00000092240 | ENSRNOG00000062002 | Kcna3 | potassium voltage-gated channel subfamily A member 3 | |
| rno-miR-665 | up | ENSRNOT00000079349 | ENSRNOG00000057924 | Olr437 | olfactory receptor 437 | |
| rno-miR-370-3p | up | ENSRNOT00000077374 | ENSRNOG00000059810 | Txnrd3 | thioredoxin reductase 3 | |
| rno-miR-665 | up | ENSRNOT00000021609 | ENSRNOG00000015751 | Cyp4f18 | cytochrome P450, family 4, subfamily f, polypeptide 18 | |
| rno-miR-214-3p | up | ENSRNOT00000015818 | ENSRNOG00000011207 | Gh1 | growth hormone 1 | |
| rno-miR-341 | up | ENSRNOT00000009066 | ENSRNOG00000006509 | Srgap3 | SLIT-ROBO Rho GTPase activating protein 3 | |
| rno-miR-673-3p | up | ENSRNOT00000027459 | ENSRNOG00000020202 | Asrgl1 | asparaginase like 1 | |
| rno-miR-665 | up | ENSRNOT00000010891 | ENSRNOG00000008245 | Ptgis | prostaglandin I2 synthase | |
| rno-miR-665 | up | ENSRNOT00000086091 | ENSRNOG00000016831 | Serpinh1 | serpin family H member 1 | |
| rno-miR-665 | up | ENSRNOT00000061878 | ENSRNOG00000006130 | Rab5b | RAB5B, member RAS oncogene family | |
| rno-miR-370-3p | up | ENSRNOT00000023567 | ENSRNOG00000017441 | Tpm3 | tropomyosin 3 | |
| rno-miR-665 | up | ENSRNOT00000085008 | ENSRNOG00000020817 | Cyp2a1 | cytochrome P450, family 2, subfamily a, polypeptide 1 | |
| rno-miR-665 | up | ENSRNOT00000054729 | ENSRNOG00000013241 | Cyp2c24 | cytochrome P450, family 2, subfamily c, polypeptide 24 | |
| rno-miR-341 | up | ENSRNOT00000028179 | ENSRNOG00000020771 | RGD1561590 | similar to SAP18 | |
| rno-miR-370-3p | up | ENSRNOT00000088928 | ENSRNOG00000016321 | Entpd4 | ectonucleoside triphosphate diphosphohydrolase 4 | |
| rno-miR-665 | up | ENSRNOT00000006776 | ENSRNOG00000005058 | Lpcat4 | lysophosphatidylcholine acyltransferase 4 | |
| rno-miR-127-3p | up | ENSRNOT00000033933 | ENSRNOG00000023812 | Raver2 | ribonucleoprotein, PTB-binding 2 | |
| rno-miR-370-3p | up | ENSRNOT00000024709 | ENSRNOG00000018018 | Kcnip2 | potassium voltage-gated channel interacting protein 2 | |
| rno-miR-370-3p | up | ENSRNOT00000024750 | ENSRNOG00000018018 | Kcnip2 | potassium voltage-gated channel interacting protein 2 | |
| rno-miR-370-3p | up | ENSRNOT00000024959 | ENSRNOG00000018467 | Mitd1 | microtubule interacting and trafficking domain containing 1 | |
| rno-miR-341 | up | ENSRNOT00000014397 | ENSRNOG00000010732 | Sap18 | Sin3-associated polypeptide 18 | |
| rno-miR-370-3p | up | ENSRNOT00000024581 | ENSRNOG00000018229 | Slc45a1 | solute carrier family 45, member 1 | |
| rno-miR-370-3p | up | ENSRNOT00000023762 | ENSRNOG00000014486 | Rfx3 | regulatory factor X3 | |
| rno-miR-665 | up | ENSRNOT00000018528 | ENSRNOG00000013753 | Tssk5 | testis-specific serine kinase 5 | |
| rno-miR-673-3p | up | ENSRNOT00000029250 | ENSRNOG00000026937 | Arsk | arylsulfatase family, member K | |
| rno-miR-127-3p | up | ENSRNOT00000027165 | ENSRNOG00000020038 | Chpf | chondroitin polymerizing factor | |
| rno-miR-370-3p | up | ENSRNOT00000023144 | ENSRNOG00000016818 | Fgfr3 | fibroblast growth factor receptor 3 | |
| rno-miR-673-3p | up | ENSRNOT00000018028 | ENSRNOG00000012690 | Nipa2 | non imprinted in Prader-Willi/Angelman syndrome 2 | |
| rno-miR-485-5p | up | ENSRNOT00000049221 | ENSRNOG00000032429 | Tmprss9 | transmembrane protease, serine 9 | |
| rno-miR-341 | up | ENSRNOT00000010252 | ENSRNOG00000007739 | Rars | arginyl-tRNA synthetase | |
| rno-miR-433-3p | up | ENSRNOT00000033749 | ENSRNOG00000015262 | Slc34a1 | solute carrier family 34 member 1 | |
| rno-miR-370-3p | up | ENSRNOT00000079846 | ENSRNOG00000019693 | Clpb | ClpB homolog, mitochondrial AAA ATPase chaperonin | |
| rno-novel-58-mature | up | ENSRNOT00000035277 | ENSRNOG00000014182 | Tns1 | tensin 1 | |
| rno-miR-136-3p | up | ENSRNOT00000087088 | ENSRNOG00000016550 | Dclk2 | doublecortin-like kinase 2 | |
| rno-miR-127-3p | up | ENSRNOT00000011560 | ENSRNOG00000008577 | Fbf1 | Fas binding factor 1 | |
| rno-miR-665 | up | ENSRNOT00000073233 | ENSRNOG00000050445 | Gde1 | glycerophosphodiester phosphodiesterase 1 | |
| rno-miR-370-3p | up | ENSRNOT00000027170 | ENSRNOG00000020029 | Mcrip2 | MAPK regulated co-repressor interacting protein 2 | |
| rno-miR-665 | up | ENSRNOT00000051473 | ENSRNOG00000024376 | Zfp111 | zinc finger protein 111 | |
| rno-miR-370-3p | up | ENSRNOT00000070807 | ENSRNOG00000047080 | Gng4 | G protein subunit gamma 4 | |
| rno-miR-370-3p | up | ENSRNOT00000021073 | ENSRNOG00000015496 | Tpm4 | tropomyosin 4 | |
| rno-miR-370-3p | up | ENSRNOT00000038069 | ENSRNOG00000033624 | Zfp426 | zinc finger protein 426 | |
| rno-miR-673-3p | up | ENSRNOT00000020123 | ENSRNOG00000014928 | Apba1 | amyloid beta precursor protein binding family A member 1 | |
| rno-miR-212-5p | up | ENSRNOT00000035034 | ENSRNOG00000022182 | Rint1 | RAD50 interactor 1 | |
| rno-miR-337-5p | up | ENSRNOT00000001387 | ENSRNOG00000001043 | Ddx55 | DEAD-box helicase 55 | |
| rno-miR-673-3p | up | ENSRNOT00000088720 | ENSRNOG00000060335 | Strn3 | striatin 3 | |
| rno-miR-6331 | up | ENSRNOT00000024129 | ENSRNOG00000017944 | Tbx10 | T-box 10 | |
| rno-miR-665 | up | ENSRNOT00000022437 | ENSRNOG00000016459 | Eif3j | eukaryotic translation initiation factor 3, subunit J | |
| rno-miR-341 | up | ENSRNOT00000051989 | ENSRNOG00000001774 | Lrch3 | leucine rich repeats and calponin homology domain containing 3 | |
| rno-miR-341 | up | ENSRNOT00000063883 | ENSRNOG00000001774 | Lrch3 | leucine rich repeats and calponin homology domain containing 3 | |
| rno-miR-341 | up | ENSRNOT00000073865 | ENSRNOG00000001774 | Lrch3 | leucine rich repeats and calponin homology domain containing 3 | |
| rno-miR-370-3p | up | ENSRNOT00000048754 | ENSRNOG00000015473 | Phactr2 | phosphatase and actin regulator 2 | |
| rno-miR-6331 | up | ENSRNOT00000088279 | ENSRNOG00000058739 | Snn | stannin | |
| rno-miR-673-3p | up | ENSRNOT00000077578 | ENSRNOG00000060335 | Strn3 | striatin 3 | |
| rno-miR-370-3p | up | ENSRNOT00000036282 | ENSRNOG00000026186 | Syde2 | synapse defective Rho GTPase homolog 2 | |
| rno-miR-485-5p | up | ENSRNOT00000066037 | ENSRNOG00000032429 | Tmprss9 | transmembrane protease, serine 9 | |
| rno-miR-665 | up | ENSRNOT00000006345 | ENSRNOG00000024025 | Nrn1l | neuritin 1-like | |
| rno-miR-341 | up | ENSRNOT00000045657 | ENSRNOG00000028501 | Zc3h18 | zinc finger CCCH-type containing 18 | |
| rno-miR-370-3p | up | ENSRNOT00000076329 | ENSRNOG00000014030 | Synm | synemin | |
| rno-miR-140-3p | up | ENSRNOT00000014323 | ENSRNOG00000025676 | Fam198a | family with sequence similarity 198, member A | |
| rno-miR-370-3p | up | ENSRNOT00000011904 | ENSRNOG00000008697 | Nov | nephroblastoma overexpressed | |
| rno-miR-214-3p | up | ENSRNOT00000038748 | ENSRNOG00000026061 | RGD1566226 | similar to hypothetical protein F830045P16 | |
| rno-miR-370-3p | up | ENSRNOT00000087043 | ENSRNOG00000011550 | Kcnab2 | potassium voltage-gated channel subfamily A regulatory beta subunit 2 | |
| rno-miR-673-3p | up | ENSRNOT00000030706 | ENSRNOG00000022697 | Clec14a | C-type lectin domain family 14, member A | |
| rno-miR-665 | up | ENSRNOT00000022983 | ENSRNOG00000016831 | Serpinh1 | serpin family H member 1 | |
| rno-miR-370-3p | up | ENSRNOT00000050721 | ENSRNOG00000020651 | Cars | cysteinyl-tRNA synthetase | |
| rno-miR-370-3p | up | ENSRNOT00000064428 | ENSRNOG00000016321 | Entpd4 | ectonucleoside triphosphate diphosphohydrolase 4 | |
| rno-miR-132-5p | up | ENSRNOT00000090987 | ENSRNOG00000059474 | Mob1a | MOB kinase activator 1A | |
| rno-miR-140-3p | up | ENSRNOT00000028479 | ENSRNOG00000020981 | Prss46 | protease, serine, 46 | |
| rno-miR-485-5p | up | ENSRNOT00000072069 | ENSRNOG00000050697 | Ctsz | cathepsin Z | |
| rno-miR-127-3p | up | ENSRNOT00000019930 | ENSRNOG00000014642 | Morn1 | MORN repeat containing 1 | |
| rno-miR-337-5p | up | ENSRNOT00000014548 | ENSRNOG00000010597 | Slc5a7 | solute carrier family 5 member 7 | |
| rno-miR-370-3p | up | ENSRNOT00000068461 | ENSRNOG00000020356 | Zfp846 | zinc finger protein 846 | |
| rno-miR-370-3p | up | ENSRNOT00000036527 | ENSRNOG00000025141 | Kiz | kizuna centrosomal protein | |
| rno-miR-370-3p | up | ENSRNOT00000045963 | ENSRNOG00000030034 | Sox11 | SRY box 11 | |
| rno-miR-665 | up | ENSRNOT00000026495 | ENSRNOG00000019403 | Afap1l1 | actin filament associated protein 1-like 1 | |
| rno-miR-665 | up | ENSRNOT00000010602 | ENSRNOG00000007842 | Aup1 | ancient ubiquitous protein 1 | |
| rno-miR-665 | up | ENSRNOT00000044206 | ENSRNOG00000011846 | Krt28 | keratin 28 | |
| rno-miR-409a-3p | up | ENSRNOT00000027550 | ENSRNOG00000020280 | Armc6 | armadillo repeat containing 6 | |
| rno-miR-370-3p | up | ENSRNOT00000014409 | ENSRNOG00000010741 | Tigd4 | tigger transposable element derived 4 | |
| rno-miR-127-3p | up | ENSRNOT00000025923 | ENSRNOG00000019176 | Kcne5 | potassium voltage-gated channel subfamily E regulatory subunit 5 | |
| rno-miR-673-3p | up | ENSRNOT00000092157 | ENSRNOG00000008788 | Mpp5 | membrane palmitoylated protein 5 | |
| rno-miR-665 | up | ENSRNOT00000045165 | ENSRNOG00000018674 | Ntrk3 | neurotrophic receptor tyrosine kinase 3 | |
| rno-miR-665 | up | ENSRNOT00000046849 | ENSRNOG00000018674 | Ntrk3 | neurotrophic receptor tyrosine kinase 3 | |
| rno-miR-665 | up | ENSRNOT00000025536 | ENSRNOG00000018674 | Ntrk3 | neurotrophic receptor tyrosine kinase 3 | |
| rno-miR-214-3p | up | ENSRNOT00000085765 | ENSRNOG00000032788 | Dysf | dysferlin | |
| rno-miR-673-3p | up | ENSRNOT00000016026 | ENSRNOG00000011589 | Camk2d | calcium/calmodulin-dependent protein kinase II delta | |
| rno-miR-370-3p | up | ENSRNOT00000089142 | ENSRNOG00000015428 | Mff | mitochondrial fission factor | |
| rno-miR-341 | up | ENSRNOT00000073188 | ENSRNOG00000046434 | Hist1h2ail1 | histone cluster 1, H2ai-like1 | |
| rno-miR-341 | up | ENSRNOT00000074024 | ENSRNOG00000049198 | Hist2h3c2 | histone cluster 2, H3c2 | |
| rno-miR-341 | up | ENSRNOT00000081042 | ENSRNOG00000053081 | Hist2h3c2 | histone cluster 2, H3c2 | |
| rno-miR-341 | up | ENSRNOT00000085945 | ENSRNOG00000060366 | Hist2h3c2 | histone cluster 2, H3c2 | |
| rno-miR-673-3p | up | ENSRNOT00000079631 | ENSRNOG00000021097 | Hpn | hepsin | |
| rno-miR-341 | up | ENSRNOT00000040906 | ENSRNOG00000028975 | LOC102548682 | histone H4-like | |
| rno-miR-673-3p | up | ENSRNOT00000073951 | ENSRNOG00000047393 | Krt18 | keratin 18 | |
| rno-miR-370-3p | up | ENSRNOT00000003714 | ENSRNOG00000002751 | Zdhhc15 | zinc finger, DHHC-type containing 15 | |
| rno-miR-370-3p | up | ENSRNOT00000021144 | ENSRNOG00000015727 | Loxl4 | lysyl oxidase-like 4 | |
| rno-novel-58-mature | up | ENSRNOT00000091931 | ENSRNOG00000056482 | AABR07068750.1 |  | |
| rno-miR-370-3p | up | ENSRNOT00000007298 | ENSRNOG00000005061 | Dlst | dihydrolipoamide S-succinyltransferase | |
| rno-miR-665 | up | ENSRNOT00000021103 | ENSRNOG00000015763 | Nat8f3 | N-acetyltransferase 8 (GCN5-related) family member 3 | |
| rno-miR-370-3p | up | ENSRNOT00000074743 | ENSRNOG00000045629 | Atp23 | ATP23 metallopeptidase and ATP synthase assembly factor homolog | |
| rno-miR-665 | up | ENSRNOT00000078994 | ENSRNOG00000014152 | Kcnip3 | potassium voltage-gated channel interacting protein 3 | |
| rno-miR-370-3p | up | ENSRNOT00000083770 | ENSRNOG00000051487 | Kremen1 | kringle containing transmembrane protein 1 | |
| rno-miR-673-3p | up | ENSRNOT00000022898 | ENSRNOG00000016892 | Nr2f6 | nuclear receptor subfamily 2, group F, member 6 | |
| rno-miR-214-3p | up | ENSRNOT00000019674 | ENSRNOG00000014519 | Slc35g3 | solute carrier family 35, member G3 | |
| rno-miR-665 | up | ENSRNOT00000022032 | ENSRNOG00000016412 | Fxyd6 | FXYD domain-containing ion transport regulator 6 | |
| rno-miR-370-3p | up | ENSRNOT00000010266 | ENSRNOG00000007810 | Gdf6 | growth differentiation factor 6 | |
| rno-miR-665 | up | ENSRNOT00000024858 | ENSRNOG00000018379 | Zfp688 | zinc finger protein 688 | |
| rno-miR-204-5p | up | ENSRNOT00000019698 | ENSRNOG00000014678 | Fzd5 | frizzled class receptor 5 | |
| rno-miR-370-3p | up | ENSRNOT00000068278 | ENSRNOG00000018018 | Kcnip2 | potassium voltage-gated channel interacting protein 2 | |
| rno-miR-370-3p | up | ENSRNOT00000022954 | ENSRNOG00000017050 | Lmntd2 | lamin tail domain containing 2 | |
| rno-miR-673-3p | up | ENSRNOT00000084632 | ENSRNOG00000006756 | Maged1 | MAGE family member D1 | |
| rno-miR-370-3p | up | ENSRNOT00000090870 | ENSRNOG00000018467 | Mitd1 | microtubule interacting and trafficking domain containing 1 | |
| rno-miR-370-3p | up | ENSRNOT00000020705 | ENSRNOG00000015428 | Mff | mitochondrial fission factor | |
| rno-miR-370-3p | up | ENSRNOT00000077859 | ENSRNOG00000015428 | Mff | mitochondrial fission factor [Source:RGD Symbol;Acc:1310230] | |
| rno-miR-673-3p | up | ENSRNOT00000077943 | ENSRNOG00000034025 | Ptprj | protein tyrosine phosphatase, receptor type, J | |
| rno-miR-665 | up | ENSRNOT00000005458 | ENSRNOG00000004108 | AABR07063901.1 |  | |
| rno-miR-214-3p | up | ENSRNOT00000073743 | ENSRNOG00000050172 | Thpo | thrombopoietin | |
| rno-miR-673-3p | up | ENSRNOT00000064105 | ENSRNOG00000013742 | Large1 | LARGE xylosyl- and glucuronyltransferase 1 | |
| rno-miR-370-3p | up | ENSRNOT00000080207 | ENSRNOG00000016070 | Trpc3 | transient receptor potential cation channel, subfamily C, member 3 | |
| rno-miR-665 | up | ENSRNOT00000019707 | ENSRNOG00000014633 | Vps28 | VPS28, ESCRT-I subunit | |
| rno-miR-370-3p | up | ENSRNOT00000088936 | ENSRNOG00000000838 | Lta | lymphotoxin alpha | |
| rno-miR-370-3p | up | ENSRNOT00000023651 | ENSRNOG00000017506 | Cltb | clathrin, light chain B | |
| rno-miR-370-3p | up | ENSRNOT00000018634 | ENSRNOG00000013886 | Fyb | FYN binding protein | |
| rno-miR-370-3p | up | ENSRNOT00000005325 | ENSRNOG00000004000 | Tefm | transcription elongation factor, mitochondrial | |
| rno-miR-370-3p | up | ENSRNOT00000086164 | ENSRNOG00000050450 | LOC100911951 | Kv channel-interacting protein 2-like | |
| rno-miR-665 | up | ENSRNOT00000022375 | ENSRNOG00000016670 | Pip4k2a | phosphatidylinositol-5-phosphate 4-kinase type 2 alpha | |
| rno-miR-665 | up | ENSRNOT00000071305 | ENSRNOG00000046863 | C1ql2 | complement C1q like 2 | |
| rno-miR-673-3p | up | ENSRNOT00000014924 | ENSRNOG00000010780 | Dlc1 | DLC1 Rho GTPase activating protein | |
| rno-miR-127-3p | up | ENSRNOT00000004361 | ENSRNOG00000003268 | Maml1 | mastermind-like transcriptional coactivator 1 | |
| rno-miR-665 | up | ENSRNOT00000021153 | ENSRNOG00000015741 | Slc2a13 | solute carrier family 2 member 13 | |
| rno-miR-665 | up | ENSRNOT00000068712 | ENSRNOG00000000436 | Egfl8 | EGF-like-domain, multiple 8 | |
| rno-miR-127-5p | up | ENSRNOT00000047700 | ENSRNOG00000026430 | Trim58 | tripartite motif-containing 58 | |
| rno-miR-370-3p | up | ENSRNOT00000033722 | ENSRNOG00000025589 | Jph4 | junctophilin 4 | |
| rno-miR-370-3p | up | ENSRNOT00000009334 | ENSRNOG00000006926 | Atp6v0d2 | ATPase H+ transporting V0 subunit D2 | |
| rno-miR-370-3p | up | ENSRNOT00000050284 | ENSRNOG00000033734 | Tnnt2 | troponin T2, cardiac type | |
| rno-miR-673-3p | up | ENSRNOT00000090886 | ENSRNOG00000013742 | Large1 | LARGE xylosyl- and glucuronyltransferase 1 | |
| rno-miR-370-3p | up | ENSRNOT00000030841 | ENSRNOG00000027891 | Dhrs11 | dehydrogenase/reductase 11 | |
| rno-miR-665 | up | ENSRNOT00000019993 | ENSRNOG00000014526 | Cars2 | cysteinyl-tRNA synthetase 2, mitochondrial | |
| rno-miR-665 | up | ENSRNOT00000023067 | ENSRNOG00000017129 | Ttll4 | tubulin tyrosine ligase like 4 | |
| rno-miR-665 | up | ENSRNOT00000008659 | ENSRNOG00000009972 | Rara | retinoic acid receptor, alpha | |
| rno-miR-485-5p | up | ENSRNOT00000075298 | ENSRNOG00000046262 | 3110082J24Rik | RIKEN cDNA 3110082J24 gene | |
| rno-miR-665 | up | ENSRNOT00000041839 | ENSRNOG00000018674 | Ntrk3 | neurotrophic receptor tyrosine kinase 3 | |
| rno-miR-214-3p | up | ENSRNOT00000022841 | ENSRNOG00000016984 | Atg13 | autophagy related 13 | |
| rno-miR-665 | up | ENSRNOT00000029395 | ENSRNOG00000021609 | LOC100912566 | UPF0500 protein C1orf216 homolog | |
| rno-novel-125-mature | up | ENSRNOT00000087961 | ENSRNOG00000020615 | Nap1l4 | nucleosome assembly protein 1-like 4 | |
| rno-miR-370-3p | up | ENSRNOT00000029386 | ENSRNOG00000036719 | Samd10 | sterile alpha motif domain containing 10 | |
| rno-miR-673-3p | up | ENSRNOT00000016175 | ENSRNOG00000012098 | Adcyap1r1 | adenylate cyclase activating polypeptide 1 receptor type 1 | |
| rno-miR-673-3p | up | ENSRNOT00000035722 | ENSRNOG00000012098 | Adcyap1r1 | adenylate cyclase activating polypeptide 1 receptor type 1 | |
| rno-miR-673-3p | up | ENSRNOT00000043851 | ENSRNOG00000012098 | Adcyap1r1 | adenylate cyclase activating polypeptide 1 receptor type 1 | |
| rno-miR-673-3p | up | ENSRNOT00000046192 | ENSRNOG00000012098 | Adcyap1r1 | adenylate cyclase activating polypeptide 1 receptor type 1 | |
| rno-miR-214-3p | up | ENSRNOT00000064626 | ENSRNOG00000042736 | LOC680442 | hypothetical protein LOC680442 | |
| rno-miR-370-3p | up | ENSRNOT00000003512 | ENSRNOG00000002592 | Rps6ka6 | ribosomal protein S6 kinase A6 | |
| rno-miR-673-3p | up | ENSRNOT00000059565 | ENSRNOG00000010888 | Ankrd33b | ankyrin repeat domain 33B | |
| rno-miR-31a-5p | up | ENSRNOT00000001970 | ENSRNOG00000001450 | Nsun5 | NOP2/Sun RNA methyltransferase family member 5 | |
| rno-miR-665 | up | ENSRNOT00000084919 | ENSRNOG00000017791 | Arhgap12 | Rho GTPase activating protein 12 | |
| rno-miR-485-5p | up | ENSRNOT00000082071 | ENSRNOG00000004577 | Fez2 | fasciculation and elongation protein zeta 2 | |
| rno-miR-370-3p | up | ENSRNOT00000026883 | ENSRNOG00000019780 | Sypl2 | synaptophysin-like 2 | |
| rno-miR-370-3p | up | ENSRNOT00000050760 | ENSRNOG00000020369 | Igf2 | insulin-like growth factor 2 | |
| rno-miR-132-3p | up | ENSRNOT00000016051 | ENSRNOG00000011949 | Ndufb5 | NADH:ubiquinone oxidoreductase subunit B5 | |
| rno-miR-370-3p | up | ENSRNOT00000005039 | ENSRNOG00000003784 | Rilp | Rab interacting lysosomal protein | |
| rno-miR-214-3p | up | ENSRNOT00000004578 | ENSRNOG00000003288 | Cacng5 | calcium voltage-gated channel auxiliary subunit gamma 5 | |
| rno-miR-673-3p | up | ENSRNOT00000024080 | ENSRNOG00000017916 | Rexo1 | RNA exonuclease 1 homolog | |
| rno-miR-370-3p | up | ENSRNOT00000084986 | ENSRNOG00000033734 | Tnnt2 | troponin T2, cardiac type | |
| rno-miR-673-3p | up | ENSRNOT00000086122 | ENSRNOG00000053828 | Ppp1r12c | protein phosphatase 1, regulatory subunit 12C | |
| rno-miR-665 | up | ENSRNOT00000022327 | ENSRNOG00000022274 | Slc2a8 | solute carrier family 2 member 8 | |
| rno-miR-673-3p | up | ENSRNOT00000070834 | ENSRNOG00000049782 | Csf2ra | colony stimulating factor 2 receptor alpha subunit | |
| rno-miR-370-3p | up | ENSRNOT00000088032 | ENSRNOG00000059479 | Adcy1 | adenylate cyclase 1 | |
| rno-miR-665 | up | ENSRNOT00000024344 | ENSRNOG00000017874 | Cd53 | Cd53 molecule | |
| rno-miR-370-3p | up | ENSRNOT00000037041 | ENSRNOG00000059019 | AABR07027339.1 |  | |
| rno-miR-673-3p | up | ENSRNOT00000022986 | ENSRNOG00000016955 | Fam188a | family with sequence similarity 188, member A | |
| rno-miR-214-3p | up | ENSRNOT00000037788 | ENSRNOG00000027376 | Il17rc | interleukin 17 receptor C | |
| rno-miR-673-3p | up | ENSRNOT00000022956 | ENSRNOG00000017087 | Man1c1 | mannosidase, alpha, class 1C, member 1 | |
| rno-miR-370-3p | up | ENSRNOT00000023495 | ENSRNOG00000017144 | Mea1 | male-enhanced antigen 1 | |
| rno-miR-665 | up | ENSRNOT00000077514 | ENSRNOG00000011936 | Abhd14a | abhydrolase domain containing 14A | |
| rno-miR-370-3p | up | ENSRNOT00000037059 | ENSRNOG00000028531 | Ccl25 | C-C motif chemokine ligand 25 | |
| rno-miR-370-3p | up | ENSRNOT00000073850 | ENSRNOG00000020369 | Igf2 | insulin-like growth factor 2 | |
| rno-miR-665 | up | ENSRNOT00000064510 | ENSRNOG00000016883 | Entpd7 | ectonucleoside triphosphate diphosphohydrolase 7 | |
| rno-miR-673-3p | up | ENSRNOT00000032386 | ENSRNOG00000021731 | LOC103689949 | nedd4 binding protein 3 | |
| rno-miR-673-3p | up | ENSRNOT00000024174 | ENSRNOG00000017951 | Ranbp9 | RAN binding protein 9 | |
| rno-miR-127-3p | up | ENSRNOT00000002188 | ENSRNOG00000001608 | Nxpe3 | neurexophilin and PC-esterase domain family, member 3 | |
| rno-miR-370-3p | up | ENSRNOT00000067916 | ENSRNOG00000018762 | Rsph3 | radial spoke 3 homolog | |
| rno-miR-665 | up | ENSRNOT00000019739 | ENSRNOG00000014706 | Dbx1 | developing brain homeobox 1 | |
| rno-miR-382-5p | up | ENSRNOT00000031164 | ENSRNOG00000028629 | Akt1 | AKT serine/threonine kinase 1 | |
| rno-miR-485-5p | up | ENSRNOT00000015147 | ENSRNOG00000011335 | Gpr50 | G protein-coupled receptor 50 | |
| rno-miR-370-3p | up | ENSRNOT00000027353 | ENSRNOG00000020140 | Pigq | phosphatidylinositol glycan anchor biosynthesis, class Q | |
| rno-novel-58-mature | up | ENSRNOT00000020041 | ENSRNOG00000014719 | Snx19 | sorting nexin 19 | |
| rno-miR-665 | up | ENSRNOT00000026184 | ENSRNOG00000019264 | Ltbr | lymphotoxin beta receptor | |
| rno-miR-665 | up | ENSRNOT00000019127 | ENSRNOG00000026295 | Rbpjl | recombination signal binding protein for immunoglobulin kappa J region-like | |
| rno-miR-370-3p | up | ENSRNOT00000010423 | ENSRNOG00000007853 | Prdm10 | PR/SET domain 10 | |
| rno-miR-485-5p | up | ENSRNOT00000068507 | ENSRNOG00000042602 | AABR07009224.1 |  | |
| rno-miR-370-3p | up | ENSRNOT00000026262 | ENSRNOG00000021521 | Chst5 | carbohydrate (N-acetylglucosamine 6-O) sulfotransferase 5 | |
| rno-miR-665 | up | ENSRNOT00000070807 | ENSRNOG00000047080 | Gng4 | G protein subunit gamma 4 | |
| rno-miR-673-3p | up | ENSRNOT00000077243 | ENSRNOG00000049782 | Csf2ra | colony stimulating factor 2 receptor alpha subunit | |
| rno-miR-127-3p | up | ENSRNOT00000024280 | ENSRNOG00000017737 | Dgkz | diacylglycerol kinase zeta | |
| rno-miR-370-3p | up | ENSRNOT00000080734 | ENSRNOG00000045821 | Slc41a3 | solute carrier family 41, member 3 | |
| rno-miR-370-3p | up | ENSRNOT00000010433 | ENSRNOG00000007763 | Plod1 | procollagen-lysine, 2-oxoglutarate 5-dioxygenase 1 | |
| rno-miR-665 | up | ENSRNOT00000092712 | ENSRNOG00000002755 | Pafah1b1 | platelet-activating factor acetylhydrolase 1b, regulatory subunit 1 | |
| rno-miR-212-5p | up | ENSRNOT00000028057 | ENSRNOG00000020673 | Pbxip1 | PBX homeobox interacting protein 1 | |
| rno-miR-673-3p | up | ENSRNOT00000075465 | ENSRNOG00000045965 | N4bp3 | Nedd4 binding protein 3 | |
| rno-miR-214-3p | up | ENSRNOT00000019690 | ENSRNOG00000014629 | Lmo1 | LIM domain only 1 | |
| rno-miR-214-3p | up | ENSRNOT00000073764 | ENSRNOG00000042736 | LOC680442 | hypothetical protein LOC680442 | |
| rno-miR-665 | up | ENSRNOT00000085328 | ENSRNOG00000015751 | Cyp4f18 | cytochrome P450, family 4, subfamily f, polypeptide 18 [Source:RGD Symbol;Acc:1305261] | |
| rno-miR-370-3p | up | ENSRNOT00000024900 | ENSRNOG00000018220 | Pde4dip | phosphodiesterase 4D interacting protein | |
| rno-miR-370-3p | up | ENSRNOT00000088041 | ENSRNOG00000018220 | Pde4dip | phosphodiesterase 4D interacting protein | |
| rno-miR-370-3p | up | ENSRNOT00000018402 | ENSRNOG00000013572 | Lxn | latexin | |
| rno-miR-341 | up | ENSRNOT00000049245 | ENSRNOG00000012367 | Pcdh7 | protocadherin 7 | |
| rno-miR-370-3p | up | ENSRNOT00000005873 | ENSRNOG00000004300 | Gtf2a1 | general transcription factor 2A subunit 1 | |
| rno-miR-341 | up | ENSRNOT00000077318 | ENSRNOG00000015623 | Guca1b | guanylate cyclase activator 1B | |
| rno-miR-214-3p | up | ENSRNOT00000066534 | ENSRNOG00000002827 | Rbfox1 | RNA binding protein, fox-1 homolog 1 | |
| rno-miR-127-3p | up | ENSRNOT00000092684 | ENSRNOG00000052296 | Shank3 | SH3 and multiple ankyrin repeat domains 3 | |
| rno-miR-127-3p | up | ENSRNOT00000092431 | ENSRNOG00000052296 | Shank3 | SH3 and multiple ankyrin repeat domains 3 | |
| rno-miR-370-3p | up | ENSRNOT00000000662 | ENSRNOG00000000551 | RGD1305587 | similar to RIKEN cDNA 2010107G23 | |
| rno-miR-382-5p | up | ENSRNOT00000006720 | ENSRNOG00000005059 | Med22 | mediator complex subunit 22 | |
| rno-miR-665 | up | ENSRNOT00000026897 | ENSRNOG00000019858 | Rom1 | retinal outer segment membrane protein 1 | |
| rno-miR-485-5p | up | ENSRNOT00000029728 | ENSRNOG00000021781 | Camk1 | calcium/calmodulin-dependent protein kinase I | |
| rno-miR-370-3p | up | ENSRNOT00000085380 | ENSRNOG00000057125 | Ddr1 | discoidin domain receptor tyrosine kinase 1 | |
| rno-miR-370-3p | up | ENSRNOT00000075700 | ENSRNOG00000048848 | Ccdc9 | coiled-coil domain containing 9 | |
| rno-miR-665 | up | ENSRNOT00000028892 | ENSRNOG00000021269 | Chgb | chromogranin B | |
| rno-miR-665 | up | ENSRNOT00000009564 | ENSRNOG00000007128 | Nop56 | NOP56 ribonucleoprotein | |
| rno-miR-212-5p | up | ENSRNOT00000008282 | ENSRNOG00000006237 | Gpr37l1 | G protein-coupled receptor 37-like 1 | |
| rno-miR-665 | up | ENSRNOT00000080068 | ENSRNOG00000001422 | Col26a1 | collagen type XXVI alpha 1 chain | |
| rno-miR-370-3p | up | ENSRNOT00000075725 | ENSRNOG00000047934 | RGD1566359 | similar to RIKEN cDNA B230219D22 | |
| rno-miR-665 | up | ENSRNOT00000015418 | ENSRNOG00000011544 | Zfp219 | zinc finger protein 219 | |
| rno-miR-665 | up | ENSRNOT00000016056 | ENSRNOG00000011936 | Abhd14a | abhydrolase domain containing 14A | |
| rno-miR-370-3p | up | ENSRNOT00000090417 | ENSRNOG00000016701 | Gng8 | G protein subunit gamma 8 | |
| rno-miR-214-3p | up | ENSRNOT00000077544 | ENSRNOG00000014486 | Rfx3 | regulatory factor X3 | |
| rno-miR-370-3p | up | ENSRNOT00000058810 | ENSRNOG00000038552 | AABR07009978.1 |  | |
| rno-miR-665 | up | ENSRNOT00000073072 | ENSRNOG00000047653 | Crybb1 | crystallin, beta B1 | |
| rno-miR-370-3p | up | ENSRNOT00000003886 | ENSRNOG00000002852 | Myo19 | myosin XIX | |
| rno-miR-370-3p | up | ENSRNOT00000036231 | ENSRNOG00000027393 | AC129824.1 |  | |
| rno-miR-140-3p | up | ENSRNOT00000010254 | ENSRNOG00000007647 | Oprk1 | opioid receptor, kappa 1 | |
| rno-miR-382-5p | up | ENSRNOT00000066193 | ENSRNOG00000049749 | LOC100911917 | mediator of RNA polymerase II transcription subunit 22-like | |
| rno-miR-370-3p | up | ENSRNOT00000085946 | ENSRNOG00000057125 | Ddr1 | discoidin domain receptor tyrosine kinase 1 | |
| rno-miR-370-3p | up | ENSRNOT00000089417 | ENSRNOG00000057125 | Ddr1 | discoidin domain receptor tyrosine kinase 1 | |
| rno-miR-665 | up | ENSRNOT00000080873 | ENSRNOG00000053656 | LOC690020 | similar to killer cell lectin-like receptor, subfamily A, member 17 | |
| rno-miR-485-5p | up | ENSRNOT00000087090 | ENSRNOG00000007254 | Ttc9 | tetratricopeptide repeat domain 9 | |
| rno-miR-127-3p | up | ENSRNOT00000067519 | ENSRNOG00000013878 | Hmgxb4 | HMG-box containing 4 | |
| rno-miR-665 | up | ENSRNOT00000003249 | ENSRNOG00000002378 | Bpnt1 | 3'(2'), 5'-bisphosphate nucleotidase 1 | |
| rno-miR-127-5p | up | ENSRNOT00000046330 | ENSRNOG00000032101 | Morc1 | MORC family CW-type zinc finger 1 | |
| rno-miR-665 | up | ENSRNOT00000030967 | ENSRNOG00000024025 | Nrn1l | neuritin 1-like | |
| rno-miR-665 | up | ENSRNOT00000088822 | ENSRNOG00000055433 | Trim46 | tripartite motif-containing 46 | |
| rno-miR-370-3p | up | ENSRNOT00000001248 | ENSRNOG00000000940 | Flt1 | FMS-related tyrosine kinase 1 | |
| rno-miR-673-3p | up | ENSRNOT00000036394 | ENSRNOG00000057650 | Hoxa10 | homeobox A10 | |
| rno-miR-214-3p | up | ENSRNOT00000079743 | ENSRNOG00000013437 | LOC691083 | hypothetical protein LOC691083 | |
| rno-miR-665 | up | ENSRNOT00000075153 | ENSRNOG00000047005 | Kcnk5 | potassium two pore domain channel subfamily K member 5 | |
| rno-miR-370-3p | up | ENSRNOT00000049985 | ENSRNOG00000001074 | Mphosph9 | M-phase phosphoprotein 9 | |
| rno-miR-665 | up | ENSRNOT00000077911 | ENSRNOG00000054917 | Mrgpre | MAS related GPR family member E | |
| rno-miR-493-3p | up | ENSRNOT00000027585 | ENSRNOG00000020354 | Mrpl23 | mitochondrial ribosomal protein L23 | |
| rno-miR-370-3p | up | ENSRNOT00000087443 | ENSRNOG00000061316 | LOC102547056 | retinoic acid early-inducible protein 1-gamma-like | |
| rno-miR-370-3p | up | ENSRNOT00000010720 | ENSRNOG00000007834 | Cand1 | cullin-associated and neddylation-dissociated 1 | |
| rno-miR-485-5p | up | ENSRNOT00000042735 | ENSRNOG00000004577 | Fez2 | fasciculation and elongation protein zeta 2 | |
| rno-miR-665 | up | ENSRNOT00000038016 | ENSRNOG00000027456 | Cdc42bpg | CDC42 binding protein kinase gamma | |
| rno-miR-127-5p | up | ENSRNOT00000044921 | ENSRNOG00000033231 | Gm12845 | predicted gene 12845 | |
| rno-miR-665 | up | ENSRNOT00000055536 | ENSRNOG00000017528 | Gpr157 | G protein-coupled receptor 157 | |
| rno-miR-431 | up | ENSRNOT00000043344 | ENSRNOG00000049289 | LOC100911068 | roundabout homolog 4-like | |
| rno-novel-58-mature | up | ENSRNOT00000011994 | ENSRNOG00000039544 | Kcnd1 | potassium voltage-gated channel subfamily D member 1 | |
| rno-miR-673-3p | up | ENSRNOT00000057347 | ENSRNOG00000024142 | Arglu1 | arginine and glutamate rich 1 | |
| rno-miR-665 | up | ENSRNOT00000088921 | ENSRNOG00000002378 | Bpnt1 | 3'(2'), 5'-bisphosphate nucleotidase 1 | |
| rno-miR-370-3p | up | ENSRNOT00000081409 | ENSRNOG00000055246 | Ncor1 | nuclear receptor co-repressor 1 | |
| rno-miR-214-3p | up | ENSRNOT00000075086 | ENSRNOG00000050766 | Rd3 | retinal degeneration 3 | |
| rno-miR-127-5p | up | ENSRNOT00000027297 | ENSRNOG00000020165 | Ahsp | alpha hemoglobin stabilizing protein | |
| rno-novel-125-mature | up | ENSRNOT00000054862 | ENSRNOG00000020615 | Nap1l4 | nucleosome assembly protein 1-like 4 | |
| rno-miR-127-5p | up | ENSRNOT00000070899 | ENSRNOG00000045902 | Izumo2 | IZUMO family member 2 | |
| rno-miR-127-5p | up | ENSRNOT00000073798 | ENSRNOG00000045902 | Izumo2 | IZUMO family member 2 | |
| rno-miR-127-5p | up | ENSRNOT00000071054 | ENSRNOG00000047254 | LOC100911976 | izumo sperm-egg fusion protein 2-like | |
| rno-miR-127-5p | up | ENSRNOT00000071503 | ENSRNOG00000047254 | LOC100911976 | izumo sperm-egg fusion protein 2-like | |
| rno-miR-370-3p | up | ENSRNOT00000047682 | ENSRNOG00000033734 | Tnnt2 | troponin T2, cardiac type | |
| rno-miR-370-3p | up | ENSRNOT00000077020 | ENSRNOG00000014030 | Synm | synemin | |
| rno-miR-370-3p | up | ENSRNOT00000027995 | ENSRNOG00000020607 | Bckdha | branched chain ketoacid dehydrogenase E1, alpha polypeptide | |
| rno-miR-665 | up | ENSRNOT00000080422 | ENSRNOG00000051483 | LOC103689961 | selenoprotein W-like | |
| rno-miR-370-3p | up | ENSRNOT00000074839 | ENSRNOG00000049757 | LOC689039 | hypothetical protein LOC689039 | |
| rno-miR-431 | up | ENSRNOT00000060300 | ENSRNOG00000050964 | LOC100911068 | roundabout homolog 4-like | |
| rno-miR-665 | up | ENSRNOT00000084085 | ENSRNOG00000009712 | Gale | UDP-galactose-4-epimerase | |
| rno-miR-214-3p | up | ENSRNOT00000012943 | ENSRNOG00000032332 | Krt4 | keratin 4 | |
| rno-miR-673-3p | up | ENSRNOT00000071373 | ENSRNOG00000046443 | Mthfsd | methenyltetrahydrofolate synthetase domain containing | |
| rno-miR-665 | up | ENSRNOT00000065673 | ENSRNOG00000042230 | RGD1307182 | similar to RIKEN cDNA B430306N03 gene | |
| rno-miR-370-3p | up | ENSRNOT00000092976 | ENSRNOG00000017030 | Arid1b | AT-rich interaction domain 1B | |
| rno-miR-370-3p | up | ENSRNOT00000084454 | ENSRNOG00000017030 | Arid1b | AT-rich interaction domain 1B | |
| rno-miR-370-3p | up | ENSRNOT00000022939 | ENSRNOG00000017030 | Arid1b | AT-rich interaction domain 1B | |
| rno-miR-665 | up | ENSRNOT00000026471 | ENSRNOG00000019509 | Fbxl15 | F-box and leucine-rich repeat protein 15 | |
| rno-miR-485-5p | up | ENSRNOT00000003092 | ENSRNOG00000002265 | Casr | calcium-sensing receptor | |
| rno-miR-370-3p | up | ENSRNOT00000090080 | ENSRNOG00000057125 | Ddr1 | discoidin domain receptor tyrosine kinase 1 | |
| rno-miR-485-5p | up | ENSRNOT00000078341 | ENSRNOG00000002265 | Casr | calcium-sensing receptor | |
| rno-miR-214-3p | up | ENSRNOT00000012187 | ENSRNOG00000009191 | Ccm2l | CCM2 like scaffolding protein | |
| rno-miR-370-3p | up | ENSRNOT00000051452 | ENSRNOG00000015695 | Sult5a1 | sulfotransferase family 5A, member 1 | |
| rno-miR-370-3p | up | ENSRNOT00000012373 | ENSRNOG00000009343 | Evpl | envoplakin | |
| rno-miR-127-3p | up | ENSRNOT00000023247 | ENSRNOG00000017225 | Fam160b1 | family with sequence similarity 160, member B1 | |
| rno-miR-665 | up | ENSRNOT00000077074 | ENSRNOG00000055433 | Trim46 | tripartite motif-containing 46 | |
| rno-miR-409a-3p | up | ENSRNOT00000092612 | ENSRNOG00000025269 | Slc25a44 | solute carrier family 25, member 44 | |
| rno-miR-665 | up | ENSRNOT00000050837 | ENSRNOG00000033165 | LOC100363867 | LRRGT00106-like | |
| rno-miR-370-3p | up | ENSRNOT00000023145 | ENSRNOG00000042879 | Cep192 | centrosomal protein 192 | |
| rno-miR-665 | up | ENSRNOT00000067612 | ENSRNOG00000011599 | Gldc | glycine decarboxylase | |
| rno-miR-665 | up | ENSRNOT00000018122 | ENSRNOG00000013201 | Tex264 | testis expressed 264 | |
| rno-miR-370-3p | up | ENSRNOT00000077465 | ENSRNOG00000033624 | Zfp426 | zinc finger protein 426 | |
| rno-miR-665 | up | ENSRNOT00000012080 | ENSRNOG00000008911 | Draxin | dorsal inhibitory axon guidance protein | |
| rno-miR-127-3p | up | ENSRNOT00000031786 | ENSRNOG00000028628 | LOC100911196 | zinc finger protein 865-like | |
| rno-miR-214-3p | up | ENSRNOT00000072402 | ENSRNOG00000046850 | NEWGENE_621438 | thrombopoietin | |
| rno-miR-370-3p | up | ENSRNOT00000020497 | ENSRNOG00000015184 | Clic3 | chloride intracellular channel 3 | |
| rno-miR-214-3p | up | ENSRNOT00000038888 | ENSRNOG00000028083 | Tmem35b | transmembrane protein 35B | |
| rno-miR-214-3p | up | ENSRNOT00000057372 | ENSRNOG00000037795 | Ldlrad1 | low density lipoprotein receptor class A domain containing 1 | |
| rno-miR-665 | up | ENSRNOT00000077428 | ENSRNOG00000054513 | Zmiz2 | zinc finger, MIZ-type containing 2 | |
| rno-miR-665 | up | ENSRNOT00000012327 | ENSRNOG00000008781 | RGD1310769 | similar to HSPC288 | |
| rno-miR-370-3p | up | ENSRNOT00000080068 | ENSRNOG00000001422 | Col26a1 | collagen type XXVI alpha 1 chain | |
| rno-miR-665 | up | ENSRNOT00000027550 | ENSRNOG00000020280 | Armc6 | armadillo repeat containing 6 | |
| rno-miR-127-3p | up | ENSRNOT00000010241 | ENSRNOG00000007304 | Herc3 | HECT and RLD domain containing E3 ubiquitin protein ligase 3 | |
| rno-miR-370-3p | up | ENSRNOT00000080695 | ENSRNOG00000016818 | Fgfr3 | fibroblast growth factor receptor 3 | |
| rno-miR-370-3p | up | ENSRNOT00000031448 | ENSRNOG00000017346 | Myt1 | myelin transcription factor 1 | |
| rno-miR-665 | up | ENSRNOT00000024022 | ENSRNOG00000017791 | Arhgap12 | Rho GTPase activating protein 12 | |
| rno-miR-673-3p | up | ENSRNOT00000073209 | ENSRNOG00000046639 | Itgae | integrin subunit alpha E | |
| rno-miR-370-3p | up | ENSRNOT00000087462 | ENSRNOG00000053311 | AABR07065139.1 |  | |
| rno-miR-370-3p | up | ENSRNOT00000064251 | ENSRNOG00000042668 | Msrb3 | methionine sulfoxide reductase B3 | |
| rno-miR-370-3p | up | ENSRNOT00000085635 | ENSRNOG00000057125 | Ddr1 | discoidin domain receptor tyrosine kinase 1 | |
| rno-miR-665 | up | ENSRNOT00000013322 | ENSRNOG00000009712 | Gale | UDP-galactose-4-epimerase | |
| rno-miR-370-3p | up | ENSRNOT00000080778 | ENSRNOG00000025071 | Qrich1 | glutamine-rich 1 | |
| rno-miR-212-5p | up | ENSRNOT00000015521 | ENSRNOG00000011352 | Furin | furin (paired basic amino acid cleaving enzyme) | |
| rno-miR-370-3p | up | ENSRNOT00000003686 | ENSRNOG00000002728 | Btc | betacellulin | |
| rno-miR-673-3p | up | ENSRNOT00000026114 | ENSRNOG00000019310 | Eid2 | EP300 interacting inhibitor of differentiation 2 | |
| rno-miR-370-3p | up | ENSRNOT00000087254 | ENSRNOG00000017132 | Snx30 | sorting nexin family member 30 | |
| rno-miR-370-3p | up | ENSRNOT00000090346 | ENSRNOG00000042458 | Stau2 | staufen double-stranded RNA binding protein 2 | |
| rno-miR-485-5p | up | ENSRNOT00000009513 | ENSRNOG00000007254 | Ttc9 | tetratricopeptide repeat domain 9 | |
| rno-miR-370-3p | up | ENSRNOT00000078834 | ENSRNOG00000052963 | Brox | BRO1 domain and CAAX motif containing | |
| rno-miR-370-3p | up | ENSRNOT00000014207 | ENSRNOG00000010473 | Cand2 | cullin-associated and neddylation-dissociated 2 (putative) | |
| rno-miR-665 | up | ENSRNOT00000033737 | ENSRNOG00000024591 | Hs3st3a1 | heparan sulfate-glucosamine 3-sulfotransferase 3A1 | |
| rno-miR-341 | up | ENSRNOT00000011178 | ENSRNOG00000007970 | Plxnc1 | plexin C1 | |
| rno-miR-214-3p | up | ENSRNOT00000082264 | ENSRNOG00000032332 | Krt4 | keratin 4 | |
| rno-miR-341 | up | ENSRNOT00000060971 | ENSRNOG00000039743 | Zdhhc11 | zinc finger, DHHC-type containing 11 | |
| rno-miR-127-3p | up | ENSRNOT00000026742 | ENSRNOG00000019727 | Dph3 | diphthamide biosynthesis 3 | |
| rno-miR-665 | up | ENSRNOT00000073991 | ENSRNOG00000047247 | Ptprs | protein tyrosine phosphatase, receptor type, S | |
| rno-miR-665 | up | ENSRNOT00000074469 | ENSRNOG00000047247 | Ptprs | protein tyrosine phosphatase, receptor type, S | |
| rno-miR-665 | up | ENSRNOT00000079999 | ENSRNOG00000047247 | Ptprs | protein tyrosine phosphatase, receptor type, S | |
| rno-miR-212-5p | up | ENSRNOT00000017684 | ENSRNOG00000013165 | Bet1l | Bet1 golgi vesicular membrane trafficking protein-like | |
| rno-miR-665 | up | ENSRNOT00000077604 | ENSRNOG00000058249 | Pgk1 | phosphoglycerate kinase 1 | |
| rno-miR-673-3p | up | ENSRNOT00000077598 | ENSRNOG00000010888 | Ankrd33b | ankyrin repeat domain 33B | |
| rno-miR-673-3p | up | ENSRNOT00000092136 | ENSRNOG00000048036 | Ncan | neurocan | |
| rno-miR-673-3p | up | ENSRNOT00000000475 | ENSRNOG00000026407 | Fam184a | family with sequence similarity 184, member A | |
| rno-miR-673-3p | up | ENSRNOT00000008391 | ENSRNOG00000006341 | Saysd1 | SAYSVFN motif domain containing 1 | |
| rno-miR-370-3p | up | ENSRNOT00000045855 | ENSRNOG00000023943 | Gbp1 | guanylate binding protein 1 | |
| rno-miR-370-3p | up | ENSRNOT00000020357 | ENSRNOG00000015093 | Sparcl1 | SPARC like 1 | |
| rno-miR-665 | up | ENSRNOT00000084933 | ENSRNOG00000046214 | Cyp27b1 | cytochrome P450, family 27, subfamily b, polypeptide 1 | |
| rno-miR-665 | up | ENSRNOT00000002396 | ENSRNOG00000001757 | Tm4sf19 | transmembrane 4 L six family member 19 | |
| rno-miR-370-3p | up | ENSRNOT00000044983 | ENSRNOG00000029658 | Rnf213 | ring finger protein 213 | |
| rno-miR-370-3p | up | ENSRNOT00000059419 | ENSRNOG00000017132 | Snx30 | sorting nexin family member 30 | |
| rno-miR-673-3p | up | ENSRNOT00000004923 | ENSRNOG00000003704 | Fam184b | family with sequence similarity 184, member B | |
| rno-miR-370-3p | up | ENSRNOT00000040856 | ENSRNOG00000036885 | Gbp3 | guanylate binding protein 3 | |
| rno-miR-127-5p | up | ENSRNOT00000028097 | ENSRNOG00000020700 | Rnaseh2c | ribonuclease H2, subunit C | |
| rno-miR-665 | up | ENSRNOT00000012162 | ENSRNOG00000009005 | Slco2a1 | solute carrier organic anion transporter family, member 2a1 | |
| rno-miR-370-3p | up | ENSRNOT00000041938 | ENSRNOG00000031099 | Tmprss12 | transmembrane protease, serine 12 | |
| rno-miR-370-3p | up | ENSRNOT00000013262 | ENSRNOG00000009538 | Etfdh | electron transfer flavoprotein dehydrogenase | |
| rno-miR-370-3p | up | ENSRNOT00000074616 | ENSRNOG00000050794 | Pdlim4 | PDZ and LIM domain 4 | |
| rno-miR-370-3p | up | ENSRNOT00000033413 | ENSRNOG00000012508 | Slc39a8 | solute carrier family 39 member 8 | |
| rno-miR-127-3p | up | ENSRNOT00000035886 | ENSRNOG00000021987 | Tnfrsf17 | TNF receptor superfamily member 17 | |
| rno-miR-370-3p | up | ENSRNOT00000018460 | ENSRNOG00000012798 | Tubgcp4 | tubulin, gamma complex associated protein 4 | |
| rno-miR-370-3p | up | ENSRNOT00000083935 | ENSRNOG00000036833 | Zfp385a | zinc finger protein 385A | |
| rno-miR-370-3p | up | ENSRNOT00000005021 | ENSRNOG00000003783 | Fthl17e | ferritin, heavy polypeptide-like 17, member E | |
| rno-miR-341 | up | ENSRNOT00000020950 | ENSRNOG00000015623 | Guca1b | guanylate cyclase activator 1B | |
| rno-miR-673-3p | up | ENSRNOT00000077581 | ENSRNOG00000009594 | Snai1 | snail family transcriptional repressor 1 | |
| rno-miR-665 | up | ENSRNOT00000020005 | ENSRNOG00000014753 | Tcf7l1 | transcription factor 7 like 1 | |
| rno-miR-370-3p | up | ENSRNOT00000090278 | ENSRNOG00000004000 | Tefm | transcription elongation factor, mitochondrial | |
| rno-miR-665 | up | ENSRNOT00000072176 | ENSRNOG00000046214 | Cyp27b1 | cytochrome P450, family 27, subfamily b, polypeptide 1 | |
| rno-miR-370-3p | up | ENSRNOT00000078202 | ENSRNOG00000061695 | Slc23a1 | solute carrier family 23 member 1 | |
| rno-miR-665 | up | ENSRNOT00000076079 | ENSRNOG00000002767 | Dlg3 | discs large MAGUK scaffold protein 3 | |
| rno-miR-673-3p | up | ENSRNOT00000077107 | ENSRNOG00000046639 | Itgae | integrin subunit alpha E | |
| rno-miR-370-3p | up | ENSRNOT00000088495 | ENSRNOG00000046202 | Metrnl | meteorin-like, glial cell differentiation regulator | |
| rno-miR-212-5p | up | ENSRNOT00000016324 | ENSRNOG00000012210 | Sptlc2 | serine palmitoyltransferase, long chain base subunit 2 | |
| rno-miR-665 | up | ENSRNOT00000081195 | ENSRNOG00000061572 | Smarcd1 | SWI/SNF related, matrix associated, actin dependent regulator of chromatin, subfamily d, member 1 | |
| rno-miR-665 | up | ENSRNOT00000003741 | ENSRNOG00000002767 | Dlg3 | discs large MAGUK scaffold protein 3 | |
| rno-miR-214-3p | up | ENSRNOT00000040981 | ENSRNOG00000029238 | Hgh1 | HGH1 homolog | |
| rno-miR-665 | up | ENSRNOT00000045082 | ENSRNOG00000002767 | Dlg3 | discs large MAGUK scaffold protein 3 | |
| rno-miR-370-3p | up | ENSRNOT00000080031 | ENSRNOG00000016135 | Fars2 | phenylalanyl-tRNA synthetase 2, mitochondrial | |
| rno-miR-485-5p | up | ENSRNOT00000093103 | ENSRNOG00000012634 | Fbxo10 | F-box protein 10 | |
| rno-miR-665 | up | ENSRNOT00000063938 | ENSRNOG00000042269 | Gpr21 | G protein-coupled receptor 21 | |
| rno-miR-665 | up | ENSRNOT00000082624 | ENSRNOG00000056184 | Lrp2 | LDL receptor related protein 2 | |
| rno-miR-370-3p | up | ENSRNOT00000055615 | ENSRNOG00000011076 | Ank2 | ankyrin 2 | |
| rno-miR-370-3p | up | ENSRNOT00000015386 | ENSRNOG00000011076 | Ank2 | ankyrin 2 | |
| rno-miR-370-3p | up | ENSRNOT00000068415 | ENSRNOG00000011076 | Ank2 | ankyrin 2 | |
| rno-miR-214-3p | up | ENSRNOT00000087443 | ENSRNOG00000061316 | LOC102547056 | retinoic acid early-inducible protein 1-gamma-like | |
| rno-miR-132-5p | up | ENSRNOT00000088898 | ENSRNOG00000036692 | Gcgr | glucagon receptor | |
| rno-miR-370-3p | up | ENSRNOT00000014687 | ENSRNOG00000029814 | Prtn3 | proteinase 3 | |
| rno-miR-665 | up | ENSRNOT00000039909 | ENSRNOG00000031335 | Ankrd37 | ankyrin repeat domain 37 | |
| rno-miR-370-3p | up | ENSRNOT00000008254 | ENSRNOG00000006236 | Dsn1 | DSN1 homolog, MIS12 kinetochore complex component | |
| rno-miR-370-3p | up | ENSRNOT00000042372 | ENSRNOG00000008755 | Acox1 | acyl-CoA oxidase 1 | |
| rno-miR-665 | up | ENSRNOT00000024547 | ENSRNOG00000018217 | Syt5 | synaptotagmin 5 | |
| rno-miR-382-5p | up | ENSRNOT00000074598 | ENSRNOG00000058822 | Vom2r65 | vomeronasal 2 receptor, 65 | |
| rno-miR-382-5p | up | ENSRNOT00000073032 | ENSRNOG00000060785 | Vom2r65 | vomeronasal 2 receptor, 65 | |
| rno-miR-382-5p | up | ENSRNOT00000033032 | ENSRNOG00000047177 | Vom2r65 | vomeronasal 2 receptor, 65 | |
| rno-miR-382-5p | up | ENSRNOT00000050916 | ENSRNOG00000049837 | Vom2r65 | vomeronasal 2 receptor, 65 | |
| rno-miR-382-5p | up | ENSRNOT00000034630 | ENSRNOG00000046767 | AABR07030977.1 |  | |
| rno-miR-382-5p | up | ENSRNOT00000049921 | ENSRNOG00000021490 | AABR07069997.1 |  | |
| rno-miR-382-5p | up | ENSRNOT00000070942 | ENSRNOG00000047388 | LOC100911926 | vomeronasal type-2 receptor 26-like | |
| rno-miR-673-3p | up | ENSRNOT00000013370 | ENSRNOG00000009950 | Msantd2 | Myb/SANT-like DNA-binding domain containing 2 | |
| rno-miR-665 | up | ENSRNOT00000017624 | ENSRNOG00000012802 | Tenm3 | teneurin transmembrane protein 3 | |
| rno-miR-370-3p | up | ENSRNOT00000080637 | ENSRNOG00000011076 | Ank2 | ankyrin 2 | |
| rno-miR-370-3p | up | ENSRNOT00000037255 | ENSRNOG00000011076 | Ank2 | ankyrin 2 | |
| rno-miR-370-3p | up | ENSRNOT00000074002 | ENSRNOG00000011076 | Ank2 | ankyrin 2 | |
| rno-miR-370-3p | up | ENSRNOT00000022380 | ENSRNOG00000016703 | Gtf2a1l | general transcription factor 2A subunit 1 like | |
| rno-miR-382-5p | up | ENSRNOT00000073546 | ENSRNOG00000050370 | Vom2r6 | vomeronasal 2 receptor, 6 | |
| rno-miR-382-5p | up | ENSRNOT00000073973 | ENSRNOG00000050954 |  |  | |
| rno-miR-127-3p | up | ENSRNOT00000045855 | ENSRNOG00000023943 | Gbp1 | guanylate binding protein 1 [Source:RGD Symbol;Acc:1311877] | |
| rno-miR-665 | up | ENSRNOT00000079254 | ENSRNOG00000010094 | Kcnmb2 | potassium calcium-activated channel subfamily M regulatory beta subunit 2 | |
| rno-miR-214-3p | up | ENSRNOT00000077911 | ENSRNOG00000054917 | Mrgpre | MAS related GPR family member E | |
| rno-miR-673-3p | up | ENSRNOT00000004118 | ENSRNOG00000003085 | Mycs | myc-like oncogene, s-myc protein | |
| rno-miR-665 | up | ENSRNOT00000025853 | ENSRNOG00000019097 | Bap1 | Brca1 associated protein 1 | |
| rno-miR-370-3p | up | ENSRNOT00000089168 | ENSRNOG00000017144 | Mea1 | male-enhanced antigen 1 | |
| rno-novel-58-mature | up | ENSRNOT00000006141 | ENSRNOG00000004414 | F11r | F11 receptor | |
| rno-miR-214-3p | up | ENSRNOT00000088870 | ENSRNOG00000012811 | Spint1 | serine peptidase inhibitor, Kunitz type 1 | |
| rno-miR-370-3p | up | ENSRNOT00000037133 | ENSRNOG00000024808 | Stk39 | serine threonine kinase 39 | |
| rno-miR-665 | up | ENSRNOT00000020483 | ENSRNOG00000015264 | Lrrc38 | leucine rich repeat containing 38 | |
| rno-miR-212-5p | up | ENSRNOT00000015790 | ENSRNOG00000011839 | Mrps31 | mitochondrial ribosomal protein S31 | |
| rno-miR-665 | up | ENSRNOT00000024969 | ENSRNOG00000018877 | Zfp689 | zinc finger protein 689 | |
| rno-miR-673-3p | up | ENSRNOT00000026286 | ENSRNOG00000019413 | Atg16l2 | autophagy related 16-like 2 | |
| rno-miR-665 | up | ENSRNOT00000013105 | ENSRNOG00000009531 | Col9a3 | collagen type IX alpha 3 chain | |
| rno-miR-665 | up | ENSRNOT00000054887 | ENSRNOG00000017748 | Nkx6-2 | NK6 homeobox 2 | |
| rno-miR-370-3p | up | ENSRNOT00000086554 | ENSRNOG00000036833 | Zfp385a | zinc finger protein 385A | |
| rno-miR-127-3p | up | ENSRNOT00000021549 | ENSRNOG00000015686 | Col4a3bp | collagen type IV alpha 3 binding protein | |
| rno-miR-132-5p | up | ENSRNOT00000083601 | ENSRNOG00000036692 | Gcgr | glucagon receptor | |
| rno-novel-58-mature | up | ENSRNOT00000088316 | ENSRNOG00000009105 | Kb15 | type II keratin Kb15 | |
| rno-miR-127-5p | up | ENSRNOT00000084613 | ENSRNOG00000014981 | Usp14 | ubiquitin specific peptidase 14 | |
| rno-miR-370-3p | up | ENSRNOT00000083223 | ENSRNOG00000057125 | Ddr1 | discoidin domain receptor tyrosine kinase 1 ] | |
| rno-miR-370-3p | up | ENSRNOT00000031362 | ENSRNOG00000023480 | Nabp2 | nucleic acid binding protein 2 | |
| rno-miR-665 | up | ENSRNOT00000013786 | ENSRNOG00000010375 | Slc39a2 | solute carrier family 39 member 2 | |
| rno-miR-132-5p | up | ENSRNOT00000054962 | ENSRNOG00000036692 | Gcgr | glucagon receptor | |
| rno-miR-370-3p | up | ENSRNOT00000003728 | ENSRNOG00000002781 | Kdsr | 3-ketodihydrosphingosine reductase | |
| rno-miR-370-3p | up | ENSRNOT00000013165 | ENSRNOG00000009271 | Smarca4 | SWI/SNF related, matrix associated, actin dependent regulator of chromatin, subfamily a, member 4 | |
| rno-miR-665 | up | ENSRNOT00000071719 | ENSRNOG00000048747 | Cdk5rap3 | CDK5 regulatory subunit associated protein 3 | |
| rno-miR-665 | up | ENSRNOT00000025933 | ENSRNOG00000019080 | Hsd3b7 | hydroxy-delta-5-steroid dehydrogenase, 3 beta- and steroid delta-isomerase 7 | |
| rno-miR-665 | up | ENSRNOT00000091497 | ENSRNOG00000055798 | LOC103689958 | olfactory receptor 49-like | |
| rno-miR-370-3p | up | ENSRNOT00000022190 | ENSRNOG00000016378 | Map3k8 | mitogen-activated protein kinase kinase kinase 8 | |
| rno-miR-665 | up | ENSRNOT00000087016 | ENSRNOG00000053124 | Olr1l | olfactory receptor 1-like | |
| rno-miR-665 | up | ENSRNOT00000084162 | ENSRNOG00000056884 | Olr386 | olfactory receptor 386 | |
| rno-miR-665 | up | ENSRNOT00000004070 | ENSRNOG00000003020 | Slc25a47 | solute carrier family 25, member 47 | |
| rno-miR-370-3p | up | ENSRNOT00000065529 | ENSRNOG00000011044 | Clmn | calmin | |
| rno-miR-214-3p | up | ENSRNOT00000017223 | ENSRNOG00000012811 | Spint1 | serine peptidase inhibitor, Kunitz type 1 | |
| rno-miR-665 | up | ENSRNOT00000025624 | ENSRNOG00000018870 | Hapln2 | hyaluronan and proteoglycan link protein 2 | |
| rno-miR-485-5p | up | ENSRNOT00000008126 | ENSRNOG00000006204 | Slc30a3 | solute carrier family 30 member 3 | |
| rno-miR-370-3p | up | ENSRNOT00000014217 | ENSRNOG00000010473 | Cand2 | cullin-associated and neddylation-dissociated 2 (putative) | |
| rno-miR-665 | up | ENSRNOT00000078512 | ENSRNOG00000058538 | AABR07002001.1 |  | |
| rno-miR-511-5p | up | ENSRNOT00000003901 | ENSRNOG00000002881 | Ddr2 | discoidin domain receptor tyrosine kinase 2 | |
| rno-miR-370-3p | up | ENSRNOT00000011994 | ENSRNOG00000039544 | Kcnd1 | potassium voltage-gated channel subfamily D member 1 | |
| rno-miR-665 | up | ENSRNOT00000077327 | ENSRNOG00000058422 | Lilrb3l | leukocyte immunoglobulin-like receptor, subfamily B (with TM and ITIM domains), member 3-like | |
| rno-miR-370-3p | up | ENSRNOT00000019415 | ENSRNOG00000014378 | Il1r2 | interleukin 1 receptor type 2 | |
| rno-miR-382-5p | up | ENSRNOT00000093436 | ENSRNOG00000062307 | Vom2r3 |  | |
| rno-miR-337-5p | up | ENSRNOT00000010558 | ENSRNOG00000007968 | RGD1305350 | similar to RIKEN cDNA 2510039O18 | |
| rno-miR-665 | up | ENSRNOT00000087669 | ENSRNOG00000053260 | Lilrb3a | leukocyte immunoglobulin-like receptor, subfamily B (with TM and ITIM domains), member 3A | |
| rno-miR-673-3p | up | ENSRNOT00000001399 | ENSRNOG00000001057 | Ctxn1 | cortexin 1 | |
| rno-miR-127-3p | up | ENSRNOT00000066618 | ENSRNOG00000006435 | Tor1b | torsin family 1, member B | |
| rno-miR-665 | up | ENSRNOT00000073999 | ENSRNOG00000050844 | Fibcd1l1 | fibrinogen C domain containing 1-like 1 | |
| rno-miR-485-5p | up | ENSRNOT00000093087 | ENSRNOG00000012634 | Fbxo10 | F-box protein 10 | |
| rno-miR-127-3p | up | ENSRNOT00000004440 | ENSRNOG00000003296 | Dck | deoxycytidine kinase | |
| rno-miR-665 | up | ENSRNOT00000080799 | ENSRNOG00000053260 | Lilrb3a | leukocyte immunoglobulin-like receptor, subfamily B (with TM and ITIM domains), member 3A | |
| rno-miR-665 | up | ENSRNOT00000080820 | ENSRNOG00000053260 | Lilrb3a | leukocyte immunoglobulin-like receptor, subfamily B (with TM and ITIM domains), member 3A | |
| rno-miR-370-3p | up | ENSRNOT00000093726 | ENSRNOG00000000040 | RGD1304622 | similar to 6820428L09 protein | |
| rno-miR-370-3p | up | ENSRNOT00000016148 | ENSRNOG00000011885 | Rhpn2 | rhophilin, Rho GTPase binding protein 2 | |
| rno-miR-370-3p | up | ENSRNOT00000056815 | ENSRNOG00000037518 | Hyi | hydroxypyruvate isomerase | |
| rno-miR-370-3p | up | ENSRNOT00000083674 | ENSRNOG00000060266 | Exoc2 | exocyst complex component 2 | |
| rno-miR-665 | up | ENSRNOT00000028034 | ENSRNOG00000020653 | S1pr2 | sphingosine-1-phosphate receptor 2 | |
| rno-miR-485-5p | up | ENSRNOT00000086097 | ENSRNOG00000002265 | Casr | calcium-sensing receptor | |
| rno-miR-214-3p | up | ENSRNOT00000018453 | ENSRNOG00000013468 | Fam213b | family with sequence similarity 213, member B | |
| rno-miR-665 | up | ENSRNOT00000001248 | ENSRNOG00000000940 | Flt1 | FMS-related tyrosine kinase 1 | |
| rno-miR-370-3p | up | ENSRNOT00000049857 | ENSRNOG00000006961 | Snrpb | small nuclear ribonucleoprotein polypeptides B and B1 | |
| rno-miR-127-5p | up | ENSRNOT00000025046 | ENSRNOG00000018520 | Nsun6 | NOP2/Sun RNA methyltransferase family member 6 | |
| rno-miR-370-3p | up | ENSRNOT00000089534 | ENSRNOG00000011076 | Ank2 | ankyrin 2 | |
| rno-miR-370-3p | up | ENSRNOT00000080470 | ENSRNOG00000011076 | Ank2 | ankyrin 2 | |
| rno-miR-370-3p | up | ENSRNOT00000084756 | ENSRNOG00000011076 | Ank2 | ankyrin 2 | |
| rno-miR-370-3p | up | ENSRNOT00000091273 | ENSRNOG00000007492 | Rpn2 | ribophorin II | |
| rno-miR-370-3p | up | ENSRNOT00000001486 | ENSRNOG00000001124 | Rnft2 | ring finger protein, transmembrane 2 | |
| rno-miR-370-3p | up | ENSRNOT00000002568 | ENSRNOG00000001877 | Med15 | mediator complex subunit 15 | |
| rno-miR-370-3p | up | ENSRNOT00000083415 | ENSRNOG00000061814 | Dgcr2 | DiGeorge syndrome critical region gene 2 | |
| rno-miR-665 | up | ENSRNOT00000091275 | ENSRNOG00000009005 | Slco2a1 | solute carrier organic anion transporter family, member 2a1 | |
| rno-miR-665 | up | ENSRNOT00000087432 | ENSRNOG00000019372 | Pc | pyruvate carboxylase | |
| rno-miR-214-3p | up | ENSRNOT00000025045 | ENSRNOG00000018740 | Ugt1a5 | UDP glucuronosyltransferase family 1 member A5 | |
| rno-miR-370-3p | up | ENSRNOT00000091602 | ENSRNOG00000060460 | Clec1a | C-type lectin domain family 1, member A | |
| rno-miR-127-3p | up | ENSRNOT00000058423 | ENSRNOG00000038372 | Ndufs2 | NADH dehydrogenase (ubiquinone) Fe-S protein 2 | |
| rno-miR-665 | up | ENSRNOT00000023659 | ENSRNOG00000017601 | Srd5a1 | steroid 5 alpha-reductase 1 | |
| rno-miR-673-3p | up | ENSRNOT00000012939 | ENSRNOG00000009594 | Snai1 | snail family transcriptional repressor 1 | |
| rno-miR-493-3p | up | ENSRNOT00000015090 | ENSRNOG00000011338 | Cecr5 | cat eye syndrome chromosome region, candidate 5 | |
| rno-miR-665 | up | ENSRNOT00000032376 | ENSRNOG00000010899 | Bfsp2 | beaded filament structural protein 2 | |
| rno-miR-665 | up | ENSRNOT00000081117 | ENSRNOG00000051399 | Col10a1 | collagen type X alpha 1 chain | |
| rno-miR-370-3p | up | ENSRNOT00000020405 | ENSRNOG00000015179 | Tradd | TNFRSF1A-associated via death domain | |
| rno-miR-370-3p | up | ENSRNOT00000011270 | ENSRNOG00000008517 | Cdc42ep1 | CDC42 effector protein 1 | |
| rno-miR-673-3p | up | ENSRNOT00000029885 | ENSRNOG00000021637 | Ddx46 | DEAD-box helicase 46 | |
| rno-miR-665 | up | ENSRNOT00000025725 | ENSRNOG00000019019 | Fitm1 | fat storage-inducing transmembrane protein 1 | |
| rno-miR-127-3p | up | ENSRNOT00000005262 | ENSRNOG00000003873 | Cpd | carboxypeptidase D | |
| rno-miR-370-3p | up | ENSRNOT00000086244 | ENSRNOG00000001074 | Mphosph9 | M-phase phosphoprotein 9 | |
| rno-miR-665 | up | ENSRNOT00000043344 | ENSRNOG00000049289 | LOC100911068 | roundabout homolog 4-like | |
| rno-miR-127-3p | up | ENSRNOT00000028891 | ENSRNOG00000021268 | LOC681292 | hypothetical protein LOC681292 | |
| rno-miR-370-3p | up | ENSRNOT00000030170 | ENSRNOG00000023094 | RGD1561661 | similar to Ferritin light chain (Ferritin L subunit) | |
| rno-miR-665 | up | ENSRNOT00000089560 | ENSRNOG00000005882 | Tle1 | transducin like enhancer of split 1 | |
| rno-miR-511-5p | up | ENSRNOT00000041533 | ENSRNOG00000018712 | Camk2a | calcium/calmodulin-dependent protein kinase II alpha | |
| rno-miR-370-3p | up | ENSRNOT00000022065 | ENSRNOG00000016390 | Eef1e1 | eukaryotic translation elongation factor 1 epsilon 1 | |
| rno-miR-665 | up | ENSRNOT00000020864 | ENSRNOG00000015152 | Pkp3 | plakophilin 3 | |
| rno-miR-214-3p | up | ENSRNOT00000086944 | ENSRNOG00000014629 | Lmo1 | LIM domain only 1 | |
| rno-novel-58-mature | up | ENSRNOT00000035312 | ENSRNOG00000023455 | Pomgnt1 | protein O-linked mannose N-acetylglucosaminyltransferase 1 (beta 1,2-) | |
| rno-miR-370-3p | up | ENSRNOT00000025363 | ENSRNOG00000018764 | B3gnt3 | UDP-GlcNAc:betaGal beta-1,3-N-acetylglucosaminyltransferase 3 | |
| rno-miR-370-3p | up | ENSRNOT00000086660 | ENSRNOG00000010473 | Cand2 | cullin-associated and neddylation-dissociated 2 (putative) | |
| rno-miR-127-5p | up | ENSRNOT00000020537 | ENSRNOG00000014981 | Usp14 | ubiquitin specific peptidase 14 | |
| rno-miR-212-5p | up | ENSRNOT00000084455 | ENSRNOG00000019638 | Lmna | lamin A/C | |
| rno-miR-485-5p | up | ENSRNOT00000000649 | ENSRNOG00000000540 | Btbd9 | BTB domain containing 9 | |
| rno-miR-370-3p | up | ENSRNOT00000025958 | ENSRNOG00000018543 | Atp4b | ATPase H+/K+ transporting beta subunit [Source:RGD Symbol;Acc:2178] | |
| rno-miR-370-3p | up | ENSRNOT00000028324 | ENSRNOG00000020875 | Celf3 | CUGBP, Elav-like family member 3 | |
| rno-miR-665 | up | ENSRNOT00000024079 | ENSRNOG00000017657 | Phf23 | PHD finger protein 23 | |
| rno-miR-370-3p | up | ENSRNOT00000014962 | ENSRNOG00000011076 | Ank2 | ankyrin 2 | |
| rno-miR-370-3p | up | ENSRNOT00000007634 | ENSRNOG00000005767 | Tulp3 | tubby-like protein 3 | |
| rno-miR-370-3p | up | ENSRNOT00000067162 | ENSRNOG00000052080 | Camk2b | calcium/calmodulin-dependent protein kinase II beta | |
| rno-miR-665 | up | ENSRNOT00000060300 | ENSRNOG00000050964 | LOC100911068 | roundabout homolog 4-like | |
| rno-miR-370-3p | up | ENSRNOT00000000735 | ENSRNOG00000000597 | Wisp3 | WNT1 inducible signaling pathway protein 3 | |
| rno-novel-58-mature | up | ENSRNOT00000016137 | ENSRNOG00000010474 | Hmces | 5-hydroxymethylcytosine (hmC) binding, ES cell-specific | |
| rno-miR-665 | up | ENSRNOT00000028271 | ENSRNOG00000020812 | Gys1 | glycogen synthase 1 | |
| rno-miR-212-5p | up | ENSRNOT00000006191 | ENSRNOG00000000504 | Fance | Fanconi anemia, complementation group E | |
| rno-miR-127-5p | up | ENSRNOT00000018009 | ENSRNOG00000013360 | Prkcsh | protein kinase C substrate 80K-H | |
| rno-miR-1193-3p | up | ENSRNOT00000022882 | ENSRNOG00000017022 | Cerk | ceramide kinase | |
| rno-miR-665 | up | ENSRNOT00000020280 | ENSRNOG00000014933 | Dnttip1 | deoxynucleotidyltransferase, terminal, interacting protein 1 | |
| rno-miR-673-3p | up | ENSRNOT00000005367 | ENSRNOG00000003969 | Fam20a | FAM20A, golgi associated secretory pathway pseudokinase | |
| rno-miR-485-5p | up | ENSRNOT00000018953 | ENSRNOG00000028064 | Fhad1 | forkhead associated phosphopeptide binding domain 1 | |
| rno-miR-212-5p | up | ENSRNOT00000085458 | ENSRNOG00000012210 | Sptlc2 | serine palmitoyltransferase, long chain base subunit 2 | |
| rno-miR-370-3p | up | ENSRNOT00000064261 | ENSRNOG00000036833 | Zfp385a | zinc finger protein 385A | |
| rno-miR-665 | up | ENSRNOT00000058906 | ENSRNOG00000005882 | Tle1 | transducin like enhancer of split 1 | |
| rno-miR-665 | up | ENSRNOT00000018232 | ENSRNOG00000013564 | Dok3 | docking protein 3 | |
| rno-miR-665 | up | ENSRNOT00000019781 | ENSRNOG00000014623 | Smurf2 | SMAD specific E3 ubiquitin protein ligase 2 | |
| rno-miR-370-3p | up | ENSRNOT00000074764 | ENSRNOG00000010680 | Med12l | mediator complex subunit 12-like | |
| rno-miR-140-3p | up | ENSRNOT00000024945 | ENSRNOG00000018497 | Olr48 | olfactory receptor 48 | |
| rno-miR-370-3p | up | ENSRNOT00000087026 | ENSRNOG00000018220 | Pde4dip | phosphodiesterase 4D interacting protein | |
| rno-miR-370-3p | up | ENSRNOT00000000890 | ENSRNOG00000000701 | Iscu | iron-sulfur cluster assembly enzyme | |
| rno-miR-370-3p | up | ENSRNOT00000013271 | ENSRNOG00000009184 | Foxp1 | forkhead box P1 | |
| rno-novel-58-mature | up | ENSRNOT00000082863 | ENSRNOG00000009563 | Krt2 | keratin 2 | |
| rno-miR-370-3p | up | ENSRNOT00000037415 | ENSRNOG00000024233 | RGD1559578 | RGD1559578 | |
| rno-miR-140-3p | up | ENSRNOT00000066674 | ENSRNOG00000002848 | Maoa | monoamine oxidase A | |
| rno-miR-370-3p | up | ENSRNOT00000012381 | ENSRNOG00000009332 | Rec114 | REC114 meiotic recombination protein | |
| rno-miR-665 | up | ENSRNOT00000013955 | ENSRNOG00000010320 | Efnb3 | ephrin B3 | |
| rno-miR-370-3p | up | ENSRNOT00000031973 | ENSRNOG00000024429 | Peg12 | paternally expressed 12 | |
| rno-miR-665 | up | ENSRNOT00000005953 | ENSRNOG00000004488 | Bdkrb1 | bradykinin receptor B1 | |
| rno-miR-665 | up | ENSRNOT00000002444 | ENSRNOG00000001794 | Muc13 | mucin 13, cell surface associated | |
| rno-miR-341 | up | ENSRNOT00000033068 | ENSRNOG00000014555 | Aox4 | aldehyde oxidase 4 | |
| rno-miR-341 | up | ENSRNOT00000092868 | ENSRNOG00000014555 | Aox4 | aldehyde oxidase 4 | |
| rno-miR-370-3p | up | ENSRNOT00000021070 | ENSRNOG00000015735 | Zswim1 | zinc finger, SWIM-type containing 1 [Source:RGD Symbol;Acc:1305715] | |
| rno-miR-370-3p | up | ENSRNOT00000081607 | ENSRNOG00000052080 | Camk2b | calcium/calmodulin-dependent protein kinase II beta | |
| rno-miR-370-3p | up | ENSRNOT00000085265 | ENSRNOG00000052080 | Camk2b | calcium/calmodulin-dependent protein kinase II beta | |
| rno-miR-665 | up | ENSRNOT00000022012 | ENSRNOG00000016148 | Gtse1 | G-2 and S-phase expressed 1 | |
| rno-miR-127-3p | up | ENSRNOT00000012512 | ENSRNOG00000032178 | Cenpa | centromere protein A | |
| rno-miR-127-5p | up | ENSRNOT00000089879 | ENSRNOG00000016516 | Mbp | myelin basic protein | |
| rno-miR-370-3p | up | ENSRNOT00000024922 | ENSRNOG00000018406 | Wipf1 | WAS/WASL interacting protein family, member 1 | |
| rno-miR-370-3p | up | ENSRNOT00000026654 | ENSRNOG00000019587 | Ptprn | protein tyrosine phosphatase, receptor type, N | |
| rno-miR-370-3p | up | ENSRNOT00000081377 | ENSRNOG00000017108 | Syngr1 | synaptogyrin 1 | |
| rno-miR-370-3p | up | ENSRNOT00000090637 | ENSRNOG00000008785 | Klf5 | Kruppel-like factor 5 | |
| rno-miR-665 | up | ENSRNOT00000021746 | ENSRNOG00000015724 | Gucy2g | guanylate cyclase 2G | |
| rno-miR-370-3p | up | ENSRNOT00000021660 | ENSRNOG00000016135 | Fars2 | phenylalanyl-tRNA synthetase 2, mitochondrial | |
| rno-miR-341 | up | ENSRNOT00000008941 | ENSRNOG00000006789 | Ddit3 | DNA-damage inducible transcript 3 | |
| rno-miR-370-3p | up | ENSRNOT00000059522 | ENSRNOG00000013603 | Dffa | DNA fragmentation factor subunit alpha | |
| rno-miR-433-3p | up | ENSRNOT00000077573 | ENSRNOG00000008552 | P2rx3 | purinergic receptor P2X 3 | |
| rno-miR-370-3p | up | ENSRNOT00000082336 | ENSRNOG00000010378 | Slc4a5 | solute carrier family 4 member 5 | |
| rno-miR-370-3p | up | ENSRNOT00000045107 | ENSRNOG00000029570 | Fam222b | family with sequence similarity 222, member B | |
| rno-miR-673-3p | up | ENSRNOT00000035463 | ENSRNOG00000010791 | Fam65c | family with sequence similarity 65, member C | |
| rno-miR-665 | up | ENSRNOT00000025559 | ENSRNOG00000018796 | Herpud1 | homocysteine inducible ER protein with ubiquitin like domain 1 | |
| rno-miR-485-5p | up | ENSRNOT00000016875 | ENSRNOG00000012634 | Fbxo10 | F-box protein 10 | |
| rno-miR-370-3p | up | ENSRNOT00000011670 | ENSRNOG00000008634 | Cc2d1b | coiled-coil and C2 domain containing 1B | |
| rno-miR-665 | up | ENSRNOT00000090760 | ENSRNOG00000058916 | Ccdc65 | coiled-coil domain containing 65 | |
| rno-miR-370-3p | up | ENSRNOT00000089179 | ENSRNOG00000013603 | Dffa | DNA fragmentation factor subunit alpha | |
| rno-miR-1193-3p | up | ENSRNOT00000057179 | ENSRNOG00000046275 | LOC100910833 | histone-lysine N-methyltransferase setd3-like | |
| rno-miR-212-5p | up | ENSRNOT00000092993 | ENSRNOG00000019638 | Lmna | lamin A/C | |
| rno-miR-127-5p | up | ENSRNOT00000067754 | ENSRNOG00000019627 | Mybpc2 | myosin binding protein C, fast-type | |
| rno-miR-370-3p | up | ENSRNOT00000026318 | ENSRNOG00000019388 | Egfl7 | EGF-like-domain, multiple 7 | |
| rno-miR-370-3p | up | ENSRNOT00000048370 | ENSRNOG00000050756 | Olr235 | olfactory receptor 235 | |
| rno-miR-370-3p | up | ENSRNOT00000051241 | ENSRNOG00000030863 | Olr239 | olfactory receptor 239 | |
| rno-miR-370-3p | up | ENSRNOT00000079923 | ENSRNOG00000006961 | Snrpb | small nuclear ribonucleoprotein polypeptides B and B1 | |
| rno-miR-665 | up | ENSRNOT00000087957 | ENSRNOG00000053260 | Lilrb3a | leukocyte immunoglobulin-like receptor, subfamily B (with TM and ITIM domains), member 3A | |
| rno-miR-212-5p | up | ENSRNOT00000029471 | ENSRNOG00000042059 | Rccd1 | RCC1 domain containing 1 | |
| rno-miR-370-3p | up | ENSRNOT00000009940 | ENSRNOG00000009284 | Foxa1 | forkhead box A1 | |
| rno-miR-370-3p | up | ENSRNOT00000077035 | ENSRNOG00000037100 | Heatr9 | HEAT repeat containing 9 | |
| rno-miR-485-5p | up | ENSRNOT00000072702 | ENSRNOG00000047958 | LOC108348032 | olfactory receptor 1 | |
| rno-miR-665 | up | ENSRNOT00000049985 | ENSRNOG00000001074 | Mphosph9 | M-phase phosphoprotein 9 | |
| rno-miR-665 | up | ENSRNOT00000031172 | ENSRNOG00000017673 | Brsk1 | BR serine/threonine kinase 1 | |
| rno-miR-370-3p | up | ENSRNOT00000037576 | ENSRNOG00000026036 | Pdyn | prodynorphin | |
| rno-miR-370-3p | up | ENSRNOT00000027760 | ENSRNOG00000020451 | Cd81 | Cd81 molecule | |
| rno-miR-214-3p | up | ENSRNOT00000000500 | ENSRNOG00000000437 | Agpat1 | 1-acylglycerol-3-phosphate O-acyltransferase 1 | |
| rno-miR-214-3p | up | ENSRNOT00000074974 | ENSRNOG00000050848 | Dtx1 | deltex E3 ubiquitin ligase 1 | |
| rno-miR-214-3p | up | ENSRNOT00000072450 | ENSRNOG00000047450 | Lmo3 | LIM domain only 3 | |
| rno-miR-665 | up | ENSRNOT00000010464 | ENSRNOG00000007671 | Smu1 | DNA replication regulator and spliceosomal factor | |
| rno-miR-214-3p | up | ENSRNOT00000091491 | ENSRNOG00000000437 | Agpat1 | 1-acylglycerol-3-phosphate O-acyltransferase 1 | |
| rno-miR-370-3p | up | ENSRNOT00000009606 | ENSRNOG00000007307 | Syde1 | synapse defective Rho GTPase homolog 1 | |
| rno-miR-370-3p | up | ENSRNOT00000014720 | ENSRNOG00000010834 | Mpped1 | metallophosphoesterase domain containing 1 | |
| rno-miR-214-3p | up | ENSRNOT00000089941 | ENSRNOG00000016253 | Slc6a18 | solute carrier family 6 member 18 | |
| rno-miR-665 | up | ENSRNOT00000078046 | ENSRNOG00000057092 | Slfn4 | schlafen 4 | |
| rno-miR-665 | up | ENSRNOT00000079325 | ENSRNOG00000057092 | Slfn4 | schlafen 4 | |
| rno-miR-370-3p | up | ENSRNOT00000092343 | ENSRNOG00000061989 | Nkrf | NFKB repressing factor | |
| rno-miR-127-3p | up | ENSRNOT00000028487 | ENSRNOG00000020970 | Rundc3a | RUN domain containing 3A | |
| rno-miR-370-3p | up | ENSRNOT00000082603 | ENSRNOG00000007400 | Srebf2 | sterol regulatory element binding transcription factor 2 | |
| rno-miR-665 | up | ENSRNOT00000081708 | ENSRNOG00000016468 | Tpmt | thiopurine S-methyltransferase | |
| rno-miR-370-3p | up | ENSRNOT00000001989 | ENSRNOG00000001457 | Rfc2 | replication factor C subunit 2 | |
| rno-miR-370-3p | up | ENSRNOT00000076398 | ENSRNOG00000007596 | Rffl | ring finger and FYVE-like domain containing E3 ubiquitin protein ligase | |
| rno-miR-212-5p | up | ENSRNOT00000001696 | ENSRNOG00000001258 | Snx8 | sorting nexin 8 | |
| rno-miR-665 | up | ENSRNOT00000007485 | ENSRNOG00000005670 | Art4 | ADP-ribosyltransferase 4 | |
| rno-miR-127-5p | up | ENSRNOT00000021946 | ENSRNOG00000016539 | Rab24 | RAB24, member RAS oncogene family | |
| rno-miR-370-3p | up | ENSRNOT00000015546 | ENSRNOG00000010846 | Ccdc22 | coiled-coil domain containing 22 | |
| rno-miR-370-3p | up | ENSRNOT00000017172 | ENSRNOG00000012877 | Rgs9bp | regulator of G protein signaling 9 binding protein | |
| rno-miR-370-3p | up | ENSRNOT00000091711 | ENSRNOG00000059408 | Prr12 | proline rich 12 | |
| rno-miR-370-3p | up | ENSRNOT00000017369 | ENSRNOG00000012619 | Epor | erythropoietin receptor | |
| rno-miR-140-3p | up | ENSRNOT00000031074 | ENSRNOG00000021776 | Cenpc | centromere protein C | |
| rno-miR-370-3p | up | ENSRNOT00000085704 | ENSRNOG00000060266 | Exoc2 | exocyst complex component 2 | |
| rno-miR-370-3p | up | ENSRNOT00000092638 | ENSRNOG00000045721 | Exoc2 | exocyst complex component 2 | |
| rno-miR-665 | up | ENSRNOT00000022549 | ENSRNOG00000016750 | Cyp26a1 | cytochrome P450, family 26, subfamily a, polypeptide 1 | |
| rno-miR-665 | up | ENSRNOT00000049038 | ENSRNOG00000033338 | Gimap6 | GTPase, IMAP family member 6 | |
| rno-miR-140-3p | up | ENSRNOT00000045521 | ENSRNOG00000002145 | Tmprss7 | transmembrane protease, serine 7 | |
| rno-miR-337-5p | up | ENSRNOT00000009008 | ENSRNOG00000006813 | Sumf1 | sulfatase modifying factor 1 | |
| rno-miR-370-3p | up | ENSRNOT00000002394 | ENSRNOG00000001750 | Chrd | chordin | |
| rno-miR-665 | up | ENSRNOT00000022748 | ENSRNOG00000016888 | Pask | PAS domain containing serine/threonine kinase | |
| rno-miR-370-3p | up | ENSRNOT00000005930 | ENSRNOG00000004494 | Lta4h | leukotriene A4 hydrolase | |
| rno-miR-541-3p | up | ENSRNOT00000080220 | ENSRNOG00000039260 | RGD1563060 | similar to AVLV472 | |
| rno-miR-665 | up | ENSRNOT00000021743 | ENSRNOG00000016224 | Tldc1 | TBC/LysM-associated domain containing 1 | |
| rno-miR-665 | up | ENSRNOT00000020780 | ENSRNOG00000015465 | Cep72 | centrosomal protein 72 | |
| rno-miR-665 | up | ENSRNOT00000027539 | ENSRNOG00000020317 | RGD1307752 | similar to RIKEN cDNA 1110008F13 | |
| rno-miR-370-3p | up | ENSRNOT00000045223 | ENSRNOG00000001082 | Abcb9 | ATP binding cassette subfamily B member 9 | |
| rno-miR-370-3p | up | ENSRNOT00000006780 | ENSRNOG00000004996 | Zcrb1 | zinc finger CCHC-type and RNA binding motif containing 1 | |
| rno-miR-214-3p | up | ENSRNOT00000036314 | ENSRNOG00000023342 | Prr27 | proline rich 27 | |
| rno-miR-493-3p | up | ENSRNOT00000027454 | ENSRNOG00000020245 | Syt8 | synaptotagmin 8 | |
| rno-miR-300-3p | up | ENSRNOT00000085094 | ENSRNOG00000011977 | Sema5a | semaphorin 5A | |
| rno-miR-127-5p | up | ENSRNOT00000045996 | ENSRNOG00000031263 | Haao | 3-hydroxyanthranilate 3,4-dioxygenase | |
| rno-miR-370-3p | up | ENSRNOT00000010895 | ENSRNOG00000007519 | Tmem43 | transmembrane protein 43 | |
| rno-miR-214-3p | up | ENSRNOT00000009946 | ENSRNOG00000007427 | Entpd6 | ectonucleoside triphosphate diphosphohydrolase 6 | |
| rno-novel-58-mature | up | ENSRNOT00000083402 | ENSRNOG00000010474 | Hmces | 5-hydroxymethylcytosine (hmC) binding, ES cell-specific | |
| rno-miR-665 | up | ENSRNOT00000000102 | ENSRNOG00000000091 | Smad9 | SMAD family member 9 | |
| rno-miR-127-3p | up | ENSRNOT00000028861 | ENSRNOG00000021247 | Spef1 | sperm flagellar 1 | |
| rno-miR-370-3p | up | ENSRNOT00000020130 | ENSRNOG00000014845 | Strc | stereocilin | |
| rno-miR-370-3p | up | ENSRNOT00000037064 | ENSRNOG00000026848 | Tex36 | testis expressed 36 | |
| rno-miR-370-3p | up | ENSRNOT00000086703 | ENSRNOG00000026848 | Tex36 | testis expressed 36 | |
| rno-miR-127-3p | up | ENSRNOT00000004722 | ENSRNOG00000003424 | Elac2 | elaC ribonuclease Z 2 | |
| rno-miR-370-3p | up | ENSRNOT00000071020 | ENSRNOG00000045568 | Rbm14 | RNA binding motif protein 14 | |
| rno-miR-370-3p | up | ENSRNOT00000072373 | ENSRNOG00000045568 | Rbm14 | RNA binding motif protein 14 | |
| rno-miR-665 | up | ENSRNOT00000030706 | ENSRNOG00000022697 | Clec14a | C-type lectin domain family 14, member A | |
| rno-miR-433-3p | up | ENSRNOT00000033427 | ENSRNOG00000008552 | P2rx3 | purinergic receptor P2X 3 | |
| rno-miR-127-5p | up | ENSRNOT00000075978 | ENSRNOG00000025791 | Dync1li2 | dynein, cytoplasmic 1 light intermediate chain 2 | |
| rno-miR-673-3p | up | ENSRNOT00000067277 | ENSRNOG00000008105 | Dmrta2 | DMRT-like family A2 | |
| rno-miR-370-3p | up | ENSRNOT00000016401 | ENSRNOG00000039284 | Haus4 | HAUS augmin-like complex, subunit 4 | |
| rno-miR-665 | up | ENSRNOT00000004385 | ENSRNOG00000003253 | Qdpr | quinoid dihydropteridine reductase | |
| rno-miR-370-3p | up | ENSRNOT00000064250 | ENSRNOG00000017116 | Zfp532 | zinc finger protein 532 | |
| rno-miR-127-3p | up | ENSRNOT00000079621 | ENSRNOG00000021170 | Plekho1 | pleckstrin homology domain containing O1 | |
| rno-miR-370-3p | up | ENSRNOT00000023455 | ENSRNOG00000016991 | Arl2bp | ADP-ribosylation factor like GTPase 2 binding protein | |
| rno-miR-673-3p | up | ENSRNOT00000017903 | ENSRNOG00000013320 | RGD1561916 | similar to testes development-related NYD-SP22 isoform 1 | |
| rno-miR-370-3p | up | ENSRNOT00000009914 | ENSRNOG00000007025 | Evc2 | EvC ciliary complex subunit 2 | |
| rno-miR-300-3p | up | ENSRNOT00000016506 | ENSRNOG00000011977 | Sema5a | semaphorin 5A | |
| rno-miR-665 | up | ENSRNOT00000018780 | ENSRNOG00000014046 | Sertm1 | serine-rich and transmembrane domain containing 1 | |
| rno-miR-370-3p | up | ENSRNOT00000054920 | ENSRNOG00000036664 | Narf | nuclear prelamin A recognition factor | |
| rno-miR-212-5p | up | ENSRNOT00000023592 | ENSRNOG00000017557 | LOC100362216 | hypothetical protein LOC100362216 ] | |
| rno-miR-370-3p | up | ENSRNOT00000005445 | ENSRNOG00000036888 | LOC691189 | hypothetical protein LOC691189 | |
| rno-miR-214-3p | up | ENSRNOT00000011490 | ENSRNOG00000008274 | Xpc | XPC complex subunit, DNA damage recognition and repair factor | |
| rno-miR-370-3p | up | ENSRNOT00000023068 | ENSRNOG00000016957 | Igfbp2 | insulin-like growth factor binding protein Symbol;Acc:2873] | |
| rno-miR-214-3p | up | ENSRNOT00000027353 | ENSRNOG00000020140 | Pigq | phosphatidylinositol glycan anchor biosynthesis, class Q | |
| rno-miR-433-3p | up | ENSRNOT00000088815 | ENSRNOG00000008552 | P2rx3 | purinergic receptor P2X 3 | |
| rno-miR-370-3p | up | ENSRNOT00000038376 | ENSRNOG00000021780 | Rad51d | RAD51 paralog D | |
| rno-miR-370-3p | up | ENSRNOT00000004999 | ENSRNOG00000003328 | Cdip1 | cell death-inducing p53 target 1 | |
| rno-miR-370-3p | up | ENSRNOT00000077526 | ENSRNOG00000056038 | Ehbp1l1 | EH domain binding protein 1-like 1 | |
| rno-miR-127-5p | up | ENSRNOT00000063831 | ENSRNOG00000009596 | LOC103689945 | zinc finger SWIM domain-containing protein 8-like | |
| rno-miR-370-3p | up | ENSRNOT00000076158 | ENSRNOG00000028856 | Pknox2 | PBX/knotted 1 homeobox 2 | |
| rno-miR-341 | up | ENSRNOT00000083472 | ENSRNOG00000006789 | Ddit3 | DNA-damage inducible transcript 3 | |
| rno-miR-673-3p | up | ENSRNOT00000085001 | ENSRNOG00000053093 | Vom1r99 | vomeronasal 1 receptor 99 | |
| rno-miR-370-3p | up | ENSRNOT00000072190 | ENSRNOG00000046202 | Metrnl | meteorin-like, glial cell differentiation regulator | |
| rno-miR-212-5p | up | ENSRNOT00000093036 | ENSRNOG00000019638 | Lmna | lamin A/C | |
| rno-miR-370-3p | up | ENSRNOT00000013720 | ENSRNOG00000010238 | Chmp4c | charged multivesicular body protein 4C | |
| rno-miR-31a-5p | up | ENSRNOT00000004888 | ENSRNOG00000003596 | Itgb1bp2 | integrin subunit beta 1 binding protein 2 | |
| rno-miR-665 | up | ENSRNOT00000059530 | ENSRNOG00000001422 | Col26a1 | collagen type XXVI alpha 1 chain | |
| rno-miR-370-3p | up | ENSRNOT00000043036 | ENSRNOG00000032417 | Gabrp | gamma-aminobutyric acid type A receptor pi subunit | |
| rno-miR-214-3p | up | ENSRNOT00000084543 | ENSRNOG00000009052 | Igf2bp3 | insulin-like growth factor 2 mRNA binding protein 3 | |
| rno-miR-665 | up | ENSRNOT00000022085 | ENSRNOG00000016468 | Tpmt | thiopurine S-methyltransferase | |
| rno-miR-665 | up | ENSRNOT00000009341 | ENSRNOG00000007126 | Gpr19 | G protein-coupled receptor 19 | |
| rno-miR-370-3p | up | ENSRNOT00000080288 | ENSRNOG00000053239 | Clmp | CXADR-like membrane protein | |
| rno-miR-127-3p | up | ENSRNOT00000028747 | ENSRNOG00000021170 | Plekho1 | pleckstrin homology domain containing O1 | |
| rno-miR-370-3p | up | ENSRNOT00000008104 | ENSRNOG00000006049 | Rfx1 | regulatory factor X1 | |
| rno-miR-370-3p | up | ENSRNOT00000056041 | ENSRNOG00000007400 | Srebf2 | sterol regulatory element binding transcription factor 2 | |
| rno-miR-370-3p | up | ENSRNOT00000078741 | ENSRNOG00000010735 | Nfyc | nuclear transcription factor Y subunit gamma | |
| rno-miR-214-3p | up | ENSRNOT00000005074 | ENSRNOG00000003807 | Wnt9b | wingless-type MMTV integration site family, member 9B | |
| rno-miR-370-3p | up | ENSRNOT00000037973 | ENSRNOG00000026572 | Zfp473 | zinc finger protein 473 | |
| rno-miR-370-3p | up | ENSRNOT00000031492 | ENSRNOG00000026821 | Arhgap42 | Rho GTPase activating protein 42 | |
| rno-miR-214-3p | up | ENSRNOT00000022346 | ENSRNOG00000016253 | Slc6a18 | solute carrier family 6 member 18 | |
| rno-miR-214-3p | up | ENSRNOT00000015327 | ENSRNOG00000011441 | Tpd52 | tumor protein D52 | |
| rno-miR-665 | up | ENSRNOT00000079544 | ENSRNOG00000058416 | LOC103691556 | mothers against decapentaplegic homolog 9 | |
| rno-miR-370-3p | up | ENSRNOT00000064571 | ENSRNOG00000025071 | Qrich1 | glutamine-rich 1 | |
| rno-miR-665 | up | ENSRNOT00000093472 | ENSRNOG00000061146 | Gar1 | GAR1 ribonucleoprotein | |
| rno-miR-370-3p | up | ENSRNOT00000080455 | ENSRNOG00000056038 | Ehbp1l1 | EH domain binding protein 1-like 1 | |
| rno-miR-665 | up | ENSRNOT00000068033 | ENSRNOG00000046955 | Cbx6 | chromobox 6 | |
| rno-miR-140-3p | up | ENSRNOT00000041085 | ENSRNOG00000031966 | Olr1501 | olfactory receptor 1501 | |
| rno-miR-140-3p | up | ENSRNOT00000010473 | ENSRNOG00000007965 | Olr420 | olfactory receptor 420 | |
| rno-miR-214-3p | up | ENSRNOT00000087180 | ENSRNOG00000060853 | Iah1 | isoamyl acetate-hydrolyzing esterase 1 homolog | |
| rno-miR-370-3p | up | ENSRNOT00000028553 | ENSRNOG00000021026 | Zfp687 | zinc finger protein 687 | |
| rno-miR-370-3p | up | ENSRNOT00000068452 | ENSRNOG00000043077 | Zfpm1 | zinc finger protein, multitype 1 | |
| rno-miR-370-3p | up | ENSRNOT00000035881 | ENSRNOG00000023643 | Mmp17 | matrix metallopeptidase 17 | |
| rno-miR-370-3p | up | ENSRNOT00000086656 | ENSRNOG00000023480 | Nabp2 | nucleic acid binding protein 2 | |
| rno-miR-370-3p | up | ENSRNOT00000040060 | ENSRNOG00000033235 | Relb | RELB proto-oncogene, NF-kB subunit | |
| rno-miR-665 | up | ENSRNOT00000001810 | ENSRNOG00000039234 | Zscan21 | zinc finger and SCAN domain containing 21 | |
| rno-miR-127-3p | up | ENSRNOT00000056200 | ENSRNOG00000037206 | Ccdc77 | coiled-coil domain containing 77 | |
| rno-miR-665 | up | ENSRNOT00000078074 | ENSRNOG00000061146 | Gar1 | GAR1 ribonucleoprotein | |
| rno-miR-370-3p | up | ENSRNOT00000057802 | ENSRNOG00000033235 | Relb | RELB proto-oncogene, NF-kB subunit | |
| rno-miR-485-5p | up | ENSRNOT00000014147 | ENSRNOG00000010392 | Nrg1 | neuregulin 1 | |
| rno-miR-485-5p | up | ENSRNOT00000014268 | ENSRNOG00000010392 | Nrg1 | neuregulin 1 | |
| rno-miR-485-5p | up | ENSRNOT00000058727 | ENSRNOG00000010392 | Nrg1 | neuregulin 1 | |
| rno-miR-370-3p | up | ENSRNOT00000027301 | ENSRNOG00000020150 | Il18bp | interleukin 18 binding protein | |
| rno-miR-370-3p | up | ENSRNOT00000005674 | ENSRNOG00000004294 | Ascl1 | achaete-scute family bHLH transcription factor 1 | |
| rno-miR-370-3p | up | ENSRNOT00000010510 | ENSRNOG00000007989 | Chst1 | carbohydrate sulfotransferase 1 | |
| rno-miR-665 | up | ENSRNOT00000047291 | ENSRNOG00000016069 | Cd3e | CD3e molecule | |
| rno-miR-370-3p | up | ENSRNOT00000064178 | ENSRNOG00000047206 | LOC100911727 | DNA ligase 1-like | |
| rno-miR-665 | up | ENSRNOT00000035805 | ENSRNOG00000024809 | Ntmt1 | N-terminal Xaa-Pro-Lys N-methyltransferase 1 | |
| rno-miR-127-3p | up | ENSRNOT00000010790 | ENSRNOG00000007931 | Gucy2d | guanylate cyclase 2D, retinal | |
| rno-miR-370-3p | up | ENSRNOT00000075189 | ENSRNOG00000050032 | Srrm5 | serine/arginine repetitive matrix 5 | |
| rno-miR-370-3p | up | ENSRNOT00000026921 | ENSRNOG00000019798 | Stub1 | STIP1 homology and U-box containing protein 1 | |
| rno-miR-370-3p | up | ENSRNOT00000059530 | ENSRNOG00000001422 | Col26a1 | collagen type XXVI alpha 1 chain | |
| rno-miR-370-3p | up | ENSRNOT00000076598 | ENSRNOG00000007596 | Rffl | ring finger and FYVE-like domain containing E3 ubiquitin protein ligase | |
| rno-miR-665 | up | ENSRNOT00000077823 | ENSRNOG00000032929 | Incenp | inner centromere protein | |
| rno-miR-212-5p | up | ENSRNOT00000068233 | ENSRNOG00000004760 | Lars2 | leucyl-tRNA synthetase 2 | |
| rno-miR-136-3p | up | ENSRNOT00000089339 | ENSRNOG00000016550 | Dclk2 | doublecortin-like kinase 2 | |
| rno-miR-370-3p | up | ENSRNOT00000030885 | ENSRNOG00000027901 | Mrm1 | mitochondrial rRNA methyltransferase 1 | |
| rno-miR-214-3p | up | ENSRNOT00000025382 | ENSRNOG00000018745 | Sgsm3 | small G protein signaling modulator 3 | |
| rno-miR-665 | up | ENSRNOT00000083935 | ENSRNOG00000036833 | Zfp385a | zinc finger protein 385A | |
| rno-miR-370-3p | up | ENSRNOT00000031472 | ENSRNOG00000028356 | LOC103694872 | coiled-coil-helix-coiled-coil-helix domain-containing protein 10, mitochondrial | |
| rno-miR-212-5p | up | ENSRNOT00000009562 | ENSRNOG00000007266 | RGD1311251 | similar to RIKEN cDNA 4930550C14 | |
| rno-miR-665 | up | ENSRNOT00000088357 | ENSRNOG00000013383 | Tmub1 | transmembrane and ubiquitin-like domain containing 1 | |
| rno-miR-665 | up | ENSRNOT00000046807 | ENSRNOG00000019477 | Zmynd15 | zinc finger, MYND-type containing 15 | |
| rno-miR-370-3p | up | ENSRNOT00000021357 | ENSRNOG00000015674 | Acap1 | ArfGAP with coiled-coil, ankyrin repeat and PH domains 1 | |
| rno-miR-665 | up | ENSRNOT00000002838 | ENSRNOG00000002072 | Eva1c | eva-1 homolog C | |
| rno-miR-214-3p | up | ENSRNOT00000039115 | ENSRNOG00000021573 | Dpy19l3 | dpy-19-like 3 (C. elegans) | |
| rno-miR-370-3p | up | ENSRNOT00000001733 | ENSRNOG00000001288 | Gpr146 | G protein-coupled receptor 146 | |
| rno-miR-214-3p | up | ENSRNOT00000013991 | ENSRNOG00000010392 | Nrg1 | neuregulin 1 | |
| rno-miR-214-3p | up | ENSRNOT00000081522 | ENSRNOG00000010392 | Nrg1 | neuregulin 1 | |
| rno-miR-214-3p | up | ENSRNOT00000082355 | ENSRNOG00000010392 | Nrg1 | neuregulin 1 | |
| rno-miR-370-3p | up | ENSRNOT00000071119 | ENSRNOG00000050035 | Sall4 | spalt-like transcription factor 4 | |
| rno-miR-1193-3p | up | ENSRNOT00000009120 | ENSRNOG00000006587 | Setd3 | SET domain containing 3 | |
| rno-miR-214-3p | up | ENSRNOT00000078561 | ENSRNOG00000003807 | Wnt9b | wingless-type MMTV integration site family, member 9B | |
| rno-miR-6331 | up | ENSRNOT00000001479 | ENSRNOG00000001117 | Fbxl18 | F-box and leucine-rich repeat protein 18 | |
| rno-miR-370-3p | up | ENSRNOT00000022046 | ENSRNOG00000016368 | Ppp1r14c | protein phosphatase 1, regulatory (inhibitor) subunit 14c | |
| rno-miR-127-5p | up | ENSRNOT00000010059 | ENSRNOG00000007655 | Zfp174 | zinc finger protein 174 | |
| rno-miR-341 | up | ENSRNOT00000022984 | ENSRNOG00000017021 | Galnt18 | polypeptide N-acetylgalactosaminyltransferase 18 | |
| rno-miR-370-3p | up | ENSRNOT00000072001 | ENSRNOG00000047052 | Clpp | caseinolytic mitochondrial matrix peptidase proteolytic subunit | |
| rno-miR-214-3p | up | ENSRNOT00000026988 | ENSRNOG00000019907 | Nfkbie | NFKB inhibitor epsilon | |
| rno-miR-214-3p | up | ENSRNOT00000049729 | ENSRNOG00000032374 | Paqr9 | progestin and adipoQ receptor family member 9 | |
| rno-miR-370-3p | up | ENSRNOT00000000308 | ENSRNOG00000000283 | Dgcr14 | DiGeorge syndrome critical region gene 14 | |
| rno-miR-370-3p | up | ENSRNOT00000034563 | ENSRNOG00000021763 | Ffar4 | free fatty acid receptor 4 | |
| rno-miR-370-3p | up | ENSRNOT00000028861 | ENSRNOG00000021247 | Spef1 | sperm flagellar 1 | |
| rno-miR-673-3p | up | ENSRNOT00000028812 | ENSRNOG00000021217 | Itga10 | integrin subunit alpha 10 | |
| rno-miR-370-3p | up | ENSRNOT00000004843 | ENSRNOG00000042691 | Armc7 | armadillo repeat containing 7 | |
| rno-miR-665 | up | ENSRNOT00000006786 | ENSRNOG00000005008 | Angpt4 | angiopoietin 4 | |
| rno-miR-541-5p | up | ENSRNOT00000028117 | ENSRNOG00000020706 | Kcnn3 | potassium calcium-activated channel subfamily N member 3 | |
| rno-miR-673-3p | up | ENSRNOT00000001322 | ENSRNOG00000000994 | Stxbp2 | syntaxin binding protein 2 | |
| rno-miR-212-5p | up | ENSRNOT00000027445 | ENSRNOG00000020246 | Myl9 | myosin light chain 9 | |
| rno-miR-370-3p | up | ENSRNOT00000022020 | ENSRNOG00000016177 | Scara3 | scavenger receptor class A, member 3 | |
| rno-miR-665 | up | ENSRNOT00000086244 | ENSRNOG00000001074 | Mphosph9 | M-phase phosphoprotein 9 | |
| rno-miR-370-3p | up | ENSRNOT00000000525 | ENSRNOG00000032708 | RT1-Bb | RT1 class II, locus Bb | |
| rno-miR-214-3p | up | ENSRNOT00000033749 | ENSRNOG00000015262 | Slc34a1 | solute carrier family 34 member 1 | |
| rno-miR-127-5p | up | ENSRNOT00000059673 | ENSRNOG00000010107 | Palld | palladin, cytoskeletal associated protein | |
| rno-miR-370-3p | up | ENSRNOT00000068135 | ENSRNOG00000007492 | Rpn2 | ribophorin II | |
| rno-miR-140-3p | up | ENSRNOT00000083667 | ENSRNOG00000057256 | Slc19a3 | solute carrier family 19 member 3 | |
| rno-miR-665 | up | ENSRNOT00000084921 | ENSRNOG00000058755 | Tgm7l1 | transglutaminase 7-like 1 | |
| rno-miR-370-3p | up | ENSRNOT00000016163 | ENSRNOG00000011876 | Fxr2 | FMR1 autosomal homolog 2 | |
| rno-miR-665 | up | ENSRNOT00000065983 | ENSRNOG00000016249 | Cep85 | centrosomal protein 85 | |
| rno-miR-370-3p | up | ENSRNOT00000015181 | ENSRNOG00000011151 | Tenm4 | teneurin transmembrane protein 4 | |
| rno-miR-665 | up | ENSRNOT00000086554 | ENSRNOG00000036833 | Zfp385a | zinc finger protein 385A | |
| rno-miR-127-3p | up | ENSRNOT00000034266 | ENSRNOG00000016825 | Cd3eap | CD3e molecule associated protein | |
| rno-miR-370-3p | up | ENSRNOT00000056651 | ENSRNOG00000010735 | Nfyc | nuclear transcription factor Y subunit gamma | |
| rno-miR-434-5p | up | ENSRNOT00000072399 | ENSRNOG00000048751 | Rtl1 | retrotransposon-like 1 | |
| rno-miR-370-3p | up | ENSRNOT00000014249 | ENSRNOG00000010378 | Slc4a5 | solute carrier family 4 member 5 | |
| rno-miR-665 | up | ENSRNOT00000074122 | ENSRNOG00000048676 | Pip5kl1 | phosphatidylinositol-4-phosphate 5-kinase-like 1 | |
| rno-miR-665 | up | ENSRNOT00000091190 | ENSRNOG00000016069 | Cd3e | CD3e molecule | |
| rno-miR-370-3p | up | ENSRNOT00000010986 | ENSRNOG00000008174 | Appl2 | adaptor protein, phosphotyrosine interacting with PH domain and leucine zipper 2 | |
| rno-miR-485-5p | up | ENSRNOT00000020728 | ENSRNOG00000015075 | Stc1 | stanniocalcin 1 | |
| rno-miR-665 | up | ENSRNOT00000075773 | ENSRNOG00000049303 | Marco | macrophage receptor with collagenous structure | |
| rno-miR-127-5p | up | ENSRNOT00000067916 | ENSRNOG00000018762 | Rsph3 | radial spoke 3 homolog | |
| rno-miR-665 | up | ENSRNOT00000071971 | ENSRNOG00000050348 | LOC684270 | similar to isochorismatase domain containing 2 | |
| rno-miR-370-3p | up | ENSRNOT00000007889 | ENSRNOG00000005984 | Etv6 | ets variant 6 | |
| rno-miR-1193-3p | up | ENSRNOT00000014284 | ENSRNOG00000010763 | Fam181b | family with sequence similarity 181, member B | |
| rno-miR-665 | up | ENSRNOT00000021534 | ENSRNOG00000015906 | Tgif1 | TGFB-induced factor homeobox 1 | |
| rno-miR-370-3p | up | ENSRNOT00000011632 | ENSRNOG00000008800 | Olr1621 | olfactory receptor 1621 | |
| rno-miR-665 | up | ENSRNOT00000074336 | ENSRNOG00000049959 | Igsf21 | immunoglobin superfamily, member 21 | |
| rno-miR-341 | up | ENSRNOT00000037185 | ENSRNOG00000014847 | Rassf10 | Ras association domain family member 10 | |
| rno-miR-541-5p | up | ENSRNOT00000083439 | ENSRNOG00000055055 | Olr1486 | olfactory receptor 1486 | |
| rno-miR-140-3p | up | ENSRNOT00000034672 | ENSRNOG00000024824 | Col22a1 | collagen type XXII alpha 1 chain | |
| rno-miR-665 | up | ENSRNOT00000064700 | ENSRNOG00000009592 | Cyb5r3 | cytochrome b5 reductase 3 | |
| rno-miR-665 | up | ENSRNOT00000093665 | ENSRNOG00000003792 | Med14 | mediator complex subunit 14 | |
| rno-novel-58-mature | up | ENSRNOT00000037564 | ENSRNOG00000028100 | Pex11g | peroxisomal biogenesis factor 11 gamma | |
| rno-novel-58-mature | up | ENSRNOT00000024581 | ENSRNOG00000018229 | Slc45a1 | solute carrier family 45, member 1 | |
| rno-miR-140-3p | up | ENSRNOT00000021926 | ENSRNOG00000016308 | Il10ra | interleukin 10 receptor subunit alpha | |
| rno-miR-370-3p | up | ENSRNOT00000049864 | ENSRNOG00000032522 | RGD1560784 | similar to RIKEN cDNA B630019K06 | |
| rno-miR-214-3p | up | ENSRNOT00000067539 | ENSRNOG00000020563 | Cma1 | chymase 1 | |
| rno-miR-370-3p | up | ENSRNOT00000004885 | ENSRNOG00000003645 | LOC103689931 | heterogeneous nuclear ribonucleoprotein A/B | |
| rno-miR-665 | up | ENSRNOT00000072511 | ENSRNOG00000049426 | Mmab | methylmalonic aciduria (cobalamin deficiency) cblB type | |
| rno-miR-370-3p | up | ENSRNOT00000082403 | ENSRNOG00000017116 | Zfp532 | zinc finger protein 532 | |
| rno-miR-673-3p | up | ENSRNOT00000025977 | ENSRNOG00000019216 | Il12rb1 | interleukin 12 receptor subunit beta 1 | |
| rno-miR-370-3p | up | ENSRNOT00000091894 | ENSRNOG00000028856 | Pknox2 | PBX/knotted 1 homeobox 2 | |
| rno-miR-665 | up | ENSRNOT00000087434 | ENSRNOG00000027869 | Sox5 | SRY box 5 | |
| rno-miR-127-5p | up | ENSRNOT00000089107 | ENSRNOG00000017220 | Tcirg1 | T-cell immune regulator 1, ATPase H+ transporting V0 subunit A3 | |
| rno-miR-665 | up | ENSRNOT00000054949 | ENSRNOG00000036682 | Pycr1 | pyrroline-5-carboxylate reductase 1 | |
| rno-miR-212-5p | up | ENSRNOT00000028263 | ENSRNOG00000020832 | C2cd4d | C2 calcium-dependent domain containing 4D | |
| rno-miR-127-3p | up | ENSRNOT00000091221 | ENSRNOG00000011875 | Slc2a2 | solute carrier family 2 member 2 | |
| rno-miR-665 | up | ENSRNOT00000061992 | ENSRNOG00000013383 | Tmub1 | transmembrane and ubiquitin-like domain containing 1 | |
| rno-miR-370-3p | up | ENSRNOT00000082290 | ENSRNOG00000010967 | Cdc37l1 | cell division cycle 37-like 1 | |
| rno-miR-127-3p | up | ENSRNOT00000068386 | ENSRNOG00000042137 | Tmem173 | transmembrane protein 173 | |
| rno-miR-665 | up | ENSRNOT00000012365 | ENSRNOG00000009316 | Bmp10 | bone morphogenetic protein 10 | |
| rno-miR-370-3p | up | ENSRNOT00000014191 | ENSRNOG00000023828 | LOC680875 | similar to dystonin isoform 1 | |
| rno-miR-127-5p | up | ENSRNOT00000023425 | ENSRNOG00000017220 | Tcirg1 | T-cell immune regulator 1, ATPase H+ transporting V0 subunit A3 | |
| rno-novel-58-mature | up | ENSRNOT00000026862 | ENSRNOG00000019812 | Nr1h2 | nuclear receptor subfamily 1, group H, member 2 | |
| rno-miR-665 | up | ENSRNOT00000030885 | ENSRNOG00000027901 | Mrm1 | mitochondrial rRNA methyltransferase 1 | |
| rno-miR-370-3p | up | ENSRNOT00000082402 | ENSRNOG00000008754 | Flvcr2 | feline leukemia virus subgroup C cellular receptor family, member 2 | |
| rno-miR-370-3p | up | ENSRNOT00000089972 | ENSRNOG00000008754 | Flvcr2 | feline leukemia virus subgroup C cellular receptor family, member 2 | |
| rno-miR-370-3p | up | ENSRNOT00000050227 | ENSRNOG00000019270 | P2ry6 | pyrimidinergic receptor P2Y6 | |
| rno-miR-212-5p | up | ENSRNOT00000040851 | ENSRNOG00000031061 | LOC306079 | similar to RIKEN cDNA 3100001N19 | |
| rno-miR-370-3p | up | ENSRNOT00000086264 | ENSRNOG00000017558 | Tubb2a | tubulin, beta 2A class IIa | |
| rno-miR-127-5p | up | ENSRNOT00000037257 | ENSRNOG00000025498 | Crb2 | crumbs 2, cell polarity complex component | |
| rno-miR-370-3p | up | ENSRNOT00000001368 | ENSRNOG00000001035 | Gtf2h3 | general transcription factor IIH subunit 3 | |
| rno-miR-665 | up | ENSRNOT00000078063 | ENSRNOG00000016249 | Cep85 | centrosomal protein 85 | |
| rno-miR-370-3p | up | ENSRNOT00000033863 | ENSRNOG00000024693 | Zfp831 | zinc finger protein 831 | |
| rno-miR-341 | up | ENSRNOT00000026063 | ENSRNOG00000019193 | Stx1b | syntaxin 1B | |
| rno-miR-370-3p | up | ENSRNOT00000052347 | ENSRNOG00000034230 | Fcrl1 | Fc receptor-like 1 | |
| rno-miR-212-5p | up | ENSRNOT00000087488 | ENSRNOG00000004760 | Lars2 | leucyl-tRNA synthetase 2 | |
| rno-miR-370-3p | up | ENSRNOT00000009648 | ENSRNOG00000007292 | Spdl1 | spindle apparatus coiled-coil protein 1 | |
| rno-miR-214-3p | up | ENSRNOT00000008327 | ENSRNOG00000006142 | Tmem8c | transmembrane protein 8C | |
| rno-miR-370-3p | up | ENSRNOT00000020864 | ENSRNOG00000015152 | Pkp3 | plakophilin 3 | |
| rno-miR-370-3p | up | ENSRNOT00000005426 | ENSRNOG00000004091 | Cwc25 | CWC25 spliceosome-associated protein homolog | |
| rno-miR-665 | up | ENSRNOT00000056030 | ENSRNOG00000015675 | Nell1 | neural EGFL like 1 | |
| rno-miR-370-3p | up | ENSRNOT00000039480 | ENSRNOG00000023467 | Fam168b | family with sequence similarity 168, member B | |
| rno-miR-665 | up | ENSRNOT00000077744 | ENSRNOG00000058820 | Olr1768 | olfactory receptor 1768 | |
| rno-miR-370-3p | up | ENSRNOT00000039709 | ENSRNOG00000026474 | Cad | carbamoyl-phosphate synthetase 2, aspartate transcarbamylase, and dihydroorotase | |
| rno-miR-665 | up | ENSRNOT00000043387 | ENSRNOG00000032929 | Incenp | inner centromere protein | |
| rno-miR-665 | up | ENSRNOT00000003458 | ENSRNOG00000002568 | Socs1 | suppressor of cytokine signaling 1 | |
| rno-miR-370-3p | up | ENSRNOT00000023582 | ENSRNOG00000017445 | Tubb2b | tubulin, beta 2B class IIb | |
| rno-miR-370-3p | up | ENSRNOT00000088048 | ENSRNOG00000014963 | Adgrg1 | adhesion G protein-coupled receptor G1 | |
| rno-miR-665 | up | ENSRNOT00000033900 | ENSRNOG00000024082 | Gldn | gliomedin | |
| rno-miR-370-3p | up | ENSRNOT00000072796 | ENSRNOG00000046515 | St6gal2 | ST6 beta-galactoside alpha-2,6-sialyltransferase 2 | |
| rno-miR-370-3p | up | ENSRNOT00000044567 | ENSRNOG00000032293 | Polg | DNA polymerase gamma, catalytic subunit | |
| rno-miR-370-3p | up | ENSRNOT00000030978 | ENSRNOG00000022637 | AABR07052585.1 |  | |
| rno-miR-665 | up | ENSRNOT00000007773 | ENSRNOG00000005929 | Them6 | thioesterase superfamily member 6 | |
| rno-miR-127-5p | up | ENSRNOT00000005908 | ENSRNOG00000004482 | Ccdc88c | coiled-coil domain containing 88C | |
| rno-miR-370-3p | up | ENSRNOT00000083705 | ENSRNOG00000017445 | Tubb2b | tubulin, beta 2B class IIb | |
| rno-miR-127-5p | up | ENSRNOT00000073220 | ENSRNOG00000045795 | Pddc1 | Parkinson disease 7 domain containing 1 | |
| rno-miR-370-3p | up | ENSRNOT00000075767 | ENSRNOG00000047505 | Tubb4a | tubulin, beta 4A class IVa | |
| rno-miR-665 | up | ENSRNOT00000065292 | ENSRNOG00000028812 | Trim80 | tripartite motif protein 80 | |
| rno-miR-370-3p | up | ENSRNOT00000023611 | ENSRNOG00000017558 | Tubb2a | tubulin, beta 2A class IIa | |
| rno-miR-665 | up | ENSRNOT00000064261 | ENSRNOG00000036833 | Zfp385a | zinc finger protein 385A | |
| rno-miR-212-5p | up | ENSRNOT00000071893 | ENSRNOG00000047531 | Ccdc57 | coiled-coil domain containing 57 | |
| rno-miR-370-3p | up | ENSRNOT00000085031 | ENSRNOG00000008754 | Flvcr2 | feline leukemia virus subgroup C cellular receptor family, member 2 | |
| rno-miR-370-3p | up | ENSRNOT00000048922 | ENSRNOG00000007371 | Rbmx2 | RNA binding motif protein, X-linked 2 | |
| rno-miR-370-3p | up | ENSRNOT00000020313 | ENSRNOG00000015077 | Acsf3 | acyl-CoA synthetase family member 3 | |
| rno-miR-665 | up | ENSRNOT00000021866 | ENSRNOG00000016291 | Marveld3 | MARVEL domain containing 3 | |
| rno-miR-370-3p | up | ENSRNOT00000066590 | ENSRNOG00000042458 | Stau2 | staufen double-stranded RNA binding protein 2 | |
| rno-miR-665 | up | ENSRNOT00000082098 | ENSRNOG00000028216 | Igsf5 | immunoglobulin superfamily, member 5 | |
| rno-miR-212-5p | up | ENSRNOT00000072819 | ENSRNOG00000050104 | Clip3 | CAP-GLY domain containing linker protein 3 | |
| rno-miR-370-3p | up | ENSRNOT00000057843 | ENSRNOG00000008785 | Klf5 | Kruppel-like factor 5 | |
| rno-miR-665 | up | ENSRNOT00000056725 | ENSRNOG00000037478 | Noc4l | nucleolar complex associated 4 homolog | |
| rno-miR-665 | up | ENSRNOT00000066128 | ENSRNOG00000038483 | Tnfrsf10b | tumor necrosis factor receptor superfamily, member 10b | |
| rno-miR-485-5p | up | ENSRNOT00000015240 | ENSRNOG00000011027 | Uba5 | ubiquitin-like modifier activating enzyme 5 | |
| rno-miR-665 | up | ENSRNOT00000039726 | ENSRNOG00000024264 | Amz1 | archaelysin family metallopeptidase 1 | |
| rno-miR-370-3p | up | ENSRNOT00000027135 | ENSRNOG00000019985 | Asic4 | acid sensing ion channel subunit family member 4 | |
| rno-miR-212-5p | up | ENSRNOT00000028247 | ENSRNOG00000020821 | LOC108348122 | CAP-Gly domain-containing linker protein 3 | |
| rno-miR-214-3p | up | ENSRNOT00000013542 | ENSRNOG00000009902 | Lrrc46 | leucine rich repeat containing 46 | |
| rno-miR-370-3p | up | ENSRNOT00000093342 | ENSRNOG00000034230 | Fcrl1 | Fc receptor-like 1 | |
| rno-miR-370-3p | up | ENSRNOT00000093330 | ENSRNOG00000037655 | Gatb | glutamyl-tRNA amidotransferase subunit B | |
| rno-miR-665 | up | ENSRNOT00000017989 | ENSRNOG00000013030 | Pip4k2b | phosphatidylinositol-5-phosphate 4-kinase type 2 beta | |
| rno-miR-665 | up | ENSRNOT00000049584 | ENSRNOG00000020443 | Tsks | testis-specific serine kinase substrate | |
| rno-miR-370-3p | up | ENSRNOT00000044844 | ENSRNOG00000019036 | Ldhd | lactate dehydrogenase D | |
| rno-miR-370-3p | up | ENSRNOT00000044467 | ENSRNOG00000033234 | Ccrl2 | C-C motif chemokine receptor like 2 | |
| rno-miR-485-5p | up | ENSRNOT00000029701 | ENSRNOG00000007440 | Dzank1 | double zinc ribbon and ankyrin repeat domains 1 | |
| rno-miR-665 | up | ENSRNOT00000076114 | ENSRNOG00000001794 | Muc13 | mucin 13, cell surface associated | |
| rno-miR-214-3p | up | ENSRNOT00000077270 | ENSRNOG00000011441 | Tpd52 | tumor protein D52 | |
| rno-miR-136-3p | up | ENSRNOT00000057062 | ENSRNOG00000016550 | Dclk2 | doublecortin-like kinase 2 | |
| rno-miR-370-3p | up | ENSRNOT00000045100 | ENSRNOG00000015210 | Ggt6 | gamma-glutamyl transferase 6 | |
| rno-miR-665 | up | ENSRNOT00000006942 | ENSRNOG00000005003 | Ptprn2 | protein tyrosine phosphatase, receptor type N2 | |
| rno-miR-370-3p | up | ENSRNOT00000022085 | ENSRNOG00000016468 | Tpmt | thiopurine S-methyltransferase | |
| rno-miR-370-3p | up | ENSRNOT00000047512 | ENSRNOG00000030775 | Adarb2 | adenosine deaminase, RNA-specific, B2 | |
| rno-miR-214-3p | up | ENSRNOT00000015206 | ENSRNOG00000023639 | Rpusd1 | RNA pseudouridylate synthase domain containing 1 | |
| rno-miR-370-3p | up | ENSRNOT00000014388 | ENSRNOG00000010841 | Col8a2 | collagen type VIII alpha 2 chain | |
| rno-miR-370-3p | up | ENSRNOT00000087322 | ENSRNOG00000010492 | Sp2 | Sp2 transcription factor | |
| rno-miR-434-5p | up | ENSRNOT00000028482 | ENSRNOG00000021794 | Znhit2 | zinc finger, HIT-type containing 2 | |
| rno-miR-214-3p | up | ENSRNOT00000007822 | ENSRNOG00000005781 | Wnt16 | wingless-type MMTV integration site family, member 16 | |
| rno-miR-665 | up | ENSRNOT00000055542 | ENSRNOG00000027869 | Sox5 | SRY box 5 | |
| rno-miR-136-3p | up | ENSRNOT00000022292 | ENSRNOG00000016550 | Dclk2 | doublecortin-like kinase 2 | |
| rno-miR-370-3p | up | ENSRNOT00000073377 | ENSRNOG00000001891 | Gnb1l | G protein subunit beta 1 like | |
| rno-miR-673-3p | up | ENSRNOT00000009870 | ENSRNOG00000006756 | Maged1 | MAGE family member D1 | |
| rno-miR-370-3p | up | ENSRNOT00000046026 | ENSRNOG00000024145 | Trim65 | tripartite motif-containing 65 | |
| rno-miR-329-3p | up | ENSRNOT00000081408 | ENSRNOG00000057180 | Gpx5 | glutathione peroxidase 5 | |
| rno-miR-665 | up | ENSRNOT00000052002 | ENSRNOG00000025923 | LOC103693776 | zinc finger MIZ domain-containing protein 2 | |
| rno-miR-370-3p | up | ENSRNOT00000066923 | ENSRNOG00000019136 | Scamp2 | secretory carrier membrane protein 2 | |
| rno-miR-665 | up | ENSRNOT00000046778 | ENSRNOG00000043414 | Fcrl3 | Fc receptor-like 3 | |
| rno-miR-370-3p | up | ENSRNOT00000079539 | ENSRNOG00000019036 | Ldhd | lactate dehydrogenase D | |
| rno-miR-140-3p | up | ENSRNOT00000000079 | ENSRNOG00000010580 | Acot7 | acyl-CoA thioesterase 7 | |
| rno-miR-127-3p | up | ENSRNOT00000044153 | ENSRNOG00000031795 | AABR07068650.1 |  | |
| rno-miR-665 | up | ENSRNOT00000028693 | ENSRNOG00000021134 | Anxa9 | annexin A9 | |
| rno-miR-665 | up | ENSRNOT00000035400 | ENSRNOG00000046452 | Fcgr2b | Fc fragment of IgG receptor IIb | |
| rno-miR-665 | up | ENSRNOT00000027454 | ENSRNOG00000020245 | Syt8 | synaptotagmin 8 | |
| rno-miR-140-3p | up | ENSRNOT00000014213 | ENSRNOG00000010580 | Acot7 | acyl-CoA thioesterase 7 | |
| rno-miR-665 | up | ENSRNOT00000028758 | ENSRNOG00000021181 | Sf3b4 | splicing factor 3b, subunit 4 | |
| rno-miR-665 | up | ENSRNOT00000082316 | ENSRNOG00000060946 | LOC100910506 | peripheral plasma membrane protein CASK-like | |
| rno-miR-370-3p | up | ENSRNOT00000031577 | ENSRNOG00000022727 | Tmem127 | transmembrane protein 127 | |
| rno-miR-370-3p | up | ENSRNOT00000076232 | ENSRNOG00000028856 | Pknox2 | PBX/knotted 1 homeobox 2 | |
| rno-miR-370-3p | up | ENSRNOT00000048875 | ENSRNOG00000028856 | Pknox2 | PBX/knotted 1 homeobox 2 | |
| rno-miR-370-3p | up | ENSRNOT00000030617 | ENSRNOG00000023700 | RGD1311847 | similar to 1700030K09Rik protein | |
| rno-miR-370-3p | up | ENSRNOT00000029508 | ENSRNOG00000027489 | Mn1 | meningioma 1 | |
| rno-miR-370-3p | up | ENSRNOT00000087857 | ENSRNOG00000016752 | Crispld2 | cysteine-rich secretory protein LCCL domain containing 2 | |
| rno-miR-370-3p | up | ENSRNOT00000091418 | ENSRNOG00000060626 | Polk | DNA polymerase kappa | |
| rno-miR-214-3p | up | ENSRNOT00000022213 | ENSRNOG00000015911 | Lrp5 | LDL receptor related protein 5 | |
| rno-miR-370-3p | up | ENSRNOT00000077253 | ENSRNOG00000059764 | Snrpn | small nuclear ribonucleoprotein polypeptide N | |
| rno-miR-127-5p | up | ENSRNOT00000028158 | ENSRNOG00000029043 | Zfp84 | zinc finger protein 84 | |
| rno-miR-370-3p | up | ENSRNOT00000083331 | ENSRNOG00000030775 | Adarb2 | adenosine deaminase, RNA-specific, B2 | |
| rno-novel-58-mature | up | ENSRNOT00000068171 | ENSRNOG00000005957 | Slc4a7 | solute carrier family 4 member 7 | |
| rno-miR-127-3p | up | ENSRNOT00000018989 | ENSRNOG00000014186 | RGD1310257 | similar to RIKEN cDNA 6330408A02 gene | |
| rno-miR-370-3p | up | ENSRNOT00000018846 | ENSRNOG00000013720 | Aebp1 | AE binding protein 1 | |
| rno-miR-370-3p | up | ENSRNOT00000004704 | ENSRNOG00000000040 | RGD1304622 | similar to 6820428L09 protein | |
| rno-miR-214-3p | up | ENSRNOT00000039730 | ENSRNOG00000008620 | Smad3 | SMAD family member 3 | |
| rno-miR-370-3p | up | ENSRNOT00000086307 | ENSRNOG00000057554 | Stk31 | serine threonine kinase 31 | |
| rno-miR-370-3p | up | ENSRNOT00000073683 | ENSRNOG00000047134 | Gtf2f1 | general transcription factor IIF subunit 1 | |
| rno-miR-673-3p | up | ENSRNOT00000083881 | ENSRNOG00000014426 | Lox | lysyl oxidase | |
| rno-miR-214-3p | up | ENSRNOT00000040498 | ENSRNOG00000042660 | LOC685081 | similar to solute carrier family 22 (organic cation transporter), member 13 | |
| rno-miR-370-3p | up | ENSRNOT00000086193 | ENSRNOG00000046272 | Hnrnpab | heterogeneous nuclear ribonucleoprotein A/B | |
| rno-miR-673-3p | up | ENSRNOT00000000707 | ENSRNOG00000000579 | Marcks | myristoylated alanine rich protein kinase C substrate | |
| rno-miR-370-3p | up | ENSRNOT00000037271 | ENSRNOG00000027015 | Ythdf1 | YTH N(6)-methyladenosine RNA binding protein 1 | |
| rno-miR-370-3p | up | ENSRNOT00000088874 | ENSRNOG00000054391 | Snurf | SNRPN upstream reading frame | |
| rno-miR-370-3p | up | ENSRNOT00000080800 | ENSRNOG00000059510 | LOC100910792 | amphiphysin-like | |
| rno-miR-665 | up | ENSRNOT00000021630 | ENSRNOG00000015904 | Wfdc1 | WAP four-disulfide core domain 1 | |
| rno-miR-370-3p | up | ENSRNOT00000026391 | ENSRNOG00000019433 | Rab3a | RAB3A, member RAS oncogene family | |
| rno-miR-673-3p | up | ENSRNOT00000073835 | ENSRNOG00000047521 | Ccdc166 | coiled-coil domain containing 166 | |
| rno-miR-127-5p | up | ENSRNOT00000078123 | ENSRNOG00000009484 | Ptcd3 | Pentatricopeptide repeat domain 3 | |
| rno-miR-370-3p | up | ENSRNOT00000081223 | ENSRNOG00000014963 | Adgrg1 | adhesion G protein-coupled receptor G1 | |
| rno-miR-370-3p | up | ENSRNOT00000020921 | ENSRNOG00000014963 | Adgrg1 | adhesion G protein-coupled receptor G1 | |
| rno-miR-370-3p | up | ENSRNOT00000022012 | ENSRNOG00000016148 | Gtse1 | G-2 and S-phase expressed 1 | |
| rno-miR-370-3p | up | ENSRNOT00000002596 | ENSRNOG00000001891 | Gnb1l | G protein subunit beta 1 like | |
| rno-miR-370-3p | up | ENSRNOT00000090097 | ENSRNOG00000002781 | Kdsr | 3-ketodihydrosphingosine reductase | |
| rno-miR-370-3p | up | ENSRNOT00000007287 | ENSRNOG00000005260 | Acp1 | acid phosphatase 1, soluble | |
| rno-miR-665 | up | ENSRNOT00000004187 | ENSRNOG00000003054 | Cask | calcium/calmodulin dependent serine protein kinase | |
| rno-miR-370-3p | up | ENSRNOT00000056192 | ENSRNOG00000037204 | Lyrm9 | LYR motif containing 9 | |
| rno-miR-214-3p | up | ENSRNOT00000078303 | ENSRNOG00000056476 | Slc22a13 | solute carrier family 22 member 13 | |
| rno-miR-673-3p | up | ENSRNOT00000084012 | ENSRNOG00000025584 | Agap2 | ArfGAP with GTPase domain, ankyrin repeat and PH domain 2 | |
| rno-miR-673-3p | up | ENSRNOT00000031230 | ENSRNOG00000025584 | Agap2 | ArfGAP with GTPase domain, ankyrin repeat and PH domain 2 | |
| rno-miR-673-3p | up | ENSRNOT00000074226 | ENSRNOG00000014426 | Lox | lysyl oxidase | |
| rno-miR-665 | up | ENSRNOT00000074079 | ENSRNOG00000046439 | NEWGENE_1309258 | VPS37C, ESCRT-I subunit | |
| rno-miR-370-3p | up | ENSRNOT00000038935 | ENSRNOG00000028121 | Otop2 | otopetrin 2 | |
| rno-miR-214-3p | up | ENSRNOT00000075041 | ENSRNOG00000050298 | Rfng | RFNG O-fucosylpeptide 3-beta-N-acetylglucosaminyltransferase | |
| rno-miR-370-3p | up | ENSRNOT00000054926 | ENSRNOG00000036669 | Uts2r | urotensin 2 receptor | |
| rno-miR-370-3p | up | ENSRNOT00000042418 | ENSRNOG00000011614 | Tmcc1 | transmembrane and coiled-coil domain family 1 | |
| rno-miR-370-3p | up | ENSRNOT00000028177 | ENSRNOG00000020743 | Cyp2s1 | cytochrome P450, family 2, subfamily s, polypeptide 1 | |
| rno-miR-370-3p | up | ENSRNOT00000014568 | ENSRNOG00000010883 | Pard6b | par-6 family cell polarity regulator beta | |
| rno-miR-665 | up | ENSRNOT00000084270 | ENSRNOG00000046439 | NEWGENE_1309258 | VPS37C, ESCRT-I subunit | |
| rno-miR-127-3p | up | ENSRNOT00000002843 | ENSRNOG00000002075 | Cnot6l | CCR4-NOT transcription complex, subunit 6-like | |
| rno-miR-665 | up | ENSRNOT00000025084 | ENSRNOG00000018553 | Pitpnm1 | phosphatidylinositol transfer protein, membrane-associated 1 | |
| rno-miR-370-3p | up | ENSRNOT00000014532 | ENSRNOG00000010434 | Dync1li1 | dynein cytoplasmic 1 light intermediate chain 1 | |
| rno-miR-370-3p | up | ENSRNOT00000092122 | ENSRNOG00000053285 | Mllt6 | MLLT6, PHD finger domain containing | |
| rno-miR-370-3p | up | ENSRNOT00000027564 | ENSRNOG00000020342 | Samd11 | sterile alpha motif domain containing 11 | |
| rno-miR-214-3p | up | ENSRNOT00000067532 | ENSRNOG00000007486 | Atg7 | autophagy related 7 | |
| rno-miR-370-3p | up | ENSRNOT00000068389 | ENSRNOG00000016752 | Crispld2 | cysteine-rich secretory protein LCCL domain containing 2 | |
| rno-miR-370-3p | up | ENSRNOT00000037997 | ENSRNOG00000022104 | Ccdc185 | coiled-coil domain containing 185 | |
| rno-miR-370-3p | up | ENSRNOT00000064924 | ENSRNOG00000047281 | Hdac6 | histone deacetylase 6 | |
| rno-miR-212-5p | up | ENSRNOT00000074517 | ENSRNOG00000048050 | Tmem120b | transmembrane protein 120B | |
| rno-miR-370-3p | up | ENSRNOT00000029723 | ENSRNOG00000042201 | Efcab2 | EF-hand calcium binding domain 2 | |
| rno-miR-665 | up | ENSRNOT00000032566 | ENSRNOG00000028216 | Igsf5 | immunoglobulin superfamily, member 5 | |
| rno-miR-212-5p | up | ENSRNOT00000085517 | ENSRNOG00000051619 | Asb2 | ankyrin repeat and SOCS box-containing 2 | |
| rno-miR-214-3p | up | ENSRNOT00000023649 | ENSRNOG00000017585 | Abt1 | activator of basal transcription 1 | |
| rno-miR-665 | up | ENSRNOT00000001918 | ENSRNOG00000001415 | Ap1s1 | adaptor-related protein complex 1, sigma 1 subunit | |
| rno-miR-127-5p | up | ENSRNOT00000017654 | ENSRNOG00000013196 | Dok5 | docking protein 5 | |
| rno-miR-370-3p | up | ENSRNOT00000039540 | ENSRNOG00000003494 | Ppfia4 | PTPRF interacting protein alpha 4 | |
| rno-miR-127-3p | up | ENSRNOT00000015866 | ENSRNOG00000011875 | Slc2a2 | solute carrier family 2 member 2 | |
| rno-miR-485-5p | up | ENSRNOT00000005920 | ENSRNOG00000004443 | Sptlc3 | serine palmitoyltransferase, long chain base subunit 3 | |
| rno-miR-370-3p | up | ENSRNOT00000077442 | ENSRNOG00000005260 | Acp1 | acid phosphatase 1, soluble | |
| rno-miR-665 | up | ENSRNOT00000091541 | ENSRNOG00000046452 | Fcgr2b | Fc fragment of IgG receptor IIb | |
| rno-miR-370-3p | up | ENSRNOT00000025415 | ENSRNOG00000018322 | Picalm | phosphatidylinositol binding clathrin assembly protein | |
| rno-miR-370-3p | up | ENSRNOT00000070978 | ENSRNOG00000048834 | Plin3 | perilipin 3 | |
| rno-miR-370-3p | up | ENSRNOT00000079488 | ENSRNOG00000046515 | St6gal2 | ST6 beta-galactoside alpha-2,6-sialyltransferase 2 | |
| rno-miR-370-3p | up | ENSRNOT00000018340 | ENSRNOG00000013039 | Add1 | adducin 1 | |
| rno-miR-370-3p | up | ENSRNOT00000017102 | ENSRNOG00000012490 | Amph | amphiphysin | |
| rno-miR-370-3p | up | ENSRNOT00000075823 | ENSRNOG00000037655 | Gatb | glutamyl-tRNA amidotransferase subunit B | |
| rno-miR-370-3p | up | ENSRNOT00000003567 | ENSRNOG00000002525 | Ptgs2 | prostaglandin-endoperoxide synthase 2 | |
| rno-miR-370-3p | up | ENSRNOT00000004218 | ENSRNOG00000021287 | Hexim2 | hexamethylene bis-acetamide inducible 2 | |
| rno-miR-370-3p | up | ENSRNOT00000065890 | ENSRNOG00000000302 | Sesn1 | sestrin 1 | |
| rno-miR-370-3p | up | ENSRNOT00000064113 | ENSRNOG00000010497 | RGD1305807 | hypothetical LOC298077 | |
| rno-miR-370-3p | up | ENSRNOT00000016884 | ENSRNOG00000012652 | RGD1565693 | similar to GLE1-like, RNA export mediator isoform 1 | |
| rno-miR-212-5p | up | ENSRNOT00000082174 | ENSRNOG00000019638 | Lmna | lamin A/C | |
| rno-miR-212-5p | up | ENSRNOT00000065989 | ENSRNOG00000004614 | Lypd6b | LY6/PLAUR domain containing 6B | |
| rno-miR-665 | up | ENSRNOT00000058497 | ENSRNOG00000046452 | Fcgr2b | Fc fragment of IgG receptor IIb | |
| rno-miR-212-5p | up | ENSRNOT00000026705 | ENSRNOG00000019638 | Lmna | lamin A/C | |
| rno-miR-665 | up | ENSRNOT00000006319 | ENSRNOG00000004645 | Galnt5 | polypeptide N-acetylgalactosaminyltransferase 5 | |
| rno-miR-370-3p | up | ENSRNOT00000027891 | ENSRNOG00000020552 | Fosl1 | FOS like 1, AP-1 transcription factor subunit | |
| rno-miR-140-3p | up | ENSRNOT00000049344 | ENSRNOG00000032942 | Neu1 | neuraminidase 1 | |
| rno-miR-665 | up | ENSRNOT00000015678 | ENSRNOG00000011718 | C1rl | complement C1r subcomponent like | |
| rno-miR-370-3p | up | ENSRNOT00000091470 | ENSRNOG00000018322 | Picalm | phosphatidylinositol binding clathrin assembly protein | |
| rno-miR-370-3p | up | ENSRNOT00000092086 | ENSRNOG00000018322 | Picalm | phosphatidylinositol binding clathrin assembly protein | |
| rno-miR-370-3p | up | ENSRNOT00000057833 | ENSRNOG00000006946 | Arhgap9 | Rho GTPase activating protein 9 | |
| rno-miR-673-3p | up | ENSRNOT00000044690 | ENSRNOG00000033609 | Irx1 | iroquois homeobox 1 | |
| rno-miR-370-3p | up | ENSRNOT00000038349 | ENSRNOG00000024111 | Cage1 | cancer antigen 1 | |
| rno-miR-370-3p | up | ENSRNOT00000074251 | ENSRNOG00000048288 | Taf12 | TATA-box binding protein associated factor 12 | |
| rno-miR-673-3p | up | ENSRNOT00000028963 | ENSRNOG00000027799 | Tmie | transmembrane inner ear | |
| rno-miR-665 | up | ENSRNOT00000024375 | ENSRNOG00000017887 | Mutyh | mutY DNA glycosylase | |
| rno-miR-370-3p | up | ENSRNOT00000025075 | ENSRNOG00000037165 | Nipal3 | NIPA-like domain containing 3 | |
| rno-miR-665 | up | ENSRNOT00000040002 | ENSRNOG00000014052 | Ccna1 | cyclin A1 | |
| rno-miR-370-3p | up | ENSRNOT00000034798 | ENSRNOG00000025238 | Bpifb5 | BPI fold containing family B, member 5 | |
| rno-miR-665 | up | ENSRNOT00000063783 | ENSRNOG00000018166 | Prkab2 | protein kinase AMP-activated non-catalytic subunit beta 2 | |
| rno-miR-214-3p | up | ENSRNOT00000088079 | ENSRNOG00000000437 | Agpat1 | 1-acylglycerol-3-phosphate O-acyltransferase 1 | |
| rno-miR-1193-3p | up | ENSRNOT00000026049 | ENSRNOG00000019195 | Polh | DNA polymerase eta | |
| rno-miR-370-3p | up | ENSRNOT00000022814 | ENSRNOG00000017017 | Zbtb43 | zinc finger and BTB domain containing 43 | |
| rno-miR-370-3p | up | ENSRNOT00000018073 | ENSRNOG00000013039 | Add1 | adducin 1 | |
| rno-miR-485-5p | up | ENSRNOT00000090181 | ENSRNOG00000018020 | Apbb1 | amyloid beta precursor protein binding family B member 1 | |
| rno-miR-370-3p | up | ENSRNOT00000046882 | ENSRNOG00000032684 | Ccdc60 | coiled-coil domain containing 60 | |
| rno-miR-127-5p | up | ENSRNOT00000081997 | ENSRNOG00000056617 | Zswim8 | zinc finger, SWIM-type containing 8 | |
| rno-miR-370-3p | up | ENSRNOT00000018976 | ENSRNOG00000014172 | Npy5r | neuropeptide Y receptor Y5 | |
| rno-miR-370-3p | up | ENSRNOT00000072696 | ENSRNOG00000049952 | Gucd1 | guanylyl cyclase domain containing 1 | |
| rno-miR-370-3p | up | ENSRNOT00000002291 | ENSRNOG00000001686 | Hlcs | holocarboxylase synthetase | |
| rno-miR-370-3p | up | ENSRNOT00000010864 | ENSRNOG00000008234 | Lrrc61 | leucine rich repeat containing 61 | |
| rno-miR-370-3p | up | ENSRNOT00000012699 | ENSRNOG00000009585 | Tcf20 | transcription factor 20 | |
| rno-miR-127-5p | up | ENSRNOT00000011675 | ENSRNOG00000008678 | Antxr1 | anthrax toxin receptor 1 | |
| rno-miR-370-3p | up | ENSRNOT00000089171 | ENSRNOG00000020369 | Igf2 | insulin-like growth factor 2 | |
| rno-miR-370-3p | up | ENSRNOT00000075201 | ENSRNOG00000047295 | Prr22 | proline rich 22 | |
| rno-miR-673-3p | up | ENSRNOT00000018359 | ENSRNOG00000013717 | Bmp6 | bone morphogenetic protein 6 | |
| rno-miR-370-3p | up | ENSRNOT00000022685 | ENSRNOG00000016894 | Dnd1 | DND microRNA-mediated repression inhibitor 1 | |
| rno-miR-370-3p | up | ENSRNOT00000077544 | ENSRNOG00000014486 | Rfx3 | regulatory factor X3 | |
| rno-miR-370-3p | up | ENSRNOT00000012317 | ENSRNOG00000009028 | Rnf126 | ring finger protein 126 | |
| rno-miR-214-3p | up | ENSRNOT00000018734 | ENSRNOG00000013816 | Cmas | cytidine monophosphate N-acetylneuraminic acid synthetase | |
| rno-miR-370-3p | up | ENSRNOT00000008113 | ENSRNOG00000006146 | Trim54 | tripartite motif-containing 54 | |
| rno-miR-665 | up | ENSRNOT00000027478 | ENSRNOG00000020289 | Akt1s1 | AKT1 substrate 1 催促 | |
| rno-miR-370-3p | up | ENSRNOT00000056808 | ENSRNOG00000037514 | Qser1 | glutamine and serine rich 1 | |
| rno-miR-370-3p | up | ENSRNOT00000028405 | ENSRNOG00000020925 | Ccdc86 | coiled-coil domain containing 86 | |
| rno-miR-370-3p | up | ENSRNOT00000019799 | ENSRNOG00000014193 | Lig1 | DNA ligase 1 | |
| rno-miR-370-3p | up | ENSRNOT00000084268 | ENSRNOG00000018322 | Picalm | phosphatidylinositol binding clathrin assembly protein ] | |
| rno-miR-370-3p | up | ENSRNOT00000025624 | ENSRNOG00000018870 | Hapln2 | hyaluronan and proteoglycan link protein 2 | |
| rno-miR-370-3p | up | ENSRNOT00000011984 | ENSRNOG00000009001 | Mtss1 | MTSS1, I-BAR domain containing | |
| rno-miR-370-3p | up | ENSRNOT00000083552 | ENSRNOG00000053122 | Scn1a | sodium voltage-gated channel alpha subunit 1 | |
| rno-miR-370-3p | up | ENSRNOT00000078393 | ENSRNOG00000021139 | Esrra | estrogen related receptor, alpha | |
| rno-miR-370-3p | up | ENSRNOT00000011599 | ENSRNOG00000008754 | Flvcr2 | feline leukemia virus subgroup C cellular receptor family, member 2 | |
| rno-miR-341 | up | ENSRNOT00000005731 | ENSRNOG00000004296 | Nt5c1b | 5'-nucleotidase, cytosolic IB | |
| rno-miR-370-3p | up | ENSRNOT00000008757 | ENSRNOG00000043151 | Cntln | centlein | |
| rno-miR-665 | up | ENSRNOT00000091003 | ENSRNOG00000051770 | Cyp2w1 | cytochrome P450, family 2, subfamily w, polypeptide 1 | |
| rno-miR-370-3p | up | ENSRNOT00000025011 | ENSRNOG00000028168 | Ankrd16 | ankyrin repeat domain 16 | |
| rno-miR-665 | up | ENSRNOT00000077797 | ENSRNOG00000025892 | Set | SET nuclear proto-oncogene | |
| rno-miR-370-3p | up | ENSRNOT00000077193 | ENSRNOG00000024693 | Zfp831 | zinc finger protein 831 | |
| rno-miR-127-3p | up | ENSRNOT00000090539 | ENSRNOG00000061298 | LOC102555328 | uncharacterized LOC102555328 | |
| rno-miR-665 | up | ENSRNOT00000082241 | ENSRNOG00000010005 | Adck2 | aarF domain containing kinase 2 | |
| rno-miR-370-3p | up | ENSRNOT00000019873 | ENSRNOG00000014805 | Wrap73 | WD repeat containing, antisense to TP73 | |
| rno-miR-370-3p | up | ENSRNOT00000003076 | ENSRNOG00000002258 | Tmem150c | transmembrane protein 150C | |
| rno-miR-541-5p | up | ENSRNOT00000023591 | ENSRNOG00000017487 | Otoa | otoancorin | |
| rno-miR-370-3p | up | ENSRNOT00000035558 | ENSRNOG00000026497 | Pigc | phosphatidylinositol glycan anchor biosynthesis, class C | |
| rno-miR-370-3p | up | ENSRNOT00000068083 | ENSRNOG00000010492 | Sp2 | Sp2 transcription factor | |
| rno-miR-673-3p | up | ENSRNOT00000023202 | ENSRNOG00000016870 | Pcif1 | PDX1 C-terminal inhibiting factor 1 | |
| rno-miR-127-3p | up | ENSRNOT00000049985 | ENSRNOG00000001074 | Mphosph9 | M-phase phosphoprotein 9 | |
| rno-miR-370-3p | up | ENSRNOT00000078639 | ENSRNOG00000060626 | Polk | DNA polymerase kappa | |
| rno-miR-127-5p | up | ENSRNOT00000023521 | ENSRNOG00000017290 | Zfp335 | zinc finger protein 335 | |
| rno-miR-665 | up | ENSRNOT00000080223 | ENSRNOG00000008332 | Smo | smoothened, frizzled class receptor | |
| rno-miR-370-3p | up | ENSRNOT00000001466 | ENSRNOG00000001104 | Foxk1 | forkhead box K1 | |
| rno-miR-665 | up | ENSRNOT00000007763 | ENSRNOG00000005301 | Eif2b4 | eukaryotic translation initiation factor 2B subunit delta | |
| rno-miR-214-3p | up | ENSRNOT00000046059 | ENSRNOG00000032569 | Lingo3 | leucine rich repeat and Ig domain containing 3 | |
| rno-miR-140-3p | up | ENSRNOT00000052202 | ENSRNOG00000000665 | Pitpnb | phosphatidylinositol transfer protein, beta | |
| rno-miR-370-3p | up | ENSRNOT00000080246 | ENSRNOG00000020369 | Igf2 | insulin-like growth factor 2 |  |
| rno-miR-370-3p | up | ENSRNOT00000079466 | ENSRNOG00000022482 | Trappc11 | trafficking protein particle complex 11 |  |
| rno-miR-665 | up | ENSRNOT00000090679 | ENSRNOG00000051911 | Rbp3 | retinol binding protein 3 |  |
| rno-miR-140-3p | up | ENSRNOT00000027054 | ENSRNOG00000019955 | Ogdhl | oxoglutarate dehydrogenase-like |  |
| rno-miR-127-5p | up | ENSRNOT00000027135 | ENSRNOG00000019985 | Asic4 | acid sensing ion channel subunit family member 4 |  |
| rno-miR-665 | up | ENSRNOT00000077898 | ENSRNOG00000037190 | Cd101 | CD101 molecule |  |
| rno-miR-673-3p | up | ENSRNOT00000087134 | ENSRNOG00000000007 | Gad1 | glutamate decarboxylase 1 |  |
| rno-miR-665 | up | ENSRNOT00000056140 | ENSRNOG00000037190 | Cd101 | CD101 molecule |  |
| rno-miR-665 | up | ENSRNOT00000086588 | ENSRNOG00000018166 | Prkab2 | protein kinase AMP-activated non-catalytic subunit beta 2 |  |
| rno-miR-127-3p | up | ENSRNOT00000028217 | ENSRNOG00000020793 | Ruvbl2 | RuvB-like AAA ATPase 2 |  |
| rno-miR-370-3p | up | ENSRNOT00000081681 | ENSRNOG00000048288 | Taf12 | TATA-box binding protein associated factor 12 |  |
| rno-miR-370-3p | up | ENSRNOT00000019930 | ENSRNOG00000014642 | Morn1 | MORN repeat containing 1 |  |
| rno-miR-665 | up | ENSRNOT00000079676 | ENSRNOG00000061612 | Zfp184 | zinc finger protein 184 |  |
| rno-miR-673-3p | up | ENSRNOT00000032918 | ENSRNOG00000023509 | Irs2 | insulin receptor substrate 2 |  |
| rno-miR-665 | up | ENSRNOT00000056955 | ENSRNOG00000023931 | Ggn | gametogenetin |  |
| rno-miR-665 | up | ENSRNOT00000035002 | ENSRNOG00000025832 | Zer1 | zyg-11 related, cell cycle regulator |  |
| rno-miR-485-5p | up | ENSRNOT00000051685 | ENSRNOG00000031661 | Zfp839 | zinc finger protein 839 |  |
| rno-miR-214-3p | up | ENSRNOT00000084567 | ENSRNOG00000018666 | Gpsm1 | G-protein signaling modulator 1 |  |
| rno-miR-370-3p | up | ENSRNOT00000073470 | ENSRNOG00000047026 | Enpp7 | ectonucleotide pyrophosphatase/phosphodiesterase 7 |  |
| rno-miR-665 | up | ENSRNOT00000020229 | ENSRNOG00000015003 | Pex11a | peroxisomal biogenesis factor 11 alpha |  |
| rno-miR-212-5p | up | ENSRNOT00000025570 | ENSRNOG00000018668 | Glg1 | golgi glycoprotein 1 |  |
| rno-miR-337-5p | up | ENSRNOT00000082486 | ENSRNOG00000019924 | Thop1 | thimet oligopeptidase 1 |  |
| rno-miR-127-3p | up | ENSRNOT00000003573 | ENSRNOG00000002660 | Magee1 | MAGE family member E1 |  |
| rno-miR-665 | up | ENSRNOT00000013277 | ENSRNOG00000010005 | Adck2 | aarF domain containing kinase 2 |  |
| rno-miR-370-3p | up | ENSRNOT00000025193 | ENSRNOG00000018655 | Adsl | adenylosuccinate lyase |  |
| rno-miR-665 | up | ENSRNOT00000028642 | ENSRNOG00000021100 | Tnfaip8l2 | TNF alpha induced protein 8 like 2 |  |
| rno-miR-370-3p | up | ENSRNOT00000080024 | ENSRNOG00000042978 | Ncald | neurocalcin delta |  |
| rno-miR-665 | up | ENSRNOT00000018620 | ENSRNOG00000013594 | Acp2 | acid phosphatase 2, lysosomal |  |
| rno-miR-485-5p | up | ENSRNOT00000012222 | ENSRNOG00000008557 | Abcb8 | ATP binding cassette subfamily B member 8 |  |
| rno-miR-673-3p | up | ENSRNOT00000073933 | ENSRNOG00000045553 | Proser2 | proline and serine rich 2 |  |
| rno-miR-370-3p | up | ENSRNOT00000065182 | ENSRNOG00000020908 | LOC303566 | E2F1-inducible gene |  |
| rno-miR-370-3p | up | ENSRNOT00000085191 | ENSRNOG00000052296 | Shank3 | SH3 and multiple ankyrin repeat domains 3 |  |
| rno-miR-370-3p | up | ENSRNOT00000024460 | ENSRNOG00000018163 | Ipcef1 | interaction protein for cytohesin exchange factors 1 |  |
| rno-miR-341 | up | ENSRNOT00000076022 | ENSRNOG00000049768 | Adcy9 | adenylate cyclase 9 |  |
| rno-miR-665 | up | ENSRNOT00000082982 | ENSRNOG00000053203 | Mical3 | microtubule associated monooxygenase, calponin and LIM domain containing 3 |  |
| rno-miR-673-3p | up | ENSRNOT00000071754 | ENSRNOG00000060005 | Surf4 | surfeit 4 |  |
| rno-miR-370-3p | up | ENSRNOT00000009295 | ENSRNOG00000048738 | LOC108348065 | histone deacetylase 6 |  |
| rno-miR-370-3p | up | ENSRNOT00000019368 | ENSRNOG00000014373 | Trim66 | tripartite motif-containing 66 |  |
| rno-miR-370-3p | up | ENSRNOT00000064513 | ENSRNOG00000042978 | Ncald | neurocalcin delta |  |
| rno-novel-58-mature | up | ENSRNOT00000028069 | ENSRNOG00000020644 | Nsg2 | neuron specific gene family member 2 |  |
| rno-miR-370-3p | up | ENSRNOT00000028697 | ENSRNOG00000021139 | Esrra | estrogen related receptor, alpha |  |
| rno-miR-127-3p | up | ENSRNOT00000003207 | ENSRNOG00000002345 | Rasgef1b | RasGEF domain family, member 1B |  |
| rno-miR-370-3p | up | ENSRNOT00000033489 | ENSRNOG00000002251 | Sec31a | SEC31 homolog A, COPII coat complex component |  |
| rno-miR-665 | up | ENSRNOT00000014611 | ENSRNOG00000028033 | Smptb | polypyrimidine tract-binding protein |  |
| rno-miR-665 | up | ENSRNOT00000074595 | ENSRNOG00000046307 | Glyctk | glycerate kinase |  |
| rno-miR-370-3p | up | ENSRNOT00000089434 | ENSRNOG00000007925 | Pak6 | p21 (RAC1) activated kinase 6 |  |
| rno-miR-370-3p | up | ENSRNOT00000084702 | ENSRNOG00000012490 | Amph | amphiphysin |  |
| rno-miR-370-3p | up | ENSRNOT00000039852 | ENSRNOG00000028512 | Ilvbl | ilvB acetolactate synthase like |  |
| rno-miR-665 | up | ENSRNOT00000038879 | ENSRNOG00000021824 | Dnajb1 | DnaJ heat shock protein family (Hsp40) member B1 |  |
| rno-miR-370-3p | up | ENSRNOT00000066303 | ENSRNOG00000003239 | Cant1 | calcium activated nucleotidase 1 |  |
| rno-miR-370-3p | up | ENSRNOT00000087978 | ENSRNOG00000056343 | Lats2 | large tumor suppressor kinase 2 |  |
| rno-miR-541-3p | up | ENSRNOT00000015116 | ENSRNOG00000011360 | Dkk2 | dickkopf WNT signaling pathway inhibitor 2 |  |
| rno-miR-370-3p | up | ENSRNOT00000010113 | ENSRNOG00000007645 | Kcnj9 | potassium voltage-gated channel subfamily J member 9 |  |
| rno-miR-370-3p | up | ENSRNOT00000035427 | ENSRNOG00000028108 | Cytl1 | cytokine like 1 |  |
| rno-miR-673-3p | up | ENSRNOT00000008962 | ENSRNOG00000006824 | Hdac11 | histone deacetylase 11 |  |
| rno-miR-370-3p | up | ENSRNOT00000001054 | ENSRNOG00000000805 | Gja1 | gap junction protein, alpha 1 |  |
| rno-miR-541-3p | up | ENSRNOT00000064526 | ENSRNOG00000000961 | Glt1d1 | glycosyltransferase 1 domain containing 1 |  |
| rno-miR-673-3p | up | ENSRNOT00000012147 | ENSRNOG00000009058 | Spata21 | spermatogenesis associated 21 |  |
| rno-miR-127-3p | up | ENSRNOT00000076740 | ENSRNOG00000012184 | Urgcp | upregulator of cell proliferation |  |
| rno-miR-673-3p | up | ENSRNOT00000091994 | ENSRNOG00000002983 | Nf1x | nuclear factor 1 X |  |
| rno-miR-214-3p | up | ENSRNOT00000092123 | ENSRNOG00000057620 | Slc6a8 | solute carrier family 6 member 8 |  |
| rno-miR-431 | up | ENSRNOT00000015169 | ENSRNOG00000011373 | Reep4 | receptor accessory protein 4 |  |
| rno-miR-212-5p | up | ENSRNOT00000003274 | ENSRNOG00000002396 | Serpinb8 | serpin family B member 8 |  |
| rno-miR-485-5p | up | ENSRNOT00000043686 | ENSRNOG00000033540 | Cts7 | cathepsin 7 |  |
| rno-miR-665 | up | ENSRNOT00000057471 | ENSRNOG00000007327 | Pars2 | prolyl-tRNA synthetase 2, mitochondrial (putative) |  |
| rno-miR-370-3p | up | ENSRNOT00000079411 | ENSRNOG00000059485 | Cdkl3 | cyclin-dependent kinase-like 3 |  |
| rno-miR-127-3p | up | ENSRNOT00000085191 | ENSRNOG00000052296 | Shank3 | SH3 and multiple ankyrin repeat domains 3 |  |
| rno-miR-370-3p | up | ENSRNOT00000018969 | ENSRNOG00000022482 | Trappc11 | trafficking protein particle complex 11 |  |
| rno-miR-370-3p | up | ENSRNOT00000080965 | ENSRNOG00000020908 | LOC303566 | E2F1-inducible gene |  |
| rno-miR-665 | up | ENSRNOT00000046898 | ENSRNOG00000008102 | Dph7 | diphthamide biosynthesis 7 |  |
| rno-miR-433-3p | up | ENSRNOT00000092026 | ENSRNOG00000061876 | Tas1r2 | taste 1 receptor member 2 |  |
| rno-miR-665 | up | ENSRNOT00000019501 | ENSRNOG00000014350 | Cyr61 | cysteine-rich, angiogenic inducer, 61 |  |
| rno-miR-665 | up | ENSRNOT00000079106 | ENSRNOG00000021824 | Dnajb1 | DnaJ heat shock protein family (Hsp40) member B1 |  |
| rno-miR-409a-5p | up | ENSRNOT00000083366 | ENSRNOG00000002369 | Rgs8 | regulator of G-protein signaling 8 |  |
| rno-miR-370-3p | up | ENSRNOT00000036223 | ENSRNOG00000021886 | B4galt7 | beta-1,4-galactosyltransferase 7 |  |
| rno-miR-370-3p | up | ENSRNOT00000003555 | ENSRNOG00000002630 | Cnot8 | CCR4-NOT transcription complex, subunit 8 |  |
| rno-miR-665 | up | ENSRNOT00000056722 | ENSRNOG00000037476 | Galnt9 | polypeptide N-acetylgalactosaminyltransferase 9 |  |
| rno-miR-370-3p | up | ENSRNOT00000051910 | ENSRNOG00000019023 | Tars | threonyl-tRNA synthetase |  |
| rno-miR-127-3p | up | ENSRNOT00000076874 | ENSRNOG00000012184 | Urgcp | upregulator of cell proliferation |  |
| rno-miR-127-5p | up | ENSRNOT00000000106 | ENSRNOG00000000095 | Gpr89b | G protein-coupled receptor 89B |  |
| rno-miR-673-3p | up | ENSRNOT00000019844 | ENSRNOG00000014426 | Lox | lysyl oxidase |  |
| rno-miR-214-3p | up | ENSRNOT00000037399 | ENSRNOG00000013442 | Ciz1 | CDKN1A interacting zinc finger protein 1 |  |
| rno-miR-665 | up | ENSRNOT00000090318 | ENSRNOG00000024264 | Amz1 | archaelysin family metallopeptidase 1 |  |
| rno-miR-370-3p | up | ENSRNOT00000017424 | ENSRNOG00000012323 | Cherp | calcium homeostasis endoplasmic reticulum protein |  |
| rno-miR-370-3p | up | ENSRNOT00000019991 | ENSRNOG00000014712 | Zfp39 | zinc finger protein 39 |  |
| rno-miR-370-3p | up | ENSRNOT00000015799 | ENSRNOG00000011826 | Lzts1 | leucine zipper tumor suppressor 1 |  |
| rno-miR-485-5p | up | ENSRNOT00000009963 | ENSRNOG00000007261 | Gli2 | GLI family zinc finger 2 |  |
| rno-miR-485-5p | up | ENSRNOT00000089817 | ENSRNOG00000008557 | Abcb8 | ATP binding cassette subfamily B member 8 |  |
| rno-miR-433-3p | up | ENSRNOT00000079516 | ENSRNOG00000061876 | Tas1r2 | taste 1 receptor member 2 |  |
| rno-miR-665 | up | ENSRNOT00000083363 | ENSRNOG00000017610 | Nedd4l | neural precursor cell expressed, developmentally down-regulated 4-like, E3 ubiquitin protein ligase |  |
| rno-miR-370-3p | up | ENSRNOT00000001373 | ENSRNOG00000001039 | Eif2b1 | eukaryotic translation initiation factor 2B subunit 1 alpha |  |
| rno-miR-370-3p | up | ENSRNOT00000092416 | ENSRNOG00000001039 | Eif2b1 | eukaryotic translation initiation factor 2B subunit 1 alpha |  |
| rno-miR-370-3p | up | ENSRNOT00000025392 | ENSRNOG00000018784 | Jph3 | junctophilin 3 |  |
| rno-miR-665 | up | ENSRNOT00000084144 | ENSRNOG00000057881 | Sppl2b | signal peptide peptidase-like 2B |  |
| rno-miR-370-3p | up | ENSRNOT00000060461 | ENSRNOG00000039454 | Pcdhgb7 | protocadherin gamma subfamily B, 7 |  |
| rno-miR-665 | up | ENSRNOT00000071130 | ENSRNOG00000045626 | Pkd1l2 | polycystic kidney disease 1-like 2 |  |
| rno-miR-127-5p | up | ENSRNOT00000034472 | ENSRNOG00000025791 | Dync1li2 | dynein, cytoplasmic 1 light intermediate chain 2 |  |
| rno-miR-665 | up | ENSRNOT00000017960 | ENSRNOG00000013216 | Usb1 | U6 snRNA biogenesis phosphodiesterase 1 |  |
| rno-miR-673-3p | up | ENSRNOT00000000008 | ENSRNOG00000000007 | Gad1 | glutamate decarboxylase 1 |  |
| rno-miR-665 | up | ENSRNOT00000055016 | ENSRNOG00000027213 | Asphd1 | aspartate beta-hydroxylase domain containing 1 |  |
| rno-miR-370-3p | up | ENSRNOT00000019258 | ENSRNOG00000014340 | Pdcd7 | programmed cell death 7 |  |
| rno-miR-370-3p | up | ENSRNOT00000009081 | ENSRNOG00000006855 | Padi4 | peptidyl arginine deiminase 4 |  |
| rno-miR-673-3p | up | ENSRNOT00000058206 | ENSRNOG00000001030 | Tsc22d1 | TSC22 domain family, member 1 |  |
| rno-miR-370-3p | up | ENSRNOT00000092945 | ENSRNOG00000018322 | Picalm | phosphatidylinositol binding clathrin assembly protein |  |
| rno-miR-370-3p | up | ENSRNOT00000067655 | ENSRNOG00000010381 | Mknk1 | MAP kinase-interacting serine/threonine kinase 1 |  |
| rno-miR-370-3p | up | ENSRNOT00000031549 | ENSRNOG00000025808 | Aars2 | alanyl-tRNA synthetase 2, mitochondrial |  |
| rno-miR-214-3p | up | ENSRNOT00000023418 | ENSRNOG00000023453 | Lrba | LPS responsive beige-like anchor protein |  |
| rno-miR-370-3p | up | ENSRNOT00000052131 | ENSRNOG00000030328 | Fam98a | family with sequence similarity 98, member A |  |
| rno-miR-370-3p | up | ENSRNOT00000022014 | ENSRNOG00000016273 | Fam136a | family with sequence similarity 136, member A |  |
| rno-miR-370-3p | up | ENSRNOT00000030658 | ENSRNOG00000025796 | RGD1306072 | hypothetical LOC304654 |  |
| rno-novel-58-mature | up | ENSRNOT00000010852 | ENSRNOG00000008182 | Htra3 | HtrA serine peptidase 3 |  |
| rno-miR-127-5p | up | ENSRNOT00000079841 | ENSRNOG00000060542 | Mid2 | midline 2 |  |
| rno-miR-541-5p | up | ENSRNOT00000077863 | ENSRNOG00000061876 | Tas1r2 | taste 1 receptor member 2 |  |
| rno-miR-31a-5p | up | ENSRNOT00000081738 | ENSRNOG00000059851 | Nxpe4 | neurexophilin and PC-esterase domain family, member 4 |  |
| rno-miR-370-3p | up | ENSRNOT00000028081 | ENSRNOG00000020669 | Ovol1 | ovo like transcriptional repressor 1 |  |
| rno-miR-370-3p | up | ENSRNOT00000016070 | ENSRNOG00000011475 | Srcin1 | SRC kinase signaling inhibitor 1 |  |
| rno-miR-136-3p | up | ENSRNOT00000066004 | ENSRNOG00000016550 | Dclk2 | doublecortin-like kinase 2 |  |
| rno-miR-136-3p | up | ENSRNOT00000079954 | ENSRNOG00000016550 | Dclk2 | doublecortin-like kinase 2 |  |
| rno-miR-370-3p | up | ENSRNOT00000006827 | ENSRNOG00000004940 | Rnf215 | ring finger protein 215 |  |
| rno-miR-127-3p | up | ENSRNOT00000090506 | ENSRNOG00000057556 | Pdzrn3 | PDZ domain containing RING finger 3 |  |
| rno-miR-341 | up | ENSRNOT00000090438 | ENSRNOG00000004296 | Nt5c1b | 5'-nucleotidase, cytosolic IB |  |
| rno-miR-370-3p | up | ENSRNOT00000000124 | ENSRNOG00000000112 | Champ1 | chromosome alignment maintaining phosphoprotein 1 |  |
| rno-miR-370-3p | up | ENSRNOT00000082241 | ENSRNOG00000010005 | Adck2 | aarF domain containing kinase 2 |  |
| rno-miR-673-3p | up | ENSRNOT00000042394 | ENSRNOG00000016763 | Fgfr4 | fibroblast growth factor receptor 4 |  |
| rno-miR-370-3p | up | ENSRNOT00000055320 | ENSRNOG00000010793 | Spryd3 | SPRY domain containing 3 |  |
| rno-miR-665 | up | ENSRNOT00000006946 | ENSRNOG00000005228 | Gnptab | N-acetylglucosamine-1-phosphate transferase, alpha and beta subunits |  |
| rno-miR-140-3p | up | ENSRNOT00000019545 | ENSRNOG00000049336 | Lime1 | Lck interacting transmembrane adaptor 1 |  |
| rno-miR-370-3p | up | ENSRNOT00000041207 | ENSRNOG00000006132 | Fam71f1 | family with sequence similarity 71, member F1 |  |
| rno-miR-132-3p | up | ENSRNOT00000014028 | ENSRNOG00000010407 | LOC103690024 | peroxisomal targeting signal 1 receptor-like |  |
| rno-miR-140-3p | up | ENSRNOT00000026331 | ENSRNOG00000019463 | Mad2l1bp | MAD2L1 binding protein |  |
| rno-miR-370-3p | up | ENSRNOT00000019528 | ENSRNOG00000014461 | Galns | galactosamine (N-acetyl)-6-sulfatase |  |
| rno-miR-673-3p | up | ENSRNOT00000004638 | ENSRNOG00000003457 | Bicdl2 | BICD family like cargo adaptor 2 |  |
| rno-miR-370-3p | up | ENSRNOT00000090834 | ENSRNOG00000052873 | Npnt | nephronectin |  |
| rno-miR-214-3p | up | ENSRNOT00000075700 | ENSRNOG00000048848 | Ccdc9 | coiled-coil domain containing 9 |  |
| rno-miR-370-3p | up | ENSRNOT00000031236 | ENSRNOG00000012140 | Cep89 | centrosomal protein 89 |  |
| rno-miR-665 | up | ENSRNOT00000060838 | ENSRNOG00000039668 | Col8a1 | collagen type VIII alpha 1 chain |  |
| rno-miR-370-3p | up | ENSRNOT00000026960 | ENSRNOG00000019891 | Sgta | small glutamine rich tetratricopeptide repeat containing alpha |  |
| rno-miR-665 | up | ENSRNOT00000001688 | ENSRNOG00000001252 | Chst12 | carbohydrate sulfotransferase 12 |  |
| rno-miR-673-3p | up | ENSRNOT00000005067 | ENSRNOG00000003741 | Nptx1 | neuronal pentraxin 1 |  |
| rno-miR-673-3p | up | ENSRNOT00000081604 | ENSRNOG00000000498 | Anks1a | ankyrin repeat and sterile alpha motif domain containing 1A |  |
| rno-miR-665 | up | ENSRNOT00000021405 | ENSRNOG00000015035 | Myo7b | myosin VIIb |  |
| rno-miR-370-3p | up | ENSRNOT00000070865 | ENSRNOG00000049761 | Htr6 | 5-hydroxytryptamine receptor 6 |  |
| rno-miR-132-3p | up | ENSRNOT00000014030 | ENSRNOG00000010407 | LOC103690024 | peroxisomal targeting signal 1 receptor-like |  |
| rno-miR-433-3p | up | ENSRNOT00000088766 | ENSRNOG00000061876 | Tas1r2 | taste 1 receptor member 2 |  |
| rno-miR-127-5p | up | ENSRNOT00000012551 | ENSRNOG00000009137 | Ubxn2b | UBX domain protein 2B |  |
| rno-miR-370-3p | up | ENSRNOT00000065383 | ENSRNOG00000003785 | Usp43 | ubiquitin specific peptidase 43 |  |
| rno-miR-665 | up | ENSRNOT00000037248 | ENSRNOG00000026981 | Zfp282 | zinc finger protein 282 |  |
| rno-miR-673-3p | up | ENSRNOT00000016273 | ENSRNOG00000011058 | Utrn | utrophin |  |
| rno-miR-370-3p | up | ENSRNOT00000019053 | ENSRNOG00000013641 | Myo7a | myosin VIIA |  |
| rno-miR-127-3p | up | ENSRNOT00000086244 | ENSRNOG00000001074 | Mphosph9 | M-phase phosphoprotein 9 |  |
| rno-miR-370-3p | up | ENSRNOT00000045180 | ENSRNOG00000006747 | Cc2d1a | coiled-coil and C2 domain containing 1A |  |
| rno-miR-337-5p | up | ENSRNOT00000027045 | ENSRNOG00000019924 | Thop1 | thimet oligopeptidase 1 |  |
| rno-miR-127-5p | up | ENSRNOT00000084304 | ENSRNOG00000016684 | Wnk2 | WNK lysine deficient protein kinase 2 |  |
| rno-miR-673-3p | up | ENSRNOT00000030583 | ENSRNOG00000028198 | Sh2b3 | SH2B adaptor protein 3 |  |
| rno-miR-370-3p | up | ENSRNOT00000019770 | ENSRNOG00000014626 | Katnb1 | katanin regulatory subunit B1 |  |
| rno-miR-665 | up | ENSRNOT00000049798 | ENSRNOG00000008474 | Acox3 | acyl-CoA oxidase 3, pristanoyl |  |
| rno-miR-665 | up | ENSRNOT00000021471 | ENSRNOG00000016011 | Plekhg1 | pleckstrin homology and RhoGEF domain containing G1 |  |
| rno-miR-370-3p | up | ENSRNOT00000022480 | ENSRNOG00000016687 | Ssc5d | scavenger receptor cysteine rich family member with 5 domains |  |
| rno-miR-370-3p | up | ENSRNOT00000089587 | ENSRNOG00000012140 | Cep89 | centrosomal protein 89 |  |
| rno-miR-370-3p | up | ENSRNOT00000013107 | ENSRNOG00000009790 | Kcnk3 | potassium two pore domain channel subfamily K member 3 |  |
| rno-miR-665 | up | ENSRNOT00000074558 | ENSRNOG00000046851 | Cecr6 | cat eye syndrome chromosome region, candidate 6 |  |
| rno-miR-370-3p | up | ENSRNOT00000080638 | ENSRNOG00000007645 | Kcnj9 | potassium voltage-gated channel subfamily J member 9 |  |
| rno-miR-665 | up | ENSRNOT00000011079 | ENSRNOG00000008133 | Mfng | MFNG O-fucosylpeptide 3-beta-N-acetylglucosaminyltransferase |  |
| rno-miR-370-3p | up | ENSRNOT00000078608 | ENSRNOG00000052873 | Npnt | nephronectin |  |
| rno-miR-212-5p | up | ENSRNOT00000017531 | ENSRNOG00000012337 | Pde1c | phosphodiesterase 1C |  |
| rno-miR-299a-3p | up | ENSRNOT00000003551 | ENSRNOG00000025318 | Scyl3 | SCY1 like pseudokinase 3 |  |
| rno-miR-673-3p | up | ENSRNOT00000086574 | ENSRNOG00000011058 | Utrn | utrophin |  |
| rno-miR-127-5p | up | ENSRNOT00000015921 | ENSRNOG00000011854 | RGD1563986 | similar to RIKEN cDNA E330009J07 gene |  |
| rno-miR-665 | up | ENSRNOT00000016274 | ENSRNOG00000011873 | Atg14 | autophagy related 14 |  |
| rno-miR-370-3p | up | ENSRNOT00000013573 | ENSRNOG00000010083 | Prpsap1 | phosphoribosyl pyrophosphate synthetase-associated protein 1 |  |
| rno-miR-370-3p | up | ENSRNOT00000077313 | ENSRNOG00000052687 | Megf8 | multiple EGF-like-domains 8 |  |
| rno-miR-370-3p | up | ENSRNOT00000088221 | ENSRNOG00000056162 | Mrfap1 | Morf4 family associated protein 1 |  |
| rno-miR-370-3p | up | ENSRNOT00000043971 | ENSRNOG00000029134 | Adgrl1 | adhesion G protein-coupled receptor L1 |  |
| rno-miR-370-3p | up | ENSRNOT00000046155 | ENSRNOG00000017023 | Tcf25 | transcription factor 25 |  |
| rno-miR-370-3p | up | ENSRNOT00000046019 | ENSRNOG00000032991 | Rufy4 | RUN and FYVE domain containing 4 |  |
| rno-miR-370-3p | up | ENSRNOT00000017559 | ENSRNOG00000013036 | Epha8 | Eph receptor A8 |  |
| rno-miR-370-3p | up | ENSRNOT00000065131 | ENSRNOG00000024039 | Serinc5 | serine incorporator 5 |  |
| rno-miR-433-3p | up | ENSRNOT00000003428 | ENSRNOG00000002538 | Epb41l5 | erythrocyte membrane protein band 4.1 like 5 |  |
| rno-miR-665 | up | ENSRNOT00000093708 | ENSRNOG00000028377 | Mpl | MPL proto-oncogene, thrombopoietin receptor |  |
| rno-miR-31a-5p | up | ENSRNOT00000019245 | ENSRNOG00000013718 | Herc2 | HECT and RLD domain containing E3 ubiquitin protein ligase 2 |  |
| rno-miR-370-3p | up | ENSRNOT00000033437 | ENSRNOG00000022176 | Gpr75 | G protein-coupled receptor 75 |  |
| rno-miR-127-3p | up | ENSRNOT00000016468 | ENSRNOG00000012184 | Urgcp | upregulator of cell proliferation |  |
| rno-miR-370-3p | up | ENSRNOT00000057058 | ENSRNOG00000009722 | Chd3 | chromodomain helicase DNA binding protein 3 |  |
| rno-miR-370-3p | up | ENSRNOT00000023671 | ENSRNOG00000017456 | Vstm2b | V-set and transmembrane domain containing 2B |  |
| rno-miR-665 | up | ENSRNOT00000014586 | ENSRNOG00000010849 | Adprhl2 | ADP-ribosylhydrolase like 2 |  |
| rno-miR-370-3p | up | ENSRNOT00000055362 | ENSRNOG00000036877 | Clca4l | chloride channel calcium activated 4-like |  |
| rno-miR-370-3p | up | ENSRNOT00000080155 | ENSRNOG00000009001 | Mtss1 | MTSS1, I-BAR domain containing |  |
| rno-miR-370-3p | up | ENSRNOT00000088202 | ENSRNOG00000019023 | Tars | threonyl-tRNA synthetase |  |
| rno-miR-673-3p | up | ENSRNOT00000086496 | ENSRNOG00000012820 | Add3 | adducin 3 |  |
| rno-miR-370-3p | up | ENSRNOT00000074374 | ENSRNOG00000046922 | Phactr4 | phosphatase and actin regulator 4 |  |
| rno-miR-673-3p | up | ENSRNOT00000082836 | ENSRNOG00000016763 | Fgfr4 | fibroblast growth factor receptor 4 |  |
| rno-novel-58-mature | up | ENSRNOT00000008759 | ENSRNOG00000005957 | Slc4a7 | solute carrier family 4 member 7 |  |
| rno-novel-58-mature | up | ENSRNOT00000049771 | ENSRNOG00000005957 | Slc4a7 | solute carrier family 4 member 7 [Source:RGD Symbol;Acc:621208] |  |
| rno-miR-665 | up | ENSRNOT00000020233 | ENSRNOG00000015061 | Igfals | insulin-like growth factor binding protein, acid labile subunit |  |
| rno-miR-409a-5p | up | ENSRNOT00000003239 | ENSRNOG00000002369 | Rgs8 | regulator of G-protein signaling 8 |  |
| rno-miR-665 | up | ENSRNOT00000013623 | ENSRNOG00000010275 | Slc17a9 | solute carrier family 17 member 9 |  |
| rno-miR-370-3p | up | ENSRNOT00000079479 | ENSRNOG00000052873 | Npnt | nephronectin [Source:MGI |  |
| rno-miR-665 | up | ENSRNOT00000089781 | ENSRNOG00000055433 | Trim46 | tripartite motif-containing 46 |  |
| rno-miR-370-3p | up | ENSRNOT00000079641 | ENSRNOG00000014461 | Galns | galactosamine (N-acetyl)-6-sulfatase |  |
| rno-miR-673-3p | up | ENSRNOT00000006624 | ENSRNOG00000004752 | Nalcn | sodium leak channel, non-selective |  |
| rno-miR-673-3p | up | ENSRNOT00000057404 | ENSRNOG00000004752 | Nalcn | sodium leak channel, non-selective |  |
| rno-miR-370-3p | up | ENSRNOT00000092388 | ENSRNOG00000052296 | Shank3 | SH3 and multiple ankyrin repeat domains 3 |  |
| rno-miR-665 | up | ENSRNOT00000016962 | ENSRNOG00000012685 | Adck1 | aarF domain containing kinase 1 |  |
| rno-miR-665 | up | ENSRNOT00000066199 | ENSRNOG00000043370 | Fubp1 | far upstream element binding protein 1 |  |
| rno-miR-673-3p | up | ENSRNOT00000087712 | ENSRNOG00000000007 | Gad1 | glutamate decarboxylase 1 |  |
| rno-miR-1193-3p | up | ENSRNOT00000089723 | ENSRNOG00000047756 | Mef2a | myocyte enhancer factor 2a |  |
| rno-miR-370-3p | up | ENSRNOT00000003072 | ENSRNOG00000002251 | Sec31a | SEC31 homolog A, COPII coat complex component |  |
| rno-miR-370-3p | up | ENSRNOT00000079999 | ENSRNOG00000047247 | Ptprs | protein tyrosine phosphatase, receptor type, S |  |
| rno-miR-1193-3p | up | ENSRNOT00000084946 | ENSRNOG00000047756 | Mef2a | myocyte enhancer factor 2a |  |
| rno-miR-370-3p | up | ENSRNOT00000073991 | ENSRNOG00000047247 | Ptprs | protein tyrosine phosphatase, receptor type, S |  |
| rno-miR-370-3p | up | ENSRNOT00000074469 | ENSRNOG00000047247 | Ptprs | protein tyrosine phosphatase, receptor type, S |  |
| rno-miR-673-3p | up | ENSRNOT00000089695 | ENSRNOG00000004752 | Nalcn | sodium leak channel, non-selective |  |
| rno-miR-214-3p | up | ENSRNOT00000011392 | ENSRNOG00000008572 | Ppcs | phosphopantothenoylcysteine synthetase |  |
| rno-miR-370-3p | up | ENSRNOT00000081604 | ENSRNOG00000000498 | Anks1a | ankyrin repeat and sterile alpha motif domain containing 1A |  |
| rno-miR-370-3p | up | ENSRNOT00000015210 | ENSRNOG00000011321 | Rftn1 | raftlin lipid raft linker 1 |  |
| rno-miR-132-3p | up | ENSRNOT00000085675 | ENSRNOG00000010407 | LOC103690024 | peroxisomal targeting signal 1 receptor-like |  |
| rno-miR-665 | up | ENSRNOT00000093752 | ENSRNOG00000028377 | Mpl | MPL proto-oncogene, thrombopoietin receptor |  |
| rno-miR-673-3p | up | ENSRNOT00000016191 | ENSRNOG00000011981 | Slc39a13 | solute carrier family 39 member 13 |  |
| rno-miR-370-3p | up | ENSRNOT00000020529 | ENSRNOG00000015191 | Phc1 | polyhomeotic homolog 1 |  |
| rno-miR-370-3p | up | ENSRNOT00000078028 | ENSRNOG00000049761 | Htr6 | 5-hydroxytryptamine receptor 6 |  |
| rno-miR-493-3p | up | ENSRNOT00000049512 | ENSRNOG00000000973 | RGD1563425 | similar to FLJ35784 protein |  |
| rno-miR-665 | up | ENSRNOT00000089325 | ENSRNOG00000013055 | Zfyve16 | zinc finger FYVE-type containing 16 |  |
| rno-miR-212-5p | up | ENSRNOT00000028971 | ENSRNOG00000021338 | Tmem132a | transmembrane protein 132A |  |
| rno-miR-212-5p | up | ENSRNOT00000005085 | ENSRNOG00000003745 | Atf3 | activating transcription factor 3 |  |
| rno-miR-370-3p | up | ENSRNOT00000011592 | ENSRNOG00000008578 | Cdk16 | cyclin-dependent kinase 16 |  |
| rno-miR-132-3p | up | ENSRNOT00000073341 | ENSRNOG00000049203 | Pex5 | peroxisomal biogenesis factor 5 |  |
| rno-miR-127-3p | up | ENSRNOT00000092388 | ENSRNOG00000052296 | Shank3 | SH3 and multiple ankyrin repeat domains 3 |  |
| rno-miR-665 | up | ENSRNOT00000011356 | ENSRNOG00000008332 | Smo | smoothened, frizzled class receptor |  |
| rno-miR-665 | up | ENSRNOT00000023645 | ENSRNOG00000017500 | Mtss1l | MTSS1L, I-BAR domain containing |  |
| rno-miR-370-3p | up | ENSRNOT00000021041 | ENSRNOG00000015671 | Tectb | tectorin beta |  |
| rno-miR-370-3p | up | ENSRNOT00000034102 | ENSRNOG00000025634 | Zfhx2 | zinc finger homeobox 2 |  |
| rno-miR-370-3p | up | ENSRNOT00000091367 | ENSRNOG00000002251 | Sec31a | SEC31 homolog A, COPII coat complex component |  |
| rno-miR-127-5p | up | ENSRNOT00000039954 | ENSRNOG00000010031 | Vtn | vitronectin |  |
| rno-miR-370-3p | up | ENSRNOT00000014819 | ENSRNOG00000011140 | Fam213a | family with sequence similarity 213, member A |  |
| rno-miR-370-3p | up | ENSRNOT00000047380 | ENSRNOG00000032561 | Slc52a2 | solute carrier family 52 member 2 |  |
| rno-miR-370-3p | up | ENSRNOT00000004361 | ENSRNOG00000003268 | Maml1 | mastermind-like transcriptional coactivator 1 |  |
| rno-miR-214-3p | up | ENSRNOT00000034992 | ENSRNOG00000028403 | Ptcd2 | pentatricopeptide repeat domain 2 |  |
| rno-miR-212-5p | up | ENSRNOT00000005834 | ENSRNOG00000004161 | Mtif2 | mitochondrial translational initiation factor 2 |  |
| rno-miR-341 | up | ENSRNOT00000072802 | ENSRNOG00000049768 | Adcy9 | adenylate cyclase 9 |  |
| rno-miR-31a-5p | up | ENSRNOT00000049082 | ENSRNOG00000007285 | Tp53rk | TP53 regulating kinase |  |
| rno-miR-665 | up | ENSRNOT00000000831 | ENSRNOG00000000664 | Tpst2 | tyrosylprotein sulfotransferase 2 |  |
| rno-miR-370-3p | up | ENSRNOT00000049624 | ENSRNOG00000024101 | Phkb | phosphorylase kinase regulatory subunit beta |  |
| rno-miR-370-3p | up | ENSRNOT00000093552 | ENSRNOG00000060729 | Qtrt2 | queuine tRNA-ribosyltransferase accessory subunit 2 |  |
| rno-miR-673-3p | up | ENSRNOT00000064501 | ENSRNOG00000018838 | Paox | polyamine oxidase |  |
| rno-miR-673-3p | up | ENSRNOT00000017600 | ENSRNOG00000012820 | Add3 | adducin 3 |  |
| rno-miR-673-3p | up | ENSRNOT00000044827 | ENSRNOG00000012820 | Add3 | adducin 3 |  |
| rno-miR-370-3p | up | ENSRNOT00000077869 | ENSRNOG00000060008 | Cog7 | component of oligomeric golgi complex 7 |  |
| rno-miR-370-3p | up | ENSRNOT00000080534 | ENSRNOG00000060008 | Cog7 | component of oligomeric golgi complex 7 |  |
| rno-miR-370-3p | up | ENSRNOT00000026273 | ENSRNOG00000019276 | RGD735029 | SEL1 domain containing protein RGD735029 |  |
| rno-miR-140-3p | up | ENSRNOT00000055321 | ENSRNOG00000036859 | Gdpd5 | glycerophosphodiester phosphodiesterase domain containing 5 |  |
| rno-miR-665 | up | ENSRNOT00000027026 | ENSRNOG00000027299 | Pcdhga5 | protocadherin gamma subfamily A, 5 |  |
| rno-miR-370-3p | up | ENSRNOT00000086187 | ENSRNOG00000024101 | Phkb | phosphorylase kinase regulatory subunit beta |  |
| rno-miR-673-3p | up | ENSRNOT00000044395 | ENSRNOG00000000498 | Anks1a | ankyrin repeat and sterile alpha motif domain containing 1A |  |
| rno-miR-214-3p | up | ENSRNOT00000078260 | ENSRNOG00000018586 | Fam134a | family with sequence similarity 134, member A |  |
| rno-miR-665 | up | ENSRNOT00000074998 | ENSRNOG00000009260 | Cnr2 | cannabinoid receptor 2 |  |
| rno-miR-370-3p | up | ENSRNOT00000026075 | ENSRNOG00000019211 | Olfml3 | olfactomedin-like 3 |  |
| rno-miR-370-3p | up | ENSRNOT00000009806 | ENSRNOG00000006747 | Cc2d1a | coiled-coil and C2 domain containing 1A |  |
| rno-miR-214-3p | up | ENSRNOT00000005668 | ENSRNOG00000004152 | Lrp12 | LDL receptor related protein 12 |  |
| rno-miR-433-3p | up | ENSRNOT00000055570 | ENSRNOG00000011053 | Cyp2u1 | cytochrome P450, family 2, subfamily u, polypeptide 1 |  |
| rno-miR-665 | up | ENSRNOT00000034506 | ENSRNOG00000026900 | Ppil2 | peptidylprolyl isomerase like 2 |  |
| rno-miR-665 | up | ENSRNOT00000022534 | ENSRNOG00000016665 | Tpra1 | transmembrane protein adipocyte associated 1 |  |
| rno-miR-370-3p | up | ENSRNOT00000010970 | ENSRNOG00000023440 | Phex | phosphate regulating endopeptidase homolog, X-linked |  |
| rno-miR-665 | up | ENSRNOT00000064949 | ENSRNOG00000042502 | Smim17 | small integral membrane protein 17 |  |
| rno-miR-370-3p | up | ENSRNOT00000013277 | ENSRNOG00000010005 | Adck2 | aarF domain containing kinase 2 |  |
| rno-miR-665 | up | ENSRNOT00000088624 | ENSRNOG00000017610 | Nedd4l | neural precursor cell expressed, developmentally down-regulated 4-like, E3 ubiquitin protein ligase |  |
| rno-miR-214-3p | up | ENSRNOT00000057146 | ENSRNOG00000004398 | Pkhd1l1 | polycystic kidney and hepatic disease 1-like 1 |  |
| rno-miR-665 | up | ENSRNOT00000031225 | ENSRNOG00000022838 | Cnksr1 | connector enhancer of kinase suppressor of Ras 1 |  |
| rno-miR-665 | up | ENSRNOT00000092866 | ENSRNOG00000062108 | Tomm5 | translocase of outer mitochondrial membrane 5 |  |
| rno-miR-341 | up | ENSRNOT00000089332 | ENSRNOG00000042860 | Pappa2 | pappalysin 2 |  |
| rno-miR-31a-5p | up | ENSRNOT00000090796 | ENSRNOG00000013718 | Herc2 | HECT and RLD domain containing E3 ubiquitin protein ligase 2 |  |
| rno-miR-132-3p | up | ENSRNOT00000071625 | ENSRNOG00000049203 | Pex5 | peroxisomal biogenesis factor 5 |  |
| rno-miR-370-3p | up | ENSRNOT00000091259 | ENSRNOG00000053122 | Scn1a | sodium voltage-gated channel alpha subunit 1 |  |
| rno-miR-673-3p | up | ENSRNOT00000001638 | ENSRNOG00000001223 | Pttg1ip | pituitary tumor-transforming 1 interacting protein |  |
| rno-miR-370-3p | up | ENSRNOT00000080189 | ENSRNOG00000052685 | Cd248 | CD248 molecule |  |
| rno-miR-370-3p | up | ENSRNOT00000074361 | ENSRNOG00000050278 | LOC108348067 | endosialin |  |
| rno-miR-214-3p | up | ENSRNOT00000075555 | ENSRNOG00000047396 | Rmnd5b | required for meiotic nuclear division 5 homolog B |  |
| rno-miR-665 | up | ENSRNOT00000046381 | ENSRNOG00000025155 | Lmtk2 | lemur tyrosine kinase 2 |  |
| rno-miR-665 | up | ENSRNOT00000017705 | ENSRNOG00000013055 | Zfyve16 | zinc finger FYVE-type containing 16 |  |
| rno-miR-214-3p | up | ENSRNOT00000075525 | ENSRNOG00000047719 | LOC103689927 | protein RMD5 homolog B |  |
| rno-miR-370-3p | up | ENSRNOT00000043197 | ENSRNOG00000028892 | Prr36 | proline rich 36 |  |
| rno-miR-214-3p | up | ENSRNOT00000068717 | ENSRNOG00000007552 | Arhgap36 | Rho GTPase activating protein 36 |  |
| rno-miR-370-3p | up | ENSRNOT00000025634 | ENSRNOG00000018971 | Mob3a | MOB kinase activator 3A |  |
| rno-miR-370-3p | up | ENSRNOT00000078884 | ENSRNOG00000014528 | Zswim6 | zinc finger, SWIM-type containing 6 |  |
| rno-miR-665 | up | ENSRNOT00000082470 | ENSRNOG00000054838 | Mcrs1 | microspherule protein 1 |  |
| rno-miR-140-3p | up | ENSRNOT00000091811 | ENSRNOG00000009588 | Snph | syntaphilin |  |
| rno-miR-140-3p | up | ENSRNOT00000012727 | ENSRNOG00000009588 | Snph | syntaphilin |  |
| rno-miR-665 | up | ENSRNOT00000018956 | ENSRNOG00000014163 | Zfp536 | zinc finger protein 536 |  |
| rno-miR-370-3p | up | ENSRNOT00000083595 | ENSRNOG00000032991 | Rufy4 | RUN and FYVE domain containing 4 |  |
| rno-miR-665 | up | ENSRNOT00000085479 | ENSRNOG00000002659 | Ciita | class II, major histocompatibility complex, transactivator |  |
| rno-miR-370-3p | up | ENSRNOT00000010763 | ENSRNOG00000008024 | Pxk | PX domain containing serine/threonine kinase |  |
| rno-miR-370-3p | up | ENSRNOT00000008355 | ENSRNOG00000005218 | Sf3a1 | splicing factor 3a, subunit 1 |  |
| rno-miR-370-3p | up | ENSRNOT00000084970 | ENSRNOG00000017746 | Pard6a | par-6 family cell polarity regulator alpha |  |
| rno-miR-665 | up | ENSRNOT00000027992 | ENSRNOG00000020623 | Aldh16a1 | aldehyde dehydrogenase 16 family, member A1 |  |
| rno-miR-370-3p | up | ENSRNOT00000026120 | ENSRNOG00000019299 | Edem2 | ER degradation enhancing alpha-mannosidase like protein 2 |  |
| rno-miR-214-3p | up | ENSRNOT00000005958 | ENSRNOG00000004398 | Pkhd1l1 | polycystic kidney and hepatic disease 1-like 1 |  |
| rno-miR-370-3p | up | ENSRNOT00000010471 | ENSRNOG00000007925 | Pak6 | p21 (RAC1) activated kinase 6 |  |
| rno-miR-673-3p | up | ENSRNOT00000076044 | ENSRNOG00000045913 | Prdm16 | PR/SET domain 16 |  |
| rno-miR-370-3p | up | ENSRNOT00000027339 | ENSRNOG00000020197 | LOC100911932 | endosialin-like |  |
| rno-miR-341 | up | ENSRNOT00000088288 | ENSRNOG00000058842 | Sptbn2 | spectrin, beta, non-erythrocytic 2 |  |
| rno-miR-370-3p | up | ENSRNOT00000091543 | ENSRNOG00000010381 | Mknk1 | MAP kinase-interacting serine/threonine kinase 1 |  |
| rno-miR-673-3p | up | ENSRNOT00000071280 | ENSRNOG00000045913 | Prdm16 | PR/SET domain 16 |  |
| rno-miR-673-3p | up | ENSRNOT00000072059 | ENSRNOG00000045913 | Prdm16 | PR/SET domain 16 |  |
| rno-miR-673-3p | up | ENSRNOT00000077015 | ENSRNOG00000045913 | Prdm16 | PR/SET domain 16 |  |
| rno-miR-370-3p | up | ENSRNOT00000064292 | ENSRNOG00000014528 | Zswim6 | zinc finger, SWIM-type containing 6 |  |
| rno-miR-665 | up | ENSRNOT00000023890 | ENSRNOG00000017610 | Nedd4l | neural precursor cell expressed, developmentally down-regulated 4-like, E3 ubiquitin protein ligase |  |
| rno-miR-370-3p | up | ENSRNOT00000078692 | ENSRNOG00000055531 | Saa4 | serum amyloid A4 |  |
| rno-miR-6331 | up | ENSRNOT00000083056 | ENSRNOG00000053178 | Dyrk4 | dual specificity tyrosine phosphorylation regulated kinase 4 |  |
| rno-miR-433-3p | up | ENSRNOT00000089674 | ENSRNOG00000011053 | Cyp2u1 | cytochrome P450, family 2, subfamily u, polypeptide 1 |  |
| rno-miR-493-3p | up | ENSRNOT00000000203 | ENSRNOG00000000187 | Csf2rb | colony stimulating factor 2 receptor beta common subunit |  |
| rno-miR-214-3p | up | ENSRNOT00000085911 | ENSRNOG00000001432 | Dtx2 | deltex E3 ubiquitin ligase 2 |  |
| rno-miR-370-3p | up | ENSRNOT00000037115 | ENSRNOG00000024213 | Golim4 | golgi integral membrane protein 4 |  |
| rno-miR-370-3p | up | ENSRNOT00000066032 | ENSRNOG00000014087 | Kifc3 | kinesin family member C3 |  |
| rno-miR-370-3p | up | ENSRNOT00000067866 | ENSRNOG00000003590 | Tom1l2 | target of myb1 like 2 membrane trafficking protein |  |
| rno-miR-370-3p | up | ENSRNOT00000025743 | ENSRNOG00000019043 | Pdcd1 | programmed cell death 1 |  |
| rno-miR-665 | up | ENSRNOT00000034762 | ENSRNOG00000028377 | Mpl | MPL proto-oncogene, thrombopoietin receptor |  |
| rno-novel-125-mature | up | ENSRNOT00000087619 | ENSRNOG00000052925 | NEWGENE_621351 | collagen, type I, alpha 2 |  |
| rno-miR-665 | up | ENSRNOT00000067665 | ENSRNOG00000009473 | Osbpl7 | oxysterol binding protein-like 7 |  |
| rno-miR-370-3p | up | ENSRNOT00000089611 | ENSRNOG00000000302 | Sesn1 | sestrin 1 |  |
| rno-miR-370-3p | up | ENSRNOT00000044395 | ENSRNOG00000000498 | Anks1a | ankyrin repeat and sterile alpha motif domain containing 1A |  |
| rno-miR-665 | up | ENSRNOT00000093502 | ENSRNOG00000028377 | Mpl | MPL proto-oncogene, thrombopoietin receptor |  |
| rno-miR-140-3p | up | ENSRNOT00000019465 | ENSRNOG00000014079 | Stat1 | signal transducer and activator of transcription 1 |  |
| rno-miR-370-3p | up | ENSRNOT00000011864 | ENSRNOG00000008652 | Phip | pleckstrin homology domain interacting protein |  |
| rno-miR-665 | up | ENSRNOT00000065494 | ENSRNOG00000016366 | Colec12 | collectin sub-family member 12 |  |
| rno-miR-665 | up | ENSRNOT00000003684 | ENSRNOG00000002653 | Kcnk2 | potassium two pore domain channel subfamily K member 2 |  |
| rno-miR-665 | up | ENSRNOT00000021926 | ENSRNOG00000016308 | Il10ra | interleukin 10 receptor subunit alpha |  |
| rno-miR-433-3p | up | ENSRNOT00000084155 | ENSRNOG00000004377 | Lpin1 | lipin 1 |  |
| rno-miR-214-3p | up | ENSRNOT00000064792 | ENSRNOG00000013442 | Ciz1 | CDKN1A interacting zinc finger protein 1 |  |
| rno-miR-665 | up | ENSRNOT00000081206 | ENSRNOG00000054274 | Cacnb3 | calcium voltage-gated channel auxiliary subunit beta 3 |  |
| rno-miR-370-3p | up | ENSRNOT00000068586 | ENSRNOG00000028892 | Prr36 | proline rich 36 |  |
| rno-miR-370-3p | up | ENSRNOT00000009778 | ENSRNOG00000007433 | Cyb561 | cytochrome b-561 |  |
| rno-miR-665 | up | ENSRNOT00000093301 | ENSRNOG00000028377 | Mpl | MPL proto-oncogene, thrombopoietin receptor |  |
| rno-miR-665 | up | ENSRNOT00000085310 | ENSRNOG00000002659 | Ciita | class II, major histocompatibility complex, transactivator |  |
| rno-miR-370-3p | up | ENSRNOT00000009803 | ENSRNOG00000025120 | Gli1 | GLI family zinc finger 1 |  |
| rno-miR-212-5p | up | ENSRNOT00000083169 | ENSRNOG00000061176 | Nudt3 | nudix hydrolase 3 |  |
| rno-miR-127-5p | up | ENSRNOT00000048845 | ENSRNOG00000000536 | Mdga1 | MAM domain containing glycosylphosphatidylinositol anchor 1 |  |
| rno-miR-665 | up | ENSRNOT00000067272 | ENSRNOG00000043378 | Nkpd1 | NTPase, KAP family P-loop domain containing 1 |  |
| rno-miR-6331 | up | ENSRNOT00000047817 | ENSRNOG00000001627 | Abi3bp | ABI family member 3 binding protein |  |
| rno-miR-370-3p | up | ENSRNOT00000012480 | ENSRNOG00000009360 | Sh3bp1 | SH3-domain binding protein 1 |  |
| rno-miR-341 | up | ENSRNOT00000014444 | ENSRNOG00000010718 | Gpr153 | G protein-coupled receptor 153 |  |
| rno-miR-370-3p | up | ENSRNOT00000025531 | ENSRNOG00000018691 | Ccnl2 | cyclin L2 |  |
| rno-miR-370-3p | up | ENSRNOT00000038016 | ENSRNOG00000027456 | Cdc42bpg | CDC42 binding protein kinase gamma |  |
| rno-miR-673-3p | up | ENSRNOT00000025576 | ENSRNOG00000018822 | Slc5a5 | solute carrier family 5 member 5 |  |
| rno-miR-433-3p | up | ENSRNOT00000020838 | ENSRNOG00000015173 | Mbtps1 | membrane-bound transcription factor peptidase, site 1 |  |
| rno-miR-140-5p | up | ENSRNOT00000024310 | ENSRNOG00000017777 | Ahcy | adenosylhomocysteinase |  |
| rno-miR-370-3p | up | ENSRNOT00000008034 | ENSRNOG00000005900 | Slc2a6 | solute carrier family 2 member 6 |  |
| rno-miR-1193-3p | up | ENSRNOT00000070864 | ENSRNOG00000047756 | Mef2a | myocyte enhancer factor 2a |  |
| rno-miR-370-3p | up | ENSRNOT00000045699 | ENSRNOG00000029134 | Adgrl1 | adhesion G protein-coupled receptor L1 |  |
| rno-miR-485-5p | up | ENSRNOT00000078403 | ENSRNOG00000052894 | Epg5 | ectopic P-granules autophagy protein 5 homolog |  |
| rno-miR-673-3p | up | ENSRNOT00000064308 | ENSRNOG00000048036 | Ncan | neurocan |  |
| rno-novel-125-mature | up | ENSRNOT00000038454 | ENSRNOG00000022964 | Rnf222 | ring finger protein 222 |  |
| rno-miR-665 | up | ENSRNOT00000028709 | ENSRNOG00000021143 | Setdb1 | SET domain, bifurcated 1 |  |
| rno-miR-132-5p | up | ENSRNOT00000021170 | ENSRNOG00000015602 | Cdh2 | cadherin 2 |  |
| rno-miR-433-3p | up | ENSRNOT00000071306 | ENSRNOG00000004564 | Lrriq1 | leucine-rich repeats and IQ motif containing 1 |  |
| rno-miR-665 | up | ENSRNOT00000032240 | ENSRNOG00000023210 | Trappc10 | trafficking protein particle complex 10 |  |
| rno-miR-214-3p | up | ENSRNOT00000093538 | ENSRNOG00000007552 | Arhgap36 | Rho GTPase activating protein 36 |  |
| rno-miR-6331 | up | ENSRNOT00000088116 | ENSRNOG00000054669 | RGD1305110 | similar to KIAA1841 protein |  |
| rno-miR-665 | up | ENSRNOT00000009883 | ENSRNOG00000007270 | Il12rb2 | interleukin 12 receptor subunit beta 2 |  |
| rno-miR-6331 | up | ENSRNOT00000091143 | ENSRNOG00000054669 | RGD1305110 | similar to KIAA1841 protein |  |
| rno-miR-299a-3p | up | ENSRNOT00000034282 | ENSRNOG00000015002 | Abhd15 | abhydrolase domain containing 15 |  |
| rno-miR-1193-3p | up | ENSRNOT00000072915 | ENSRNOG00000047756 | Mef2a | myocyte enhancer factor 2a |  |
| rno-miR-370-3p | up | ENSRNOT00000012219 | ENSRNOG00000009198 | Rab6b | RAB6B, member RAS oncogene family |  |
| rno-miR-665 | up | ENSRNOT00000016221 | ENSRNOG00000011375 | Tsc2 | tuberous sclerosis 2 |  |
| rno-miR-665 | up | ENSRNOT00000026739 | ENSRNOG00000019735 | Dph2 | DPH2 homolog |  |
| rno-miR-673-3p | up | ENSRNOT00000073642 | ENSRNOG00000047028 | Mmp24 | matrix metallopeptidase 24 |  |
| rno-miR-370-3p | up | ENSRNOT00000081567 | ENSRNOG00000020669 | Ovol1 | ovo like transcriptional repressor 1 |  |
| rno-miR-665 | up | ENSRNOT00000022742 | ENSRNOG00000016483 | Myo16 | myosin XVI |  |
| rno-miR-370-3p | up | ENSRNOT00000089229 | ENSRNOG00000024213 | Golim4 | golgi integral membrane protein 4 |  |
| rno-miR-140-3p | up | ENSRNOT00000041371 | ENSRNOG00000013773 | Secisbp2 | SECIS binding protein 2 |  |
| rno-miR-370-3p | up | ENSRNOT00000070822 | ENSRNOG00000047246 | Chtf8 | chromosome transmission fidelity factor 8 |  |
| rno-miR-673-3p | up | ENSRNOT00000040270 | ENSRNOG00000010326 | Col20a1 | collagen type XX alpha 1 chain |  |
| rno-novel-125-mature | up | ENSRNOT00000089564 | ENSRNOG00000052925 | NEWGENE_621351 | collagen, type I, alpha 2 |  |
| rno-novel-125-mature | up | ENSRNOT00000089292 | ENSRNOG00000011292 | NEWGENE_621351 | collagen, type I, alpha 2 |  |
| rno-novel-125-mature | up | ENSRNOT00000016423 | ENSRNOG00000011292 | NEWGENE_621351 | collagen, type I, alpha 2 |  |
| rno-miR-665 | up | ENSRNOT00000007447 | ENSRNOG00000005357 | Ctc1 | CST telomere replication complex component 1 |  |
| rno-miR-665 | up | ENSRNOT00000052138 | ENSRNOG00000011375 | Tsc2 | tuberous sclerosis 2 |  |
| rno-miR-214-3p | up | ENSRNOT00000001950 | ENSRNOG00000001432 | Dtx2 | deltex E3 ubiquitin ligase 2 |  |
| rno-miR-370-3p | up | ENSRNOT00000006773 | ENSRNOG00000004925 | Ppp1r12a | protein phosphatase 1, regulatory subunit 12A |  |
| rno-miR-370-3p | up | ENSRNOT00000041473 | ENSRNOG00000004925 | Ppp1r12a | protein phosphatase 1, regulatory subunit 12A |  |
| rno-miR-370-3p | up | ENSRNOT00000087100 | ENSRNOG00000014626 | Katnb1 | katanin regulatory subunit B1 |  |
| rno-miR-665 | up | ENSRNOT00000085830 | ENSRNOG00000009473 | Osbpl7 | oxysterol binding protein-like 7 |  |
| rno-miR-665 | up | ENSRNOT00000059255 | ENSRNOG00000003147 | Sqstm1 | sequestosome 1 |  |
| rno-miR-370-3p | up | ENSRNOT00000001668 | ENSRNOG00000001243 | Zcchc8 | zinc finger CCHC-type containing 8 |  |
| rno-miR-370-3p | up | ENSRNOT00000070819 | ENSRNOG00000018395 | Usp40 | ubiquitin specific peptidase 40 |  |
| rno-miR-673-3p | up | ENSRNOT00000004104 | ENSRNOG00000003073 | Gpr161 | G protein-coupled receptor 161 |  |
| rno-miR-341 | up | ENSRNOT00000039210 | ENSRNOG00000042860 | Pappa2 | pappalysin 2 |  |
| rno-miR-665 | up | ENSRNOT00000001968 | ENSRNOG00000001449 | Pom121 | POM121 transmembrane nucleoporin |  |
| rno-novel-58-mature | up | ENSRNOT00000024891 | ENSRNOG00000018000 | Ranbp10 | RAN binding protein 10 |  |
| rno-miR-370-3p | up | ENSRNOT00000024714 | ENSRNOG00000018288 | Ncoa6 | nuclear receptor coactivator 6 |  |
| rno-miR-370-3p | up | ENSRNOT00000060020 | ENSRNOG00000039214 | RGD1305455 | similar to hypothetical protein FLJ10925 |  |
| rno-miR-665 | up | ENSRNOT00000040393 | ENSRNOG00000023485 | Ushbp1 | USH1 protein network component harmonin binding protein 1 |  |
| rno-miR-665 | up | ENSRNOT00000085527 | ENSRNOG00000019484 | Slc6a9 | solute carrier family 6 member 9 |  |
| rno-miR-673-3p | up | ENSRNOT00000090100 | ENSRNOG00000060707 | Atg2a | autophagy related 2A |  |
| rno-miR-122-5p | up | ENSRNOT00000037371 | ENSRNOG00000024780 | MGC95210 | hypothetical LOC287798 |  |
| rno-miR-665 | up | ENSRNOT00000078793 | ENSRNOG00000010284 | St3gal5 | ST3 beta-galactoside alpha-2,3-sialyltransferase 5 |  |
| rno-miR-214-3p | up | ENSRNOT00000057330 | ENSRNOG00000018586 | Fam134a | family with sequence similarity 134, member A |  |
| rno-miR-665 | up | ENSRNOT00000009999 | ENSRNOG00000007515 | Kif3a | kinesin family member 3a |  |
| rno-miR-370-3p | up | ENSRNOT00000033285 | ENSRNOG00000024259 | Tmem54 | transmembrane protein 54 |  |
| rno-miR-370-3p | up | ENSRNOT00000084313 | ENSRNOG00000060750 | Rnf111 | ring finger protein 111 |  |
| rno-miR-370-3p | up | ENSRNOT00000017407 | ENSRNOG00000012357 | Unc45a | unc-45 myosin chaperone A |  |
| rno-miR-665 | up | ENSRNOT00000012600 | ENSRNOG00000009431 | Tbc1d4 | TBC1 domain family, member 4 |  |
| rno-miR-665 | up | ENSRNOT00000064942 | ENSRNOG00000009431 | Tbc1d4 | TBC1 domain family, member 4 |  |
| rno-miR-370-3p | up | ENSRNOT00000017063 | ENSRNOG00000012762 | Zfp64 | zinc finger protein 64 |  |
| rno-miR-370-3p | up | ENSRNOT00000024779 | ENSRNOG00000018255 | Clptm1 | cleft lip and palate associated transmembrane protein 1 |  |
| rno-miR-370-3p | up | ENSRNOT00000057739 | ENSRNOG00000017714 | Usp3 | ubiquitin specific peptidase 3 |  |
| rno-miR-6331 | up | ENSRNOT00000085886 | ENSRNOG00000054669 | RGD1305110 | similar to KIAA1841 protein |  |
| rno-miR-370-3p | up | ENSRNOT00000075967 | ENSRNOG00000023577 | Gigyf2 | GRB10 interacting GYF protein 2 |  |
| rno-miR-214-3p | up | ENSRNOT00000027241 | ENSRNOG00000020088 | Klhl26 | kelch-like family member 26 |  |
| rno-miR-370-3p | up | ENSRNOT00000006376 | ENSRNOG00000004361 | Parp10 | poly (ADP-ribose) polymerase family, member 10 |  |
| rno-miR-214-3p | up | ENSRNOT00000010968 | ENSRNOG00000008144 | Irf1 | interferon regulatory factor 1 |  |
| rno-miR-370-3p | up | ENSRNOT00000017264 | ENSRNOG00000012866 | Nek8 | NIMA-related kinase 8 |  |
| rno-miR-665 | up | ENSRNOT00000015827 | ENSRNOG00000011908 | Asxl2 | additional sex combs like 2, transcriptional regulator |  |
| rno-miR-370-3p | up | ENSRNOT00000004196 | ENSRNOG00000043381 | Cyth1 | cytohesin 1 |  |
| rno-miR-485-5p | up | ENSRNOT00000024402 | ENSRNOG00000018020 | Apbb1 | amyloid beta precursor protein binding family B member 1 |  |
| rno-miR-145-3p | up | ENSRNOT00000019189 | ENSRNOG00000014000 | Kif2a | kinesin family member 2A |  |
| rno-miR-370-3p | up | ENSRNOT00000010968 | ENSRNOG00000008144 | Irf1 | interferon regulatory factor 1 |  |
| rno-miR-665 | up | ENSRNOT00000064041 | ENSRNOG00000012420 | Bcl9l | B-cell CLL/lymphoma 9-like |  |
| rno-miR-370-3p | up | ENSRNOT00000064309 | ENSRNOG00000008578 | Cdk16 | cyclin-dependent kinase 16 |  |
| rno-miR-6331 | up | ENSRNOT00000025266 | ENSRNOG00000018529 | Csnk1g2 | casein kinase 1, gamma 2 |  |
| rno-miR-370-3p | up | ENSRNOT00000017317 | ENSRNOG00000012789 | Mlnr | motilin receptor |  |
| rno-miR-370-3p | up | ENSRNOT00000009900 | ENSRNOG00000007324 | Plxna2 | plexin A2 |  |
| rno-miR-370-3p | up | ENSRNOT00000022976 | ENSRNOG00000016905 | Hmg20a | high mobility group 20A |  |
| rno-miR-370-3p | up | ENSRNOT00000031252 | ENSRNOG00000022664 | Kat7 | lysine acetyltransferase 7 |  |
| rno-miR-6331 | up | ENSRNOT00000015802 | ENSRNOG00000011752 | Sh3d19 | SH3 domain containing 19 |  |
| rno-miR-370-3p | up | ENSRNOT00000072033 | ENSRNOG00000047628 | Khsrp | KH-type splicing regulatory protein |  |
| rno-miR-673-3p | up | ENSRNOT00000015845 | ENSRNOG00000011526 | Pcsk6 | proprotein convertase subtilisin/kexin type 6 |  |
| rno-miR-673-3p | up | ENSRNOT00000014672 | ENSRNOG00000010870 | Foxn1 | forkhead box N1 |  |
| rno-miR-214-3p | up | ENSRNOT00000026201 | ENSRNOG00000019236 | Rnf220 | ring finger protein 220 |  |
| rno-miR-665 | up | ENSRNOT00000023078 | ENSRNOG00000016907 | Ppp5c | protein phosphatase 5, catalytic subunit |  |
| rno-miR-341 | up | ENSRNOT00000068050 | ENSRNOG00000046594 | Exoc7 | exocyst complex component 7 |  |
| rno-miR-493-3p | up | ENSRNOT00000084577 | ENSRNOG00000000999 | Smurf1 | SMAD specific E3 ubiquitin protein ligase 1 |  |
| rno-miR-665 | up | ENSRNOT00000041106 | ENSRNOG00000029028 | Mknk2 | MAP kinase-interacting serine/threonine kinase 2 |  |
| rno-miR-370-3p | up | ENSRNOT00000017937 | ENSRNOG00000013140 | Pdzd2 | PDZ domain containing 2 |  |
| rno-miR-665 | up | ENSRNOT00000048770 | ENSRNOG00000019484 | Slc6a9 | solute carrier family 6 member 9 |  |
| rno-miR-665 | up | ENSRNOT00000042786 | ENSRNOG00000029855 | Letmd1 | LETM1 domain containing 1 |  |
| rno-miR-493-3p | up | ENSRNOT00000082989 | ENSRNOG00000000999 | Smurf1 | SMAD specific E3 ubiquitin protein ligase 1 |  |
| rno-miR-665 | up | ENSRNOT00000077569 | ENSRNOG00000007515 | Kif3a | kinesin family member 3a |  |
| rno-miR-493-3p | up | ENSRNOT00000031901 | ENSRNOG00000000999 | Smurf1 | SMAD specific E3 ubiquitin protein ligase 1 |  |
| rno-miR-665 | up | ENSRNOT00000005665 | ENSRNOG00000004147 | Abca8a | ATP-binding cassette, subfamily A (ABC1), member 8a |  |
| rno-miR-665 | up | ENSRNOT00000003572 | ENSRNOG00000002659 | Ciita | class II, major histocompatibility complex, transactivator |  |
| rno-miR-665 | up | ENSRNOT00000077325 | ENSRNOG00000021143 | Setdb1 | SET domain, bifurcated 1 |  |
| rno-miR-485-5p | up | ENSRNOT00000060475 | ENSRNOG00000039463 | Pcdhga1 | protocadherin gamma subfamily A, 1 |  |
| rno-miR-485-5p | up | ENSRNOT00000026969 | ENSRNOG00000027232 | Pcdhga11 | protocadherin gamma subfamily A, 11 |  |
| rno-miR-485-5p | up | ENSRNOT00000026947 | ENSRNOG00000019799 | Pcdhgc3 | protocadherin gamma subfamily C, 3 |  |
| rno-miR-370-3p | up | ENSRNOT00000082709 | ENSRNOG00000060750 | Rnf111 | ring finger protein 111 |  |
| rno-miR-665 | up | ENSRNOT00000005438 | ENSRNOG00000004048 | Lrrk2 | leucine-rich repeat kinase 2 |  |
| rno-miR-370-3p | up | ENSRNOT00000037067 | ENSRNOG00000024557 | Cep112 | centrosomal protein 112 |  |
| rno-miR-370-3p | up | ENSRNOT00000012658 | ENSRNOG00000009465 | Sfrp2 | secreted frizzled-related protein 2 |  |
| rno-miR-370-3p | up | ENSRNOT00000043925 | ENSRNOG00000019674 | Mfsd13a | major facilitator superfamily domain containing 13A |  |
| rno-miR-370-3p | up | ENSRNOT00000086830 | ENSRNOG00000061348 | Fam53b | family with sequence similarity 53, member B |  |
| rno-miR-370-3p | up | ENSRNOT00000064588 | ENSRNOG00000024213 | Golim4 | golgi integral membrane protein 4 |  |
| rno-miR-665 | up | ENSRNOT00000039836 | ENSRNOG00000028103 | Psmd3 | proteasome 26S subunit, non-ATPase 3 |  |
| rno-miR-6331 | up | ENSRNOT00000080846 | ENSRNOG00000000614 | Bicc1 | BicC family RNA binding protein 1 |  |
| rno-miR-370-3p | up | ENSRNOT00000035770 | ENSRNOG00000012806 | Rbbp6 | RB binding protein 6, ubiquitin ligase |  |
| rno-miR-665 | up | ENSRNOT00000006582 | ENSRNOG00000032391 | Esyt2 | extended synaptotagmin 2 |  |
| rno-miR-341 | up | ENSRNOT00000013281 | ENSRNOG00000009617 | LOC100911734 | exocyst complex component 7-like |  |
| rno-novel-125-mature | up | ENSRNOT00000024966 | ENSRNOG00000018502 | Mon1a | MON1 homolog A, secretory trafficking associated |  |
| rno-miR-433-3p | up | ENSRNOT00000005863 | ENSRNOG00000004377 | Lpin1 | lipin 1 |  |
| rno-miR-665 | up | ENSRNOT00000020669 | ENSRNOG00000015365 | Col4a3 | collagen type IV alpha 3 chain |  |
| rno-miR-31a-5p | up | ENSRNOT00000004517 | ENSRNOG00000003395 | Cby3 | chibby family member 3 |  |
| rno-miR-673-3p | up | ENSRNOT00000065867 | ENSRNOG00000007681 | Brd3 | bromodomain containing 3 |  |
| rno-miR-370-3p | up | ENSRNOT00000077653 | ENSRNOG00000047628 | Khsrp | KH-type splicing regulatory protein |  |
| rno-miR-370-3p | up | ENSRNOT00000009582 | ENSRNOG00000007189 | Ttc22 | tetratricopeptide repeat domain 22 |  |
| rno-miR-341 | up | ENSRNOT00000019652 | ENSRNOG00000014254 | Cpt1a | carnitine palmitoyltransferase 1A |  |
| rno-miR-493-3p | up | ENSRNOT00000023696 | ENSRNOG00000017564 | Mib2 | mindbomb E3 ubiquitin protein ligase 2 |  |
| rno-miR-665 | up | ENSRNOT00000015055 | ENSRNOG00000011316 | Fam167a | family with sequence similarity 167, member A |  |
| rno-miR-665 | up | ENSRNOT00000027773 | ENSRNOG00000020433 | Actn4 | actinin alpha 4 |  |
| rno-miR-665 | up | ENSRNOT00000089305 | ENSRNOG00000020433 | Actn4 | actinin alpha 4 |  |
| rno-miR-665 | up | ENSRNOT00000082677 | ENSRNOG00000053410 | Adcy10 | adenylate cyclase 10 (soluble) |  |
| rno-miR-6331 | up | ENSRNOT00000000754 | ENSRNOG00000000614 | Bicc1 | BicC family RNA binding protein 1 |  |
| rno-miR-665 | up | ENSRNOT00000052360 | ENSRNOG00000031208 | Mgat1 | mannosyl (alpha-1,3-)-glycoprotein beta-1,2-N-acetylglucosaminyltransferase |  |
| rno-miR-370-3p | up | ENSRNOT00000023309 | ENSRNOG00000017321 | Trub1 | TruB pseudouridine synthase family member 1 |  |
| rno-miR-6331 | up | ENSRNOT00000080342 | ENSRNOG00000018529 | Csnk1g2 | casein kinase 1, gamma 2 |  |
| rno-miR-370-3p | up | ENSRNOT00000048379 | ENSRNOG00000018533 | Iffo1 | intermediate filament family orphan 1 |  |
| rno-miR-370-3p | up | ENSRNOT00000015705 | ENSRNOG00000011803 | Lrfn2 | leucine rich repeat and fibronectin type III domain containing 2 |  |
| rno-miR-665 | up | ENSRNOT00000090402 | ENSRNOG00000020433 | Actn4 | actinin alpha 4 |  |
| rno-miR-370-3p | up | ENSRNOT00000037147 | ENSRNOG00000028521 | Plekhm1 | pleckstrin homology and RUN domain containing M1 |  |
| rno-miR-370-3p | up | ENSRNOT00000011573 | ENSRNOG00000008431 | Gabbr2 | gamma-aminobutyric acid type B receptor subunit 2 |  |
| rno-miR-673-3p | up | ENSRNOT00000028262 | ENSRNOG00000020813 | Ltbp3 | latent transforming growth factor beta binding protein 3 |  |
| rno-miR-370-3p | up | ENSRNOT00000080285 | ENSRNOG00000004925 | Ppp1r12a | protein phosphatase 1, regulatory subunit 12A |  |
| rno-miR-370-3p | up | ENSRNOT00000007295 | ENSRNOG00000005388 | Rassf3 | Ras association domain family member 3 |  |
| rno-miR-370-3p | up | ENSRNOT00000010932 | ENSRNOG00000008246 | Emilin1 | elastin microfibril interfacer 1 |  |
| rno-miR-665 | up | ENSRNOT00000085832 | ENSRNOG00000031208 | Mgat1 | mannosyl (alpha-1,3-)-glycoprotein beta-1,2-N-acetylglucosaminyltransferase |  |
| rno-miR-665 | up | ENSRNOT00000065947 | ENSRNOG00000004147 | Abca8a | ATP-binding cassette, subfamily A (ABC1), member 8a |  |
| rno-miR-370-3p | up | ENSRNOT00000001625 | ENSRNOG00000001214 | Pfkl | phosphofructokinase, liver type |  |
| rno-miR-665 | up | ENSRNOT00000026531 | ENSRNOG00000019550 | Slc11a2 | solute carrier family 11 member 2 |  |
| rno-miR-370-3p | up | ENSRNOT00000009440 | ENSRNOG00000006965 | Aff4 | AF4/FMR2 family, member 4 |  |
| rno-miR-370-3p | up | ENSRNOT00000021582 | ENSRNOG00000015916 | Ttc38 | tetratricopeptide repeat domain 38 |  |
| rno-miR-370-3p | up | ENSRNOT00000038549 | ENSRNOG00000010392 | Nrg1 | neuregulin 1 |  |
| rno-miR-370-3p | up | ENSRNOT00000092641 | ENSRNOG00000000511 | Srpk1 | SRSF protein kinase 1 |  |
| rno-miR-214-3p | up | ENSRNOT00000026700 | ENSRNOG00000049913 | Rbm12 | RNA binding motif protein 12 |  |
| rno-miR-370-3p | up | ENSRNOT00000000597 | ENSRNOG00000000502 | Def6 | DEF6 guanine nucleotide exchange factor |  |
| rno-miR-370-3p | up | ENSRNOT00000080319 | ENSRNOG00000017714 | Usp3 | ubiquitin specific peptidase 3 |  |
| rno-novel-58-mature | up | ENSRNOT00000042576 | ENSRNOG00000009514 | Mme | membrane metallo-endopeptidase |  |
| rno-miR-370-3p | up | ENSRNOT00000000651 | ENSRNOG00000000542 | Dnah8 | dynein, axonemal, heavy chain 8 |  |
| rno-miR-665 | up | ENSRNOT00000026730 | ENSRNOG00000019651 | Slc12a4 | solute carrier family 12 member 4 |  |
| rno-miR-370-3p | up | ENSRNOT00000045964 | ENSRNOG00000007801 | R3hdm2 | R3H domain containing 2 |  |
| rno-miR-370-3p | up | ENSRNOT00000011832 | ENSRNOG00000008942 | RGD1304963 | similar to hypothetical protein MGC38716 |  |
| rno-miR-673-3p | up | ENSRNOT00000084491 | ENSRNOG00000007681 | Brd3 | bromodomain containing 3 |  |
| rno-miR-665 | up | ENSRNOT00000019521 | ENSRNOG00000014551 | Ccnj | cyclin J |  |
| rno-miR-665 | up | ENSRNOT00000068666 | ENSRNOG00000019196 | Xpnpep3 | X-prolyl aminopeptidase 3 |  |
| rno-miR-370-3p | up | ENSRNOT00000084082 | ENSRNOG00000013795 | Asb7 | ankyrin repeat and SOCS box-containing 7 |  |
| rno-miR-485-5p | up | ENSRNOT00000040969 | ENSRNOG00000010795 | Cnot4 | CCR4-NOT transcription complex, subunit 4 |  |
| rno-miR-665 | up | ENSRNOT00000038652 | ENSRNOG00000024952 | Atp8b1 | ATPase phospholipid transporting 8B1 |  |
| rno-miR-127-3p | up | ENSRNOT00000023451 | ENSRNOG00000017448 | Exoc3l2 | exocyst complex component 3-like 2 |  |
| rno-miR-370-3p | up | ENSRNOT00000045053 | ENSRNOG00000018118 | Atad3a | ATPase family, AAA domain containing 3A |  |
| rno-miR-665 | up | ENSRNOT00000025009 | ENSRNOG00000018242 | Camkk1 | calcium/calmodulin-dependent protein kinase kinase 1 |  |
| rno-miR-665 | up | ENSRNOT00000015296 | ENSRNOG00000011387 | Tet3 | tet methylcytosine dioxygenase 3 |  |
| rno-miR-140-3p | up | ENSRNOT00000027094 | ENSRNOG00000020000 | Tmem219 | transmembrane protein 219 |  |
| rno-miR-370-3p | up | ENSRNOT00000020168 | ENSRNOG00000014592 | Zbtb46 | zinc finger and BTB domain containing 46 |  |
| rno-miR-665 | up | ENSRNOT00000089853 | ENSRNOG00000033508 | Mink1 | misshapen-like kinase 1 |  |
| rno-miR-370-3p | up | ENSRNOT00000064320 | ENSRNOG00000001854 | Tmtc1 | transmembrane and tetratricopeptide repeat containing 1 |  |
| rno-miR-370-3p | up | ENSRNOT00000085853 | ENSRNOG00000039214 | RGD1305455 | similar to hypothetical protein FLJ10925 |  |
| rno-miR-370-3p | up | ENSRNOT00000071233 | ENSRNOG00000050650 | Ttc16 | tetratricopeptide repeat domain 16 |  |
| rno-miR-485-5p | up | ENSRNOT00000090700 | ENSRNOG00000012427 | Veph1 | ventricular zone expressed PH domain-containing 1 |  |
| rno-miR-370-3p | up | ENSRNOT00000027897 | ENSRNOG00000020525 | Col5a3 | collagen type V alpha 3 chain |  |
| rno-miR-665 | up | ENSRNOT00000080797 | ENSRNOG00000009381 | Mapk6 | mitogen-activated protein kinase 6 |  |
| rno-miR-665 | up | ENSRNOT00000041600 | ENSRNOG00000031448 | Stpg1 | sperm-tail PG-rich repeat containing 1 |  |
| rno-miR-665 | up | ENSRNOT00000027847 | ENSRNOG00000020501 | Tjp3 | tight junction protein 3 |  |
| rno-miR-370-3p | up | ENSRNOT00000084385 | ENSRNOG00000004566 | Arhgef15 | Rho guanine nucleotide exchange factor 15 |  |
| rno-miR-370-3p | up | ENSRNOT00000014695 | ENSRNOG00000036980 | Zgrf1 | zinc finger, GRF-type containing 1 |  |
| rno-miR-433-3p | up | ENSRNOT00000033980 | ENSRNOG00000026466 | Lrrtm3 | leucine rich repeat transmembrane neuronal 3 |  |
| rno-miR-665 | up | ENSRNOT00000077610 | ENSRNOG00000031448 | Stpg1 | sperm-tail PG-rich repeat containing 1 |  |
| rno-miR-433-3p | up | ENSRNOT00000087886 | ENSRNOG00000015173 | Mbtps1 | membrane-bound transcription factor peptidase, site 1 |  |
| rno-miR-370-3p | up | ENSRNOT00000003277 | ENSRNOG00000002301 | Uso1 | USO1 vesicle transport factor |  |
| rno-miR-665 | up | ENSRNOT00000072519 | ENSRNOG00000047924 | Zbtb7c | zinc finger and BTB domain containing 7C |  |
| rno-miR-665 | up | ENSRNOT00000045964 | ENSRNOG00000007801 | R3hdm2 | R3H domain containing 2 |  |
| rno-miR-493-3p | up | ENSRNOT00000025284 | ENSRNOG00000018304 | Lars | leucyl-tRNA synthetase |  |
| rno-miR-370-3p | up | ENSRNOT00000001242 | ENSRNOG00000000936 | Stx2 | syntaxin 2 |  |
| rno-miR-214-3p | up | ENSRNOT00000085410 | ENSRNOG00000058545 | Arhgap4 | Rho GTPase activating protein 4 |  |
| rno-miR-370-3p | up | ENSRNOT00000090001 | ENSRNOG00000007801 | R3hdm2 | R3H domain containing 2 |  |
| rno-miR-127-3p | up | ENSRNOT00000055877 | ENSRNOG00000037080 | Adamts17 | ADAM metallopeptidase with thrombospondin type 1 motif, 17 |  |
| rno-miR-665 | up | ENSRNOT00000056706 | ENSRNOG00000037462 | Eml6 | echinoderm microtubule associated protein like 6 |  |
| rno-miR-370-3p | up | ENSRNOT00000014147 | ENSRNOG00000010392 | Nrg1 | neuregulin 1 |  |
| rno-miR-214-3p | up | ENSRNOT00000026960 | ENSRNOG00000019891 | Sgta | small glutamine rich tetratricopeptide repeat containing alpha |  |
| rno-miR-665 | up | ENSRNOT00000050249 | ENSRNOG00000010284 | St3gal5 | ST3 beta-galactoside alpha-2,3-sialyltransferase 5 |  |
| rno-miR-140-3p | up | ENSRNOT00000038408 | ENSRNOG00000025075 | Relt | RELT tumor necrosis factor receptor |  |
| rno-miR-665 | up | ENSRNOT00000012407 | ENSRNOG00000009046 | Phf13 | PHD finger protein 13 |  |
| rno-miR-214-3p | up | ENSRNOT00000086849 | ENSRNOG00000060538 | Cdc42se1 | CDC42 small effector 1 |  |
| rno-miR-665 | up | ENSRNOT00000001961 | ENSRNOG00000001442 | Por | cytochrome p450 oxidoreductase |  |
| rno-miR-127-5p | up | ENSRNOT00000050696 | ENSRNOG00000016684 | Wnk2 | WNK lysine deficient protein kinase 2 |  |
| rno-miR-370-3p | up | ENSRNOT00000035125 | ENSRNOG00000022921 | Dact2 | dishevelled-binding antagonist of beta-catenin 2 |  |
| rno-miR-541-5p | up | ENSRNOT00000045357 | ENSRNOG00000025528 | Efr3a | EFR3 homolog A |  |
| rno-miR-370-3p | up | ENSRNOT00000058727 | ENSRNOG00000010392 | Nrg1 | neuregulin 1 |  |
| rno-miR-370-3p | up | ENSRNOT00000090273 | ENSRNOG00000053288 | Ank3 | ankyrin 3 |  |
| rno-miR-370-3p | up | ENSRNOT00000085985 | ENSRNOG00000053288 | Ank3 | ankyrin 3 |  |
| rno-miR-665 | up | ENSRNOT00000087072 | ENSRNOG00000016647 | Pla2g2c | phospholipase A2, group IIC |  |
| rno-miR-370-3p | up | ENSRNOT00000064798 | ENSRNOG00000021061 | Map4k2 | mitogen activated protein kinase kinase kinase kinase 2 |  |
| rno-miR-127-5p | up | ENSRNOT00000059571 | ENSRNOG00000013408 | Npas2 | neuronal PAS domain protein 2 |  |
| rno-miR-370-3p | up | ENSRNOT00000093141 | ENSRNOG00000056716 | Zbtb20 | zinc finger and BTB domain containing 20 |  |
| rno-miR-370-3p | up | ENSRNOT00000077620 | ENSRNOG00000053288 | Ank3 | ankyrin 3 |  |
| rno-miR-673-3p | up | ENSRNOT00000065871 | ENSRNOG00000010237 | Map7d1 | MAP7 domain containing 1 |  |
| rno-miR-212-5p | up | ENSRNOT00000078158 | ENSRNOG00000052775 | RGD1305938 | similar to expressed sequence AW549877 |  |
| rno-miR-370-3p | up | ENSRNOT00000065827 | ENSRNOG00000012434 | Zfp598 | zinc finger protein 598 |  |
| rno-miR-665 | up | ENSRNOT00000007233 | ENSRNOG00000005451 | Dnah11 | dynein, axonemal, heavy chain 11 |  |
| rno-miR-370-3p | up | ENSRNOT00000035743 | ENSRNOG00000024793 | Kctd21 | potassium channel tetramerization domain containing 21 |  |
| rno-miR-212-5p | up | ENSRNOT00000089841 | ENSRNOG00000003745 | Atf3 | activating transcription factor 3 |  |
| rno-miR-665 | up | ENSRNOT00000010884 | ENSRNOG00000008052 | Atp13a2 | ATPase 13A2 |  |
| rno-miR-127-5p | up | ENSRNOT00000029994 | ENSRNOG00000022099 | Trim72 | tripartite motif containing 72 |  |
| rno-miR-665 | up | ENSRNOT00000077956 | ENSRNOG00000061348 | Fam53b | family with sequence similarity 53, member B |  |
| rno-miR-370-3p | up | ENSRNOT00000014268 | ENSRNOG00000010392 | Nrg1 | neuregulin 1 |  |
| rno-miR-665 | up | ENSRNOT00000090001 | ENSRNOG00000007801 | R3hdm2 | R3H domain containing 2 |  |
| rno-miR-214-3p | up | ENSRNOT00000083594 | ENSRNOG00000051204 | Dopey2 | dopey family member 2 |  |
| rno-miR-140-3p | up | ENSRNOT00000005584 | ENSRNOG00000004200 | Sybu | syntabulin |  |
| rno-miR-665 | up | ENSRNOT00000091317 | ENSRNOG00000022802 | Tmem184b | transmembrane protein 184B |  |
| rno-miR-665 | up | ENSRNOT00000073537 | ENSRNOG00000048973 | NEWGENE_1306399 | cyclin J |  |
| rno-miR-665 | up | ENSRNOT00000031117 | ENSRNOG00000022802 | Tmem184b | transmembrane protein 184B |  |
| rno-miR-370-3p | up | ENSRNOT00000077651 | ENSRNOG00000055371 | Sptbn4 | spectrin, beta, non-erythrocytic 4 |  |
| rno-miR-370-3p | up | ENSRNOT00000079697 | ENSRNOG00000060568 | Ptpn21 | protein tyrosine phosphatase, non-receptor type 21 |  |
| rno-miR-485-5p | up | ENSRNOT00000046155 | ENSRNOG00000017023 | Tcf25 | transcription factor 25 |  |
| rno-miR-370-3p | up | ENSRNOT00000022952 | ENSRNOG00000017102 | Cmtr2 | cap methyltransferase 2 |  |
| rno-miR-485-5p | up | ENSRNOT00000065693 | ENSRNOG00000038784 | Piezo2 | piezo-type mechanosensitive ion channel component 2 |  |
| rno-miR-214-3p | up | ENSRNOT00000047246 | ENSRNOG00000019723 | LOC100910990 | copine-1-like |  |
| rno-miR-370-3p | up | ENSRNOT00000029209 | ENSRNOG00000002180 | Tbc1d1 | TBC1 domain family member 1 |  |
| rno-miR-665 | up | ENSRNOT00000008730 | ENSRNOG00000006467 | Eif2b2 | eukaryotic translation initiation factor 2B subunit beta |  |
| rno-miR-665 | up | ENSRNOT00000011906 | ENSRNOG00000008915 | Prima1 | proline rich membrane anchor 1 |  |
| rno-miR-665 | up | ENSRNOT00000021407 | ENSRNOG00000015859 | Chdh | choline dehydrogenase |  |
| rno-miR-370-3p | up | ENSRNOT00000025699 | ENSRNOG00000018974 | Ern2 | endoplasmic reticulum to nucleus signaling 2 |  |
| rno-miR-370-3p | up | ENSRNOT00000012114 | ENSRNOG00000008981 | Pdcd6ip | programmed cell death 6 interacting protein |  |
| rno-miR-431 | up | ENSRNOT00000005987 | ENSRNOG00000004291 | Cltc | clathrin heavy chain |  |
| rno-miR-665 | up | ENSRNOT00000017871 | ENSRNOG00000013353 | Tmem260 | transmembrane protein 260 |  |
| rno-miR-370-3p | up | ENSRNOT00000034157 | ENSRNOG00000022249 | Mllt10 | myeloid/lymphoid or mixed-lineage leukemia; translocated to, 10 |  |
| rno-miR-214-3p | up | ENSRNOT00000016768 | ENSRNOG00000011951 | Plk2 | polo-like kinase 2 |  |
| rno-miR-212-5p | up | ENSRNOT00000010433 | ENSRNOG00000007763 | Plod1 | procollagen-lysine, 2-oxoglutarate 5-dioxygenase 1 |  |
| rno-miR-370-3p | up | ENSRNOT00000026893 | ENSRNOG00000019857 | Gng7 | G protein subunit gamma 7 |  |
| rno-miR-665 | up | ENSRNOT00000086374 | ENSRNOG00000033508 | Mink1 | misshapen-like kinase 1 |  |
| rno-miR-370-3p | up | ENSRNOT00000080065 | ENSRNOG00000022921 | Dact2 | dishevelled-binding antagonist of beta-catenin 2 |  |
| rno-miR-370-3p | up | ENSRNOT00000003320 | ENSRNOG00000002403 | Fam129a | family with sequence similarity 129, member A |  |
| rno-miR-370-3p | up | ENSRNOT00000019076 | ENSRNOG00000013969 | Ahi1 | Abelson helper integration site 1 |  |
| rno-miR-214-3p | up | ENSRNOT00000001292 | ENSRNOG00000000967 | Aacs | acetoacetyl-CoA synthetase |  |
| rno-miR-370-3p | up | ENSRNOT00000082181 | ENSRNOG00000053288 | Ank3 | ankyrin 3 |  |
| rno-miR-370-3p | up | ENSRNOT00000006143 | ENSRNOG00000004464 | Sel1l | SEL1L ERAD E3 ligase adaptor subunit |  |
| rno-miR-665 | up | ENSRNOT00000023889 | ENSRNOG00000017583 | Mapkap1 | mitogen-activated protein kinase associated protein 1 |  |
| rno-miR-212-5p | up | ENSRNOT00000081244 | ENSRNOG00000012337 | Pde1c | phosphodiesterase 1C |  |
| rno-miR-370-3p | up | ENSRNOT00000000169 | ENSRNOG00000000156 | LOC100911486 | multiple epidermal growth factor-like domains protein 6-like |  |
| rno-miR-665 | up | ENSRNOT00000074348 | ENSRNOG00000048004 | Garem2 | GRB2 associated regulator of MAPK1 subtype 2 |  |
| rno-miR-214-3p | up | ENSRNOT00000078429 | ENSRNOG00000060005 | Surf4 | surfeit 4 |  |
| rno-miR-370-3p | up | ENSRNOT00000066934 | ENSRNOG00000025418 | Armc9 | armadillo repeat containing 9 |  |
| rno-miR-665 | up | ENSRNOT00000023451 | ENSRNOG00000017448 | Exoc3l2 | exocyst complex component 3-like 2 |  |
| rno-miR-370-3p | up | ENSRNOT00000001570 | ENSRNOG00000001187 | Oasl | 2'-5'-oligoadenylate synthetase-like |  |
| rno-miR-370-3p | up | ENSRNOT00000009517 | ENSRNOG00000006952 | Prex1 | phosphatidylinositol-3,4,5-trisphosphate-dependent Rac exchange factor 1 |  |
| rno-miR-485-5p | up | ENSRNOT00000016621 | ENSRNOG00000012427 | Veph1 | ventricular zone expressed PH domain-containing 1 |  |
| rno-miR-665 | up | ENSRNOT00000061100 | ENSRNOG00000020828 | Pde4a | phosphodiesterase 4A |  |
| rno-miR-341 | up | ENSRNOT00000021750 | ENSRNOG00000016246 | Tshz1 | teashirt zinc finger homeobox 1 |  |
| rno-miR-665 | up | ENSRNOT00000040488 | ENSRNOG00000033508 | Mink1 | misshapen-like kinase 1 |  |
| rno-miR-370-3p | up | ENSRNOT00000039273 | ENSRNOG00000028501 | Zc3h18 | zinc finger CCCH-type containing 18 |  |
| rno-miR-370-3p | up | ENSRNOT00000021435 | ENSRNOG00000015928 | Dhx35 | DEAH-box helicase 35 |  |
| rno-miR-214-3p | up | ENSRNOT00000056508 | ENSRNOG00000009204 | Il17re | interleukin 17 receptor E |  |
| rno-miR-370-3p | up | ENSRNOT00000093382 | ENSRNOG00000056716 | Zbtb20 | zinc finger and BTB domain containing 20 |  |
| rno-miR-370-3p | up | ENSRNOT00000093179 | ENSRNOG00000056716 | Zbtb20 | zinc finger and BTB domain containing 20 |  |
| rno-miR-665 | up | ENSRNOT00000026439 | ENSRNOG00000019423 | Dvl1 | dishevelled segment polarity protein 1 |  |
| rno-miR-665 | up | ENSRNOT00000039631 | ENSRNOG00000028545 | Ahnak2 | AHNAK nucleoprotein 2 |  |
| rno-miR-665 | up | ENSRNOT00000077443 | ENSRNOG00000058193 | Slc27a6 | solute carrier family 27 member 6 |  |
| rno-miR-370-3p | up | ENSRNOT00000058429 | ENSRNOG00000008036 | Dennd4c | DENN domain containing 4C |  |
| rno-miR-370-3p | up | ENSRNOT00000082596 | ENSRNOG00000008036 | Dennd4c | DENN domain containing 4C |  |
| rno-miR-433-3p | up | ENSRNOT00000080108 | ENSRNOG00000061876 | Tas1r2 | taste 1 receptor member 2 |  |
| rno-miR-127-3p | up | ENSRNOT00000066777 | ENSRNOG00000007242 | Ehmt1 | euchromatic histone lysine methyltransferase 1 |  |
| rno-miR-370-3p | up | ENSRNOT00000084866 | ENSRNOG00000015928 | Dhx35 | DEAH-box helicase 35 |  |
| rno-miR-370-3p | up | ENSRNOT00000091417 | ENSRNOG00000000156 | LOC100911486 | multiple epidermal growth factor-like domains protein 6-like |  |
| rno-miR-370-3p | up | ENSRNOT00000018956 | ENSRNOG00000014163 | Zfp536 | zinc finger protein 536 |  |
| rno-miR-673-3p | up | ENSRNOT00000037624 | ENSRNOG00000027264 | Dagla | diacylglycerol lipase, alpha |  |
| rno-miR-204-5p | up | ENSRNOT00000009839 | ENSRNOG00000006803 | Dnajc10 | DnaJ heat shock protein family (Hsp40) member C10 |  |
| rno-miR-370-3p | up | ENSRNOT00000045224 | ENSRNOG00000016671 | Dtna | dystrobrevin, alpha |  |
| rno-miR-140-3p | up | ENSRNOT00000080522 | ENSRNOG00000014079 | Stat1 | signal transducer and activator of transcription 1 |  |
| rno-miR-370-3p | up | ENSRNOT00000054913 | ENSRNOG00000036658 | Tbcd | tubulin folding cofactor D |  |
| rno-miR-485-5p | up | ENSRNOT00000004412 | ENSRNOG00000003302 | Flcn | folliculin |  |
| rno-miR-665 | up | ENSRNOT00000028262 | ENSRNOG00000020813 | Ltbp3 | latent transforming growth factor beta binding protein 3 |  |
| rno-miR-370-3p | up | ENSRNOT00000086841 | ENSRNOG00000060367 | Rfx7 | regulatory factor X, 7 |  |
| rno-miR-370-3p | up | ENSRNOT00000014553 | ENSRNOG00000010959 | Klhl25 | kelch-like family member 25 |  |
| rno-miR-370-3p | up | ENSRNOT00000045870 | ENSRNOG00000004464 | Sel1l | SEL1L ERAD E3 ligase adaptor subunit |  |
| rno-miR-370-3p | up | ENSRNOT00000079630 | ENSRNOG00000053288 | Ank3 | ankyrin 3 |  |
| rno-miR-370-3p | up | ENSRNOT00000028094 | ENSRNOG00000020702 | Cyb561a3 | cytochrome b561 family, member A3 |  |
| rno-miR-665 | up | ENSRNOT00000012363 | ENSRNOG00000009094 | Nudt4 | nudix hydrolase 4 |  |
| rno-miR-370-3p | up | ENSRNOT00000042623 | ENSRNOG00000032437 | Pard3 | par-3 family cell polarity regulator |  |
| rno-miR-370-3p | up | ENSRNOT00000000891 | ENSRNOG00000000702 | Sart3 | squamous cell carcinoma antigen recognized by T-cells 3 |  |
| rno-miR-370-3p | up | ENSRNOT00000085336 | ENSRNOG00000019728 | Itgad | integrin subunit alpha D |  |
| rno-miR-370-3p | up | ENSRNOT00000055386 | ENSRNOG00000000156 | LOC100911486 | multiple epidermal growth factor-like domains protein 6-like |  |
| rno-miR-665 | up | ENSRNOT00000068493 | ENSRNOG00000018526 | Dlg4 | discs large MAGUK scaffold protein 4 |  |
| rno-miR-370-3p | up | ENSRNOT00000022485 | ENSRNOG00000016653 | Ngef | neuronal guanine nucleotide exchange factor |  |
| rno-miR-127-3p | up | ENSRNOT00000072399 | ENSRNOG00000048751 | Rtl1 | retrotransposon-like 1 |  |
| rno-miR-122-5p | up | ENSRNOT00000022932 | ENSRNOG00000017092 | Zfp169 | zinc finger protein 169 |  |
| rno-miR-665 | up | ENSRNOT00000059426 | ENSRNOG00000038883 | LOC294154 | similar to chromosome 6 open reading frame 106 isoform a |  |
| rno-miR-370-3p | up | ENSRNOT00000016790 | ENSRNOG00000012164 | Plppr2 | phospholipid phosphatase related 2 |  |
| rno-miR-127-3p | up | ENSRNOT00000091370 | ENSRNOG00000018651 | Agtpbp1 | ATP/GTP binding protein 1 |  |
| rno-miR-370-3p | up | ENSRNOT00000093596 | ENSRNOG00000056716 | Zbtb20 | zinc finger and BTB domain containing 20 |  |
| rno-miR-127-3p | up | ENSRNOT00000086843 | ENSRNOG00000007242 | Ehmt1 | euchromatic histone lysine methyltransferase 1 |  |
| rno-miR-673-3p | up | ENSRNOT00000022461 | ENSRNOG00000016756 | Ptgir | prostaglandin I2 (prostacyclin) receptor (IP) |  |
| rno-miR-212-5p | up | ENSRNOT00000043623 | ENSRNOG00000032946 | Pdzd7 | PDZ domain containing 7 |  |
| rno-miR-665 | up | ENSRNOT00000089004 | ENSRNOG00000020501 | Tjp3 | tight junction protein 3 |  |
| rno-miR-665 | up | ENSRNOT00000064160 | ENSRNOG00000042620 | Marveld1 | MARVEL domain containing 1 |  |
| rno-miR-665 | up | ENSRNOT00000079909 | ENSRNOG00000057706 | Kdm5c | lysine demethylase 5C |  |
| rno-miR-665 | up | ENSRNOT00000087945 | ENSRNOG00000057706 | Kdm5c | lysine demethylase 5C |  |
| rno-miR-665 | up | ENSRNOT00000077528 | ENSRNOG00000057706 | Kdm5c | lysine demethylase 5C |  |
| rno-miR-127-5p | up | ENSRNOT00000072399 | ENSRNOG00000048751 | Rtl1 | retrotransposon-like 1 |  |
| rno-miR-370-3p | up | ENSRNOT00000074810 | ENSRNOG00000050742 | Dnmbp | dynamin binding protein |  |
| rno-miR-665 | up | ENSRNOT00000013053 | ENSRNOG00000009381 | Mapk6 | mitogen-activated protein kinase 6 |  |
| rno-miR-370-3p | up | ENSRNOT00000011223 | ENSRNOG00000008278 | Nfrkb | nuclear factor related to kappa B binding protein |  |
| rno-miR-370-3p | up | ENSRNOT00000009345 | ENSRNOG00000006934 | Acvr1b | activin A receptor type 1B |  |
| rno-miR-370-3p | up | ENSRNOT00000011898 | ENSRNOG00000008533 | Ago2 | argonaute 2, RISC catalytic component |  |
| rno-miR-673-3p | up | ENSRNOT00000093357 | ENSRNOG00000016640 | Dner | delta/notch-like EGF repeat containing |  |
| rno-miR-214-3p | up | ENSRNOT00000045410 | ENSRNOG00000029572 | Apeh | acylaminoacyl-peptide hydrolase |  |
| rno-miR-370-3p | up | ENSRNOT00000001292 | ENSRNOG00000000967 | Aacs | acetoacetyl-CoA synthetase |  |
| rno-miR-370-3p | up | ENSRNOT00000034096 | ENSRNOG00000024089 | Fndc3b | fibronectin type III domain containing 3B |  |
| rno-miR-665 | up | ENSRNOT00000050721 | ENSRNOG00000020651 | Cars | cysteinyl-tRNA synthetase |  |
| rno-miR-370-3p | up | ENSRNOT00000003991 | ENSRNOG00000002932 | Wdr19 | WD repeat domain 19 |  |
| rno-miR-370-3p | up | ENSRNOT00000081443 | ENSRNOG00000056716 | Zbtb20 | zinc finger and BTB domain containing 20 |  |
| rno-miR-665 | up | ENSRNOT00000030440 | ENSRNOG00000029022 | Zfp112 | zinc finger protein 112 |  |
| rno-miR-665 | up | ENSRNOT00000087253 | ENSRNOG00000029022 | Zfp112 | zinc finger protein 112 |  |
| rno-miR-370-3p | up | ENSRNOT00000004632 | ENSRNOG00000003407 | Ptpn14 | protein tyrosine phosphatase, non-receptor type 14 |  |
| rno-miR-370-3p | up | ENSRNOT00000086003 | ENSRNOG00000019728 | Itgad | integrin subunit alpha D |  |
| rno-miR-431 | up | ENSRNOT00000014861 | ENSRNOG00000010986 | Cmtm7 | CKLF-like MARVEL transmembrane domain containing 7 |  |
| rno-miR-370-3p | up | ENSRNOT00000035758 | ENSRNOG00000025781 | Sec31b | SEC31 homolog B, COPII coat complex component |  |
| rno-miR-665 | up | ENSRNOT00000084218 | ENSRNOG00000060946 | LOC100910506 | peripheral plasma membrane protein CASK-like |  |
| rno-miR-370-3p | up | ENSRNOT00000048543 | ENSRNOG00000009350 | Sez6 | seizure related 6 homolog |  |
| rno-miR-370-3p | up | ENSRNOT00000089319 | ENSRNOG00000046428 | Lrrc75b | leucine rich repeat containing 75B |  |
| rno-miR-370-3p | up | ENSRNOT00000000468 | ENSRNOG00000000412 | Slc35f1 | solute carrier family 35, member F1 |  |
| rno-miR-370-3p | up | ENSRNOT00000082355 | ENSRNOG00000010392 | Nrg1 | neuregulin 1 |  |
| rno-miR-370-3p | up | ENSRNOT00000093719 | ENSRNOG00000060729 | Qtrt2 | queuine tRNA-ribosyltransferase accessory subunit 2 |  |
| rno-miR-665 | up | ENSRNOT00000035597 | ENSRNOG00000025679 | Stk40 | serine/threonine kinase 40 |  |
| rno-miR-212-5p | up | ENSRNOT00000042623 | ENSRNOG00000032437 | Pard3 | par-3 family cell polarity regulator |  |
| rno-miR-370-3p | up | ENSRNOT00000081522 | ENSRNOG00000010392 | Nrg1 | neuregulin 1 |  |
| rno-miR-214-3p | up | ENSRNOT00000020603 | ENSRNOG00000014819 | Hap1 | huntingtin-associated protein 1 |  |
| rno-miR-370-3p | up | ENSRNOT00000064290 | ENSRNOG00000010922 | Ppp2r1b | protein phosphatase 2 scaffold subunit A beta |  |
| rno-miR-214-3p | up | ENSRNOT00000020390 | ENSRNOG00000015051 | Golga7b | golgin A7 family, member B |  |
| rno-miR-370-3p | up | ENSRNOT00000068689 | ENSRNOG00000027747 | Ninl | ninein-like |  |
| rno-miR-665 | up | ENSRNOT00000081242 | ENSRNOG00000015835 | Cacna2d2 | calcium voltage-gated channel auxiliary subunit alpha2delta 2 |  |
| rno-miR-665 | up | ENSRNOT00000006969 | ENSRNOG00000005093 | Lgr6 | leucine-rich repeat-containing G protein-coupled receptor 6 |  |
| rno-miR-370-3p | up | ENSRNOT00000086720 | ENSRNOG00000056009 | Rab11fip5 | RAB11 family interacting protein 5 |  |
| rno-miR-665 | up | ENSRNOT00000009323 | ENSRNOG00000007017 | Tmc2 | transmembrane channel-like 2 |  |
| rno-miR-140-3p | up | ENSRNOT00000015778 | ENSRNOG00000011704 | Fbxo34 | F-box protein 34 |  |
| rno-miR-214-3p | up | ENSRNOT00000068295 | ENSRNOG00000029195 | Uba7 | ubiquitin-like modifier activating enzyme 7 |  |
| rno-miR-370-3p | up | ENSRNOT00000073019 | ENSRNOG00000047896 | Kctd10 | potassium channel tetramerization domain containing 10 |  |
| rno-miR-370-3p | up | ENSRNOT00000086775 | ENSRNOG00000003407 | Ptpn14 | protein tyrosine phosphatase, non-receptor type 14 |  |
| rno-miR-6331 | up | ENSRNOT00000093640 | ENSRNOG00000011752 | Sh3d19 | SH3 domain containing 19 |  |
| rno-miR-665 | up | ENSRNOT00000088351 | ENSRNOG00000007981 | Klhl23 | kelch-like family member 23 |  |
| rno-miR-434-5p | up | ENSRNOT00000009620 | ENSRNOG00000006227 | Ifih1 | interferon induced with helicase C domain 1 |  |
| rno-miR-665 | up | ENSRNOT00000018000 | ENSRNOG00000013474 | Casz1 | castor zinc finger 1 |  |
| rno-miR-214-3p | up | ENSRNOT00000067840 | ENSRNOG00000018666 | Gpsm1 | G-protein signaling modulator 1 |  |
| rno-miR-370-3p | up | ENSRNOT00000011432 | ENSRNOG00000008602 | Steap4 | STEAP4 metalloreductase |  |
| rno-miR-370-3p | up | ENSRNOT00000056166 | ENSRNOG00000020748 | Map4 | microtubule-associated protein 4 |  |
| rno-miR-665 | up | ENSRNOT00000041238 | ENSRNOG00000020299 | Klc2 | kinesin light chain 2 |  |
| rno-miR-665 | up | ENSRNOT00000035908 | ENSRNOG00000026163 | Cpt1c | carnitine palmitoyltransferase 1c |  |
| rno-miR-214-3p | up | ENSRNOT00000014235 | ENSRNOG00000010633 | Acsl1 | acyl-CoA synthetase long-chain family member 1 |  |
| rno-miR-127-3p | up | ENSRNOT00000025381 | ENSRNOG00000018651 | Agtpbp1 | ATP/GTP binding protein 1 |  |
| rno-miR-214-3p | up | ENSRNOT00000068351 | ENSRNOG00000005099 | Top3a | topoisomerase (DNA) III alpha |  |
| rno-novel-125-mature | up | ENSRNOT00000001533 | ENSRNOG00000001158 | Abcg1 | ATP binding cassette subfamily G member 1 |  |
| rno-miR-665 | up | ENSRNOT00000004361 | ENSRNOG00000003268 | Maml1 | mastermind-like transcriptional coactivator 1 |  |
| rno-miR-370-3p | up | ENSRNOT00000083659 | ENSRNOG00000008278 | Nfrkb | nuclear factor related to kappa B binding protein |  |
| rno-miR-370-3p | up | ENSRNOT00000022346 | ENSRNOG00000016253 | Slc6a18 | solute carrier family 6 member 18 |  |
| rno-miR-127-3p | up | ENSRNOT00000014553 | ENSRNOG00000010959 | Klhl25 | kelch-like family member 25 |  |
| rno-miR-665 | up | ENSRNOT00000091382 | ENSRNOG00000060946 | LOC100910506 | peripheral plasma membrane protein CASK-like |  |
| rno-miR-665 | up | ENSRNOT00000078816 | ENSRNOG00000026985 | Phldb1 | pleckstrin homology-like domain, family B, member 1 |  |
| rno-miR-665 | up | ENSRNOT00000030224 | ENSRNOG00000008463 | Rundc3b | RUN domain containing 3B |  |
| rno-miR-485-5p | up | ENSRNOT00000088559 | ENSRNOG00000012427 | Veph1 | ventricular zone expressed PH domain-containing 1 |  |
| rno-miR-665 | up | ENSRNOT00000005522 | ENSRNOG00000004132 | Lasp1 | LIM and SH3 protein 1 |  |
| rno-miR-370-3p | up | ENSRNOT00000013991 | ENSRNOG00000010392 | Nrg1 | neuregulin 1 |  |
| rno-miR-370-3p | up | ENSRNOT00000078475 | ENSRNOG00000052354 | Arhgef40 | Rho guanine nucleotide exchange factor 40 |  |
| rno-miR-665 | up | ENSRNOT00000045315 | ENSRNOG00000001229 | Col18a1 | collagen type XVIII alpha 1 chain |  |
| rno-miR-370-3p | up | ENSRNOT00000072241 | ENSRNOG00000050204 | Naif1 | nuclear apoptosis inducing factor 1 |  |
| rno-miR-370-3p | up | ENSRNOT00000000572 | ENSRNOG00000000483 | Syngap1 | synaptic Ras GTPase activating protein 1 |  |
| rno-miR-370-3p | up | ENSRNOT00000085727 | ENSRNOG00000061928 | Myo1e | myosin IE |  |
| rno-miR-370-3p | up | ENSRNOT00000009437 | ENSRNOG00000007160 | Kat14 | lysine acetyltransferase 14 |  |
| rno-miR-370-3p | up | ENSRNOT00000082463 | ENSRNOG00000059445 | Aifm2 | apoptosis inducing factor, mitochondria associated 2 |  |
| rno-miR-132-5p | up | ENSRNOT00000025009 | ENSRNOG00000018242 | Camkk1 | calcium/calmodulin-dependent protein kinase kinase 1 |  |
| rno-miR-370-3p | up | ENSRNOT00000051065 | ENSRNOG00000022911 | Hjurp | Holliday junction recognition protein |  |
| rno-miR-370-3p | up | ENSRNOT00000016273 | ENSRNOG00000011058 | Utrn | utrophin |  |
| rno-miR-127-5p | up | ENSRNOT00000009489 | ENSRNOG00000007202 | Sema3d | semaphorin 3D |  |
| rno-miR-370-3p | up | ENSRNOT00000085547 | ENSRNOG00000000463 | Col11a2 | collagen type XI alpha 2 chain |  |
| rno-miR-673-3p | up | ENSRNOT00000067939 | ENSRNOG00000042855 | Ahdc1 | AT hook, DNA binding motif, containing 1 |  |
| rno-miR-140-3p | up | ENSRNOT00000092161 | ENSRNOG00000011704 | Fbxo34 | F-box protein 34 |  |
| rno-miR-214-3p | up | ENSRNOT00000021134 | ENSRNOG00000015236 | Mybbp1a | MYB binding protein 1a |  |
| rno-miR-214-3p | up | ENSRNOT00000089060 | ENSRNOG00000059605 | Ddn | dendrin |  |
| rno-miR-214-3p | up | ENSRNOT00000084429 | ENSRNOG00000029195 | Uba7 | ubiquitin-like modifier activating enzyme 7 |  |
| rno-miR-665 | up | ENSRNOT00000065827 | ENSRNOG00000012434 | Zfp598 | zinc finger protein 598 |  |
| rno-miR-370-3p | up | ENSRNOT00000086574 | ENSRNOG00000011058 | Utrn | utrophin |  |
| rno-miR-665 | up | ENSRNOT00000021218 | ENSRNOG00000015835 | Cacna2d2 | calcium voltage-gated channel auxiliary subunit alpha2delta 2 |  |
| rno-miR-665 | up | ENSRNOT00000072208 | ENSRNOG00000045952 | Ephb4 | EPH receptor B4 |  |
| rno-miR-485-5p | up | ENSRNOT00000085440 | ENSRNOG00000031773 | Cngb1 | cyclic nucleotide gated channel beta 1 |  |
| rno-miR-485-5p | up | ENSRNOT00000064115 | ENSRNOG00000001149 | Pxn | paxillin |  |
| rno-miR-127-3p | up | ENSRNOT00000026514 | ENSRNOG00000019590 | Smg5 | SMG5 nonsense mediated mRNA decay factor |  |
| rno-miR-370-3p | up | ENSRNOT00000024866 | ENSRNOG00000023317 | Colgalt1 | collagen beta(1-O)galactosyltransferase 1 |  |
| rno-miR-665 | up | ENSRNOT00000085630 | ENSRNOG00000005747 | Il27ra | interleukin 27 receptor subunit alpha |  |
| rno-miR-370-3p | up | ENSRNOT00000057386 | ENSRNOG00000016281 | Col4a1 | collagen type IV alpha 1 chain |  |
| rno-miR-370-3p | up | ENSRNOT00000073087 | ENSRNOG00000050834 | Fam102a | family with sequence similarity 102, member A |  |
| rno-miR-665 | up | ENSRNOT00000007079 | ENSRNOG00000005330 | Crebbp | CREB binding protein |  |
| rno-miR-370-3p | up | ENSRNOT00000012289 | ENSRNOG00000009239 | Entpd8 | ectonucleoside triphosphate diphosphohydrolase 8 |  |
| rno-miR-370-3p | up | ENSRNOT00000040859 | ENSRNOG00000000483 | Syngap1 | synaptic Ras GTPase activating protein 1 ] |  |
| rno-miR-541-3p | up | ENSRNOT00000086550 | ENSRNOG00000023781 | Plec | plectin |  |
| rno-miR-665 | up | ENSRNOT00000022290 | ENSRNOG00000023548 | Sned1 | sushi, nidogen and EGF-like domains 1 |  |
| rno-miR-665 | up | ENSRNOT00000029508 | ENSRNOG00000027489 | Mn1 | meningioma 1 |  |
| rno-miR-541-3p | up | ENSRNOT00000091285 | ENSRNOG00000023781 | Plec | plectin |  |
| rno-miR-673-3p | up | ENSRNOT00000011609 | ENSRNOG00000008607 | U2surp | U2 snRNP-associated SURP domain containing |  |
| rno-miR-370-3p | up | ENSRNOT00000030750 | ENSRNOG00000028215 | Nup160 | nucleoporin 160 |  |
| rno-miR-370-3p | up | ENSRNOT00000078559 | ENSRNOG00000032437 | Pard3 | par-3 family cell polarity regulator |  |
| rno-miR-370-3p | up | ENSRNOT00000043545 | ENSRNOG00000001875 | Smpd4 | sphingomyelin phosphodiesterase 4 [Source:RGD Symbol;Acc:1310674] |  |
| rno-miR-370-3p | up | ENSRNOT00000068340 | ENSRNOG00000019677 | Arid3b | AT-rich interaction domain 3B |  |
| rno-miR-541-3p | up | ENSRNOT00000088945 | ENSRNOG00000023781 | Plec | plectin |  |
| rno-miR-485-5p | up | ENSRNOT00000047466 | ENSRNOG00000001149 | Pxn | paxillin |  |
| rno-miR-140-3p | up | ENSRNOT00000074448 | ENSRNOG00000018226 | Zcchc14 | zinc finger CCHC-type containing 14 |  |
| rno-miR-485-5p | up | ENSRNOT00000008573 | ENSRNOG00000006570 | Plekhg3 | pleckstrin homology and RhoGEF domain containing G3 |  |
| rno-miR-370-3p | up | ENSRNOT00000000481 | ENSRNOG00000000421 | Skiv2l | Ski2 like RNA helicase |  |
| rno-miR-541-3p | up | ENSRNOT00000091840 | ENSRNOG00000023781 | Plec | plectin |  |
| rno-miR-673-3p | up | ENSRNOT00000057120 | ENSRNOG00000042899 | Fcgbpl1 | Fc fragment of IgG binding protein-like 1 |  |
| rno-miR-541-3p | up | ENSRNOT00000042642 | ENSRNOG00000023781 | Plec | plectin |  |
| rno-miR-541-3p | up | ENSRNOT00000081021 | ENSRNOG00000023781 | Plec | plectin |  |
| rno-miR-212-5p | up | ENSRNOT00000027574 | ENSRNOG00000020353 | Sh3pxd2a | SH3 and PX domains 2A |  |
| rno-miR-370-3p | up | ENSRNOT00000014022 | ENSRNOG00000010161 | Myo10 | myosin X |  |
| rno-miR-370-3p | up | ENSRNOT00000015641 | ENSRNOG00000011702 | Foxp3 | forkhead box P3 |  |
| rno-miR-127-3p | up | ENSRNOT00000020042 | ENSRNOG00000014856 | Etnk1 | ethanolamine kinase 1 |  |
| rno-miR-665 | up | ENSRNOT00000072841 | ENSRNOG00000050240 | LOC100909856 | metal regulatory transcription factor 1-like |  |
| rno-miR-370-3p | up | ENSRNOT00000016983 | ENSRNOG00000012531 | Ephb2 | Eph receptor B2 |  |
| rno-miR-541-3p | up | ENSRNOT00000082271 | ENSRNOG00000023781 | Plec | plectin |  |
| rno-miR-127-5p | up | ENSRNOT00000030914 | ENSRNOG00000025167 | Sema4b | semaphorin 4B |  |
| rno-miR-665 | up | ENSRNOT00000031928 | ENSRNOG00000025724 | Mtf1 | metal-regulatory transcription factor 1 |  |
| rno-miR-370-3p | up | ENSRNOT00000065897 | ENSRNOG00000010161 | Myo10 | myosin X |  |
| rno-miR-665 | up | ENSRNOT00000027578 | ENSRNOG00000020310 | Grik5 | glutamate ionotropic receptor kainate type subunit 5 |  |
| rno-miR-370-3p | up | ENSRNOT00000083448 | ENSRNOG00000052707 | Cacna1a | calcium voltage-gated channel subunit alpha1 A |  |
| rno-miR-665 | up | ENSRNOT00000093103 | ENSRNOG00000012634 | Fbxo10 | F-box protein 10 |  |
| rno-miR-541-3p | up | ENSRNOT00000006311 | ENSRNOG00000023781 | Plec | plectin |  |
| rno-miR-673-3p | up | ENSRNOT00000046873 | ENSRNOG00000008607 | U2surp | U2 snRNP-associated SURP domain containing |  |
| rno-miR-370-3p | up | ENSRNOT00000087548 | ENSRNOG00000059015 | Triobp | TRIO and F-actin binding protein |  |
| rno-miR-214-3p | up | ENSRNOT00000044293 | ENSRNOG00000031266 | Siae | sialic acid acetylesterase |  |
| rno-miR-127-5p | up | ENSRNOT00000036646 | ENSRNOG00000004279 | Adgra3 | adhesion G protein-coupled receptor A3 |  |
| rno-miR-370-3p | up | ENSRNOT00000089574 | ENSRNOG00000012531 | Ephb2 | Eph receptor B2 |  |
| rno-miR-370-3p | up | ENSRNOT00000046054 | ENSRNOG00000010890 | Bmp1 | bone morphogenetic protein 1 |  |
| rno-miR-370-3p | up | ENSRNOT00000045533 | ENSRNOG00000000463 | Col11a2 | collagen type XI alpha 2 chain |  |
| rno-miR-212-5p | up | ENSRNOT00000078559 | ENSRNOG00000032437 | Pard3 | par-3 family cell polarity regulator |  |
| rno-miR-665 | up | ENSRNOT00000088082 | ENSRNOG00000008052 | Atp13a2 | ATPase 13A2 |  |
| rno-miR-370-3p | up | ENSRNOT00000080945 | ENSRNOG00000061230 | L1cam | L1 cell adhesion molecule |  |
| rno-miR-370-3p | up | ENSRNOT00000027430 | ENSRNOG00000020239 | Capn15 | calpain 15 |  |
| rno-miR-370-3p | up | ENSRNOT00000010755 | ENSRNOG00000007957 | Tspoap1 | TSPO associated protein 1 |  |
| rno-miR-370-3p | up | ENSRNOT00000091529 | ENSRNOG00000007160 | Kat14 | lysine acetyltransferase 14 |  |
| rno-miR-370-3p | up | ENSRNOT00000064212 | ENSRNOG00000023107 | Yeats2 | YEATS domain containing 2 |  |
| rno-miR-370-3p | up | ENSRNOT00000090114 | ENSRNOG00000024934 | Npat | nuclear protein, co-activator of histone transcription |  |
| rno-miR-212-5p | up | ENSRNOT00000082799 | ENSRNOG00000020353 | Sh3pxd2a | SH3 and PX domains 2A |  |
| rno-miR-370-3p | up | ENSRNOT00000046215 | ENSRNOG00000028872 | Rai14 | retinoic acid induced 14 |  |
| rno-miR-127-5p | up | ENSRNOT00000034102 | ENSRNOG00000025634 | Zfhx2 | zinc finger homeobox 2 |  |
| rno-miR-485-5p | up | ENSRNOT00000009914 | ENSRNOG00000007025 | Evc2 | EvC ciliary complex subunit 2 |  |
| rno-miR-370-3p | up | ENSRNOT00000075327 | ENSRNOG00000047575 | Dhx34 | DExH-box helicase 34 |  |
| rno-miR-431 | up | ENSRNOT00000065145 | ENSRNOG00000024711 | Sdk2 | sidekick cell adhesion molecule 2 |  |
| rno-miR-673-3p | up | ENSRNOT00000078799 | ENSRNOG00000013962 | Magi2 | membrane associated guanylate kinase, WW and PDZ domain containing 2 |  |
| rno-miR-665 | up | ENSRNOT00000034102 | ENSRNOG00000025634 | Zfhx2 | zinc finger homeobox 2 |  |
| rno-miR-370-3p | up | ENSRNOT00000089718 | ENSRNOG00000047575 | Dhx34 | DExH-box helicase 34 |  |
| rno-miR-214-3p | up | ENSRNOT00000090702 | ENSRNOG00000014819 | Hap1 | huntingtin-associated protein 1 |  |
| rno-miR-541-3p | up | ENSRNOT00000040762 | ENSRNOG00000023781 | Plec | plectin |  |
| rno-miR-493-3p | up | ENSRNOT00000040836 | ENSRNOG00000034022 | Taf2 | TATA-box binding protein associated factor 2 |  |
| rno-miR-370-3p | up | ENSRNOT00000078703 | ENSRNOG00000008895 | Hnf4a | hepatocyte nuclear factor 4, alpha |  |
| rno-miR-127-3p | up | ENSRNOT00000004632 | ENSRNOG00000003407 | Ptpn14 | protein tyrosine phosphatase, non-receptor type 14 |  |
| rno-miR-370-3p | up | ENSRNOT00000080962 | ENSRNOG00000015528 | Pja2 | praja ring finger ubiquitin ligase 2 |  |
| rno-miR-370-3p | up | ENSRNOT00000064171 | ENSRNOG00000018395 | Usp40 | ubiquitin specific peptidase 40 |  |
| rno-miR-370-3p | up | ENSRNOT00000012702 | ENSRNOG00000009430 | Gnl2 | G protein nucleolar 2 |  |
| rno-miR-370-3p | up | ENSRNOT00000089893 | ENSRNOG00000008895 | Hnf4a | hepatocyte nuclear factor 4, alpha |  |
| rno-miR-665 | up | ENSRNOT00000016875 | ENSRNOG00000012634 | Fbxo10 | F-box protein 10 |  |
| rno-miR-370-3p | up | ENSRNOT00000045657 | ENSRNOG00000028501 | Zc3h18 | zinc finger CCCH-type containing 18 |  |
| rno-miR-127-3p | up | ENSRNOT00000011633 | ENSRNOG00000008190 | Pnpla7 | patatin-like phospholipase domain containing 7 |  |
| rno-miR-370-3p | up | ENSRNOT00000036910 | ENSRNOG00000018823 | Nisch | nischarin |  |
| rno-miR-370-3p | up | ENSRNOT00000034298 | ENSRNOG00000027194 | Sgsm2 | small G protein signaling modulator 2 |  |
| rno-miR-127-3p | up | ENSRNOT00000086775 | ENSRNOG00000003407 | Ptpn14 | protein tyrosine phosphatase, non-receptor type 14 |  |
| rno-miR-673-3p | up | ENSRNOT00000066643 | ENSRNOG00000013962 | Magi2 | membrane associated guanylate kinase, WW and PDZ domain containing 2 |  |
| rno-miR-370-3p | up | ENSRNOT00000082289 | ENSRNOG00000055531 | Saa4 | serum amyloid A4 |  |
| rno-miR-370-3p | up | ENSRNOT00000086796 | ENSRNOG00000030705 | Cpsf1 | cleavage and polyadenylation specific factor 1 |  |
| rno-miR-665 | up | ENSRNOT00000078857 | ENSRNOG00000014589 | Arpin | actin-related protein 2/3 complex inhibitor |  |
| rno-miR-370-3p | up | ENSRNOT00000083016 | ENSRNOG00000055300 | Ncapd2 | non-SMC condensin I complex, subunit D2 |  |
| rno-miR-370-3p | up | ENSRNOT00000066736 | ENSRNOG00000043102 | Bahcc1 | BAH domain and coiled-coil containing 1 |  |
| rno-miR-370-3p | up | ENSRNOT00000056790 | ENSRNOG00000037505 | Ulk1 | unc-51 like autophagy activating kinase 1 |  |
| rno-miR-665 | up | ENSRNOT00000081616 | ENSRNOG00000056092 | Hephl1 | hephaestin-like 1 |  |
| rno-miR-370-3p | up | ENSRNOT00000068328 | ENSRNOG00000019154 | Zmynd8 | zinc finger, MYND-type containing 8 |  |
| rno-miR-140-5p | up | ENSRNOT00000049245 | ENSRNOG00000012367 | Pcdh7 | protocadherin 7 |  |
| rno-novel-58-mature | up | ENSRNOT00000002921 | ENSRNOG00000002135 | Sgcb | sarcoglycan, beta |  |
| rno-miR-370-3p | up | ENSRNOT00000066244 | ENSRNOG00000030705 | Cpsf1 | cleavage and polyadenylation specific factor 1 |  |
| rno-miR-31a-5p | up | ENSRNOT00000037251 | ENSRNOG00000025164 | Bhlha15 | basic helix-loop-helix family, member a15 |  |
| rno-miR-370-3p | up | ENSRNOT00000047482 | ENSRNOG00000031816 | Nckipsd | NCK interacting protein with SH3 domain |  |
| rno-miR-370-3p | up | ENSRNOT00000024831 | ENSRNOG00000018359 | Smad7 | SMAD family member 7 |  |
| rno-miR-127-3p | up | ENSRNOT00000011026 | ENSRNOG00000007975 | Ncoa2 | nuclear receptor coactivator 2 |  |
| rno-miR-212-5p | up | ENSRNOT00000002867 | ENSRNOG00000002093 | Tgfbr3 | transforming growth factor beta receptor 3 |  |
| rno-miR-665 | up | ENSRNOT00000028786 | ENSRNOG00000018372 | Cul9 | cullin 9 |  |
| rno-miR-665 | up | ENSRNOT00000066477 | ENSRNOG00000024594 | Fhdc1 | FH2 domain containing 1 |  |
| rno-miR-370-3p | up | ENSRNOT00000020154 | ENSRNOG00000014874 | Zfyve28 | zinc finger FYVE-type containing 28 |  |
| rno-miR-370-3p | up | ENSRNOT00000011551 | ENSRNOG00000008629 | Secisbp2l | SECIS binding protein 2-like |  |
| rno-miR-370-3p | up | ENSRNOT00000034692 | ENSRNOG00000023337 | Sema3a | semaphorin 3A |  |
| rno-miR-370-3p | up | ENSRNOT00000020717 | ENSRNOG00000015026 | Arhgef11 | Rho guanine nucleotide exchange factor 11 |  |
| rno-miR-370-3p | up | ENSRNOT00000049076 | ENSRNOG00000024934 | Npat | nuclear protein, co-activator of histone transcription |  |
| rno-miR-370-3p | up | ENSRNOT00000085325 | ENSRNOG00000018395 | Usp40 | ubiquitin specific peptidase 40 |  |
| rno-miR-140-3p | up | ENSRNOT00000037390 | ENSRNOG00000022938 | Ap4e1 | adaptor-related protein complex 4, epsilon 1 subunit |  |
| rno-miR-370-3p | up | ENSRNOT00000012463 | ENSRNOG00000008936 | Map3k6 | mitogen-activated protein kinase kinase kinase 6 |  |
| rno-miR-485-5p | up | ENSRNOT00000055137 | ENSRNOG00000006548 | Mrc2 | mannose receptor, C type 2 |  |
| rno-miR-370-3p | up | ENSRNOT00000079261 | ENSRNOG00000008129 | Pla2r1 | phospholipase A2 receptor 1 |  |
| rno-miR-370-3p | up | ENSRNOT00000092314 | ENSRNOG00000027194 | Sgsm2 | small G protein signaling modulator 2 |  |
| rno-miR-127-3p | up | ENSRNOT00000016376 | ENSRNOG00000011945 | Cyfip1 | cytoplasmic FMR1 interacting protein 1 |  |
| rno-miR-127-5p | up | ENSRNOT00000022497 | ENSRNOG00000016760 | Kctd19 | potassium channel tetramerization domain containing 19 |  |
| rno-miR-337-5p | up | ENSRNOT00000058241 | ENSRNOG00000021800 | Zfp541 | zinc finger protein 541 |  |
| rno-miR-665 | up | ENSRNOT00000018584 | ENSRNOG00000013887 | Adra2b | adrenoceptor alpha 2B |  |
| rno-miR-673-3p | up | ENSRNOT00000084959 | ENSRNOG00000060105 | Astn2 | astrotactin 2 |  |
| rno-miR-370-3p | up | ENSRNOT00000047763 | ENSRNOG00000023152 | Tmem201 | transmembrane protein 201 |  |
| rno-miR-370-3p | up | ENSRNOT00000078403 | ENSRNOG00000052894 | Epg5 | ectopic P-granules autophagy protein 5 homolog |  |
| rno-miR-665 | up | ENSRNOT00000080815 | ENSRNOG00000018372 | Cul9 | cullin 9 |  |
| rno-miR-370-3p | up | ENSRNOT00000010784 | ENSRNOG00000007993 | Sh3tc1 | SH3 domain and tetratricopeptide repeats 1 |  |
| rno-miR-370-3p | up | ENSRNOT00000019502 | ENSRNOG00000014243 | Pear1 | platelet endothelial aggregation receptor 1 |  |
| rno-miR-370-3p | up | ENSRNOT00000028445 | ENSRNOG00000020951 | Slc4a1 | solute carrier family 4 member 1 |  |
| rno-miR-370-3p | up | ENSRNOT00000030996 | ENSRNOG00000025185 | Nup188 | nucleoporin 188 ] |  |
| rno-miR-127-3p | up | ENSRNOT00000088459 | ENSRNOG00000011945 | Cyfip1 | cytoplasmic FMR1 interacting protein 1 |  |
| rno-miR-370-3p | up | ENSRNOT00000092102 | ENSRNOG00000020951 | Slc4a1 | solute carrier family 4 member 1 |  |
| rno-miR-673-3p | up | ENSRNOT00000023376 | ENSRNOG00000017299 | Phrf1 | PHD and ring finger domains 1 |  |
| rno-miR-665 | up | ENSRNOT00000039710 | ENSRNOG00000027837 | Gm14569 | predicted gene 14569 |  |
| rno-miR-665 | up | ENSRNOT00000083172 | ENSRNOG00000017593 | Mtr | 5-methyltetrahydrofolate-homocysteine methyltransferase |  |
| rno-miR-673-3p | up | ENSRNOT00000084147 | ENSRNOG00000060105 | Astn2 | astrotactin 2 |  |
| rno-miR-541-3p | up | ENSRNOT00000083429 | ENSRNOG00000023781 | Plec | plectin |  |
| rno-miR-370-3p | up | ENSRNOT00000012895 | ENSRNOG00000009318 | Ttll5 | tubulin tyrosine ligase like 5 |  |
| rno-miR-665 | up | ENSRNOT00000066480 | ENSRNOG00000003792 | Med14 | mediator complex subunit 14 |  |
| rno-miR-370-3p | up | ENSRNOT00000093145 | ENSRNOG00000025140 | Zfat | zinc finger and AT hook domain containing |  |
| rno-miR-370-3p | up | ENSRNOT00000064083 | ENSRNOG00000019122 | Sec16a | SEC16 homolog A, endoplasmic reticulum export factor |  |
| rno-miR-370-3p | up | ENSRNOT00000024824 | ENSRNOG00000018416 | Ttbk1 | tau tubulin kinase 1 |  |
| rno-miR-370-3p | up | ENSRNOT00000074347 | ENSRNOG00000050983 | Hpcal4 | hippocalcin-like 4 |  |
| rno-miR-140-3p | up | ENSRNOT00000045870 | ENSRNOG00000004464 | Sel1l | SEL1L ERAD E3 ligase adaptor subunit |  |
| rno-miR-370-3p | up | ENSRNOT00000001334 | ENSRNOG00000001004 | Ncor2 | nuclear receptor co-repressor 2 |  |
| rno-miR-132-3p | up | ENSRNOT00000011662 | ENSRNOG00000008614 | Zfyve1 | zinc finger FYVE-type containing 1 |  |
| rno-miR-370-3p | up | ENSRNOT00000055310 | ENSRNOG00000018225 | Tp53inp2 | tumor protein p53 inducible nuclear protein 2 |  |
| rno-miR-433-3p | up | ENSRNOT00000072399 | ENSRNOG00000048751 | Rtl1 | retrotransposon-like 1 |  |
| rno-miR-370-3p | up | ENSRNOT00000079324 | ENSRNOG00000019154 | Zmynd8 | zinc finger, MYND-type containing 8 |  |
| rno-miR-370-3p | up | ENSRNOT00000013378 | ENSRNOG00000009402 | Rabgap1 | RAB GTPase activating protein 1 |  |
| rno-miR-370-3p | up | ENSRNOT00000027132 | ENSRNOG00000020014 | Myh14 | myosin heavy chain 14 |  |
| rno-miR-370-3p | up | ENSRNOT00000080104 | ENSRNOG00000018416 | Ttbk1 | tau tubulin kinase 1 |  |
| rno-miR-370-3p | up | ENSRNOT00000090307 | ENSRNOG00000061038 | Myh15 | myosin, heavy chain 15 |  |
| rno-miR-370-3p | up | ENSRNOT00000086856 | ENSRNOG00000055531 | Saa4 | serum amyloid A4 |  |
| rno-miR-140-3p | up | ENSRNOT00000050734 | ENSRNOG00000024849 | Tor1aip2 | torsin 1A interacting protein 2 |  |
| rno-miR-370-3p | up | ENSRNOT00000044896 | ENSRNOG00000031801 | Ephb3 | Eph receptor B3 |  |
| rno-miR-370-3p | up | ENSRNOT00000080853 | ENSRNOG00000001126 | Fbxw8 | F-box and WD repeat domain containing 8 |  |
| rno-miR-370-3p | up | ENSRNOT00000011003 | ENSRNOG00000008129 | Pla2r1 | phospholipase A2 receptor 1 |  |
| rno-miR-673-3p | up | ENSRNOT00000051755 | ENSRNOG00000017299 | Phrf1 | PHD and ring finger domains 1 |  |
| rno-miR-212-5p | up | ENSRNOT00000064985 | ENSRNOG00000019964 | Taok2 | TAO kinase 2 |  |
| rno-miR-370-3p | up | ENSRNOT00000068291 | ENSRNOG00000025140 | Zfat | zinc finger and AT hook domain containing |  |
| rno-miR-665 | up | ENSRNOT00000093181 | ENSRNOG00000003792 | Med14 | mediator complex subunit 14 |  |
| rno-miR-665 | up | ENSRNOT00000039133 | ENSRNOG00000028274 | Myrf | myelin regulatory factor |  |
| rno-miR-214-3p | up | ENSRNOT00000006942 | ENSRNOG00000005003 | Ptprn2 | protein tyrosine phosphatase, receptor type N2 |  |
| rno-miR-214-3p | up | ENSRNOT00000044635 | ENSRNOG00000001888 | Arvcf | armadillo repeat gene deleted in velo-cardio-facial syndrome |  |
| rno-miR-127-5p | up | ENSRNOT00000043739 | ENSRNOG00000042030 | Nlrp4a | NLR family, pyrin domain containing 4A |  |
| rno-miR-370-3p | up | ENSRNOT00000081290 | ENSRNOG00000014395 | Gli3 | GLI family zinc finger 3 |  |
| rno-miR-370-3p | up | ENSRNOT00000005977 | ENSRNOG00000004430 | Cep131 | centrosomal protein 131 |  |
| rno-miR-370-3p | up | ENSRNOT00000077666 | ENSRNOG00000054515 | Fgd6 | FYVE, RhoGEF and PH domain containing 6 |  |
| rno-miR-370-3p | up | ENSRNOT00000012391 | ENSRNOG00000009253 | Igsf9b | immunoglobulin superfamily, member 9B |  |
| rno-miR-370-3p | up | ENSRNOT00000079250 | ENSRNOG00000060603 | Nhsl1 | NHS-like 1 |  |
| rno-miR-370-3p | up | ENSRNOT00000081621 | ENSRNOG00000015813 | Ubr2 | ubiquitin protein ligase E3 component n-recognin 2 |  |
| rno-miR-370-3p | up | ENSRNOT00000021158 | ENSRNOG00000015813 | Ubr2 | ubiquitin protein ligase E3 component n-recognin 2 |  |
| rno-miR-370-3p | up | ENSRNOT00000091336 | ENSRNOG00000015026 | Arhgef11 | Rho guanine nucleotide exchange factor 11 [Source:RGD Symbol;Acc:619705] |  |
| rno-miR-665 | up | ENSRNOT00000090932 | ENSRNOG00000060168 | Prkx | protein kinase, X-linked |  |
| rno-miR-370-3p | up | ENSRNOT00000012190 | ENSRNOG00000009033 | Cntn2 | contactin 2 |  |
| rno-miR-370-3p | up | ENSRNOT00000006345 | ENSRNOG00000024025 | Nrn1l | neuritin 1-like |  |
| rno-miR-127-3p | up | ENSRNOT00000072841 | ENSRNOG00000050240 | LOC100909856 | metal regulatory transcription factor 1-like |  |
| rno-miR-370-3p | up | ENSRNOT00000036321 | ENSRNOG00000026204 | Dclre1a | DNA cross-link repair 1A |  |
| rno-miR-370-3p | up | ENSRNOT00000008454 | ENSRNOG00000006412 | Zhx1 | zinc fingers and homeoboxes 1 |  |
| rno-miR-127-3p | up | ENSRNOT00000031928 | ENSRNOG00000025724 | Mtf1 | metal-regulatory transcription factor 1 |  |
| rno-miR-1193-3p | up | ENSRNOT00000011049 | ENSRNOG00000007982 | Slc9a1 | solute carrier family 9 member A1 |  |
| rno-miR-370-3p | up | ENSRNOT00000091760 | ENSRNOG00000020014 | Myh14 | myosin heavy chain 14 |  |
| rno-miR-370-3p | up | ENSRNOT00000027271 | ENSRNOG00000019977 | Ptprf | protein tyrosine phosphatase, receptor type, F |  |
| rno-miR-665 | up | ENSRNOT00000087025 | ENSRNOG00000060544 | Kdm5b | lysine demethylase 5B |  |
| rno-miR-665 | up | ENSRNOT00000023974 | ENSRNOG00000017593 | Mtr | 5-methyltetrahydrofolate-homocysteine methyltransferase |  |
| rno-miR-431 | up | ENSRNOT00000091129 | ENSRNOG00000024711 | Sdk2 | sidekick cell adhesion molecule 2 |  |
| rno-miR-6331 | up | ENSRNOT00000001464 | ENSRNOG00000001098 | Pds5b | PDS5 cohesin associated factor B |  |
| rno-miR-673-3p | up | ENSRNOT00000033491 | ENSRNOG00000026857 | Kif7 | kinesin family member 7 |  |
| rno-miR-665 | up | ENSRNOT00000064551 | ENSRNOG00000010652 | Dock6 | dedicator of cytokinesis 6 |  |
| rno-novel-58-mature | up | ENSRNOT00000010316 | ENSRNOG00000007713 | Tmcc3 | transmembrane and coiled-coil domain family 3 |  |
| rno-miR-132-5p | up | ENSRNOT00000002814 | ENSRNOG00000002053 | Fras1 | Fraser extracellular matrix complex subunit 1 |  |
| rno-miR-370-3p | up | ENSRNOT00000092606 | ENSRNOG00000002894 | Mnt | MAX network transcriptional repressor |  |
| rno-miR-370-3p | up | ENSRNOT00000079161 | ENSRNOG00000052062 | Zcchc11 | zinc finger CCHC-type containing 11 |  |
| rno-miR-370-3p | up | ENSRNOT00000007003 | ENSRNOG00000005263 | LOC688906 | similar to splicing factor, arginine/serine-rich 2, interacting protein |  |
| rno-miR-665 | up | ENSRNOT00000072261 | ENSRNOG00000050223 | Rin1 | Ras and Rab interactor 1 |  |
| rno-miR-665 | up | ENSRNOT00000093176 | ENSRNOG00000003792 | Med14 | mediator complex subunit 14 |  |
| rno-miR-485-5p | up | ENSRNOT00000036848 | ENSRNOG00000018412 | Sfi1 | SFI1 centrin binding protein |  |
| rno-miR-370-3p | up | ENSRNOT00000084390 | ENSRNOG00000061230 | L1cam | L1 cell adhesion molecule |  |
| rno-miR-665 | up | ENSRNOT00000048781 | ENSRNOG00000011927 | Sdc3 | syndecan 3 |  |
| rno-miR-140-3p | up | ENSRNOT00000025881 | ENSRNOG00000019014 | Ndst1 | N-deacetylase and N-sulfotransferase 1 |  |
| rno-miR-6331 | up | ENSRNOT00000050733 | ENSRNOG00000001098 | Pds5b | PDS5 cohesin associated factor B |  |
| rno-miR-370-3p | up | ENSRNOT00000078302 | ENSRNOG00000019977 | Ptprf | protein tyrosine phosphatase, receptor type, F |  |
| rno-miR-6331 | up | ENSRNOT00000064951 | ENSRNOG00000001098 | Pds5b | PDS5 cohesin associated factor B |  |
| rno-miR-370-3p | up | ENSRNOT00000050832 | ENSRNOG00000014030 | Synm | synemin [Source:RGD Symbol;Acc:727872] |  |
| rno-miR-127-5p | up | ENSRNOT00000023487 | ENSRNOG00000017410 | Loxhd1 | lipoxygenase homology domains 1 |  |
| rno-miR-665 | up | ENSRNOT00000090424 | ENSRNOG00000010652 | Dock6 | dedicator of cytokinesis 6 |  |
| rno-miR-370-3p | up | ENSRNOT00000023548 | ENSRNOG00000017516 | Bcl9 | B-cell CLL/lymphoma 9 |  |
| rno-miR-665 | up | ENSRNOT00000077928 | ENSRNOG00000060544 | Kdm5b | lysine demethylase 5B |  |
| rno-miR-485-5p | up | ENSRNOT00000060351 | ENSRNOG00000031773 | Cngb1 | cyclic nucleotide gated channel beta 1 |  |
| rno-miR-665 | up | ENSRNOT00000009839 | ENSRNOG00000006803 | Dnajc10 | DnaJ heat shock protein family (Hsp40) member C10 |  |
| rno-miR-214-3p | up | ENSRNOT00000020924 | ENSRNOG00000015585 | Suv39h2 | suppressor of variegation 3-9 homolog 2 |  |
| rno-miR-127-5p | up | ENSRNOT00000027897 | ENSRNOG00000020525 | Col5a3 | collagen type V alpha 3 chain |  |
| rno-miR-214-3p | up | ENSRNOT00000073235 | ENSRNOG00000048577 | Zfp955a | zinc finger protein 955A |  |
| rno-miR-665 | up | ENSRNOT00000030037 | ENSRNOG00000019555 | Arap1 | ArfGAP with RhoGAP domain, ankyrin repeat and PH domain 1 |  |
| rno-miR-485-5p | up | ENSRNOT00000075424 | ENSRNOG00000047046 | Plin4 | perilipin 4 |  |
| rno-miR-370-3p | up | ENSRNOT00000019396 | ENSRNOG00000014395 | Gli3 | GLI family zinc finger 3 |  |
| rno-miR-214-3p | up | ENSRNOT00000060432 | ENSRNOG00000019688 | Diaph1 | diaphanous-related formin 1 |  |
| rno-miR-31a-5p | up | ENSRNOT00000025683 | ENSRNOG00000018988 | Ing5 | inhibitor of growth family, member 5 |  |
| rno-miR-212-5p | up | ENSRNOT00000020929 | ENSRNOG00000015318 | Heyl | hes-related family bHLH transcription factor with YRPW motif-like |  |
| rno-miR-127-3p | up | ENSRNOT00000019867 | ENSRNOG00000014576 | Dock3 | dedicator of cyto-kinesis 3 |  |
| rno-miR-370-3p | up | ENSRNOT00000009173 | ENSRNOG00000006137 | Arid1a | AT-rich interaction domain 1A |  |
| rno-miR-665 | up | ENSRNOT00000045735 | ENSRNOG00000022343 | Alms1 | ALMS1, centrosome and basal body associated protein |  |
| rno-miR-370-3p | up | ENSRNOT00000030967 | ENSRNOG00000024025 | Nrn1l | neuritin 1-like |  |
| rno-miR-370-3p | up | ENSRNOT00000025878 | ENSRNOG00000018977 | Ap3d1 | adaptor-related protein complex 3, delta 1 subunit |  |
| rno-miR-665 | up | ENSRNOT00000072399 | ENSRNOG00000048751 | Rtl1 | retrotransposon-like 1 |  |
| rno-miR-370-3p | up | ENSRNOT00000018628 | ENSRNOG00000013917 | Igsf10 | immunoglobulin superfamily, member 10 |  |
| rno-miR-665 | up | ENSRNOT00000057461 | ENSRNOG00000023972 | Col4a2 | collagen type IV alpha 2 chain |  |
| rno-miR-665 | up | ENSRNOT00000086539 | ENSRNOG00000052887 | Dnajc6 | DnaJ heat shock protein family (Hsp40) member C6 |  |
| rno-miR-665 | up | ENSRNOT00000026643 | ENSRNOG00000019689 | Vwf | von Willebrand factor |  |
| rno-miR-370-3p | up | ENSRNOT00000047200 | ENSRNOG00000033110 | Svep1 | sushi, von Willebrand factor type A, EGF and pentraxin domain containing 1 ] |  |
| rno-miR-370-3p | up | ENSRNOT00000009963 | ENSRNOG00000007261 | Gli2 | GLI family zinc finger 2 |  |
| rno-miR-673-3p | up | ENSRNOT00000079999 | ENSRNOG00000047247 | Ptprs | protein tyrosine phosphatase, receptor type, S |  |
| rno-miR-673-3p | up | ENSRNOT00000074469 | ENSRNOG00000047247 | Ptprs | protein tyrosine phosphatase, receptor type, S |  |
| rno-miR-214-3p | up | ENSRNOT00000023974 | ENSRNOG00000017593 | Mtr | 5-methyltetrahydrofolate-homocysteine methyltransferase |  |
| rno-miR-370-3p | up | ENSRNOT00000056735 | ENSRNOG00000037483 | Ep400 | E1A binding protein p400 |  |
| rno-miR-370-3p | up | ENSRNOT00000084214 | ENSRNOG00000037483 | Ep400 | E1A binding protein p400 |  |
| rno-miR-673-3p | up | ENSRNOT00000071929 | ENSRNOG00000046327 | Rbpj | recombination signal binding protein for immunoglobulin kappa J region |  |
| rno-miR-665 | up | ENSRNOT00000002851 | ENSRNOG00000002080 | Urb1 | URB1 ribosome biogenesis 1 homolog (S. cerevisiae) |  |
| rno-miR-370-3p | up | ENSRNOT00000066480 | ENSRNOG00000003792 | Med14 | mediator complex subunit 14 |  |
| rno-miR-214-3p | up | ENSRNOT00000080788 | ENSRNOG00000009956 | Wnk1 | WNK lysine deficient protein kinase 1 |  |
| rno-miR-541-5p | up | ENSRNOT00000080108 | ENSRNOG00000061876 | Tas1r2 | taste 1 receptor member 2 |  |
| rno-miR-665 | up | ENSRNOT00000086259 | ENSRNOG00000052887 | Dnajc6 | DnaJ heat shock protein family (Hsp40) member C6 |  |
| rno-miR-214-3p | up | ENSRNOT00000043626 | ENSRNOG00000033893 | Cacna1h | calcium voltage-gated channel subunit alpha1 H |  |
| rno-miR-214-3p | up | ENSRNOT00000079513 | ENSRNOG00000009956 | Wnk1 | WNK lysine deficient protein kinase 1 |  |
| rno-miR-127-3p | up | ENSRNOT00000079383 | ENSRNOG00000053881 | Baz2a | bromodomain adjacent to zinc finger domain, 2A |  |
| rno-miR-370-3p | up | ENSRNOT00000030497 | ENSRNOG00000001448 | Hip1 | huntingtin interacting protein 1 |  |
| rno-miR-370-3p | up | ENSRNOT00000093181 | ENSRNOG00000003792 | Med14 | mediator complex subunit 14 |  |
| rno-miR-431 | up | ENSRNOT00000055137 | ENSRNOG00000006548 | Mrc2 | mannose receptor, C type 2 |  |
| rno-miR-370-3p | up | ENSRNOT00000086550 | ENSRNOG00000023781 | Plec | plectin |  |
| rno-miR-370-3p | up | ENSRNOT00000091285 | ENSRNOG00000023781 | Plec | plectin |  |
| rno-miR-665 | up | ENSRNOT00000023326 | ENSRNOG00000017208 | Cspg4 | chondroitin sulfate proteoglycan 4 |  |
| rno-miR-370-3p | up | ENSRNOT00000083248 | ENSRNOG00000016635 | LOC361646 | similar to K04F10.2 |  |
| rno-miR-665 | up | ENSRNOT00000048528 | ENSRNOG00000029260 | Pitpnm2 | phosphatidylinositol transfer protein, membrane-associated 2 |  |
| rno-miR-370-3p | up | ENSRNOT00000088945 | ENSRNOG00000023781 | Plec | plectin |  |
| rno-miR-673-3p | up | ENSRNOT00000032189 | ENSRNOG00000025502 | Arhgef37 | Rho guanine nucleotide exchange factor 37 |  |
| rno-miR-370-3p | up | ENSRNOT00000086186 | ENSRNOG00000013917 | Igsf10 | immunoglobulin superfamily, member 10 |  |
| rno-miR-370-3p | up | ENSRNOT00000015927 | ENSRNOG00000011460 | Arfgef3 | ARFGEF family member 3 |  |
| rno-miR-370-3p | up | ENSRNOT00000091840 | ENSRNOG00000023781 | Plec | plectin |  |
| rno-miR-127-5p | up | ENSRNOT00000085303 | ENSRNOG00000016833 | Ide | insulin degrading enzyme |  |
| rno-miR-665 | up | ENSRNOT00000047303 | ENSRNOG00000026985 | Phldb1 | pleckstrin homology-like domain, family B, member 1 |  |
| rno-miR-370-3p | up | ENSRNOT00000022365 | ENSRNOG00000016635 | LOC361646 | similar to K04F10.2 |  |
| rno-miR-370-3p | up | ENSRNOT00000042642 | ENSRNOG00000023781 | Plec | plectin |  |
| rno-miR-212-5p | up | ENSRNOT00000035770 | ENSRNOG00000012806 | Rbbp6 | RB binding protein 6, ubiquitin ligase |  |
| rno-miR-370-3p | up | ENSRNOT00000081021 | ENSRNOG00000023781 | Plec | plectin |  |
| rno-miR-370-3p | up | ENSRNOT00000012078 | ENSRNOG00000008932 | Ncapd3 | non-SMC condensin II complex, subunit D3 |  |
| rno-miR-370-3p | up | ENSRNOT00000082271 | ENSRNOG00000023781 | Plec | plectin |  |
| rno-miR-370-3p | up | ENSRNOT00000062017 | ENSRNOG00000040297 | Ttc37 | tetratricopeptide repeat domain 37 |  |
| rno-miR-665 | up | ENSRNOT00000057122 | ENSRNOG00000019129 | Fcgbp | Fc fragment of IgG binding protein |  |
| rno-miR-370-3p | up | ENSRNOT00000006311 | ENSRNOG00000023781 | Plec | plectin |  |
| rno-miR-204-5p | up | ENSRNOT00000044511 | ENSRNOG00000018877 | Zfp689 | zinc finger protein 689 |  |
| rno-miR-665 | up | ENSRNOT00000081850 | ENSRNOG00000060410 | Pcdh1 | protocadherin 1 |  |
| rno-miR-665 | up | ENSRNOT00000009960 | ENSRNOG00000007033 | Sorcs2 | sortilin-related VPS10 domain containing receptor 2 |  |
| rno-miR-673-3p | up | ENSRNOT00000031370 | ENSRNOG00000027066 | Stk32a | serine/threonine kinase 32A |  |
| rno-miR-431 | up | ENSRNOT00000072399 | ENSRNOG00000048751 | Rtl1 | retrotransposon-like 1 |  |
| rno-miR-665 | up | ENSRNOT00000088034 | ENSRNOG00000026985 | Phldb1 | pleckstrin homology-like domain, family B, member 1 |  |
| rno-miR-127-3p | up | ENSRNOT00000012373 | ENSRNOG00000009343 | Evpl | envoplakin |  |
| rno-miR-370-3p | up | ENSRNOT00000093176 | ENSRNOG00000003792 | Med14 | mediator complex subunit 14 |  |
| rno-miR-370-3p | up | ENSRNOT00000028633 | ENSRNOG00000021091 | Trank1 | tetratricopeptide repeat and ankyrin repeat containing 1 |  |
| rno-miR-370-3p | up | ENSRNOT00000024060 | ENSRNOG00000017767 | Mrvi1 | murine retrovirus integration site 1 homolog |  |
| rno-miR-665 | up | ENSRNOT00000010906 | ENSRNOG00000008145 | Traf3 | Tnf receptor-associated factor 3 |  |
| rno-miR-370-3p | up | ENSRNOT00000004834 | ENSRNOG00000003635 | Disp1 | dispatched RND transporter family member 1 |  |
| rno-miR-370-3p | up | ENSRNOT00000040762 | ENSRNOG00000023781 | Plec | plectin |  |
| rno-miR-665 | up | ENSRNOT00000051512 | ENSRNOG00000030515 | Nfasc | neurofascin |  |
| rno-miR-370-3p | up | ENSRNOT00000037389 | ENSRNOG00000025174 | Kat6a | lysine acetyltransferase 6A |  |
| rno-miR-433-3p | up | ENSRNOT00000021703 | ENSRNOG00000016046 | Hecw1 | HECT, C2 and WW domain containing E3 ubiquitin protein ligase 1 |  |
| rno-miR-370-3p | up | ENSRNOT00000025394 | ENSRNOG00000018434 | Stab1 | stabilin 1 |  |
| rno-miR-665 | up | ENSRNOT00000022063 | ENSRNOG00000016545 | Ift140 | intraflagellar transport 140 |  |
| rno-miR-665 | up | ENSRNOT00000040334 | ENSRNOG00000003848 | Med12 | mediator complex subunit 12 |  |
| rno-miR-214-3p | up | ENSRNOT00000018654 | ENSRNOG00000013669 | Pik3r4 | phosphoinositide-3-kinase, regulatory subunit 4 |  |
| rno-miR-370-3p | up | ENSRNOT00000085698 | ENSRNOG00000033110 | Svep1 | sushi, von Willebrand factor type A, EGF and pentraxin domain containing 1 |  |
| rno-miR-665 | up | ENSRNOT00000089334 | ENSRNOG00000007033 | Sorcs2 | sortilin-related VPS10 domain containing receptor 2 |  |
| rno-novel-58-mature | up | ENSRNOT00000088095 | ENSRNOG00000019885 | Magi3 | membrane associated guanylate kinase, WW and PDZ domain containing 3 |  |
| rno-novel-58-mature | up | ENSRNOT00000026952 | ENSRNOG00000019885 | Magi3 | membrane associated guanylate kinase, WW and PDZ domain containing 3 |  |
| rno-miR-665 | up | ENSRNOT00000090192 | ENSRNOG00000003848 | Med12 | mediator complex subunit 12 |  |
| rno-miR-665 | up | ENSRNOT00000044972 | ENSRNOG00000030515 | Nfasc | neurofascin |  |
| rno-novel-58-mature | up | ENSRNOT00000083220 | ENSRNOG00000054080 | Cgnl1 | cingulin-like 1 |  |
| rno-miR-370-3p | up | ENSRNOT00000007776 | ENSRNOG00000022015 | Cntrl | centriolin |  |
| rno-miR-370-3p | up | ENSRNOT00000078913 | ENSRNOG00000020297 | Gon4l | gon-4 like |  |
| rno-miR-370-3p | up | ENSRNOT00000064774 | ENSRNOG00000005391 | Prex2 | phosphatidylinositol-3,4,5-trisphosphate-dependent Rac exchange factor 2 |  |
| rno-miR-370-3p | up | ENSRNOT00000021837 | ENSRNOG00000016067 | Ckap5 | cytoskeleton associated protein 5 |  |
| rno-miR-665 | up | ENSRNOT00000060871 | ENSRNOG00000030515 | Nfasc | neurofascin |  |
| rno-miR-122-5p | up | ENSRNOT00000018931 | ENSRNOG00000013938 | Tshz3 | teashirt zinc finger homeobox 3 |  |
| rno-miR-214-3p | up | ENSRNOT00000055875 | ENSRNOG00000016965 | Anapc1 | anaphase promoting complex subunit 1 |  |
| rno-miR-665 | up | ENSRNOT00000042865 | ENSRNOG00000031643 | Dchs1 | dachsous cadherin-related 1 |  |
| rno-miR-665 | up | ENSRNOT00000088568 | ENSRNOG00000014375 | Adgrb2 | adhesion G protein-coupled receptor B2 |  |
| rno-miR-665 | up | ENSRNOT00000093710 | ENSRNOG00000060984 | Dnah7 | dynein, axonemal, heavy chain 7 |  |
| rno-miR-673-3p | up | ENSRNOT00000080751 | ENSRNOG00000009615 | Mtor | mechanistic target of rapamycin |  |
| rno-miR-370-3p | up | ENSRNOT00000064041 | ENSRNOG00000012420 | Bcl9l | B-cell CLL/lymphoma 9-like |  |
| rno-miR-665 | up | ENSRNOT00000076957 | ENSRNOG00000003848 | Med12 | mediator complex subunit 12 |  |
| rno-miR-665 | up | ENSRNOT00000032719 | ENSRNOG00000022720 | Usp37 | ubiquitin specific peptidase 37 |  |
| rno-miR-665 | up | ENSRNOT00000064948 | ENSRNOG00000014375 | Adgrb2 | adhesion G protein-coupled receptor B2 |  |
| rno-miR-673-3p | up | ENSRNOT00000014167 | ENSRNOG00000009615 | Mtor | mechanistic target of rapamycin |  |
| rno-miR-127-5p | up | ENSRNOT00000090307 | ENSRNOG00000061038 | Myh15 | myosin, heavy chain 15 |  |
| rno-miR-665 | up | ENSRNOT00000082992 | ENSRNOG00000010663 | Col6a5 | collagen type VI alpha 5 chain |  |
| rno-miR-665 | up | ENSRNOT00000038160 | ENSRNOG00000010663 | Col6a5 | collagen type VI alpha 5 chain |  |
| rno-miR-370-3p | up | ENSRNOT00000083429 | ENSRNOG00000023781 | Plec | plectin |  |
| rno-miR-370-3p | up | ENSRNOT00000092745 | ENSRNOG00000022015 | Cntrl | centriolin |  |
| rno-miR-370-3p | up | ENSRNOT00000061873 | ENSRNOG00000023053 | Ice1 | interactor of little elongation complex ELL subunit 1 |  |
| rno-miR-370-3p | up | ENSRNOT00000077698 | ENSRNOG00000020297 | Gon4l | gon-4 like |  |
| rno-miR-370-3p | up | ENSRNOT00000017787 | ENSRNOG00000013267 | Helz2 | helicase with zinc finger 2, transcriptional coactivator |  |
| rno-miR-370-3p | up | ENSRNOT00000056789 | ENSRNOG00000020297 | Gon4l | gon-4 like |  |
| rno-miR-370-3p | up | ENSRNOT00000017892 | ENSRNOG00000013053 | Trpm6 | transient receptor potential cation channel, subfamily M, member 6 |  |
| rno-miR-370-3p | up | ENSRNOT00000054685 | ENSRNOG00000014705 | Rbm20 | RNA binding motif protein 20 |  |
| rno-miR-370-3p | up | ENSRNOT00000004716 | ENSRNOG00000003537 | Spta1 | spectrin, alpha, erythrocytic 1 |  |
| rno-miR-370-3p | up | ENSRNOT00000066509 | ENSRNOG00000003537 | Spta1 | spectrin, alpha, erythrocytic 1 |  |
| rno-miR-665 | up | ENSRNOT00000035906 | ENSRNOG00000025848 | Sspo | SCO-spondin |  |
| rno-miR-665 | up | ENSRNOT00000086033 | ENSRNOG00000018461 | Pdgfrb | platelet derived growth factor receptor beta |  |
| rno-miR-370-3p | up | ENSRNOT00000067649 | ENSRNOG00000026415 | Col14a1 | collagen type XIV alpha 1 chain |  |
| rno-miR-665 | up | ENSRNOT00000068535 | ENSRNOG00000018461 | Pdgfrb | platelet derived growth factor receptor beta |  |
| rno-miR-370-3p | up | ENSRNOT00000022742 | ENSRNOG00000016483 | Myo16 | myosin XVI |  |
| rno-miR-370-3p | up | ENSRNOT00000055888 | ENSRNOG00000037085 | Xirp1 | xin actin-binding repeat containing 1 |  |
| rno-miR-370-3p | up | ENSRNOT00000055829 | ENSRNOG00000012716 | Chd2 | chromodomain helicase DNA binding protein 2 |  |
| rno-miR-485-5p | up | ENSRNOT00000017636 | ENSRNOG00000013017 | Arnt2 | aryl hydrocarbon receptor nuclear translocator 2 |  |
| rno-miR-370-3p | up | ENSRNOT00000068594 | ENSRNOG00000014452 | Zfhx3 | zinc finger homeobox 3 |  |
| rno-miR-370-3p | up | ENSRNOT00000026414 | ENSRNOG00000019414 | Tmem79 | transmembrane protein 79 |  |
| rno-miR-370-3p | up | ENSRNOT00000019408 | ENSRNOG00000014452 | Zfhx3 | zinc finger homeobox 3 |  |
| rno-miR-300-3p | up | ENSRNOT00000051846 | ENSRNOG00000007090 | Cacna1c | calcium voltage-gated channel subunit alpha1 C |  |
| rno-miR-127-5p | up | ENSRNOT00000038017 | ENSRNOG00000016714 | Nrap | nebulin-related anchoring protein |  |
| rno-miR-127-5p | up | ENSRNOT00000030511 | ENSRNOG00000011411 | Adgrg6 | adhesion G protein-coupled receptor G6 |  |
| rno-miR-370-3p | up | ENSRNOT00000078743 | ENSRNOG00000025174 | Kat6a | lysine acetyltransferase 6A |  |
| rno-miR-665 | up | ENSRNOT00000064705 | ENSRNOG00000011969 | Dock9 | dedicator of cytokinesis 9 |  |
| rno-miR-673-3p | up | ENSRNOT00000073991 | ENSRNOG00000047247 | Ptprs | protein tyrosine phosphatase, receptor type, S |  |
| rno-miR-300-3p | up | ENSRNOT00000052017 | ENSRNOG00000007090 | Cacna1c | calcium voltage-gated channel subunit alpha1 C |  |
| rno-miR-299a-3p | up | ENSRNOT00000085291 | ENSRNOG00000016443 | Vps13d | vacuolar protein sorting 13D |  |
| rno-miR-214-3p | up | ENSRNOT00000055137 | ENSRNOG00000006548 | Mrc2 | mannose receptor, C type 2 |  |
| rno-miR-212-5p | up | ENSRNOT00000082100 | ENSRNOG00000052157 | Nav3 | neuron navigator 3 |  |
| rno-miR-299a-3p | up | ENSRNOT00000022185 | ENSRNOG00000016443 | Vps13d | vacuolar protein sorting 13D |  |
| rno-miR-665 | up | ENSRNOT00000090572 | ENSRNOG00000011969 | Dock9 | dedicator of cytokinesis 9 |  |
| rno-miR-370-3p | up | ENSRNOT00000090149 | ENSRNOG00000012716 | Chd2 | chromodomain helicase DNA binding protein 2 |  |
| rno-miR-665 | up | ENSRNOT00000021353 | ENSRNOG00000015285 | Lrp4 | LDL receptor related protein 4 |  |
| rno-miR-127-5p | up | ENSRNOT00000038038 | ENSRNOG00000016714 | Nrap | nebulin-related anchoring protein |  |
| rno-miR-300-3p | up | ENSRNOT00000041571 | ENSRNOG00000007090 | Cacna1c | calcium voltage-gated channel subunit alpha1 C |  |
| rno-miR-214-3p | up | ENSRNOT00000047462 | ENSRNOG00000030597 | Ankrd52 | ankyrin repeat domain 52 |  |
| rno-miR-370-3p | up | ENSRNOT00000090519 | ENSRNOG00000015852 | Arhgap35 | Rho GTPase activating protein 35 |  |
| rno-miR-214-3p | up | ENSRNOT00000014832 | ENSRNOG00000010852 | Nup205 | nucleoporin 205 |  |
| rno-miR-370-3p | up | ENSRNOT00000007079 | ENSRNOG00000005330 | Crebbp | CREB binding protein |  |
| rno-miR-433-3p | up | ENSRNOT00000015092 | ENSRNOG00000010771 | Pkd1 | polycystin 1, transient receptor potential channel interacting |  |
| rno-miR-370-3p | up | ENSRNOT00000091816 | ENSRNOG00000016632 | Dsg3 | desmoglein 3 |  |
| rno-miR-665 | up | ENSRNOT00000081445 | ENSRNOG00000001272 | Mcm3ap | minichromosome maintenance complex component 3 associated protein |  |
| rno-miR-665 | up | ENSRNOT00000009028 | ENSRNOG00000006911 | Sptb | spectrin, beta, erythrocytic |  |
| rno-miR-214-3p | up | ENSRNOT00000013621 | ENSRNOG00000009956 | Wnk1 | WNK lysine deficient protein kinase 1 |  |
| rno-miR-214-3p | up | ENSRNOT00000013355 | ENSRNOG00000009956 | Wnk1 | WNK lysine deficient protein kinase 1 |  |
| rno-miR-370-3p | up | ENSRNOT00000076300 | ENSRNOG00000012716 | Chd2 | chromodomain helicase DNA binding protein 2 |  |
| rno-miR-370-3p | up | ENSRNOT00000026843 | ENSRNOG00000019791 | Sipa1l2 | signal-induced proliferation-associated 1 like 2 |  |
| rno-miR-370-3p | up | ENSRNOT00000056161 | ENSRNOG00000020748 | Map4 | microtubule-associated protein 4 |  |
| rno-miR-370-3p | up | ENSRNOT00000046381 | ENSRNOG00000025155 | Lmtk2 | lemur tyrosine kinase 2 |  |
| rno-miR-214-3p | up | ENSRNOT00000079133 | ENSRNOG00000059219 | Myo15a | myosin XVA |  |
| rno-miR-370-3p | up | ENSRNOT00000065201 | ENSRNOG00000043357 | Zfp407 | zinc finger protein 407 |  |
| rno-miR-665 | up | ENSRNOT00000017878 | ENSRNOG00000012892 | Abca4 | ATP binding cassette subfamily A member 4 |  |
| rno-miR-127-3p | up | ENSRNOT00000048218 | ENSRNOG00000001276 | Pcnt | pericentrin |  |
| rno-miR-214-3p | up | ENSRNOT00000086083 | ENSRNOG00000059219 | Myo15a | myosin XVA |  |
| rno-miR-665 | up | ENSRNOT00000013189 | ENSRNOG00000009936 | Dido1 | death inducer-obliterator 1 |  |
| rno-miR-127-3p | up | ENSRNOT00000088138 | ENSRNOG00000034013 | Acaca | acetyl-CoA carboxylase alpha |  |
| rno-miR-665 | up | ENSRNOT00000028633 | ENSRNOG00000021091 | Trank1 | tetratricopeptide repeat and ankyrin repeat containing 1 |  |
| rno-miR-214-3p | up | ENSRNOT00000077546 | ENSRNOG00000059260 | AABR07053509.2 |  |  |
| rno-miR-1193-3p | up | ENSRNOT00000007776 | ENSRNOG00000022015 | Cntrl | centriolin |  |
| rno-miR-665 | up | ENSRNOT00000064536 | ENSRNOG00000032788 | Dysf | dysferlin |  |
| rno-miR-665 | up | ENSRNOT00000087990 | ENSRNOG00000052572 | Aff2 | AF4/FMR2 family, member 2 |  |
| rno-miR-673-3p | up | ENSRNOT00000003837 | ENSRNOG00000002841 | Cdc42bpa | CDC42 binding protein kinase alpha |  |
| rno-miR-1193-3p | up | ENSRNOT00000092745 | ENSRNOG00000022015 | Cntrl | centriolin |  |
| rno-miR-140-3p | up | ENSRNOT00000006319 | ENSRNOG00000004645 | Galnt5 | polypeptide N-acetylgalactosaminyltransferase 5 |  |
| rno-miR-665 | up | ENSRNOT00000037456 | ENSRNOG00000004346 | Notch3 | notch 3 |  |
| rno-miR-370-3p | up | ENSRNOT00000024443 | ENSRNOG00000017940 | Rere | arginine-glutamic acid dipeptide repeats |  |
| rno-miR-665 | up | ENSRNOT00000085765 | ENSRNOG00000032788 | Dysf | dysferlin |  |
| rno-miR-665 | up | ENSRNOT00000087617 | ENSRNOG00000052572 | Aff2 | AF4/FMR2 family, member 2 |  |
| rno-miR-665 | up | ENSRNOT00000066548 | ENSRNOG00000043219 | Fbn2 | fibrillin 2 |  |
| rno-miR-127-5p | up | ENSRNOT00000066548 | ENSRNOG00000043219 | Fbn2 | fibrillin 2 |  |
| rno-miR-370-3p | up | ENSRNOT00000048218 | ENSRNOG00000001276 | Pcnt | pericentrin |  |
| rno-miR-665 | up | ENSRNOT00000008459 | ENSRNOG00000006905 | Hectd1 | HECT domain E3 ubiquitin protein ligase 1 |  |
| rno-miR-673-3p | up | ENSRNOT00000004035 | ENSRNOG00000003028 | Dnah17 | dynein, axonemal, heavy chain 17 |  |
| rno-miR-665 | up | ENSRNOT00000021840 | ENSRNOG00000014997 | Igf2r | insulin-like growth factor 2 receptor |  |
| rno-miR-370-3p | up | ENSRNOT00000016784 | ENSRNOG00000012148 | Trio | trio Rho guanine nucleotide exchange factor |  |
| rno-miR-370-3p | up | ENSRNOT00000026212 | ENSRNOG00000019322 | Notch1 | notch 1 |  |
| rno-miR-214-3p | up | ENSRNOT00000088188 | ENSRNOG00000023453 | Lrba | LPS responsive beige-like anchor protein |  |
| rno-miR-214-3p | up | ENSRNOT00000088807 | ENSRNOG00000023453 | Lrba | LPS responsive beige-like anchor protein |  |
| rno-miR-485-5p | up | ENSRNOT00000077382 | ENSRNOG00000006104 | Tg | thyroglobulin |  |
| rno-miR-665 | up | ENSRNOT00000082113 | ENSRNOG00000030714 | Bsn | bassoon (presynaptic cytomatrix protein) |  |
| rno-miR-665 | up | ENSRNOT00000092162 | ENSRNOG00000060984 | Dnah7 | dynein, axonemal, heavy chain 7 |  |
| rno-miR-485-5p | up | ENSRNOT00000009240 | ENSRNOG00000006104 | Tg | thyroglobulin |  |
| rno-miR-665 | up | ENSRNOT00000086078 | ENSRNOG00000022893 | Rimbp2 | RIMS binding protein 2 |  |
| rno-miR-370-3p | up | ENSRNOT00000039235 | ENSRNOG00000028659 | Szt2 | seizure threshold 2 homolog (mouse) |  |
| rno-miR-665 | up | ENSRNOT00000091287 | ENSRNOG00000058561 | Srrm2 | serine/arginine repetitive matrix 2 |  |
| rno-miR-31a-5p | up | ENSRNOT00000049146 | ENSRNOG00000033791 | Apc2 | APC2, WNT signaling pathway regulator |  |
| rno-miR-665 | up | ENSRNOT00000079215 | ENSRNOG00000059865 | Dnah12 | dynein, axonemal, heavy chain 12 |  |
| rno-miR-665 | up | ENSRNOT00000020558 | ENSRNOG00000014901 | Uggt1 | UDP-glucose glycoprotein glucosyltransferase 1 |  |
| rno-miR-665 | up | ENSRNOT00000043811 | ENSRNOG00000029662 | Wdfy4 | WDFY family member 4 |  |
| rno-miR-341 | up | ENSRNOT00000039631 | ENSRNOG00000028545 | Ahnak2 | AHNAK nucleoprotein 2 |  |
| rno-miR-673-3p | up | ENSRNOT00000004047 | ENSRNOG00000003028 | Dnah17 | dynein, axonemal, heavy chain 17 |  |
| rno-miR-370-3p | up | ENSRNOT00000008637 | ENSRNOG00000005726 | Pclo | piccolo (presynaptic cytomatrix protein) |  |
| rno-miR-127-5p | up | ENSRNOT00000042865 | ENSRNOG00000031643 | Dchs1 | dachsous cadherin-related 1 |  |
| rno-miR-485-5p | up | ENSRNOT00000076776 | ENSRNOG00000028627 | Hmcn1 | hemicentin 1 |  |
| rno-miR-370-3p | up | ENSRNOT00000007608 | ENSRNOG00000005726 | Pclo | piccolo (presynaptic cytomatrix protein) |  |
| rno-miR-665 | up | ENSRNOT00000042528 | ENSRNOG00000030714 | Bsn | bassoon (presynaptic cytomatrix protein) |  |
| rno-miR-665 | up | ENSRNOT00000081667 | ENSRNOG00000051291 | Dnhd1 | dynein heavy chain domain 1 |  |
| rno-miR-485-5p | up | ENSRNOT00000030971 | ENSRNOG00000028627 | Hmcn1 | hemicentin 1 |  |
| rno-miR-665 | up | ENSRNOT00000076783 | ENSRNOG00000051291 | Dnhd1 | dynein heavy chain domain 1 |  |
| rno-miR-341 | up | ENSRNOT00000031005 | ENSRNOG00000025053 | Lrp1 | LDL receptor related protein 1 |  |
| rno-miR-370-3p | up | ENSRNOT00000077502 | ENSRNOG00000061499 | Kmt2d | lysine methyltransferase 2D |  |
| rno-miR-665 | up | ENSRNOT00000086550 | ENSRNOG00000023781 | Plec | plectin |  |
| rno-miR-665 | up | ENSRNOT00000091285 | ENSRNOG00000023781 | Plec | plectin |  |
| rno-miR-665 | up | ENSRNOT00000088945 | ENSRNOG00000023781 | Plec | plectin |  |
| rno-miR-665 | up | ENSRNOT00000091840 | ENSRNOG00000023781 | Plec | plectin |  |
| rno-miR-665 | up | ENSRNOT00000042642 | ENSRNOG00000023781 | Plec | plectin |  |
| rno-miR-665 | up | ENSRNOT00000081021 | ENSRNOG00000023781 | Plec | plectin |  |
| rno-miR-665 | up | ENSRNOT00000082271 | ENSRNOG00000023781 | Plec | plectin |  |
| rno-miR-665 | up | ENSRNOT00000006311 | ENSRNOG00000023781 | Plec | plectin |  |
| rno-miR-665 | up | ENSRNOT00000040762 | ENSRNOG00000023781 | Plec | plectin |  |
| rno-miR-665 | up | ENSRNOT00000079109 | ENSRNOG00000010050 | Stard9 | StAR-related lipid transfer domain containing 9 |  |
| rno-miR-665 | up | ENSRNOT00000082138 | ENSRNOG00000018183 | Ubr4 | ubiquitin protein ligase E3 component n-recognin 4 |  |
| rno-miR-665 | up | ENSRNOT00000039663 | ENSRNOG00000018183 | Ubr4 | ubiquitin protein ligase E3 component n-recognin 4 |  |
| rno-miR-127-3p | up | ENSRNOT00000035906 | ENSRNOG00000025848 | Sspo | SCO-spondin |  |
| rno-miR-140-3p | up | ENSRNOT00000039631 | ENSRNOG00000028545 | Ahnak2 | AHNAK nucleoprotein 2 |  |
| rno-miR-665 | up | ENSRNOT00000044452 | ENSRNOG00000012207 | Dst | dystonin |  |
| rno-miR-370-3p | up | ENSRNOT00000044452 | ENSRNOG00000012207 | Dst | dystonin |  |
| rno-miR-370-3p | up | ENSRNOT00000014158 | ENSRNOG00000010653 | Elmsan1 | ELM2 and Myb/SANT domain containing 1 |  |
| rno-miR-665 | up | ENSRNOT00000085547 | ENSRNOG00000000463 | Col11a2 | collagen type XI alpha 2 chain |  |
| rno-miR-665 | up | ENSRNOT00000018116 | ENSRNOG00000012920 | Col9a1 | collagen type IX alpha 1 chain |  |
| rno-miR-665 | up | ENSRNOT00000033047 | ENSRNOG00000025997 | Mrrf | mitochondrial ribosome recycling factor |  |
| rno-miR-665 | up | ENSRNOT00000083429 | ENSRNOG00000023781 | Plec | plectin |  |
| rno-miR-665 | up | ENSRNOT00000029511 | ENSRNOG00000025705 | Armcx2 | armadillo repeat containing, X-linked 2 |  |
| rno-miR-665 | up | ENSRNOT00000004510 | ENSRNOG00000046785 | Col23a1 | collagen type XXIII alpha 1 chain |  |
| rno-miR-370-3p | up | ENSRNOT00000027493 | ENSRNOG00000020272 | RGD1310209 | similar to KIAA1324 protein |  |
| rno-miR-370-3p | up | ENSRNOT00000042528 | ENSRNOG00000030714 | Bsn | bassoon (presynaptic cytomatrix protein) |  |
| rno-miR-665 | up | ENSRNOT00000004956 | ENSRNOG00000003357 | Col3a1 | collagen type III alpha 1 chain |  |
| rno-miR-665 | up | ENSRNOT00000045533 | ENSRNOG00000000463 | Col11a2 | collagen type XI alpha 2 chain |  |
| rno-miR-370-3p | up | ENSRNOT00000029510 | ENSRNOG00000028526 | Mansc1 | MANSC domain containing 1 |  |
| rno-miR-665 | up | ENSRNOT00000090390 | ENSRNOG00000015035 | Myo7b | myosin VIIb |  |
| rno-miR-370-3p | up | ENSRNOT00000026102 | ENSRNOG00000019268 | Pelp1 | proline, glutamate and leucine rich protein 1 |  |
| rno-miR-370-3p | up | ENSRNOT00000000474 | ENSRNOG00000000417 | Numa1 | nuclear mitotic apparatus protein 1 |  |
| rno-miR-665 | up | ENSRNOT00000084117 | ENSRNOG00000000463 | Col11a2 | collagen type XI alpha 2 chain |  |
| rno-miR-665 | up | ENSRNOT00000001905 | ENSRNOG00000001407 | Tfr2 | transferrin receptor 2 |  |
| rno-miR-665 | up | ENSRNOT00000082895 | ENSRNOG00000001407 | Tfr2 | transferrin receptor 2 |  |
| rno-miR-370-3p | up | ENSRNOT00000083976 | ENSRNOG00000001101 | Rnf216 | ring finger protein 216 |  |
| rno-miR-665 | up | ENSRNOT00000027897 | ENSRNOG00000020525 | Col5a3 | collagen type V alpha 3 chain |  |
| rno-miR-370-3p | up | ENSRNOT00000078816 | ENSRNOG00000026985 | Phldb1 | pleckstrin homology-like domain, family B, member 1 |  |
| rno-miR-370-3p | up | ENSRNOT00000055249 | ENSRNOG00000000417 | Numa1 | nuclear mitotic apparatus protein 1 |  |
| rno-miR-665 | up | ENSRNOT00000000203 | ENSRNOG00000000187 | Csf2rb | colony stimulating factor 2 receptor beta common subunit |  |
| rno-miR-665 | up | ENSRNOT00000014388 | ENSRNOG00000010841 | Col8a2 | collagen type VIII alpha 2 chain |  |
| rno-miR-370-3p | up | ENSRNOT00000001458 | ENSRNOG00000001101 | Rnf216 | ring finger protein 216 |  |
| rno-miR-665 | up | ENSRNOT00000017217 | ENSRNOG00000039197 | LOC108348074 | collagen alpha-1(XV) chain-like |  |
| rno-miR-370-3p | up | ENSRNOT00000042421 | ENSRNOG00000032693 | LOC498155 | LRRGT00167 |  |
| rno-miR-370-3p | up | ENSRNOT00000082216 | ENSRNOG00000004650 | Begain | brain-enriched guanylate kinase-associated |  |
| rno-miR-665 | up | ENSRNOT00000086830 | ENSRNOG00000061348 | Fam53b | family with sequence similarity 53, member B |  |
| rno-miR-370-3p | up | ENSRNOT00000041903 | ENSRNOG00000004650 | Begain | brain-enriched guanylate kinase-associated |  |
| rno-miR-370-3p | up | ENSRNOT00000040203 | ENSRNOG00000002879 | Psen2 | presenilin 2 |  |
| rno-miR-665 | up | ENSRNOT00000086062 | ENSRNOG00000058560 | Col2a1 | collagen type II alpha 1 chain |  |
| rno-miR-370-3p | up | ENSRNOT00000061404 | ENSRNOG00000011720 | L3mbtl3 | l(3)mbt-like 3 (Drosophila) |  |
| rno-miR-370-3p | up | ENSRNOT00000023490 | ENSRNOG00000017198 | Hif3a | hypoxia inducible factor 3, alpha subunit |  |
| rno-miR-127-3p | up | ENSRNOT00000065791 | ENSRNOG00000020420 | Pklr | pyruvate kinase, liver and RBC |  |
| rno-miR-665 | up | ENSRNOT00000088385 | ENSRNOG00000060381 | Col15a1 | collagen type XV alpha 1 chain |  |
| rno-miR-370-3p | up | ENSRNOT00000090143 | ENSRNOG00000020457 | Tacc2 | transforming, acidic coiled-coil containing protein 2 |  |
| rno-miR-665 | up | ENSRNOT00000068413 | ENSRNOG00000023148 | Col11a1 | collagen type XI alpha 1 chain |  |
| rno-miR-665 | up | ENSRNOT00000015092 | ENSRNOG00000010771 | Pkd1 | polycystin 1, transient receptor potential channel interacting |  |
| rno-miR-665 | up | ENSRNOT00000077503 | ENSRNOG00000014851 | Col4a4 | collagen type IV alpha 4 chain |  |
| rno-miR-665 | up | ENSRNOT00000064478 | ENSRNOG00000018951 | Col4a5 | collagen type IV alpha 5 chain |  |
| rno-miR-370-3p | up | ENSRNOT00000068434 | ENSRNOG00000010983 | Otog | otogelin |  |
| rno-miR-370-3p | up | ENSRNOT00000047161 | ENSRNOG00000020457 | Tacc2 | transforming, acidic coiled-coil containing protein 2 |  |
| rno-miR-370-3p | up | ENSRNOT00000010786 | ENSRNOG00000007710 | Usp20 | ubiquitin specific peptidase 20 |  |
| rno-miR-370-3p | up | ENSRNOT00000085614 | ENSRNOG00000017422 | Ambra1 | autophagy and beclin 1 regulator 1 |  |
| rno-miR-665 | up | ENSRNOT00000012644 | ENSRNOG00000009450 | Hcn4 | hyperpolarization activated cyclic nucleotide-gated potassium channel 4 |  |
| rno-miR-6331 | up | ENSRNOT00000039631 | ENSRNOG00000028545 | Ahnak2 | AHNAK nucleoprotein 2 |  |
| rno-miR-665 | up | ENSRNOT00000010932 | ENSRNOG00000008246 | Emilin1 | elastin microfibril interfacer 1 |  |
| rno-miR-370-3p | up | ENSRNOT00000059486 | ENSRNOG00000001424 | Cux1 | cut-like homeobox 1 |  |
| rno-miR-370-3p | up | ENSRNOT00000007124 | ENSRNOG00000005379 | Prp2 | proline rich protein 2 |  |
| rno-miR-665 | up | ENSRNOT00000087619 | ENSRNOG00000052925 | NEWGENE_621351 | collagen, type I, alpha 2 |  |
| rno-miR-370-3p | up | ENSRNOT00000047303 | ENSRNOG00000026985 | Phldb1 | pleckstrin homology-like domain, family B, member 1 |  |
| rno-miR-370-3p | up | ENSRNOT00000088034 | ENSRNOG00000026985 | Phldb1 | pleckstrin homology-like domain, family B, member 1 |  |
| rno-miR-370-3p | up | ENSRNOT00000045644 | ENSRNOG00000017422 | Ambra1 | autophagy and beclin 1 regulator 1 |  |
| rno-miR-665 | up | ENSRNOT00000089564 | ENSRNOG00000052925 | NEWGENE_621351 | collagen, type I, alpha 2 |  |
| rno-miR-665 | up | ENSRNOT00000089292 | ENSRNOG00000011292 | NEWGENE_621351 | collagen, type I, alpha 2 |  |
| rno-miR-665 | up | ENSRNOT00000016423 | ENSRNOG00000011292 | NEWGENE_621351 | collagen, type I, alpha 2 |  |
| rno-miR-370-3p | up | ENSRNOT00000006970 | ENSRNOG00000004831 | Arid2 | AT-rich interaction domain 2 |  |
| rno-miR-665 | up | ENSRNOT00000005311 | ENSRNOG00000003897 | Col1a1 | collagen type I alpha 1 chain |  |
| rno-miR-6331 | up | ENSRNOT00000089171 | ENSRNOG00000020369 | Igf2 | insulin-like growth factor 2 |  |
| rno-miR-665 | up | ENSRNOT00000047563 | ENSRNOG00000031782 | Col6a4 | collagen, type VI, alpha 4 |  |
| rno-miR-665 | up | ENSRNOT00000010333 | ENSRNOG00000007657 | Col27a1 | collagen type XXVII alpha 1 chain |  |
| rno-miR-665 | up | ENSRNOT00000056145 | ENSRNOG00000020862 | Ptpn23 | protein tyrosine phosphatase, non-receptor type 23 |  |
| rno-miR-665 | up | ENSRNOT00000010516 | ENSRNOG00000008005 | Akna | AT-hook transcription factor |  |
| rno-miR-370-3p | up | ENSRNOT00000092024 | ENSRNOG00000060528 | Cacna1g | calcium voltage-gated channel subunit alpha1 G |  |
| rno-miR-370-3p | up | ENSRNOT00000033123 | ENSRNOG00000025198 | Gas2l3 | growth arrest-specific 2 like 3 |  |
| rno-miR-370-3p | up | ENSRNOT00000088323 | ENSRNOG00000054757 | Adcy6 | adenylate cyclase 6 |  |
| rno-miR-370-3p | up | ENSRNOT00000082699 | ENSRNOG00000054757 | Adcy6 | adenylate cyclase 6 |  |
| rno-miR-665 | up | ENSRNOT00000035285 | ENSRNOG00000022244 | Olr1462 | olfactory receptor 1462 |  |
| rno-miR-370-3p | up | ENSRNOT00000081307 | ENSRNOG00000060528 | Cacna1g | calcium voltage-gated channel subunit alpha1 G |  |
| rno-miR-665 | up | ENSRNOT00000079096 | ENSRNOG00000028426 | Mcf2l | MCF.2 cell line derived transforming sequence-like |  |
| rno-miR-370-3p | up | ENSRNOT00000078785 | ENSRNOG00000056338 | AABR07058884.1 |  |  |
| rno-miR-370-3p | up | ENSRNOT00000012209 | ENSRNOG00000009195 | Tnks1bp1 | tankyrase 1 binding protein 1 |  |
| rno-miR-665 | up | ENSRNOT00000023352 | ENSRNOG00000028426 | Mcf2l | MCF.2 cell line derived transforming sequence-like |  |
| rno-miR-370-3p | up | ENSRNOT00000000697 | ENSRNOG00000000572 | Chst3 | carbohydrate sulfotransferase 3 |  |
| rno-miR-665 | up | ENSRNOT00000087095 | ENSRNOG00000028426 | Mcf2l | MCF.2 cell line derived transforming sequence-like |  |
| rno-miR-370-3p | up | ENSRNOT00000079298 | ENSRNOG00000015701 | Rreb1 | ras responsive element binding protein 1 |  |
| rno-miR-370-3p | up | ENSRNOT00000060218 | ENSRNOG00000015701 | Rreb1 | ras responsive element binding protein 1 |  |
| rno-miR-665 | up | ENSRNOT00000027994 | ENSRNOG00000020579 | Col7a1 | collagen type VII alpha 1 chain |  |
| rno-miR-370-3p | up | ENSRNOT00000088712 | ENSRNOG00000010653 | Elmsan1 | ELM2 and Myb/SANT domain containing 1 |  |
| rno-miR-370-3p | up | ENSRNOT00000054937 | ENSRNOG00000020457 | Tacc2 | transforming, acidic coiled-coil containing protein 2 |  |
| rno-miR-370-3p | up | ENSRNOT00000067912 | ENSRNOG00000006004 | Phc2 | polyhomeotic homolog 2 |  |
| rno-miR-370-3p | up | ENSRNOT00000077908 | ENSRNOG00000020457 | Tacc2 | transforming, acidic coiled-coil containing protein 2 |  |
| rno-miR-370-3p | up | ENSRNOT00000070961 | ENSRNOG00000048430 | Myo18b | myosin XVIIIb |  |
| rno-miR-370-3p | up | ENSRNOT00000010015 | ENSRNOG00000007596 | Rffl | ring finger and FYVE-like domain containing E3 ubiquitin protein ligase |  |
| rno-miR-370-3p | up | ENSRNOT00000004249 | ENSRNOG00000042086 | Rab26 | RAB26, member RAS oncogene family |  |
| rno-miR-665 | up | ENSRNOT00000057386 | ENSRNOG00000016281 | Col4a1 | collagen type IV alpha 1 chain |  |
| rno-miR-665 | up | ENSRNOT00000064078 | ENSRNOG00000003349 | Col23a1 | collagen type XXIII alpha 1 chain |  |
| rno-miR-370-3p | up | ENSRNOT00000002776 | ENSRNOG00000002031 | Naa11 | N(alpha)-acetyltransferase 11, NatA catalytic subunit |  |
| rno-miR-665 | up | ENSRNOT00000074816 | ENSRNOG00000050706 | Col25a1 | collagen type XXV alpha 1 chain |  |
| rno-miR-370-3p | up | ENSRNOT00000065848 | ENSRNOG00000004591 | Alg12 | ALG12, alpha-1,6-mannosyltransferase |  |
| rno-miR-665 | up | ENSRNOT00000005073 | ENSRNOG00000003736 | Col5a2 | collagen type V alpha 2 chain |  |
| rno-miR-665 | up | ENSRNOT00000012927 | ENSRNOG00000009735 | Fibcd1 | fibrinogen C domain containing 1 |  |
| rno-miR-370-3p | up | ENSRNOT00000041389 | ENSRNOG00000007596 | Rffl | ring finger and FYVE-like domain containing E3 ubiquitin protein ligase |  |
| rno-miR-370-3p | up | ENSRNOT00000086868 | ENSRNOG00000013661 | Kif26a | kinesin family member 26A |  |
| rno-miR-665 | up | ENSRNOT00000071463 | ENSRNOG00000050706 | Col25a1 | collagen type XXV alpha 1 chain |  |
| rno-miR-665 | up | ENSRNOT00000075728 | ENSRNOG00000050706 | Col25a1 | collagen type XXV alpha 1 chain |  |
| rno-miR-665 | up | ENSRNOT00000045270 | ENSRNOG00000033618 | Col28a1 | collagen type XXVIII alpha 1 chain |  |
| rno-miR-370-3p | up | ENSRNOT00000018278 | ENSRNOG00000013661 | Kif26a | kinesin family member 26A |  |
| rno-miR-434-3p | up | ENSRNOT00000072399 | ENSRNOG00000048751 | Rtl1 | retrotransposon-like 1 |  |
| rno-miR-370-3p | up | ENSRNOT00000076089 | ENSRNOG00000007596 | Rffl | ring finger and FYVE-like domain containing E3 ubiquitin protein ligase |  |
| rno-miR-370-3p | up | ENSRNOT00000091715 | ENSRNOG00000002879 | Psen2 | presenilin 2 |  |
| rno-miR-370-3p | up | ENSRNOT00000060832 | ENSRNOG00000000851 | Bag6 | BCL2-associated athanogene 6 |  |
| rno-miR-370-3p | up | ENSRNOT00000013943 | ENSRNOG00000010485 | Prpmp5 | proline-rich protein MP5 |  |
| rno-miR-370-3p | up | ENSRNOT00000076596 | ENSRNOG00000007596 | Rffl | ring finger and FYVE-like domain containing E3 ubiquitin protein ligase |  |
| rno-miR-370-3p | up | ENSRNOT00000082113 | ENSRNOG00000030714 | Bsn | bassoon (presynaptic cytomatrix protein) |  |
| rno-miR-370-3p | up | ENSRNOT00000001129 | ENSRNOG00000000851 | Bag6 | BCL2-associated athanogene 6 |  |
| rno-miR-370-3p | up | ENSRNOT00000085598 | ENSRNOG00000000851 | Bag6 | BCL2-associated athanogene 6 |  |
