# Supplementary material for: Differential Expression Profile of microRNAs and Tight Junction in the Lung Tissues of Rat With Mitomycin-C-Induced Pulmonary Veno-Occlusive Disease
Source: Front Cardiovasc Med. 2022 Feb 16;9:746888. doi: 10.3389/fcvm.2022.746888 (PMC8889576; doi:10.3389/fcvm.2022.746888)
Supplement: Supplementary file 6 [file Table_6.docx]

**Supplement table 6.** Identified target genes regulated by miRNA-214-5p.

| **Target genes** | **Diseases/Cells** | **References** |
| --- | --- | --- |
| FASLG | Cardiac cells | Lu et al. [1] |
| ROCK1 | Hepatocellular carcinoma cell | Hu et al. [2] |
| CRMP5 | Human prostate cancer cell | Zheng et al. [3] |
| JAG1 | Pancreatic cancer cell | Cao et al [4] |
| Jagged 1 | Trophoblast cells | Gong et al. [5] |
| TM4SF1 | Fibroblasts | Xu et al. [6] |
| P53 | H9c2 cell | Teng et al. [7] |
| ROCK1 | Human osteosarcoma cells | Zhang et al. [8] |
| [KLF5](https://pubmed.ncbi.nlm.nih.gov/30206974/) | Hepatocellular carcinoma cell | Pang et al.[9] |
| SOX4 | Osteosarcoma cell | Chen et al.[10] |
| SOX4 | U2OS and HOS cells | Zhou et al. [11] |
| CXCR5 | Glioblastoma | Yang et al. [12] |
| TGF-β/Smad2 | Bone marrow stem cell | Qiu et al. [13] |
| UGT1A | HepG2 and Huh7 cell | Paulusch et al. [14] |
| WASL | Hepatocellular Carcinoma cell | Li et al. [15] |
| BMP2 | Bone marrow mesenchymal stem cell | Li et al. [16] |
| CIZ1 | Non-small cell lung cancer cell | Chen et al. [17] |
| SOX4 | [Cervical cancer cells](https://pubmed.ncbi.nlm.nih.gov/32106739/) | Guo et al. [18] |
| COL4A1 | Osteoblastic cell | Li et al. [19] |
| PPARGC1B | Chondrocytes | Xu et al. [20] |
| DDX5 | Osteosarcoma cell | Mao et al. [21] |
| GLP-1R | Mouse proximal tubular cell | Guo et al. [22] |
| RAB14 | [Esophageal cancer cells](https://pubmed.ncbi.nlm.nih.gov/31957829/) | Liu et al. [23] |
| SEMA4C | [Cervical cancer cells](https://pubmed.ncbi.nlm.nih.gov/33394293/) | Li et al. [24] |
| E2F2 | Retinoblastoma cell | Zhang et al. [25] |
| E2F2 | Pancreatic cancer cell | Yao et al. [26] |
| CRY2 | Ovarian cancer cell line | Chen et al. [27] |
| BMP2 | Bone marrow mesenchymal stem cell | He et al. [28] |
| E2F2 | Renal cell carcinoma cell | Guo et al. [29] |
| TWIST1 | JEG-3 cells | Wang et al. [30] |
| HCG11 | Colorectal cancer cell | Xie et al. [31] |
| CLIC4 | Breast cancer cell | Lv et al. [32] |
| HNF1B | Human embryonic kidney cell | Goda et al. [33] |
| INHBA | Granulosa Cell | Ma et al. [34] |
| JAG1 | Colorectal cancer | Pan et al. [35] |
| TWIST1 | Colorectal cancer | Jing et al. [36] |
| UCP-2 | [Visceral adipose tissues](https://www.embase.com/a/#/search/results?subaction=viewrecord&rid=2&page=4&id=L624032256) | Kurylowicz et al. [37] |

**References**

1. Lu Y, Xi J, Zhang Y, Li C, Chen W, Hu X, et al. MicroRNA-214-5p protects against myocardial ischemia reperfusion injury through targeting the FAS ligand. Arch Med Sci. 2019;16(5):1119-1129.
2. Hu M, Han Y, Zhang Y, Zhou Y, Ye L. Retracted Article: lncRNA TINCR sponges miR-214-5p to upregulate ROCK1 in hepatocellular carcinoma. BMC Med Genet. 2020;21(1):2.
3. Zheng C, Guo K, Chen B, Wen Y, Xu Y. miR-214-5p inhibits human prostate cancer proliferation and migration through regulating CRMP5. Cancer Biomark. 2019;26(2):193-202.
4. Cao TH, Ling X, Chen C, Tang W, Hu DM, Yin GJ. Role of miR-214-5p in the migration and invasion of pancreatic cancer cells. Eur Rev Med Pharmacol Sci. 2018 Nov;22(21):7214-7221.
5. Gong F, Chai W, Wang J, Cheng H, Shi Y, Cui L, et al. miR-214-5p suppresses the proliferation, migration and invasion of trophoblast cells in pre-eclampsia by targeting jagged 1 to inhibit notch signaling pathway. Acta Histochem. 2020;122(3):151527.
6. Xu M, Sun J, Yu Y, Pang Q, Lin X, Barakat M, et al. TM4SF1 involves in miR-1-3p/miR-214-5p-mediated inhibition of the migration and proliferation in keloid by regulating AKT/ERK signaling. Life Sci. 2020;254:117746.
7. Teng Y, Ding M, Wang X, Li H, Guo Q, Yan J, et al. LncRNA RMRP accelerates hypoxia-induced injury by targeting miR-214-5p in H9c2 cells. J Pharmacol Sci. 2020;142(2):69-78.
8. Zhang M, Wang D, Zhu T, Yin R. miR-214-5p Targets ROCK1 and Suppresses Proliferation and Invasion of Human Osteosarcoma Cells. Oncol Res. 2017;25(1):75-81.
9. Pang J, Li Z, Wang G, et al. miR-214-5p targets KLF5 and suppresses proliferation of human hepatocellular carcinoma cells. J Cell Biochem. 2018.
10. Chen H, Liu T, Ouyang H, Lin S, Zhong H, Zhang H, et al. Upregulation of FTX Promotes Osteosarcoma Tumorigenesis by Increasing SOX4 Expression via miR-214-5p. Onco Targets Ther. 2020;13:7125-7136.
11. Zhou Y, Li X, Yang H. LINC00612 functions as a ceRNA for miR-214-5p to promote the proliferation and invasion of osteosarcoma in vitro and in vivo. Exp Cell Res. 2020;392(1):112012.
12. Yang JK, Liu HJ, Wang Y, Li C, Yang JP, Yang L, et al. Exosomal miR-214-5p Released from Glioblastoma Cells Modulates Inflammatory Response of Microglia after Lipopolysaccharide Stimulation through Targeting CXCR5. CNS Neurol Disord Drug Targets. 2019;18(1):78-87.
13. Qiu J, Huang G, Na N, Chen L. MicroRNA-214-5p/TGF-β/Smad2 signaling alters adipogenic differentiation of bone marrow stem cells in postmenopausal osteoporosis. Mol Med Rep. 2018;17(5):6301-6310.
14. Paulusch S, Kalthoff S, Landerer S, Jansen C, Schierwagen R, Klein S, et al. Regulation of uridine diphosphate-glucuronosyltransferase 1A expression by miRNA-214-5p and miRNA-486-3p. Epigenomics. 2021;13(4):271-283.
15. Li H, Wang H, Ren Z. MicroRNA-214-5p Inhibits the Invasion and Migration of Hepatocellular Carcinoma Cells by Targeting Wiskott-Aldrich Syndrome Like. Cell Physiol Biochem. 2018;46(2):757-764.
16. Li L, Fang J, Liu Y, Xiao L. LncRNA LOC100506178 promotes osteogenic differentiation via regulating miR-214-5p-BMP2 axis in human bone marrow mesenchymal stem cells. PeerJ. 2020;8:e8909.
17. Chen YR, Wu YS, Wang WS, Zhang JS, Wu QG. Upregulation of lncRNA DANCR functions as an oncogenic role in non-small lung cancer by regulating miR-214-5p/CIZ1 axis. Eur Rev Med Pharmacol Sci. 2020;24(5):2539-2547.
18. Guo M, Lin B, Li G, Lin J, Jiang X. LncRNA TDRG1 promotes the proliferation, migration, and invasion of cervical cancer cells by sponging miR-214-5p to target SOX4. J Recept Signal Transduct Res. 2020;40(3):281-293.
19. Li QS, Meng FY, Zhao YH, Jin CL, Tian J, Yi XJ. Inhibition of microRNA-214-5p promotes cell survival and extracellular matrix formation by targeting collagen type IV alpha 1 in osteoblastic MC3T3-E1 cells. Bone Joint Res. 2017;6(8):464-471.
20. Xu J, Pei Y, Lu J, Liang X, Li Y, Wang J, et al. LncRNA SNHG7 alleviates IL-1β-induced osteoarthritis by inhibiting miR-214-5p-mediated PPARGC1B signaling pathways. Int Immunopharmacol. 2021;90:107150.
21. Mao X, Guo S, Gao L, Li G. Circ-XPR1 promotes osteosarcoma proliferation through regulating the miR-214-5p/DDX5 axis. Hum Cell. 2021;34(1):122-131.
22. Guo C, Ye FX, Jian YH, Liu CH, Tu ZH, Yang DP. MicroRNA-214-5p aggravates sepsis-related acute kidney injury in mice. Drug Dev Res. 2021.
23. Liu HF, Zhen Q, Fan YK. LINC00963 predicts poor prognosis and promotes esophageal cancer cells invasion via targeting miR-214-5p/RAB14 axis. Eur Rev Med Pharmacol Sci. 2020;24(1):164-173.
24. Li X, Zhang C, Tian Y. Long non-coding RNA TDRG1 promotes hypoxia-induced glycolysis by targeting the miR-214-5p/SEMA4C axis in cervical cancer cells. J Mol Histol. 2021;52(2):245-256.
25. Zhang H, Qiu X, Song Z, Lan L, Ren X, Ye B. CircCUL2 suppresses retinoblastoma cells by regulating miR-214-5p/E2F2 Axis. Anticancer Drugs. 2021.
26. Yao Z, Chen Q, Ni Z, Zhou L, Wang Y, Yang Y, et al. Long Non-Coding RNA Differentiation Antagonizing Nonprotein Coding RNA (DANCR) Promotes Proliferation and Invasion of Pancreatic Cancer by Sponging miR-214-5p to Regulate E2F2 Expression. Med Sci Monit. 2019;25:4544-4552.
27. Chen GY, Zhang ZS, Chen Y, Li Y. Long non-coding RNA SNHG9 inhibits ovarian cancer progression by sponging microRNA-214-5p. Oncol Lett. 2021;21(2):80.
28. He Q, Li R, Hu B, Li X, Wu Y, Sun P, et al. Stromal cell-derived factor-1 promotes osteoblastic differentiation of human bone marrow mesenchymal stem cells via the lncRNA-H19/miR-214-5p/BMP2 axis. J Gene Med. 2021;23(9):e3366.
29. Guo R, Zou B, Liang Y, Bian J, Xu J, Zhou Q, et al. LncRNA RCAT1 promotes tumor progression and metastasis via miR-214-5p/E2F2 axis in renal cell carcinoma. Cell Death Dis. 2021;12(7):689.
30. Wang Y, Zhang Z. Increased expression of lncRNA SNHG7 promotes the cell viability, migration, and invasion in pre-eclampsia via modulating the miR-214-5p/TWIST1 axis. Hypertens Pregnancy. 2021:1-10.
31. Xie J, Zhu J, Pang J, Ma Y. HLA complex group 11 is involved in colorectal carcinoma cisplatin resistance via the miR-214-5p/SOX4 axis. Oncol Lett. 2021 Jul;22(1):535.
32. Lv Y, Dong K, Gao H. Long non-coding RNA TDRG1 facilitates cell proliferation, migration and invasion in breast cancer via targeting miR-214-5p/CLIC4 axis. Cancer Biol Ther. 2021;22(3):248-256.
33. Goda N, Murase H, Kasezawa N, Goda T, Yamakawa-Kobayashi K. Polymorphism in microRNA-binding site in HNF1B influences the susceptibility of type 2 diabetes mellitus: a population based case-control study. BMC Med Genet. 2015;16:75.
34. Ma M, Wang H, Zhang Y, Zhang J, Liu J, Pan Z. circRNA-Mediated Inhibin-Activin Balance Regulation in Ovarian Granulosa Cell Apoptosis and Follicular Atresia. Int J Mol Sci. 2021;22(17):9113.
35. Pan L, Du M, Liu H, Cheng B, Zhu M, Jia B, et al. LncRNA FTX promotes the malignant progression of colorectal cancer by regulating the miR-214-5p–JAG1 axis. Annals of Translational Medicine 2021;9(17):1369.
36. Jing L, Shen J, Liu Y, Zhu X, Gu X. MIR-214-5P inhibits migration and invasion of colorectal cancer cells by targeting TWIST1. Acta Medica Mediterranea. 2020 36(6):3457-3463.
37. Kurylowicz A, Owczarz M, Polosak J, Jonas M, Jonas M, Lisik W, et al. MicroRNAs regulate expression of uncoupling protein 2 gene (UCP-2) in visceral adipose tissues of obese individuals. Diabetologia. 2018;61:Supplement 1 (S278-).
