# Supplementary material for: Differential Expression Profile of microRNAs and Tight Junction in the Lung Tissues of Rat With Mitomycin-C-Induced Pulmonary Veno-Occlusive Disease
Source: Front Cardiovasc Med. 2022 Feb 16;9:746888. doi: 10.3389/fcvm.2022.746888 (PMC8889576; doi:10.3389/fcvm.2022.746888)
Supplement: Supplementary file 7 [file Table_7.docx]

**Supplement table 7.** Identified target genes regulated by miRNA-141-3p.

| **Target genes** | **Diseases/Cells** | **References** |
| --- | --- | --- |
| DLC1 | Nasopharyngeal carcinoma cell | Mu et al. [1] |
| NME1 | Nasopharyngeal epithelial cell | Li et al. [2] |
| RIPK1 | Human Caco-2 cell | Li et al. [3] |
| MNX1 | Intestinal epithelial cell | Chen et al [4] |
| Keap1 | Vascular smooth muscle cell | Zhang et al. [5] |
| GP73 | Hepatocellular carcinoma cell | Hou et al. [6] |
| Mitf | B16-4A5 cell | Itoh et al. [7] |
| EGFR | Colorectal cancer cell | Xing et al. [8] |
| ZNF217 | Human fibroblast cell | Liang et al. [9] |
| UGT1A | HEK293 cell | Tatsumi et al. [10] |
| KLF-12 | Endometrial stromal cell | Zhang et al. [11] |
| HMGB1 | Human normal mammary epithelial cell | Sun et al. [12] |
| HMGB1 | Nasal epithelial cell | Zhu et al. [13] |
| FUS | Osteosarcoma cell | Wang et al. [14] |
| SIRT1 | PC12-cell | Zheng et al. [15] |
| TRAF5 | Colorectal cancer cell | Liang et al. [16] |
| EGFR | Osteosarcoma cell | Wang et al. [17] |
| CDK8 | Breast cancer cell | Song et al. [18] |
| DAPK1 | Granulosa cell | Li et al. [19] |
| P53 | Glioma cell | Zhou et al. [20] |
| Yin Yang 1 | Papillary thyroid carcinoma cell | Fang et al. [21] |
| GLI2 | Osteosarcoma cell | Wang et al. [22] |
| ZFR | NSCLC cell | Li et al. [23] |
| TRAF5 | T cell acute lymphoblastic leukemia cell | Zhou et al. [24] |
| Igf2 | Trophoblast stem cell | Saha et al. [25] |
| YAP | Stem cells from apical papilla | Li et al. [26] |
| FoxC1 | Synovial fibroblasts | Wang et al. [27] |
| DOK5 | Retinal vascular epithelial cell | Zhang et al. [28] |
| GAB1 | Fibroblasts | Feng et al. [29] |
| KLF9 | Prostate cancer cell | Li et al. [30] |
| FOXA2 | Cervical cancer cell | Li et al. [31] |
| ROR2 | Human vascular smooth muscle cell | Tang et al. [32] |
| SDF-1 | Bone marrow stromal cell | Periyasamy-Thandavan et al. [33] |
| TRAF5 | Prostate cancer cell | Huang et al. [34] |
| JAG1 | Neuropathic cell | Li et al. [35] |
| CCDC80 | Vascular smooth muscle cell | Gong et al. [36] |
| TSC1 | Esophageal cancer cell | Phatak et al. [37] |
| ZEB2/ E2F3 | Hepatocellular carcinoma cell | Ye et al. [38] |
| ATF5 | Glioma cell | Wang et al. [39] |
| PTEN | Esophageal squamous cell carcinoma cell | Jin et al. [40] |
| SNHG16 | Mice mesangial cell | Jiang et al. [41] |
| NCAM1 | Human ameloblastoma cell | Guan et al. [42] |
| WIPF1 | Gastric cancer cell | Liu et al. [43] |
| KLF9 | Nasopharyngeal carcinoma cell | Yi et al. [44] |
| STAT4 | Gastric cancer cell | Zhou et al. [45] |
| BDH2 | Pancreatic cancer cell | Cui et al. [46] |
| CTNNB1 | Human renal proximal tubular epithelial cells | Zhang et al. [47] |
| PTEN | HepG2 cell | Ji et al. [48] |
| HMGB1 | BV2 microglial cell | Shen et al. [49] |
| PTEN | Mouse hepatocyte cell | Dang et al. [50] |
| ZEB1/ ZEB2 | Breast cancer cell | Zhang et al. [51] |
| IGF2BP2 | Neural stem cell | Jiang et al. [52] |
| PHLPP2 | Esophageal epithelial cell | Ishibashi et al. [53] |
| GSN | Hepatocellular carcinoma cell | Xiao et al. [54] |
| YAP1 | Glioma cell | Yu et al. [55] |
| β-catenin | Mesenchymal stem cell | Song et al. [56] |
| STAT4 | [Myocardial](javascript:;) [cell](javascript:;) | Pan et al. [57] |
| XIST | Human embryonic kidney cell | Sun et al. [58] |
| Fkbp5/Fibin | C2C12 cell | Lee et al. [59] |
| RARB | Th17 cell | Bahmani et al. [60] |
| GLS | Osteosarcoma cell | Zhou et al. [61] |
| BCL2L13 | Primary OA chondrocytes | Zhang et al. [62] |
| NEK6 | Clear cell renal cell carcinoma cell | Liu et al. [63] |
| ZEB1 | Alveolar type II epithelial (RLE-6TN) cell | Qian et al. [64] |
| SIX1 | Osteosarcoma cell | Ji et al. [65] |
| HMGB1 | Astrocytes | Fang et al. [66] |
| Wnt5a | Vascular smooth muscle cell | Zhang et al. [67] |
| Notch2 | Human umbilical vein endothelial cell | Ling et al. [68] |
| MAP4K4 | B-cell lymphoma cell | Li et al. [69] |
| TGF-β2 | Gastric cancer | Lei et al. [70] |
| TTR | JEG3 cell | Cao et al. [71] |
| PTEN | Diabetic Renal Fibrosis | Li et al. [72] |
| ELF3 | Hepatocellular carcinoma | Zheng et al. [73] |
| Bcl2 | Colon cancer cell | Tong et al. [74] |
| YAP1 | Endometriosis | Wang et al. [75] |
| Cyclin D | Renal cell carcinoma | Dasgupta et al. [76] |
| DAPK1 | Endometrial cancer | Cui et al. [77] |
| ZMPSTE24 | Human mesenchymal stem cell | Yu et al. [78] |
| AR | Prostate cancer cell | Wang et al. [79] |
| RBMS3 | Breast cancer | Dong et al. [80] |
| ZEB1/ZEB2 | Non-small cell lung cancer | Sun et al. [81] |
| KLF12 | Breast cancer | Zhou et al. [82] |
| KEAP1 | Lung cancer | Wang et al. [83] |
| MCL1 | Hypertrophic scar | Yang et al .[84] |
| LATS2 | Non-small cell lung cancer | Zhang et al. [85] |
| ZEB1 | Preeclampsia | Yang et al. [86] |
| KEAP1 | Intervertebral disc | Xu et al. [87] |
| ZEB1 | Hepatocellular carcinoma | Cheng et al. [88] |
| HDAC4 | Coronary atherosclerosis disease | Zhang et al. [89] |
| SNHG16/SUSD2 | Pneumonia | Xia et al. [90] |
| FUS | Nucleus pulposus cell | Guo et al. [91] |
| TTR | Placental trophoblast cell | Saha et al. [92] |
| TfR1 | Pancreatic adenocarcinoma | Miyazawa et al. [93] |
| AUF1 | Bladder cancer | Li et al. [94] |
| UGT1A1 | HEK293 cell | Papageorgiou et al. [95] |
| RHEB | Hepatocellular carcinoma | Huang et al. [96] |
| MYPT1 | Metastatic prostate cancer | Chao et al. [97] |
| IGF1R | Prostate cancer | Zhang et al. [98] |
| YAP1 | Intrahepatic cholangiocarcinoma | Xia et al. [99] |

**References**

1. Mu JW, Zhou XY, Wang QJ, Han LH, Jiao JB. MicroRNA-141-3p promoted the progression of nasopharyngeal carcinoma through targeting DLC1. Eur Rev Med Pharmacol Sci. 2020;24(21):11105-11113.
2. Li M, Huang H, Cheng F, Hu X, Liu J. miR-141-3p promotes proliferation and metastasis of nasopharyngeal carcinoma by targeting NME1. Adv Med Sci. 2020;65(2):252-258.
3. Li X, Wang Y, Wang Y, He X. MiR-141-3p ameliorates RIPK1-mediated necroptosis of intestinal epithelial cells in necrotizing enterocolitis. Aging (Albany NY). 2020;12(18):18073-18083.
4. Chen H, Zeng L, Zheng W, Li X, Lin B. Increased Expression of microRNA-141-3p Improves Necrotizing Enterocolitis of Neonates Through Targeting MNX1. Front Pediatr. 2020;8:385.\
5. Zhang C, Kong X, Ma D. miR-141-3p inhibits vascular smooth muscle cell proliferation and migration via regulating Keap1/Nrf2/HO-1 pathway. IUBMB Life. 2020;72(10):2167-2179.
6. Hou X, Yang L, Jiang X, Liu Z, Li X, Xie S, et al. Role of microRNA-141-3p in the progression and metastasis of hepatocellular carcinoma cell. Int J Biol Macromol. 2019;128:331-339.
7. Itoh T, Fukatani K, Nakashima A, Suzuki K. MicroRNA-141-3p and microRNA-200a-3p regulate α-melanocyte stimulating hormone-stimulated melanogenesis by directly targeting microphthalmia-associated transcription factor. Sci Rep. 2020;10(1):2149.
8. Xing Y, Jing H, Zhang Y, Suo J, Qian M. MicroRNA-141-3p affected proliferation, chemosensitivity, migration and invasion of colorectal cancer cells by targeting EGFR. Int J Biochem Cell Biol. 2020;118:105643.
9. Liang ZH, Pan YC, Lin SS, Qiu ZY, Zhang Z. LncRNA MALAT1 promotes wound healing via regulating miR-141-3p/ZNF217 axis. Regen Ther. 2020;15:202-209.
10. Tatsumi N, Tokumitsu S, Nakano M, Fukami T, Nakajima Ml. miR-141-3p commonly regulates human UGT1A isoforms via different mechanisms. Drug Metab Pharmacokinet. 2018;33(4):203-210.
11. Zhang Y, Yan J, Pan X. miR-141-3p affects apoptosis and migration of endometrial stromal cells by targeting KLF-12. Pflugers Arch. 2019;471(8):1055-1063.
12. Sun S, Ma J, Xie P, Wu Z, Tian X. Hypoxia-responsive miR-141-3p is involved in the progression of breast cancer via mediating the HMGB1/HIF-1α signaling pathway. J Gene Med. 2020;22(10):e3230.
13. Zhu YM, Wu F, Zhou JY. Analysis the effect of miR-141-3p/HMGB1 in LPS-induced mucus production and the apoptosis in nasal epithelial cells. Kaohsiung J Med Sci. 2020 Aug;36(8):622-629.
14. Wang L. MiR-141-3p overexpression suppresses the malignancy of osteosarcoma by targeting FUS to degrade LDHB. Biosci Rep. 2020;40(6):BSR20193404.
15. Zheng Y, Dong L, Liu N, Luo X, He Z. Mir-141-3p Regulates Apoptosis and Mitochondrial Membrane Potential via Targeting Sirtuin1 in a 1-Methyl-4-Phenylpyridinium in vitro Model of Parkinson's Disease. Biomed Res Int. 2020;2020:7239895.
16. Liang Z, Li X, Liu S, Li C, Wang X, Xing J. MiR-141-3p inhibits cell proliferation, migration and invasion by targeting TRAF5 in colorectal cancer. Biochem Biophys Res Commun. 2019;514(3):699-705.
17. Wang J, Wang G, Li B, Qiu C, He M. miR-141-3p is a key negative regulator of the EGFR pathway in osteosarcoma. Onco Targets Ther. 2018;11:4461-4478.
18. Song W, Wu S, Wu Q, Zhou L, Yu L, Zhu B, et al. The microRNA-141-3p/ CDK8 pathway regulates the chemosensitivity of breast cancer cells to trastuzumab. J Cell Biochem. 2019;120(8):14095-14106.
19. Li D, Xu D, Xu Y, Chen L, Li C, Dai X, et al. MicroRNA-141-3p targets DAPK1 and inhibits apoptosis in rat ovarian granulosa cells. Cell Biochem Funct. 2017;35(4):197-20.
20. Zhou X, Wu W, Zeng A, Nie E, Jin X, Yu T, et al. MicroRNA-141-3p promotes glioma cell growth and temozolomide resistance by directly targeting p53. Oncotarget. 2017;8(41):71080-71094.
21. Fang M, Huang W, Wu X, Gao Y, Ou J, Zhang X, et al. MiR-141-3p Suppresses Tumor Growth and Metastasis in Papillary Thyroid Cancer via Targeting Yin Yang 1. Anat Rec (Hoboken). 2019;302(2):258-268.
22. Wang N, Li P, Liu W, Wang N, Lu Z, Feng J, et al. miR-141-3p suppresses proliferation and promotes apoptosis by targeting GLI2 in osteosarcoma cells. Oncol Rep. 2018;39(2):747-754.
23. Li W, Cui Y, Wang D, Wang Y, Wang L. MiR-141-3p functions as a tumor suppressor through directly targeting ZFR in non-small cell lung cancer. Biochem Biophys Res Commun. 2019;509(3):647-656.
24. Zhou R, Mo W, Wang S, Zhou W, Chen X, Pan S. miR-141-3p and TRAF5 Network Contributes to the Progression of T-Cell Acute Lymphoblastic Leukemia. Cell Transplant. 2019;28(1_suppl):59S-65S.
25. Saha S, Choudhury J, Ain R. MicroRNA-141-3p and miR-200a-3p regulate insulin-like growth factor 2 during mouse placental development. Mol Cell Endocrinol. 2015;414:186-93.
26. Li Z, Ge X, Lu J, Bian M, Li N, Wu X, et al. MiR-141-3p regulates proliferation and senescence of stem cells from apical papilla by targeting YAP. Exp Cell Res. 2019;383(2):111562.
27. Wang J, Wang Y, Zhang H, Chang J, Lu M, Gao W, et al. Identification of a novel microRNA-141-3p/Forkhead box C1/β-catenin axis associated with rheumatoid arthritis synovial fibroblast function in vivo and in vitro. Theranostics. 2020;10(12):5412-5434.
28. Zhang LQ, Cui H, Yu YB, Shi HQ, Zhou Y, Liu MJ. MicroRNA-141-3p inhibits retinal neovascularization and retinal ganglion cell apoptosis in glaucoma mice through the inactivation of Docking protein 5-dependent mitogen-activated protein kinase signaling pathway. J Cell Physiol. 2019;234(6):8873-8887.
29. Feng J, Xue S, Pang Q, Rang Z, Cui F. miR-141-3p inhibits fibroblast proliferation and migration by targeting GAB1 in keloids. Biochem Biophys Res Commun. 2017;490(2):302-308.
30. Li JZ, Li J, Wang HQ, Li X, Wen B, Wang YJ. MiR-141-3p promotes prostate cancer cell proliferation through inhibiting kruppel-like factor-9 expression. Biochem Biophys Res Commun. 2017;482(4):1381-1386.
31. Li JH, Zhang Z, Du MZ, Guan YC, Yao JN, Yu HY, et al. microRNA-141-3p fosters the growth, invasion, and tumorigenesis of cervical cancer cells by targeting FOXA2. Arch Biochem Biophys. 2018;657:23-30.
32. Tang Y, Hu J, Zhong Z, Liu Y, Wang Y. Long Noncoding RNA TUG1 Promotes the Function in ox-LDL-Treated HA-VSMCs via miR-141-3p/ROR2 Axis. Cardiovasc Ther. 2020;2020:6758934.
33. Periyasamy-Thandavan S, Burke J, Mendhe B, Kondrikova G, Kolhe R, Hunter M, et al. MicroRNA-141-3p Negatively Modulates SDF-1 Expression in Age-Dependent Pathophysiology of Human and Murine Bone Marrow Stromal Cells. J Gerontol A Biol Sci Med Sci. 2019;74(9):1368-1374.
34. Huang S, Wa Q, Pan J, Peng X, Ren D, Huang Y, et al. Downregulation of miR-141-3p promotes bone metastasis via activating NF-κB signaling in prostate cancer. J Exp Clin Cancer Res. 2017;36(1):173.
35. Li H, Fan L, Zhang Y, Cao Y, Liu X. SNHG16 aggravates chronic constriction injury-induced neuropathic pain in rats via binding with miR-124-3p and miR-141-3p to upregulate JAG1. Brain Res Bull. 2020;165:228-237.
36. Gong D, Zhao ZW, Zhang Q, Yu XH, Wang G, Zou J, et al. The Long Noncoding RNA Metastasis-Associated Lung Adenocarcinoma Transcript-1 Regulates CCDC80 Expression by Targeting miR-141-3p/miR-200a-3p in Vascular Smooth Muscle Cells. J Cardiovasc Pharmacol. 2020;75(4):336-343.
37. Phatak P, Noe M, Asrani K, Chesnick IE, Greenwald BD, Donahue JM. MicroRNA-141-3p regulates cellular proliferation, migration, and invasion in esophageal cancer by targeting tuberous sclerosis complex 1. Mol Carcinog. 2021;60(2):125-137.
38. Ye J, Tan L, Fu Y, Xu H, Wen L, Deng Y, et al. LncRNA SNHG15 promotes hepatocellular carcinoma progression by sponging miR-141-3p. J Cell Biochem. 2019;120(12):19775-19783.
39. Wang M, Hu M, Li Z, Qian D, Wang B, Liu DX. miR-141-3p functions as a tumor suppressor modulating activating transcription factor 5 in glioma. Biochem Biophys Res Commun. 2017;490(4):1260-1267.
40. Jin YY, Chen QJ, Xu K, Ren HT, Bao X, Ma YN, et al. Involvement of microRNA-141-3p in 5-fluorouracil and oxaliplatin chemo-resistance in esophageal cancer cells via regulation of PTEN. Mol Cell Biochem. 2016;422(1-2):161-170.
41. Jiang X, Ru Q, Li P, Ge X, Shao K, Xi L, et al. LncRNA SNHG16 induces proliferation and fibrogenesis via modulating miR-141-3p and CCND1 in diabetic nephropathy. Gene Ther. 2020;27(12):557-566.
42. Guan G, Niu X, Qiao X, Wang X, Liu J, Zhong M. Upregulation of Neural Cell Adhesion Molecule 1 (NCAM1) by hsa-miR-141-3p Suppresses Ameloblastoma Cell Migration. Med Sci Monit. 2020;26:e923491.
43. Liu Y, Lin W, Dong Y, Li X, Lin Z, et al. Long noncoding RNA HCG18 up-regulates the expression of WIPF1 and YAP/TAZ by inhibiting miR-141-3p in gastric cancer. Cancer Med. 2020;9(18):6752-6765.
44. Yi TW, Lv XX, Fan H, Zan N, Su XD. LncRNA SNHG15 promotes the proliferation of nasopharyngeal carcinoma via sponging miR-141-3p to upregulate KLF9. Eur Rev Med Pharmacol Sci. 2020;24(12):6744-6751.
45. Zhou Y, Zhong JH, Gong FS, Xiao J. MiR-141-3p suppresses gastric cancer induced transition of normal fibroblast and BMSC to cancer-associated fibroblasts via targeting STAT4. Exp Mol Pathol. 2019;107:85-94.
46. Cui XP, Wang CX, Wang ZY, Li J, Tan YW, Gu ST, et al. LncRNA TP73-AS1 sponges miR-141-3p to promote the migration and invasion of pancreatic cancer cells through the up-regulation of BDH2. Biosci Rep. 2019;39(3):BSR20181937.
47. Zhang B, Zhao C, Hou L, Wu Y. Silencing of the lncRNA TUG1 attenuates the epithelial-mesenchymal transition of renal tubular epithelial cells by sponging miR-141-3p via regulating β-catenin. Am J Physiol Renal Physiol. 2020;319(6):F1125-F1134.
48. Ji J, Qin Y, Ren J, Lu C, Wang R, Dai X, et al. Mitochondria-related miR-141-3p contributes to mitochondrial dysfunction in HFD-induced obesity by inhibiting PTEN. Sci Rep. 2015;5:16262.
49. Shen WS, Xu XQ, Zhai NN, Zhou ZS, Shao J, Yu YH. Potential mechanisms of microRNA-141-3p to alleviate chronic inflammatory pain by downregulation of downstream target gene HMGB1: in vitro and in vivo studies. Gene Ther. 2017;24(6):353-360.
50. Dang SY, Leng Y, Wang ZX, Xiao X, Zhang X, Wen T, et al. Exosomal transfer of obesity adipose tissue for decreased miR-141-3p mediate insulin resistance of hepatocytes. Int J Biol Sci. 2019;15(2):351-368.
51. Zhang Y, Li J, Jia S, Wang Y, Kang Y, Zhang W. Down-regulation of lncRNA-ATB inhibits epithelial-mesenchymal transition of breast cancer cells by increasing miR-141-3p expression. Biochem Cell Biol. 2019;97(2):193-200.
52. Jiang Q, Wang Y, Shi X. Propofol Inhibits Neurogenesis of Rat Neural Stem Cells by Upregulating MicroRNA-141-3p. Stem Cells Dev. 2017;26(3):189-196.
53. Ishibashi O, Akagi I, Ogawa Y, Inui T. MiR-141-3p is upregulated in esophageal squamous cell carcinoma and targets pleckstrin homology domain leucine-rich repeat protein phosphatase-2, a negative regulator of the PI3K/AKT pathway. Biochem Biophys Res Commun. 2018;501(2):507-513.
54. Xiao Y, Liu G, Sun Y, Gao Y, Ouyang X, Chang C, et al. Targeting the estrogen receptor alpha (ERα)-mediated circ-SMG1.72/miR-141-3p/Gelsolin signaling to better suppress the HCC cell invasion. Oncogene. 2020;39(12):2493-2508.
55. Yu M, Yi B, Zhou W, Gong W, Li G, Yu S. Linc00475 promotes the progression of glioma by regulating the miR-141-3p/YAP1 axis. J Cell Mol Med. 2021;25(1):463-472.
56. Song G, Zhou J, Song R, Liu D, Yu W, Xie W, et al. Long noncoding RNA H19 regulates the therapeutic efficacy of mesenchymal stem cells in rats with severe acute pancreatitis by sponging miR-138-5p and miR-141-3p. Stem Cell Res Ther. 2020;11(1):420.
57. Pan A, Tan Y, Wang Z, Xu G. STAT4 silencing underlies a novel inhibitory role of microRNA-141-3p in inflammation response of mice with experimental autoimmune myocarditis. Am J Physiol Heart Circ Physiol. 2019;317(3):H531-H540.
58. Sun J, Zhang Y. LncRNA XIST enhanced TGF-β2 expression by targeting miR-141-3p to promote pancreatic cancer cells invasion. Biosci Rep. 2019;39(7):BSR20190332.
59. Lee H, Kim YI, Nirmala FS, Kim JS, Seo HD, Ha TY, et al. MiR-141-3p promotes mitochondrial dysfunction in ovariectomy-induced sarcopenia via targeting Fkbp5 and Fibin. Aging (Albany NY). 2021;13(4):4881-4894.
60. Bahmani L, Baghi M, Peymani M, Javeri A, Ghaedi K. MiR-141-3p and miR-200a-3p are involved in Th17 cell differentiation by negatively regulating RARB expression. Hum Cell. 2021;34(5):1375-1387.
61. Zhou X, Wei P, Wang X, Zhang J, Shi Y. miR-141-3p promotes the cisplatin sensitivity of osteosarcoma cell through targeting the Glutaminase (GLS)-mediated glutamine metabolism. Curr Mol Med. 2021.
62. Zhang X, Huang CR, Pan S, Pang Y, Chen YS, Zha GC, et al. Long non-coding RNA SNHG15 is a competing endogenous RNA of miR-141-3p that prevents osteoarthritis progression by upregulating BCL2L13 expression. Int Immunopharmacol. 2020;83:106425.
63. Liu Y, Fu W, Yin F, et al. miR-141-3p suppresses development of clear cell renal cell carcinoma by regulating NEK6. Anticancer Drugs. 2021.
64. Liu Y, Fu W, Yin F, Xia L, Zhang Y, Wang B, et al. lncRNA ZEB1-AS1 promotes pulmonary fibrosis through ZEB1-mediated epithelial-mesenchymal transition by competitively binding miR-141-3p. Cell Death Dis. 2019;10(2):129.
65. Ji Q, Zhu J, Fang CL, Jin H, Zhan DP, Huang J. Down-regulation of MIAT suppresses osteosarcoma progression by acting as a ceRNA for miR-141-3p to regulate SIX1-mediated PI3K/AKT pathway. Eur Rev Med Pharmacol Sci. 2020;24(5):2218-2228.
66. Fang X, Wang H, Zhuo Z, Tian P, Chen Z, Wang Y, et al. miR-141-3p inhibits the activation of astrocytes and the release of inflammatory cytokines in bacterial meningitis through down-regulating HMGB1. Brain Res. 2021;1770:147611.
67. Zhang F, Sun P, Yuan N. miR-141-3p Reduces Cell Migration and Proliferation in an In Vitro Modelof Atherosclerosis by Targeting Wnt5a. J Invest Surg. 2021:1-7.
68. Ling Z, Chen M, Li T, Qian Y, Li C, et al. MiR-141-3p downregulation promotes tube formation, migration, invasion and inhibits apoptosis in hypoxia-induced human umbilical vein endothelial cells by targeting Notch2. Reprod Biol. 2021;21(2):100483.
69. Li Q, Li B, Lu CL, Wang JY, Gao M, Gao W. LncRNA LINC01857 promotes cell growth and diminishes apoptosis via PI3K/mTOR pathway and EMT process by regulating miR-141-3p/MAP4K4 axis in diffuse large B-cell lymphoma. Cancer Gene Ther. 2021;28(9):1046-1057.
70. Lei K, Liang X, Gao Y, Xu B, Xu Y, Li Y, et al. Lnc-ATB contributes to gastric cancer growth through a MiR-141-3p/TGFβ2 feedback loop. Biochem Biophys Res Commun. 2017 Mar 11;484(3):514-521.
71. Cao G, Cui R, Liu C, Zhang Z. MicroRNA regulation of transthyretin in trophoblast biofunction and preeclampsia. Arch Biochem Biophys. 2019;676:108129.
72. Li XY, Wang SS, Han Z, Han F, Chang YP, Yang Y, et al. Triptolide Restores Autophagy to Alleviate Diabetic Renal Fibrosis through the miR-141-3p/PTEN/Akt/mTOR Pathway. Mol Ther Nucleic Acids. 2017;9:48-56.
73. Zheng L, Xu M, Xu J, Wu K, Fang Q, Liang Y, et al. ELF3 promotes epithelial-mesenchymal transition by protecting ZEB1 from miR-141-3p-mediated silencing in hepatocellular carcinoma. Cell Death Dis. 2018;9(3):387.
74. Tong SJ, Zhang XY, Guo HF, Yang J, Qi YP, Lu S. Study on effects of miR-141-3p in proliferation, migration, invasion and apoptosis of colon cancer cells by inhibiting Bcl2. Clin Transl Oncol. 2021.
75. Wang D, Luo Y, Wang G, Yang Q. CircATRNL1 promotes epithelial-mesenchymal transition in endometriosis by upregulating Yes-associated protein 1 in vitro. Cell Death Dis. 2020;11(7):594.
76. Dasgupta P, Kulkarni P, Majid S, Hashimoto Y, Shiina M, Shahryari V, et al. LncRNA CDKN2B-AS1/miR-141/cyclin D network regulates tumor progression and metastasis of renal cell carcinoma. Cell Death Dis. 2020;11(8):660.
77. Cui Z, An X, Li J, Liu Q, Liu W. LncRNA MIR22HG negatively regulates miR-141-3p to enhance DAPK1 expression and inhibits endometrial carcinoma cells proliferation. Biomed Pharmacother. 2018;104:223-228.
78. Yu KR, Lee S, Jung JW, Hong IS, Kim HS, Seo Y, et al. MicroRNA-141-3p plays a role in human mesenchymal stem cell aging by directly targeting ZMPSTE24. J Cell Sci. 2013;126(Pt 23):5422-31.
79. Wang C, Ouyang Y, Lu M, Wei J, Zhang H. miR-141-3p regulates the expression of androgen receptor by targeting its 3'UTR in prostate cancer LNCaP cells. Xi Bao Yu Fen Zi Mian Yi Xue Za Zhi. 2015;31(6):736-9.
80. Dong S, Ma M, Li M, Guo Y, Zuo X, Gu X, et al. LncRNA MEG3 regulates breast cancer proliferation and apoptosis through miR-141-3p/RBMS3 axis. Genomics. 2021;113(4):1689-1704.
81. Sun Z, Shao B, Liu Z, Dang Q, Guo Y, Chen C, et al. LINC01296/miR-141-3p/ZEB1-ZEB2 axis promotes tumor metastasis via enhancing epithelial-mesenchymal transition process. J Cancer. 2021;12(9):2723-2734.
82. Zhou D, Gu J, Wang Y, Wu H, Cheng W, Wang Q, et al. Long non-coding RNA NEAT1 transported by extracellular vesicles contributes to breast cancer development by sponging microRNA-141-3p and regulating KLF12. Cell Biosci. 2021;11(1):68.
83. Wang Y, Ren F, Sun D, Liu J, Liu B, He Y, et al. CircKEAP1 Suppresses the Progression of Lung Adenocarcinoma via the miR-141-3p/KEAP1/NRF2 Axis. Front Oncol. 2021;11:672586.
84. Yang Y, Xiao C, Liu K, Song L, Zhang Y, Dong B. Silencing of long noncoding INHBA antisense RNA1 suppresses proliferation, migration, and extracellular matrix deposition in human hypertrophic scar fibroblasts via regulating microRNA-141-3p/myeloid cell leukemia 1 axis. Bioengineered. 2021;12(1):1663-1675.
85. Zhang C, Cao J, Lv W, Mou H. CircRNA_100395 Carried by Exosomes From Adipose-Derived Mesenchymal Stem Cells Inhibits the Malignant Transformation of Non-Small Cell Lung Carcinoma Through the miR-141-3p-LATS2 Axis. Front Cell Dev Biol. 2021;9:663147.
86. Yang Q, Zheng M, Yan J, Wu J, Liu X. Rhynchophylline improves trophocyte mobility potential by upregulating ZEB1 level via the inhibition of miR-141-3p level. Biosci Biotechnol Biochem. 2021;85(2):280-286.
87. Xu J, Xie G, Yang W, Wang W, Zuo Z, Wang W. Platelet-rich plasma attenuates intervertebral disc degeneration via delivering miR-141-3p-containing exosomes. Cell Cycle. 2021;20(15):1487-1499.
88. Cheng X, Tian P, Zheng W, Yan X. Piplartine attenuates the proliferation of hepatocellular carcinoma cells via regulating hsa_circ_100338 expression. Cancer Med. 2020;9(12):4265-4273.
89. Zhang F, Cheng N, Du J, Zhang H, Zhang C. MicroRNA-200b-3p promotes endothelial cell apoptosis by targeting HDAC4 in atherosclerosis. BMC Cardiovasc Disord. 2021;21(1):172.
90. Xia L, Zhu G, Huang H, He Y, Liu X. LncRNA small nucleolar RNA host gene 16 (SNHG16) silencing protects lipopolysaccharide (LPS)-induced cell injury in human lung fibroblasts WI-38 through acting as miR-141-3p sponge. Biosci Biotechnol Biochem. 2021;85(5):1077-1087.
91. Guo W, Mu K, Zhang B, Sun C, Zhao L, Li HR, et al. The circular RNA circ-GRB10 participates in the molecular circuitry inhibiting human intervertebral disc degeneration. Cell Death Dis. 2020;11(8):612.
92. Saha S, Chakraborty S, Bhattacharya A, Biswas A, Ain R. MicroRNA regulation of Transthyretin in trophoblast differentiation and Intra-Uterine Growth Restriction. Sci Rep. 2017;7(1):16548.
93. Miyazawa M, Bogdan AR, Hashimoto K, Tsuji Y. Regulation of transferrin receptor-1 mRNA by the interplay between IRE-binding proteins and miR-7/miR-141 in the 3'-IRE stem-loops. RNA. 2018;24(4):468-479.
94. Li X, Tian Z, Jin H, Xu J, Hua X, Yan H, et al. Decreased c-Myc mRNA Stability via the MicroRNA 141-3p/AUF1 Axis Is Crucial for p63α Inhibition of Cyclin D1 Gene Transcription and Bladder Cancer Cell Tumorigenicity. Mol Cell Biol. 2018;38(21):e00273-18.
95. Papageorgiou I, Court MH. Identification and validation of microRNAs directly regulating the UDP-glucuronosyltransferase 1A subfamily enzymes by a functional genomics approach. Biochem Pharmacol. 2017;137:93-106.
96. Huang XY, Huang ZL, Zhang PB, Huang XY, Huang J, Wang HC, et al. CircRNA-100338 Is Associated With mTOR Signaling Pathway and Poor Prognosis in Hepatocellular Carcinoma. Front Oncol. 2019;9:392.
97. Chao F, Song Z, Wang S, Ma Z, Zhuo Z, Meng T, et al. Novel circular RNA circSOBP governs amoeboid migration through the regulation of the miR-141-3p/MYPT1/p-MLC2 axis in prostate cancer. Clin Transl Med. 2021;11(3):e360.
98. Zhang L, Qi M, Feng T, Hu J, Wang L, Li X, et al. IDH1R132H Promotes Malignant Transformation of Benign Prostatic Epithelium by Dysregulating MicroRNAs: Involvement of IGF1R-AKT/STAT3 Signaling Pathway. Neoplasia. 2018;20(2):207-217.
99. Xia L, Chen X, Yang J, Zhu S, Zhang L, Yin Q, et al. Long Non-Coding RNA-PAICC Promotes the Tumorigenesis of Human Intrahepatic Cholangiocarcinoma by Increasing YAP1 Transcription. Front Oncol. 2021;10:595533.
